# Design and Synthesis of Thymol Derivatives Bearing a 1,2,3-Triazole Moiety for Papaya Protection against Fusarium solani

**DOI:** 10.1021/acs.jafc.4c12770

**Published:** 2025-06-03

**Authors:** Mariana Belizário de Oliveira, Poliana Aparecida Rodrigues Gazolla, Leandra Martins Meireles, Róbson Ricardo Teixeira, Danilo Aniceto da Silva, Luiz Claudio Almeida Barbosa, Pedro Alves Bezerra Morais, Osmair Vital de Oliveira, Claudia Jorge do Nascimento, Pedro Henrique de Andrade Barrela, Jochen Junker, Nayara Araujo dos Santos, Wanderson Romão, Valdemar Lacerda, Waldir Cintra de Jesus Júnior, Eduardo Seiti Gomide Mizubuti, Vagner Tebaldi de Queiroz, Demetrius Profeti, Willian Bucker Moraes, Rodrigo Scherer, Adilson Vidal Costa

**Affiliations:** † Departamento de Química e Física, Grupo de Pesquisa de Estudos Aplicados em Produtos Naturais e Síntese Orgânica (GEAPS), Universidade Federal do Espírito Santo, Alto Universitário, s/n, 29500-000 Alegre, Espírito Santo State, Brazil; ‡ Universidade de Vila Velha, Departamento de Farmácia, Programa de Pós-Graduação em Ciências Farmacêuticas, Av. Comissário José Dantas de Melo 21, 29102-770 Vila Velha, Espírito Santo State, Brazil; § Departamento de Química, Grupo de Síntese e Pesquisa de Compostos Bioativos (GSPCB), Universidade Federal de Viçosa, Av. P.H. RolFs s/n, 36570-900 Viçosa, Minas Gerais State, Brazil; ∥ Departamento de Química, 28114 Universidade Federal de Minas Gerais, Av. Antonio Carlos 6627, 31270-901 Belo Horizonte, Minas Gerais State, Brazil; ⊥ Instituto Federal de São Paulo, Campus Catanduva, 15808-305 Catanduva, SP, Brazil; # Departamento de Ciências Naturais, Instituto de Biociências, 89111 Universidade Federal do Estado do Rio de Janeiro (UNIRIO), Av. Pauster, 22290-240 Rio de Janeiro, Rio de Janeiro State, Brazil; ¶ Centro de Desenvolvimento Tecnológico em Saúde, Fundação Oswaldo Cruz, Av. Brasil 4365, 21040-900 Rio de Janeiro, RJ, Brazil; ∇ Laboratório de Petroleômica e Forense, Departamento de Química, Universidade Federal do Espírito Santo, Av. Fernando Ferrari 514, 29075-910 Vitória, ES, Brazil; ○ Universidade Federal de São Carlos, Campus Lagoa do Sino, 18290-000 Buri, São Paulo State, Brazil; ⧫ Departamento de Fitopatologia, Universidade Federal de Viçosa, Av. P.H. RolFs s/n, 36570-900 Viçosa, Minas Gerais State, Brazil; †† Programa de Pós-Gradução em Agroquímica, Universidade Federal do Espírito Santo, Alto Universitário, s/n, 29500-000 Alegre, Espírito Santo State, Brazil; ‡‡ Programa de Pós-Gradução em Agronomia, Universidade Federal do Espírito Santo, Alto Universitário, s/n, 29500-000 Alegre, Espírito Santo State, Brazil

**Keywords:** Fusarium solani, fungicide activity, 1,2,3-triazole, papaya, molecular docking, thymol

## Abstract

Azole-based fungicides are among the market’s most widely used and effective agents. However, their indiscriminate use can lead to reduced efficacy and increased pathogen resistance. This highlights the need for novel fungicides that offer improved efficiency and lower environmental impact for controlling phytopathogenic fungi. In this study, a series of 20 novel thymol derivatives, incorporating a 1,2,3-triazole moiety, were synthesized via a three-step process, with the key step being the copper­(I)-catalyzed azide–alkyne cycloaddition (CuAAC) reaction. The antifungal activity of these compounds was evaluated against Fusarium solani, the etiological agent of papaya fruit and stem rot. Additionally, molecular docking was performed to assess the binding energy and interaction modes of these derivatives with the F. solani lanosterol 14α-demethylase (*Fs*CYP51) enzyme. Docking results demonstrated that all derivatives bound to the catalytic pocket of *Fs*CYP51 with lower binding energy (<−10 kcal/mol) compared to the azole fungicide tebuconazole (−8.2 kcal/mol) and the substrate lanosterol (−9.0 kcal/mol). The observed fungicidal activity is likely due to the occupancy of the entrance tunnel and active site of the *Fs*CYP51 by these derivatives, thereby blocking lanosterol and its conversion into ergosterol.

## Graphical abstract

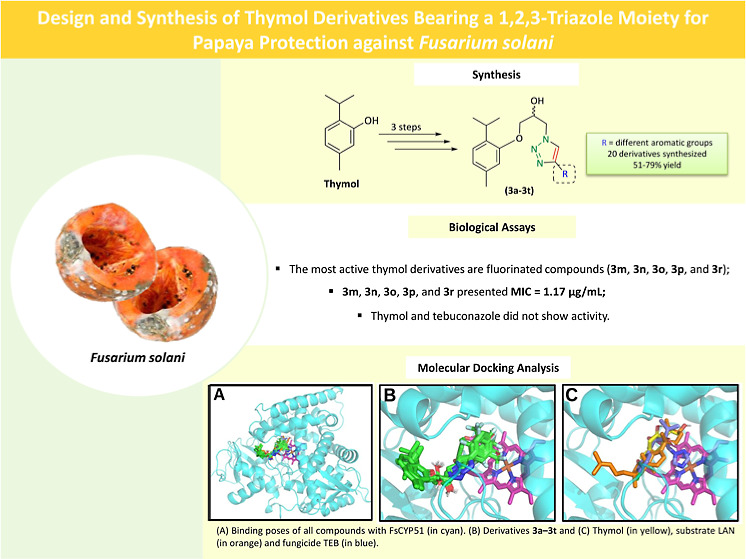

## Introduction

Agriculture is a cornerstone of economic development, particularly through the production of export goods.^1^ In recent years, fruit farming has experienced steady growth, significantly contributing to income generation by cultivating a wide variety of species.^2^ For instance, the Food and Agriculture Organization of the United Nations reported that the global trade volume of major tropical fruits (pineapple, avocado, papaya, and the commodity cluster composed of mango, mangosteen, and guava) reached a new peak of USD 11.2 billion in 2023, marking a 12% increase compared to 2022.^2^

Papaya is a fruit that holds substantial economic value. Brazil, Mexico, Costa Rica, and the Dominican Republic are the leading producers and exporters of papaya in Latin America, generating significant income and fostering economic growth within these countries.^3^ Brazil, known for its favorable soil and climatic conditions, ranks as the second-largest papaya producer in the world.^4^ The papaya tree, which belongs to the Caricaceae family, is native to the American continent and thrives in tropical and subtropical climates.^5^ In addition to its commercial importance, papaya is recognized for its high nutritional and medicinal value, with studies demonstrating its use against gastrointestinal disorders, bacterial infections, fever, and asthma.^6–8^

Papaya producers are well aware of the fruit’s high deterioration rate, primarily due to severe infections by various pathogens that thrive both on the fruit surface and internally during postharvest handling.^9^ These postharvest decays are irreversible, leading to significant declines in overall fruit quality and substantially increasing loss rates throughout the postharvest supply chain in papaya-producing countries.^10^

Among the various pathogens that afflict papaya,^9^ Fusarium solani is one of the most common. This species is frequently observed in rotten fruits, producing spreading colonies with cottony, woolly, flat, or fluffy aspects, resulting in small lesions and depression on the fruit, that diminish the fruit́s marketability.^9^

Traditional postharvest management of F. solani relies heavily on synthetic chemical fungicides.^11^ However, the overuse of these products threatens both their efficacy and environmental sustainability while also contributing to the development of pathogen resistance.^12,13^

A viable alternative to address the challenges mentioned above is the development of new agrochemicals derived from natural products.^14–16^ These products have served as a significant source of bioinspiration for laboratories and industries, leading to the discovery of innovative agents for weed, plant pathogens, and insect pest control. Natural products have played a crucial role in advancing crop protection research and uncovering novel biological mechanisms and modes of action. Notable examples of agrochemicals inspired by natural products include pyrethroids,^15,16^ neonicotinoids,^15–17^ and strobilurins.^15–18^

Thymol is a natural monoterpenoid phenol, characterized by its crystalline, colorless appearance and distinct aroma. Chemically, it is known as 2-isopropyl-5-methylphenol and is a major component of the essential oils of thyme (Thymus vulgaris L.) and oregano (Origanum vulgare L.). Thymol has also been isolated from various other plants, including Ocimum gratissimum L., Origanum L., Trachyspermum ammi (L.), species of the genera Satureja L. and Monarda L. (Lamiaceae), Carum copticum L., Oliveria decumbens Vent (Apiaceae), Anemopsis californica (Saururaceae), and species from the Verbenaceae, Scrophulariaceae, and Ranunculaceae families. This versatile molecule has numerous practical applications, across fields such as medicine, dentistry, veterinary science, food production, and agrochemicals. Its pharmacological properties, particularly its antimicrobial, antioxidant, anti-inflammatory, and wound-healing effects have been researched and documented.^19^

Thymol also demonstrates significant fungicidal activity, especially against fluconazole-resistant strains and plant-pathogenic fungi. Its antifungal mechanism disrupts the hyphal structure, causing aggregation, reduced hyphal diameters, and eventual lysis of the hyphal wall. Additionally, thymol’s hydrophobicity enables it to penetrate fungal cell membranes, disrupting pH balance and compromising cellular integrity. Given its potent antifungal effects, thymol shows promise as a valuable compound for research aimed at developing new chemical agents to control fungal species.^20,21^

Azoles, a class of 14α-demethylase inhibitors, have remained the primary choice for managing fungal diseases in plants for over 40 years. With more than 25 azole compounds now available for crop disease control, these fungicides represent approximately 20–25% of the global market. They are recognized for their enduring efficacy against numerous plant pathogens, with only a moderate risk of resistance development. Field results have generally shown sustained or minimally diminished effectiveness over time. Thus, azoles continue to play a central role in many diseases management programs. They are frequently used on their own or in combination with other fungicide classes to expand the control range and mitigate the risk of resistance.^22^ The azole class includes 1,2,3- and 1,2,4-triazoles, which are synthetic isomers with extensive industrial applications and a broad spectrum of biological activities, such as antimalarial, anticancer, antiviral, antileishmanial, antibacterial, and antifungal.^23–29^

Our research group has used phenolic natural compounds as starting materials to synthesize 1,2,3-triazole derivatives to control fungal phytopathogens. A series of 11 fluorinated eugenol-derived triazoles was synthesized, and their in vitro inhibitory activity against the mycelial growth of a Colletotrichum sp. strain, responsible for anthracnose in papaya fruits, was evaluated. The most promising result was obtained for the compound 1-(4-allyl-2-methoxyphenoxy)-3-(4-(2-fluorophenyl)-1*H*-1,2,3-triazol-1-yl)­propan-2-ol, which exhibited a mean growth inhibition zone of 5.10 mm in a well-diffusion assay (EC_50_ = 1.50 mg mL^–1^; MIC = 1.00–2.50 mg mL^–1^). In a more recent study, eugenol was transformed into 19 1,2,3-triazole-containing derivatives, which were screened against Colletotrichum gloeosporioides. At a concentration of 100 ppm, the derivatives 4-((4-allyl-2-methoxyphenoxy)­methyl)-1-(2-fluorophenyl)-1*H*-1,2,3-triazole, 4-((4-allyl-2-methoxyphenoxy)­methyl)-1-(2-(trifluoromethyl)­phenyl)-1*H*-1,2,3-triazole, 4-((4-allyl-2-methoxyphenoxy)­methyl)-1-(4-(trifluoromethyl)­phenyl)-1*H*-1,2,3-triazole, and 4-((4-allyl-2-methoxyphenoxy)­methyl)-1-(3-(trifluoromethyl)­phenyl)-1*H*-1,2,3-triazole were the most effective, reducing mycelial growth by 88.3%, 85.5%, 82.4%, and 81.4%, respectively. Furthermore, molecular docking studies provided insight into the binding mode of these derivatives within the catalytic pocket of C. gloeosporioides CYP51.^31^ As part of our ongoing efforts to discover and develop more effective antifungal agents, we report the synthesis of 20 new thymol-based derivatives incorporating 1,2,3-triazole moieties. These compounds were evaluated for their fungicidal activity against F. solani, a significant pathogen affecting papaya cultivation.^30,32^

## Materials and Methods

### Chemicals and Instrument

Reagents and high-purity solvents were procured from Sigma-Aldrich (St. Louis, MO, USA) and Êxodo Científica (Sumaré, SP, Brazil), respectively. Thin-layer chromatography (TLC) was conducted on aluminum-backed, silica gel-precoated plates with various solvent systems. TLC plates were visualized under ultraviolet (UV) light and/or by staining with a phosphomolybdic acid solution. Column chromatography separations used silica gel (70–230 mesh, Sigma-Aldrich) as the stationary phase. Melting temperatures were determined with an MA-381 device (Marconi, São Paulo, Brazil) and are reported as uncorrected values.

Infrared (IR) spectra were recorded using a Tensor 27 device (Bruker, Bremen, Germany), with the attenuated total reflection (ATR) technique, scanning from 4000 to 500 cm^–1^. Nuclear magnetic resonance spectra for hydrogen (^1^H NMR) and carbon (^13^C NMR) were obtained on a Varian Mercury 300 spectrometer (Varian, Palo Alto, CA, USA) and Bruker AVANCE III 400 spectrometer (Bruker, Billerica, MA, USA) at frequencies of 300 or 400 MHz, respectively for ^1^H and 75 or 100 MHz, respectively for ^13^C, using hexadeutero dimethyl sulfoxide (DMSO-*d*_6_) as solvent. NMR data are presented as chemical shift (δ) in parts per million (ppm), multiplicity, the number of hydrogens, and coupling constants (*J*) values in Hertz (Hz). Multiplicities are indicated by the abbreviations: s (singlet), d (doublet), dap (apparent doublet), dd (double of doublets), t (triplet), tap (apparent triplet), td (triplet of doublets), q (quartet), ddtap (apparent doublet of doublets of triplets), sept (septet) and m (multiplet).

Mass spectra were acquired on a Vanquish Flex chromatography (Thermo Scientific, Bremen, Germany) coupled to an LTQ-XL mass spectrometer (Thermo Scientific, Bremen, Germany). Chromatographic separation was performed using a C18 column of 100 Å, 150 × 2.1 mm, Luna Omega 1.6 μm (Phenomenex, São Paulo, Brazil), with a 2 μL sample injection and a flow rate of 350 μL/min. The gradient ranged from 5% to 95% over 7 min at 60 °C, utilizing water and methanol with 0.1% formic acid as mobile phases. The mass spectrometer was set to operate in a mass range of 100–1500 Da. Electrospray ionization (ESI) conditions included a heater temperature of 350 °C, sheath gas flow rate 30 arb, auxiliary gas flow rate of 10 arb, spray voltage of 4.0 kV, and capillary voltage of 44.0 kV.

### Synthetic Procedures

#### Synthesis of (±)-2-((2-isopropyl-5-methylphenoxy)­methyl)­oxirano (**1**)

Thymol (1.50 g, 9.98 mmol), epichlorohydrin (4.62 g; 49.9 mmol), potassium hydroxide (1.40 g; 25.0 mmol), and tetrabutylammonium bromide (0.640 g; 1.99 mmol) were added to a 50 mL round-bottom flask containing a magnetic stir bar. The reaction mixture was stirred at 0 °C for 30 min and then at room temperature for 3 h. The reactiońs progress was monitored by TLC analysis and distilled water (15.0 mL) was added upon its completion. Then, aqueous phase was extracted with dichloromethane (3 × 30.0 mL). The combined organic layers were washed with a saturated sodium chloride solution, dried over anhydrous sodium sulfate, filtered, and concentrated under reduced pressure. The resulting residue was purified by silica gel column chromatography to yield compound **1** as a yellow oil with a 65% yield (1.34 g, 4.00 mmol).

#### Synthesis of (±)-1-Azido-3-(2-isopropyl-5-methylphenoxy)­propan-2-ol (**2**)

To a 100 mL round-bottom flask, compound **1** (1.00 g; 4.84 mmol), sodium azide (1.58 g; 24.2 mmol), ammonium chloride (0.648 g; 12.1 mmol), methanol (4.00 mL), and distilled water (1.00 mL) were added. The reaction mixture was stirred under reflux at 60 °C for 3 h, and during this period, its progress was monitored by TLC analysis. Upon completion, distilled water (15.0 mL) was added, and the aqueous phase was extracted with dichloromethane (3 × 30.0 mL). The combined organic layers were washed with a saturated sodium chloride solution, dried over anhydrous sodium sulfate, filtered, and concentrated under reduced pressure. The crude product was purified by silica gel column chromatography affording compound **2** as a yellow oil in 94% yield (1.14 g, 4.56 mol).

#### General Procedure for the Synthesis of 1,2,3-Triazole Compounds (**3a**–**3t**)

To a 50 mL round-bottom flask, azide (**2**) (1.00 equiv), terminal alkyne (1.20 equiv), sodium ascorbate (0.400 equiv), CuSO_4_·5H_2_O (0.400 equiv) were combined in a solvent mixture of distilled water and dichloromethane (1:1 v/v, 8.0 mL). The reaction mixture was stirred at room temperature for 1 h, until completion as revealed by TLC analysis. Then, distilled water (15.0 mL) was added, and the aqueous phase was extracted with dichloromethane (3 × 30.0 mL). The combined organic layers were washed with a saturated solution of ethylenediamine tetraacetic acid (EDTA), dried over anhydrous sodium sulfate, filtered, and concentrated under reduced pressure. The crude products were purified by silica gel column chromatography. Spectroscopic and physical data for all compounds are provided in the Supporting Information.

### Biological Assays

#### Microorganisms

The filamentous fungi F. solani ATCC 40099 was selected to assess the antimicrobial activity of newly synthesized triazole compounds.

#### Determination of Antifungal Activity

The assay followed the CLSI M38-A2 gold-standard protocol for susceptibility testing.^33^ The fungi were cultured on Potato Dextrose Agar (PDA) for 7 days at 35 °C. A spore suspension was prepared in saline solution (0.85% w/v) with 0.1% (w/v) Tween 20 and adjusted with a spectrophotometer to an optical density between 0.08 and 0.13 at 530 nm. This suspension was then diluted in RPMI-1640 to achieve a concentration of 0.4 × 10^4^ to 5 × 10^4^ CFU/mL. Tebuconazole, thiabendazole, thymol, and 20 new thymol-derived triazole compounds were tested at concentrations ranging from 300 μg/mL to 0.58 μg/mL. The assays were performed in duplicate in 96-well microplates. After 48 h of incubation, 15 μL of 0.01% (w/v) resazurin dye was added to each well, followed by a further 4 h reincubation at 35 °C. The Minimum Inhibitory Concentration (MIC) was determined by a color change in the dye from blue (indicating growth) to pink (indicating no growth). The lowest concentration with no visible growth compared to the control well was considered the MIC.

#### Minimum Fungicidal Concentration (MFC)

The minimum fungicidal concentration (MFC) was determined for microorganisms exhibiting MIC values below 9.3 μg/mL for the new thymol-derived triazoles. From the wells with MIC, 2 × MIC, and 4 × MIC concentrations, a 20 μL sample was transferred to Sabouraud Dextrose Agar (SDA) plates. The sample was spread over the surface of the medium surface using a Drigalski spatula and incubated at 35 °C for 48 h. The MFC was defined as the lowest concentration at which no visual colony growth after incubation (or up to 3 CFUs) was observed, following the criteria established by Espinel-Ingroff et al.^34^

#### Molecular Docking

The 3D homology structure of lanosterol 14α-demethylase (*Fs*CYP51) from F. solani was generated using the SWISS-MODEL server.^35^ The amino acid sequence was obtained from the NCBI database (http://www.ncbi.nlm.nih.gov accessed on October 03, 2024) with reference sequence number of KAJ4231894.1. The crystal structure of fungi Aspergillus fumigatus CYP51B (PDB ID 4UYM),^36^ which shares 59.9% sequence identity with *Fs*CYP51, served as the template for model building. Vermeulen et al. (2022) also used the PDB ID 5EQB as template to build the 3D structure of the F. solani CYP51, although this template shares only 45.1% enzymes present sequence similarity with *Fs*CYP51.^37^ For the ligands, Avogadro software (Avogadro: an open-source molecular builder and visualization tool. Version 1.93.0. http://avogadro.cc/) was used to generate the initial structures of thymol derivatives based on the 1,2,3-triazoles (**3a**–**3t**), as well as thymol, lanosterol, and tebuconazole molecules. These initial structures were optimized using the MOPAC2016 package^38^ with the semiempirical Hamiltonian PM7 method.^39^ The generated PDB files were then converted to PDBQT format using the OBABEL program (O’Boyle et al. 2011).^40^ For docking calculations, the active site and substrate access tunnel region of the *Fs*CYP51 enzyme model were selected. A cubic grid box with a 34 Å edge length and a grid spacing of 1 Å, centered at coordinates 5.088 × −6.378 × 0.51 was used. The maximum number of binding models was set to 20, with an exhaustiveness parameter of 8 for each compound. Ligand conformations were treated as flexible, while the CYP51A receptor remained rigid. Notably, this same protocol was recently applied to dock other 1,2,3-triazole derivatives into *Fs*CYP51 enzyme. Docking calculations were performed using the AutoDock Vina package (Trott & Olson, 2010). Results were analyzed with PyMOL software version 2.0 (The PyMOL Molecular Graphics System, Version 2.0 Schrödinger, LLC.) and Discovery Studio (DS) Visualizer 21.1.0.20298 (https://discover.3ds.com/discovery-studio-visualizer-download).

## Results and Discussion

### Chemistry

The synthetic steps for preparing thymol derivatives containing 1,2,3-triazole moieties (**3a–3t**) are illustrated in Scheme 1. Initially, commercially available thymol underwent structural modification in the presence of (±)-epichlorohydrin, tetrabutylammonium bromide (Bu)_4_NBr), and potassium hydroxide, yielding epoxide (**1**) with a 65% yield.^41^ Next, azido-alcohol (**2**) was synthesized with a 94% yield through the reaction of epoxide (**1**) with sodium azide in the presence of ammonium chloride (NH_4_Cl).^42,43^ Compound (**2**) was further subjected to copper­(I)-catalyzed azide–alkyne cycloaddition (CuAAC) reactions with various terminal alkynes affording the desired thymol-triazole derivatives (**3a**–**3t**) in yields of 51% to 79%.^44–49^

**Scheme 1:**
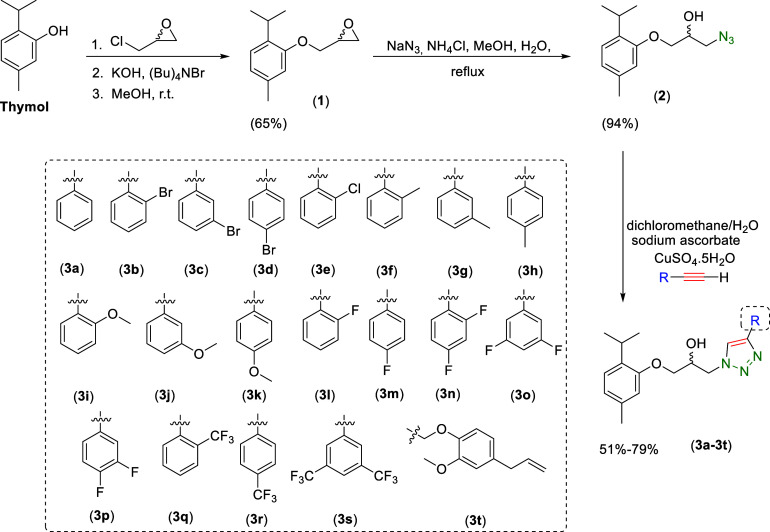
Synthetic Route for the Conversion of Thymol to 1,2,3-Triazole Derivatives **3a–3t**

All compounds **3a**–**3t** were characterized using ^1^H and ^13^C nuclear magnetic resonance (NMR) spectroscopy, infrared (IR) spectroscopy, and mass spectrometry. All expected bands corresponding to the functional groups were confirmed in the IR spectra. The band in the 1607–1620 cm^–1^ region was attributed to the N=N bond stretching, characteristic of the 1,2,3-triazole ring structure. In the ^1^H NMR spectra, the hydroxyl hydrogen appeared as a doublet between 5.56 and 5.69 ppm, while the hydrogen associated with the triazole ring was observed as a singlet or doublet within the 7.76–9.00 ppm range. For the ^13^C NMR, the chemical shifts and number of signals were consistent with compound structures, with the triazole ring carbons appearing between 121.6 and 127.7 ppm. Finally, the molecular formulas of the triazole derivatives were confirmed by LTQ-XL. Full spectroscopic data used for compound characterization is available in the Supporting Information.

Once synthesized and characterized, the antifungal activity of the new thymol derivatives (**3a**–**3t**) against F. solani was evaluated.

## Biological Assays

Table 1 presents the inhibitory activity of the new triazole molecules against the filamentous fungus F. solani ATCC 40099. Most compounds demonstrated strong activity, indicated by low MIC values. Growth inhibition occurred within the range of 1.17 to 37.5 μg/mL, with the lowest values observed for compounds **3m**, **3n**, **3o**, **3p**, and **3r** (1.17 μg/mL). As a general trend, compounds containing phenyl rings with electron-donating groups (methyl or methoxy) did not display activity against F. solani. Exception to this generalization is **3f** (MIC = 9.3 μg/mL). It is also noteworthy that the most active thymol derivatives—**3m**, **3n**, **3o**, **3p**, and **3r**—are fluorinated compounds. As pointed out by Jeschke,^50^ the incorporation of halogens, particularly fluorine atoms or fluorinated groups, into active ingredients (a.i.s) remains a valuable approach in developing agricultural products that balance effectiveness, environmental safety, user convenience, and cost-efficiency. In this study, the incorporation of fluorine and the trifluoromethyl group produced compounds with notable antifungal effect. It is worth mentioning that fluorinated compounds or those containing fluorine motifs make up approximately 20% of available agrochemicals.^51^ Notably, due to complex structure–activity relationships of biologically active molecules, fluorine substitution can either enhance or diminish a compound’s efficacy. This variation arises from changes in the compound́s mode of action, physicochemical properties, target binding, or susceptibility to metabolic transformation. As a result, predicting the optimal sites for fluorine substitution within a molecule to achieve the desired effects remains challenging.^52^

**Table 1:** Antifungal Activity of Derivatives, Thymol, Tebuconazole, and Thiabendazole against Fusarium solani ATCC 4099

compound	R group^a^	MIC (μg/mL)
**3a**	phenyl	37.5
**3b**	2-bromophenyl	9.3
**3c**	3-bromophenyl	4.6
**3d**	4-bromophenyl	18.7
**3e**	2-chlorophenyl	37.5
**3f**	2-methylphenyl	9.3
**3g**	3-methylphenyl	
**3h**	4-methylphenyl	
**3i**	2-methoxyphenyl	
**3j**	3-methoxyphenyl	
**3k**	4-methoxyphenyl	
**3l**	2-fluorophenyl	9.37
**3m**	4-fluorophenyl	1.17
**3n**	2,4-difluorophenyl	1.17
**3o**	3,5-difluorophenyl	1.17
**3p**	3,4-difluorophenyl	1.17
**3q**	2-(trifluoromethyl)phenyl	
**3r**	4-(trifluoromethyl)phenyl	1.17
**3s**	3,5-bis(trifluoromethyl)phenyl	
**3t**	(4-allyl-2-methoxyphenoxy)methyl	75.0
**thymol**		
**tebuconazole**		
**thiabendazole**		7.00

a It corresponds to the R groups attached to the 1,2,3-triazole ring in Scheme 1. (−) No activity.

Triazole compounds inhibit lanosterol 14α-demethylase, a crucial enzyme in the ergosterol biosynthesis pathway. The binding of triazoles to this enzyme involves the interaction of the triazole ring with the heme iron in the active site, as well as additional interactions with amino acid residues along the enzyme’s side chain. This binding decreases ergosterol production and leads to the accumulation of toxic intermediates, such as 14-methyl-3,6-diol ((3*S*,6*S*,10*S*,13*R*,14*R*)-17-(5,6-dimethylheptan-2-yl)-10,13,14-trimethyl-2,3,4,5,6,7,10,11,12,13,14,15,16,17-tetradecahydro-1*H*-cyclopenta­[*a*]­phenanthrene-3,6-diol)). The incorporation of this intermediate into the fungal cell membrane compromises its structural integrity, thereby inhibiting the growth of the microorganism.^53^

The literature reports a low sensitivity of F. solani to triazole compounds, which is attributed to the microorganism’s intrinsic resistance. This resistance is linked to mutations in the CYP51a gene, particularly the substitution of leucine at position 218 (L218), which alters the entry of triazoles into the channel of the lanosterol 14α-demethylase enzyme.^54,55^ In the present study, however, the observed effects on F. solani may be due to the unique molecular structure of the new triazoles, which may circumvent these intrinsic enzyme modifications, effectively inhibiting the enzyme and preventing fungal growth. Notably, compounds **3m**, **3n**, **3o**, **3p**, and **3r** emerged as promising candidates for antifungal development with potential applications in both agricultural and medical applications.

The fungicidal activity of the new compounds, as well as of the positive controls thiabendazole and tebuconazole, was also evaluated. Although the new triazole molecules exhibited potent activity against F. solani, they did not demonstrate fungicidal activity but fungistatic activity, similar to the controls, thiabendazole and tebuconazole. Fungistatic activity is characterized by the continued growth of the microorganism following the minimum fungicidal concentration (MFC) assay. This result is attributed to the intrinsic characteristics of the microorganisms and the triazole compounds.

### Molecular Docking Analysis

Although there are currently no commercial antifungal agents based on 1,2,3-triazole derivatives, this scaffold has been extensively investigated in the literature due to the ease of its synthesis via click chemistry and its demonstrated fungicidal activity. Moreover, it is well established that all azole-based drugs share a common mechanism of action involving the inhibition of lanosterol 14α-demethylase (CYP51), thereby disrupting ergosterol biosynthesis.^56,57^ Very recently, Wang and coauthors (2025) provided compelling evidence that a 1,2,3-triazole derivative interacts with the CYP51 enzyme, as shown through chemical proteomics and chemicobiological studies involving C. gloeosporioides in mango.^58^ In view of that, we propose that 1,2,3-triazoles likely operate via the same mechanism as commercial azole derivatives (e.g., imidazoles and 1,2,4-triazoles).

In this study, an in silico analysis based on the molecular docking method was performed to determine the binding mode of the compounds **3a**–**3t** within the *Fs*CYP51 enzyme of the filamentous fungus F. solani. This analysis is crucial for understanding the mechanism of action and the role of the key residues involved in protein–ligand interactions. *Fs*CYP51 was selected as the target because it plays a central role in ergosterol biosynthesis, an essential component of the cell membrane. Inhibition of *Fs*CYP51 with 1,2,3-triazole derivatives disrupts ergosterol formation, leading to fungal cell death. Furthermore, F. solani exhibited susceptibility to most of the derivatives, as shown in Table 1. The docking calculations reveal that all compounds exhibit binding affinities within the *Fs*CYP51 binding site, as illustrated in Figure 1.

**Figure 1:**
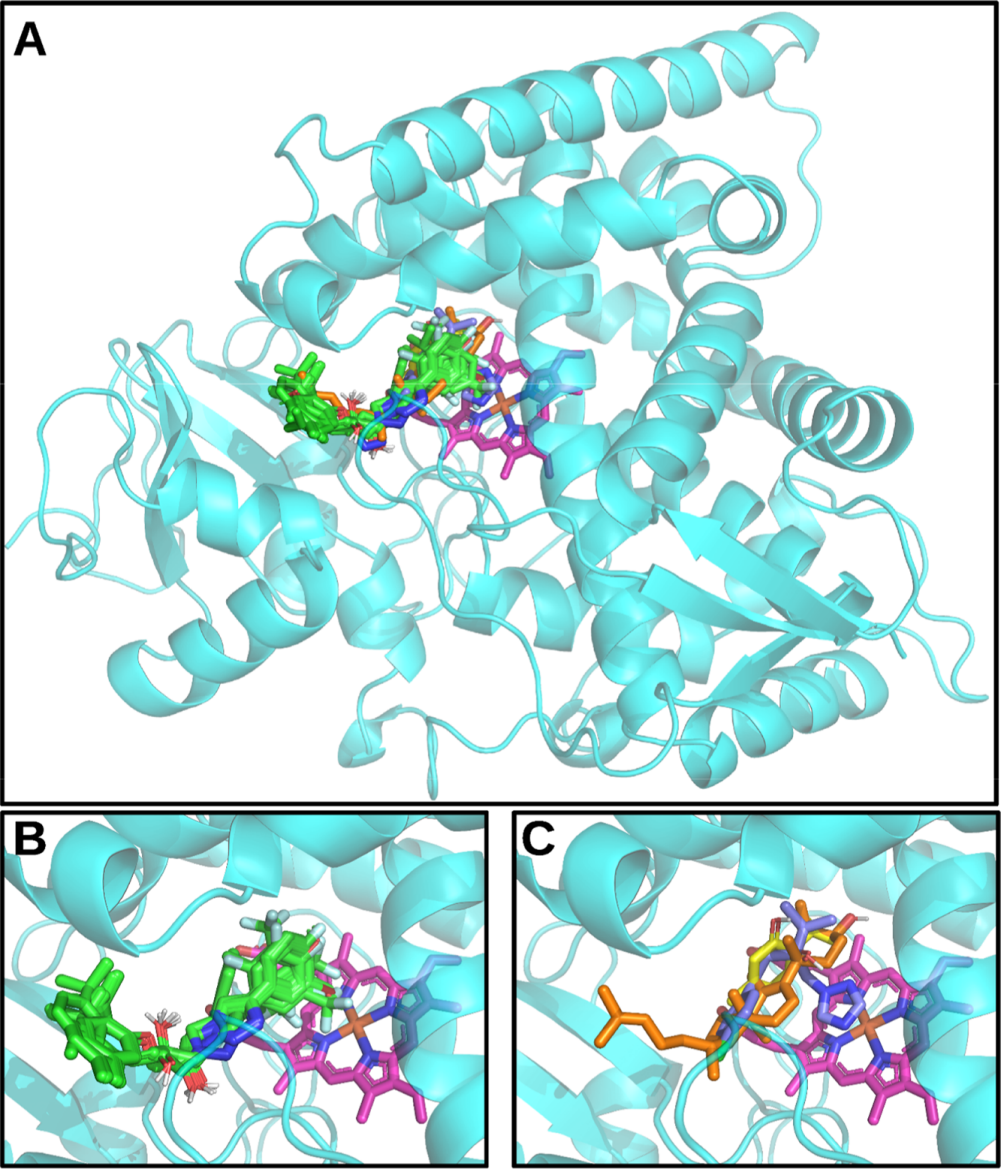
(A) Binding poses of all compounds with *Fs*CYP51 (in cyan). Zoom of the *Fs*CYP51 active site complexed with compounds: (B) Derivatives **3a**–**3t** and (C) Thymol (in yellow), substrate LAN (in orange) and fungicide TEB (in blue). The heme group is in magenta color.

As expected, both the substrate lanosterol (LAN) and fungicide tebuconazole (TEB) bind closely with the heme group (Figure 1C). Similarly, all 1,2,3-triazole derivatives docked in the region occupied by LAN, TEB, and thymol (see Figure 1A–C). The derivatives **3a–3t** exhibit similar docking conformations within the *Fs*CYP51 binding region (Figure 1B). Notably, **3a–3t** interact with the heme cofactor through their phenyl moieties, while their thymol fragments are positioned near the substrate tunnel entrance of *Fs*CYP51. Consequently, in these derivatives, the phenyl moiety mediates interaction with the heme group, while the thymol fragments stabilize the entrance of the lanosterol tunnel. For derivative **3t**, however, docking calculations predicted a different binding pattern, likely due to the unique structure of the moiety linked to its 1,2,3-triazole ring (see Scheme 1). This distinct structural feature may explain lower antifungal activity compared to other active derivatives, as shown in Table 1. Specifically, when the 1,2,3-triazole ring is linked to a phenyl moiety, it positions closer to the heme group. For instance, the average distance between the triazole nitrogen in derivatives **3a–3r** and the heme iron is 8.4 Å, whereas for **3t**, the distance is 10.8 Å. This closer proximity between 1,2,3-triazole ring and heme group appears critical for effective inhibition of F. solani CYP51. Table 2 presents the binding energy and residues interacting with *Fs*CYP51.

**Table 2:** Binding Energy (*E*_b_, kcal/mol) Predicted by Docking Calculations and the Residues Interactions of Compounds **3a-3t** with *Fs*CYP51 Enzyme

compounds	*E* [Table-fn t2fn2]	residues
**3a**	–9.9	Tyr51, Phe217, Ala291, Ile359, His360, Ser361, Leu489, Phe490
**3b**	–10.2	Tyr51, Leu74, Phe75, Tyr105, Phe217, Ile359, Ile362, Leu489
**3c**	–10.4	Tyr51, Phe217, Ala291, Ile359, His360, Ser361, Leu489, Phe490
**3d**	–10.2	Tyr51, Tyr105, Phe217, Ala291, Ile359, Ser361, Leu489
**3e**	–10.1	Tyr51, Leu74, Phe75, Tyr105, Phe217, Ile359, Leu489
**3f**	–10.2	Tyr51, Tyr105, Phe217, Ile359, Ser361, Leu489, Phe490
**3g**	–10.4	Tyr51, Phe217, Ala291, Ile359, His360, Ser361, Leu489, Phe490
**3h**	–10.2	Tyr51, Tyr105, Phe217, Ala291, Ile359, Ser361, Leu489
**3i**	–10.0	Tyr51, Phe217, Ala291, Ile359, His360, Ser361, Leu489, Phe490
**3j**	–10.2	Tyr51, Phe217, Ala291, Ile359, His360, Ser361, Leu489, Phe490
**3k**	–10.0	Tyr51, Tyr105, Phe113, Phe217, Ala291, Ile359, Ser361, Leu489, Phe490
**3L**	–10.2	Tyr51, Tyr105, Phe217, Ile359, Ser361, Leu489, Phe490
**3m**	–10.2	Tyr51, Phe217, Ala291, Ile359, His360, Ser361, Leu489, Phe490
**3n**	–10.4	Tyr51, Phe217, Ala291, Ile359, His360, Ser361, Leu489, Phe490
**3o**	–10.4	Tyr51, Tyr105, Phe217, Ile359, Ser361, Leu489, Phe490
**3p**	–10.5	Tyr51, Tyr105, Phe217, Ile359, Ser361, Leu489, Phe490
**3q**	–10.6	Tyr51, Phe217, Ala291, Ile359, His360, Ser361, Leu489, Phe490
**3r**	–10.9	Tyr51, Tyr105, Phe217, Ile359, Ser361, Leu489, Phe490
**3s**	–11.6	Tyr51, Tyr105, Phe217, Ala291, Ile359, Ser361, Leu489, Phe490
**3t**	–10.3	Tyr51, Leu74, Phe75, Pro214, Phe217, Ala291, Ile359, His360, Ser361, Leu363, Leu489
THY	–6.6	Tyr105, Tyr109
TEB	–8.2	Tyr105, Phe113, Val118, Tyr119, Ala291, Ile359, Leu489, Phe490
LAN	–9.0	Tyr51, Leu74, Tyr105, Phe217, Leu363, Leu489

a Compounds that interact directly with the heme group.

Table 2 reveals that all derivatives exhibit better docking energies (lower *E*_b._) within the *Fs*CYP51 active site compared to the fungicide tebuconazole. Therefore, the experimentally observed antifungal activity for most derivatives (Table 1) can be attributed to their lower binding energies as predicted by docking calculations. Additionally, the negative *E*_b_ values indicate that all compounds bind to *Fs*CYP51 with favorable interactions. Molecular docking further demonstrates that all compounds **3a–3t** have lower binding energies than the substrate LAN (−9.0 kcal/mol) within the binding pocket (Table 2). Therefore, the fungicidal activity for the most compounds observed in the biological tests (Table 1), suggesting that these compounds may competitively inhibit LAN by obstructing ergosterol synthesis. Notably, derivatives **3m**, **3n**, **3o**, **3p**, and **3r** exhibit a high affinity for *Fs*CYP51, with binding energies of −10.2, −10.4, −10.4, −10.5, and −10.9 kcal/mol, respectively. This aligns with the antifungal activity data shown in Table 1. In Table 2, it is noteworthy that the binding pocket for derivatives **3a**–**3t** is primarily hydrophobic, with only Ser361 being polar, consistent with the crystallographic structure of A. fumigatus CYP51 (PDB code: 4UYM) as reported by Hargrove and co-workers.^59^ This suggests that derivatives **3a**–**3t** can block LAN's entry and stabilization within the enzyme binding pocket, illustrating their mechanism of action. Although the derivatives **3g–3k**, **3q** and **3s** lack antifungal activity (Table 1), they exhibit favorable interactions with CYP51 active site (Table 2). It is important to note that docking predicts how a ligand binds to a receptor but does not provide information about the accessibility pathway to the binding region. Interestingly, derivatives **3g–3k** contain a methyl or a methoxy group in the phenyl moiety, which may hinder their access to active site due to steric effects. Overall, Table 2 shows that the derivatives interact with a largely consistent set of residues within CYP51. For instance, the residues Tyr51, Phe217, His360, and Ser361 define the entrance of the substrate tunnel, while the residues Tyr105, Ala291, Ile359, Leu489, and Phe490 comprise the CYP51 active site. These residues play a significant role in mediating interactions with the thymol derivatives. Figure 2 provides further details, illustrating the interactions between highly active antifungal compounds and the *Fs*CYP51 enzyme.

**Figure 2:**
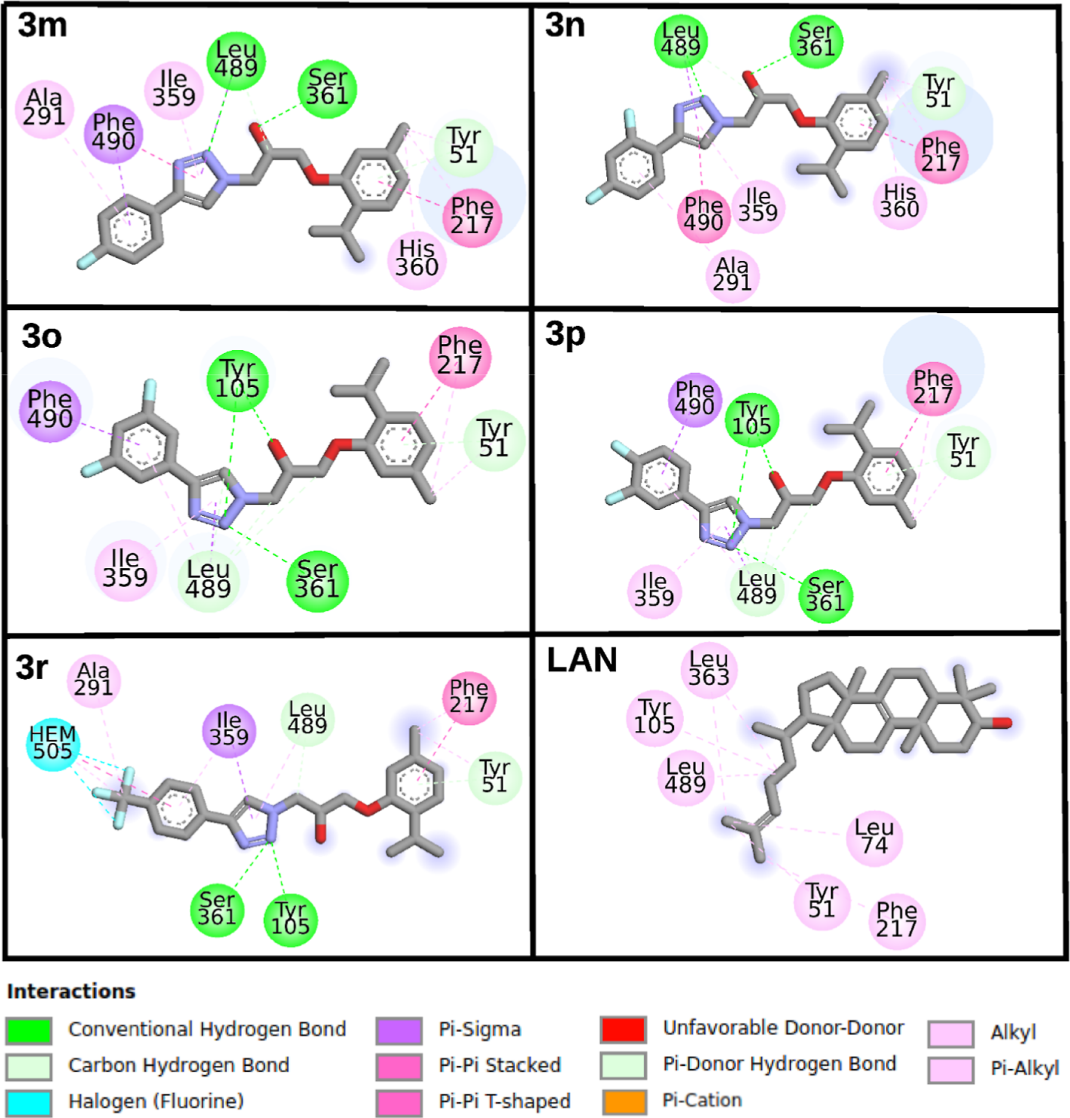
2D ligand interactions diagram for all best-docked compounds on the *Fs*CYP51.

Figure 2 shows that Tyr105 and Ser361 form hydrogen bonds with compounds **3p**, **3o**, and **3r** via their 1,2,3-triazole ring. In compounds **3m** and **3n**, hydrogen bonding occurs between Ser361 and Leu489, facilitated by their hydroxyl and 1,2,3-triazole groups, respectively. For the other derivatives, Leu489 interacts with the triazole ring through carbon–hydrogen bonding. Another key residue, Phe490, engages in π-σ interactions with the phenyl moiety of derivatives **3m**, **3o**, and **3p**. Notably, only compound **3r** interacts directly with the heme cofactor, while the remaining derivatives are positioned approximately 5 Å from the iron atom of the heme group. Regarding the substrate LAN, only weak alkyl-type interactions were observed with *Fs*CYP51. However, most residues interacting with LAN also engage with the derivatives (see Figure 2). Overall, our molecular docking results suggest that the majority of the derivatives can inhibit the *Fs*CYP51 enzyme by blocking access to the substrate entrance tunnel and preventing interactions with the heme-Fe active site.

In summary, using the natural product thymol as a starting material and a three-step synthetic sequence, we obtained a series of 20 derivatives featuring 1,2,3-triazole groups. Evaluation of their antifungal activity revealed that the five most active compounds exhibited significant effects, with a minimum inhibitory concentration (MIC) of 1.17 μg/mL. These potent compounds share a common structural feature: a fluorine atom or fluorinated moiety attached to the phenyl ring linked to the 1,2,3-triazole ring. Additionally, molecular docking analysis was performed to explore the binding mode of these 1,2,3-triazole derivatives with the *Fs*CYP51 enzyme. All derivatives demonstrated favorable binding to the *Fs*CYP51 catalytic site, with binding energies lower than −10 kcal/mol, surpassing both the substrate lanosterol (−9.0 kcal/mol) and the commercial fungicide tebuconazole (−8.2 kcal/mol). Our findings suggest that these derivatives could be more effective than tebuconazole in inhibiting *Fs*CYP51, as corroborated by experimental results. They achieve this inhibition by blocking lanosterol’s access to the substrate tunnel, thereby preventing its conversion into ergosterol. Overall, these findings indicate that the synthesized compounds may offer promising alternatives for the development of new antifungal agents for the control of F. solani in papaya crops.

## Supplementary Material



## Notes

1. Structural characterization data of compounds **1**, **2**, and triazoles **3a**–**3t**. 2. Spectroscopic data of compounds **1**, **2**, and **3a**–**3t** (PDF)
