# Supplementary material for: Design and Synthesis of Thymol Derivatives Bearing a 1,2,3-Triazole Moiety for Papaya Protection against Fusarium solani
Source: J Agric Food Chem. 2025 Jun 3;73(23):14290–9. doi: 10.1021/acs.jafc.4c12770 (PMC12164351; doi:10.1021/acs.jafc.4c12770)
Supplement: Supplementary file 1 [file jf4c12770_si_001.pdf]

Supporting Information

Design and Synthesis of Thymol Derivatives Bearing a 1,2,3-Triazole Moiety for Papaya Protection against *Fusarium solani*

Mariana Belizário de Oliveira[a], Poliana Aparecida Rodrigues Gazolla[a], Leandra Martins Meireles[b], Róbson Ricardo Teixeira[c*], Danilo Aniceto da Silva[c], Luiz Claudio Almeida Barbosa[d], Pedro Alves Bezerra Morais[a], Osmair Vital de Oliveira[e], Claudia Jorge do Nascimento[f], Pedro Henrique de Andrade Barrela[f], Jochen Junker[g], Nayara Araujo dos Santos[h], Wanderson Romão[h], Valdemar Lacerda[h], Waldir Cintra de Jesus Júnior[i], Eduardo Seiti Gomide Mizubuti[j], Vagner Tebaldi de Queiroz[a], Demetrius Profeti[k], Willian Bucker Moraes[l], Rodrigo Scherer[b] and Adilson Vidal Costa[a*].

[a]Departamento de Química e Física, Grupo de Pesquisa de Estudos Aplicados em Produtos Naturais e Síntese Orgânica (GEAPS), Universidade Federal do Espírito Santo, Alto Universitário, s/n, 29500-000 Alegre, Espírito Santo State, Brazil.

[b]Universidade de Vila Velha, Departamento de Farmácia, Programa de Pós-Graduação em Ciências Farmacêuticas, Av. Comissário José Dantas de Melo, 21, 29102-770, Vila Velha, Espírito Santo State, Brazil.

[c]Departamento de Química, Grupo de Síntese e Pesquisa de Compostos Bioativos (GSPCB), Universidade Federal de Viçosa, Av. P.H. RolFs, s/n, 36570-900 Viçosa, Minas Gerais State, Brazil

[d]Departamento de Química, Universidade Federal de Minas Gerais, Av. Antonio Carlos, 6627, 31270-901, Belo Horizonte, Minas Gerais State, Brazil.

[e]Instituto Federal de São Paulo, Campus Catanduva, 15808-305 Catanduva, SP, Brazil

[f]Departamento de Ciências Naturais, Instituto de Biociências, Universidade Federal do Estado do Rio de Janeiro (UNIRIO), Av. Pauster, 22290-240, Rio de Janeiro, Rio de Janeiro State, Brazil.

[g]Centro de Desenvolvimento Tecnológico em Saúde, Fundação Oswaldo Cruz, Av. Brasil, 4365, 21040-900 Rio de Janeiro, RJ, Brazil

[h]Laboratório de Petroleômica e Forense, Departamento de Química, Universidade Federal do Espírito Santo, Av. Fernando Ferrari 514, 29075-910 Vitória, ES, Brazil.

[i]Universidade Federal de São Carlos, Campus Lagoa do Sino, 18290-000 Buri, São Paulo State, Brazil.

[j]Departamento de Fitopatologia, Universidade Federal de Viçosa, Av. P.H. RolFs, s/n, 36570-900 Viçosa, Minas Gerais State, Brazil.

[k]Programa de Pós-Gradução em Agroquímica, Universidade Federal do Espírito Santo, Alto Universitário, s/n, 29500-000 Alegre, Espírito Santo State, Brazil.

[l]Programa de Pós-Gradução em Agronomia, Universidade Federal do Espírito Santo, Alto Universitário, s/n, 29500-000 Alegre, Espírito Santo State, Brazil.

*Corresponding authors: (e-mails: avcosta@hotmail.com, robsonr.teixeira@ufv.br)

1. Structural characterization data of compounds 1, 2, and triazoles 3a–3t

*Synthesis of (±)-2-((2-isopropyl-5-methylphenoxy)methyl) oxirane (**1**)*

Compound (**1**) was purified by silica gel column chromatography using hexane/ethyl acetate (8:1 v v^{-1}) as eluent and obtained as a colorless oil in 65% yield (1.34 g; 3.99 mmol). TLC: R_f = 0.42 (hexane/ethyl acetate 8:1 v v^{-1}); IR (ATR) $\bar{v}_{max}$ (cm^{-1}): 3054, 2961, 2930, 2867, 1613, 1502, 1452, 1414, 1288, 1262, 1174, 1093, 1040, 943, 912, 860, 811, 736, 703, 637. ^{1}H NMR (400 MHz, DMSO-d_6) δ 1.14 (s, 3H), 1.15 (s, 3H), 2.24 (s, 3H), 2.71 (dd, 1H, J = 5.2, 2.8 Hz), 2.83 (dd, 1H, J = 5.2, 4.4 Hz), 3.20 (sept, 1H, J = 6,8 Hz), 3.30–3.34 (m, 1H), 3.83 (dd, 1H, J = 11.3, 6.2 Hz), 4.29 (dd, 1H, J = 11.3, 2.4 Hz), 6.71 (d, 1H, J = 7.8 Hz), 6.75 (s, 1H), 7.04 (d, 1H, J = 7.8 Hz); ^{13}C NMR (100 MHz, DMSO-d_6) δ 21.3, 23.0, 26.6, 44.1, 50.3, 69.3, 113.2, 121.8, 126.1, 133.6, 136.3, 155.7; LC-MS (ESI) *m/z*, calcd. for [$C_{13}H_{18}O_2 + H^+$]: 207.13, found: 207.02.

*Synthesis of (±)-1-azido-3-(2-isopropyl-5-methylphenoxy)propan-2-ol (**2**)*

Compound (**2**) was purified by silica gel column chromatography using a mixture of hexane/ethyl acetate (6:1 v v^{-1}) as eluent and obtained as a colorless oil in 94% yield (1.14 g; 4.55 mmol). TLC: R_f = 0.40 (hexane/ethyl acetate 6:1 v v^{-1}); IR (ATR) $\bar{v}_{max}$ (cm^{-1}): 3401, 2962, 2930, 2875, 2102, 1618, 1578, 1506, 1456, 1284, 1253, 1166, 1095, 1095, 1042, 948, 817; ^{1}H NMR (400 MHz, DMSO-d_6) δ 1.12 (d, 3H, J =1.1 Hz), 1.13 (d, 3H, J = 1.1 Hz), 2.24 (s, 3H), 3.19 (sept, 1H, J = 6.8 Hz), 3.37–3.45 (m, 2H), 3.87 (dd, 1H, J = 9.6, 6.4 Hz), 3.92 (dd, 1H, J = 9.6, 4.8 Hz), 3.99–4.06 (m, 1H), 5.52 (d, 1H, J = 5.2 Hz), 6.70 (d, 1H, J = 7.8 Hz), 6.73 (s, 1H), 7.03 (d, 1H, J = 7.8 Hz); ^{13}C NMR (100 MHz, DMSO-d_6) δ 21.3, 23.1, 26.4, 53.8, 68.9, 69.6, 112.8, 121.6, 126.0, 133.5, 136.3, 155.5; LC-MS (ESI) *m/z*, calcd. for [$C_{13}H_{19}N_3O_2 + H^+$]: 250.15, found: 250.20; calcd. for [$C_{13}H_{19}N_3O_2 + Na^+$]: 272.13, found: 272.20.

*Synthesis of 1-(2-isopropyl-5-methylphenoxy)-3-(4-phenyl-1H-1,2,3-triazol-1-yl)propan-2-ol (**3a**)*

Compound **3a** was obtained as a yellow solid in 70% yield (0.991 g, 2.59 mmol) from phenylacetylene (0.410 g, 4.01 mmol), azide (**2**) (1.000 g, 4.01 mmol), sodium ascorbate (0.413 g,

2.09 mmol) and $CuSO_4{\cdot}5H_2O$ (0.260 g, 1.04 mmol), m.p. 112.3–115.5 °C. TLC: R_f = 0.32 (hexane/ethyl acetate 4:1 v v^{-1}); IR (ATR) $\bar{\nu}_{max}$ (cm^{-1}): 3208, 3162, 2959, 2928, 2884, 1612, 1574, 1512, 1444, 1412, 1284, 1260, 1223, 1153, 1098, 1048, 970, 942, 817, 761, 689; ^1H NMR (400 MHz, DMSO-d_6) δ 1.15 (s, 3H), 1.17 (s, 3H), 2.25 (s, 3H), 3.28 (sept, 1H, J = 6.8 Hz), 3.93 (dd, 1H, J = 9.8, 5.8 Hz), 3.99 (dd, 1H, J = 9.6, 4.8 Hz), 4.30–4.34 (m, 1H), 4.51 (dd, 1H, J = 14.0, 7.6 Hz), 4.67 (dd, 1H, J = 14.0, 3,6 Hz), 5.62 (d, 1H, J = 5.2 Hz), 6.72–6.74 (m, 2H), 7.06 (d, 1H, J = 7.6 Hz), 7.33 (t, 1H, J = 8.0 Hz), 7.44 (d, 2H, J = 8.0 Hz), 7.87 (d, 2H, J = 8.0 Hz), 8.57 (s, 1H), ^{13}C NMR (100 MHz, DMSO-d_6) δ 21.3, 23.2, 26.4, 53.4, 68.4, 69.9, 112.8, 121.6, 122.8, 125.5, 126.0, 125.5, 129.3, 131.3, 133.6, 136.3, 146.5, 155.7; LC-MS (ESI) m/z, calcd. for $[C_{21}H_{25}N_3O_2 + H^+]$: 352.20, found: 352.28; calcd. for $[C_{21}H_{25}N_3O_2 + Na^+]$: 374.18, found: 374.28.

*Synthesis of 1-(4-(2-bromophenyl)-1H-1,2,3-triazol-1-yl)-3-(2-isopropyl-5-methylphenoxy) propan-2-ol (**3b**)*

Compound **3b** was obtained as a brown solid in 68% yield (1.170 g, 2.71 mmol) from 1-bromo-2-ethynylbenzene (0.726 g, 4.01 mmol), azide (**2**) (1.00 g, 4.01 mmol), sodium ascorbate (0.413 g, 2.09 mmol) and $CuSO_4{\cdot}5H_2O$ (0.260 g, 1.04 mmol), m.p. 119.5–120.8 °C. TLC: R_f = 0.30 (hexane/ethyl acetate 3:1 v v^{-1}); IR (ATR) $\bar{\nu}_{max}$ (cm^{-1}): 3265, 3171, 2958, 2865, 1615, 1581, 1506, 1461, 1446, 1362, 1290, 1260, 1167, 1093, 1083, 1068, 1050, 1025, 979, 948, 808, 761, 728; ^1H NMR (400 MHz, DMSO-d_6) δ 1.15 (s, 3H), 1.17 (s, 3H), 2.25 (s, 3H), 3.28 (sept, 1H, J = 6.9 Hz), 3.95 (dd, 1H, J = 9.8, 5.8 Hz), 3.97 (dd, 1H, J = 9.8, 5.0 Hz), 4.29–4.36 (m, 1H), 4.58 (dd, 1H, J = 13.8, 7.4 Hz), 4.71 (dd, 1H, J = 14.0, 4.0 Hz), 5.62 (d, 1H, J = 5.2 Hz), 6.72–6.74 (m, 2H), 7.07 (d, 1H, J = 7,6 Hz), 7.31 (td, 1H, J = 7.6, 1.7 Hz), 7.50 (td, 1H, J = 7.6, 1.2 Hz), 7.74 (dd, 1H, J = 8.0, 1.2 Hz), 7.97 (dd, 1H, J = 7.8, 1.8 Hz), 8.62 (s, 1H); ^{13}C NMR (100 MHz, DMSO-d_6) δ 21.3, 23.2, 26.2, 53.3, 68.4, 69.8, 112.8, 120.9, 121.7, 125.3, 126.0, 128.4, 130.1, 130.7, 131.7, 133.6, 134.0, 136.3, 144.3, 155.6; LC-MS (ESI) m/z, calcd. for $[C_{21}H_{24}N_3O_2Br + Na^+]$: 452.09; found: 452.19.

*Synthesis of 1-(4-(3-bromophenyl)-1H-1,2,3-triazol-1-yl)-3-(2-isopropyl-5-methylphenoxy) propan-2-ol (**3c**).*

Compound **3c** was obtained as a white solid in 53% yield (0.915 g; 2.13 mmol) from 1-bromo-3-ethynylbenzene (0.726 g, 4.01 mmol), azide (**2**) (1.00 g, 4.01 mmol), sodium ascorbate (0.413 g; 2.09 mmol) and $CuSO_4{\cdot}5H_2O$ (0.260 g, 1.04 mmol), m.p. 105.3–107.2 °C. TLC: R_f = 0.30 (hexane/ethyl acetate 3:1 v v^{-1}); IR (ATR) $\bar{\nu}_{max}$ (cm^{-1}): 3236, 3155, 2962, 2918, 2862, 1612, 1568, 1506, 1463, 1412, 1344, 1260, 1232, 1170, 1113, 1055, 1056, 974, 870, 811, 793, 756, 682; ^{1}H NMR (400 MHz, DMSO-d_6) δ 1.15 (s, 3H), 1.16 (s, 3H), 2.25 (s, 3H), 3.26 (sept, 1H, J = 6.8 Hz), 3.96 (dd, 1H, J = 9.8, 5.8 Hz), 3.99 (dd, 1H, J = 9.8, 5.0 Hz), 4.27–4.34 (m, 1H), 4.51 (dd, 1H, J = 14.0, 7.6 Hz), 4.67 (dd, 1H, J = 13.8, 3.8 Hz), 5.62 (d, 1H, J = 5.2 Hz), 6.71–6.74 (m, 2H), 7.06 (d, 1H, J = 7.6 Hz), 7.42 (t, 1H, J = 7.8 Hz), 7.52 (d, 1H, J = 8.8 Hz), 7.99 (d, 1H, J = 7.6 Hz), 8.07 (s, 1H), 8.69 (s, 1H); ^{13}C NMR (100 MHz, DMSO-d_6) δ 21.3, 23.1, 26.4, 53.6, 68.4, 69.9, 112.8, 121.7, 122.7, 123,6, 124.4, 126.0, 128.0, 130.8, 131,5, 133.6, 136.4, 145.1, 155,7; LC-MS (ESI) *m/z*, calcd. for [$C_{21}H_{24}N_3O_2Br + Na^+$]: 452.09, found: 452.19.

*Synthesis of 1-(4-(4-bromophenyl)-1H-1,2,3-triazol-1-yl)-3-(2-isopropyl-5-methylphenoxy) propan-2-ol (**3d**).*

Compound **3d** was obtained as a white solid in 67% yield (1.14 g, 2.64 mmol) from 1-bromo-4-ethynylbenzene (0.726 g, 4.01 mmol), azide (**2**) (1.00 g, 4.01 mmol), sodium ascorbate (0.413 g, 2.09 mmol), $CuSO_4{\cdot}5H_2O$ (0.260 g, 1.04 mmol), m.p. 118.2–119.4 °C. TLC: R_f = 0.30 (hexane/ethyl acetate 3:1 v v^{-1}); IR (ATR) $\bar{\nu}_{max}$ (cm^{-1}): 3498, 3115, 3087, 2955, 2869, 1612, 1507, 1555, 1431, 1347, 1286, 1257, 1154, 1101, 1070, 1049, 1012, 976, 822, 815, 747, 723; ^{1}H NMR (400 MHz, DMSO-d_6) δ 1.15 (s, 3H), 1.16 (s, 3H), 2.25 (s, 3H), 3.26 (sept, 1H, J = 6.8 Hz), 3.93 (dd, 1H, J = 10.0, 5.6 Hz), 3.98 (dd, 1H, J = 10.0, 4.8 Hz), 4.27–4.34 (m, 1H), 4.51 (dd, 1H, J = 14.0, 7.6 Hz), 4.67 (dd, 1H, J = 13.8, 3.8 Hz), 5.62 (d, 1H, J = 5.2 Hz), 6.71–6.73 (m, 2H), 7.06 (d, 1H, J = 7.6 Hz), 7.64 (d, 2H, J = 8.4 Hz), 7.83 (d, 2H, J = 8.4 Hz), 8.63 (s, 1H); ^{13}C NMR (100

MHz, DMSO-d_6) δ 21.3, 23;1, 26.4, 53.5, 68.4, 69.9, 112.8, 121.1, 121.7, 123.2, 126.0, 127.5, 130.6, 132.3, 133.7, 136.4, 145.5, 155.7; LC-MS (ESI) *m/z*, calcd. for [$C_{21}H_{24}N_3O_2Br$ + H^+]: 430.11, found: 430.22.

Synthesis of 1-(4-(2-chlorophenyl)-1*H*-1,2,3-triazol-1-yl)-3-(2-isopropyl-5-methylphenoxy) propan-2-ol *(**3e**).*

Compound **3e** was obtained as a white solid in 72% yield (1.11 g, 2.88 mmol) from 1-chloro-2-ethynylbenzene (0.560 g, 4.01 mmol), azide (**2**) (1.000 g, 4.01 mmol), sodium ascorbate (0.413 g, 2.09 mmol), $CuSO_4{\cdot}5H_2O$ (0.260 g, 1.04 mmol), m.p. 98.7–100.1 °C. TLC: R_f = 0.30 (hexane/ethyl acetate 3:1 v v^{-1}); IR (ATR) $\bar{\nu}_{max}$ (cm^{-1}): 3264, 3171, 2956, 2921, 2862, 1611, 1579, 1507, 1469, 1445, 1362, 1290, 1262, 1167, 1084, 1052, 982, 809, 763, 735; ^{1}H NMR (400 MHz, DMSO-d_6) δ 1.15 (s, 3H), 1.16 (s, 3H), 2.25 (s, 3H), 3.28 (sept, 1H, *J* = 6.8 Hz), 3.92 (dd, 1H, *J* = 9.8, 5.8 Hz), 3.98 (dd, 1H, *J* = 9.8, 5.0 Hz), 4.33–4.36 (m, 1H), 4.58 (dd, 1H, *J* = 13.8, 7.4 Hz), 4.71 (dd, 1H, *J* = 14.0, 4.0 Hz), 5.62 (d, 1H, *J* = 5.2 Hz), 6.71–6.74 (m, 2H), 7.06 (d, 1H, *J* = 7.6 Hz), 7.38 (td, 1H, *J* = 7.7, 1.7 Hz), 7.46 (td, 1H, *J* = 7.4, 1.4 Hz), 7.57 (dd, 1H, *J* = 8.0, 1.2 Hz), 8.10 (dd, 1H, *J* = 7.8, 1.8 Hz), 8.62 (s, 1H); ^{13}C NMR (100 MHz, DMSO-d_6) δ 21.3, 23.2, 26.3, 53.3, 68.4, 69.9, 112.8, 121.7, 125.6, 126.0, 128.0, 129.7, 129.8, 129.8, 130.7, 130.7, 133.6, 136.3, 142.8, 155.7; LC-MS (ESI) *m/z*, calcd. for [$C_{21}H_{24}N_3O_2Cl$ + Na^+]: 408.14, found: 408.23.

*Synthesis of 1-(2-isopropyl-5-methylphenoxy)-3-(4-(o-tolyl)-1H-1,2,3-triazol-1-yl) propan-2-ol (**3f**).*
Compound **3f** was obtained as a yellow solid in 68% yield (0.991 g, 2.71 mmol) from 2-ethynyl toluene (0.466 g, 4.01 mmol), azide (**2**) (1.00 g, 4.01 mmol), sodium ascorbate (0.413 g, 2.09 mmol), $CuSO_4{\cdot}5H_2O$ (0.260 g, 1.04 mmol), m.p. 85.2–86.5 °C. TLC: R_f = 0.45 (hexane/ethyl acetate 2:1 v v^{-1}); IR (ATR) $\bar{\nu}_{max}$ (cm^{-1}): 3246, 2959, 2928, 2871, 1611, 1579, 1508, 1465, 14,43, 1413, 1362, 1288, 1261, 1166, 1085, 1050, 980, 944, 842, 809, 764, 727, 674; ^{1}H NMR (400 MHz, DMSO-d_6) δ 1.16 (s, 3H), 1.17 (s, 3H), 2.26 (s, 3H), 2.44 (s, 3H), 3.29 (sept, 1H, *J* = 7.0 Hz), 3.94

(dd, 1H, J = 10.0, 5.6 Hz), 3.99 (dd, 1H, J = 9.8, 5.0 Hz), 4.31–4.37 (m, 1H), 4.54 (dd, 1H, J = 13.8, 7.4 Hz), 4.69 (dd, 1H, J = 13.6, 4.0 Hz), 5.61 (d, 1H, J = 5.6 Hz), 6.72–6.75 (m, 2H), 7.07 (d, 1H, J = 7.6 Hz), 7.24–7.31 (m, 3H), 7.74–7.77 (m, 1H), 8.35 (s, 1H); ^{13}C NMR (100 MHz, DMSO-d_6) δ 21.3, 21.6, 23.1, 23.2, 26.4, 53.3, 68.5, 70.0, 112.8, 121.7, 124.6, 126.0, 126.4, 128.1, 128.6, 130.5, 131.3, 133.6, 135.2, 136.3, 145.7, 155.7; LC-MS (ESI) *m/z*, calcd. for [$C_{22}H_{27}N_3O_2$ + Na^+]: 388.19; found: 388.23.

*Synthesis of 1-(2-isopropyl-5-methylphenoxy)-3-(4-(m-tolyl)-1H-1,2,3-triazol-1-yl) propan-2-ol (**3g**).*

Compound **3g** was obtained as a green solid in 64% yield (0.938 g, 2.72 mmol) from 3-ethynyl toluene (0.466 g, 4.01 mmol), azide (**2**) (1.00 g, 4.01 mmol), sodium ascorbate (0.413 g, 2.09 mmol), $CuSO_4 \cdot 5H_2O$ (0.260 g, 1.04 mmol), m.p. 124.0–125.3 °C. TLC: R_f = 0.45 (hexane/ethyl acetate 2:1 v v^{-1}); IR (ATR) $\bar{\nu}_{max}$ (cm^{-1}): 3190, 2955, 2912, 2869, 1612, 1578, 1506, 1452, 1414, 1382, 1361, 1285, 1243, 1168, 1085, 1045, 967, 941, 847, 808, 783, 746, 692, 641; ^{1}H NMR (400 MHz, DMSO-d_6) δ 1.16 (s, 3H), 1.17 (s, 3H), 2.25 (s, 3H), 2.36 (s, 3H), 3.28 (sept, 1H, J = 7.0 Hz), 3.93 (dd, 1H, J = 10.0, 4.8 Hz), 3.98 (dd, 1H, J = 9.8, 5.0 Hz), 4.31–4.37 (m, 1H), 4.50 (dd, 1H, J = 14.0, 7.6 Hz), 4.66 (dd, 1H, J = 13.8, 3.8 Hz), 5.62 (d, 1H, J = 3.6 Hz), 6.71–6.74 (m, 2H), 7.06 (d, 1H, J = 7.6 Hz), 7.14 (d, 1H, J = 7.6 Hz), 7.33 (t, 1H, J = 7.6 Hz), 7.65 (d, 1H, J = 8.0 Hz), 7.70 (s, 1H), 8.53 (s, 1H); ^{13}C NMR (100 MHz, DMSO-d_6) δ 21.3, 21.5, 23.1, 26.4, 53.4, 68.4, 69.9, 112.8, 121.6, 122.7, 124.0, 126.1, 128.8, 129.2, 131.2, 133.6, 136.3, 138.4, 146.6, 155.7; LC-MS (ESI) *m/z*, calcd. for [$C_{22}H_{27}N_3O_2$ + H^+]: 366.21, found: 366.30; calcd. for [$C_{22}H_{27}N_3O_2$ + Na^+]: 388.19, found: 388.28.

*Synthesis of 1-(2-isopropyl-5-methylphenoxy)-3-(4-(p-tolyl)-1H-1,2,3-triazol-1-yl) propan-2-ol (**3h**).*

Compound **3h** was obtained as a yellow solid in 69% yield (1.020 g, 2.78 mmol) from 4-ethynyl toluene (0.466 g, 4.01 mmol), azide (**2**) (1.000 g, 4.01 mmol), sodium ascorbate (0.413 g, 2.09 mmol), $CuSO_4{\cdot}5H_2O$ (0.260 g, 1.04 mmol), m.p. 109.1–111.7 °C. TLC: R_f = 0.45 (hexane/ethyl acetate 2:1 v v^{-1}); IR (ATR) $\bar{\nu}_{max}$ (cm^{-1}): 3221, 2965, 2931, 2872, 1611, 1582, 1504, 1455, 1411, 1345, 1286, 1254, 1159, 1116, 1042, 835, 808, 802, 749, 707, 668; ^1H NMR (400 MHz, DMSO-d_6) δ 1.15 (s, 3H), 1.17 (s, 3H), 2.25 (s, 3H), 2.33 (s, 3H), 3.27 (sept, 1H, J = 7.0 Hz), 3.93 (dd, 1H, J = 10.0, 5.6 Hz), 3.98 (dd, 1H, J = 10.0, 4.8 Hz), 4.27–4.34 (m, 1H), 4.49 (dd, 1H, J = 13.6, 7.6 Hz), 4.65 (dd, 1H, J = 13.8, 3.8 Hz), 5.61 (d, 1H, J = 5.2 Hz), 6.71–6.73 (m, 2H), 7.06 (d, 1H, J = 7.2 Hz), 7.25 (d, 2H, J = 8.0 Hz), 7.75 (d, 2H, J = 8.0 Hz), 8.50 (s, 1H); ^{13}C NMR (100 MHz, DMSO-d_6) δ 21.2, 21.3, 23.1, 23.2, 26.4, 53.4, 68.4, 69.9, 112.8, 121.6, 122.4, 125.5, 126.0, 128.5, 129.8, 133.6, 136.4, 137.4, 146.5, 155.7; LC-MS (ESI) *m/z*, calcd. for $[C_{22}H_{27}N_3O_2 + H^+]$: 366.21, found: 366.29.

*Synthesis of 1-(2-isopropyl-5-methylphenoxy)-3-(4-(2-methoxyphenyl)-1H-1,2,3-triazol-1-yl) propan-2-ol (**3i**).*

Compound **3i** was obtained as a yellow solid in 67% yield (1.030 g, 2.69 mmol) from 2-ethynyl anisole (0.636 g, 4.01 mmol), azide (**2**) (1.000 g, 4.01 mmol), sodium ascorbate (0.413 g, 2.09 mmol), $CuSO_4{\cdot}5H_2O$ (0.260 g, 1.04 mmol), m.p. 102.1–104.3 °C.TLC: R_f = 0.40 (hexane/ethyl acetate 2:1 v v^{-1}); IR (ATR) $\bar{\nu}_{max}$ (cm^{-1}) 3206, 2966, 2938, 2863, 1608, 1555, 1490, 1470, 1443, 1361, 1285, 1250, 1163, 1075, 1048, 1021, 980, 945, 805, 755, 677; ^1H NMR (400 MHz, DMSO-d_6) δ 1.16 (s, 3H), 1.18 (s, 3H), 2.25 (s, 3H), 3.29 (sept, 1H, J = 6.6 Hz), 3.88 (s, 3H), 3.90 (dd, 1H, J = 9.6, 5.6 Hz), 3.96 (dd, 1H, J = 9.8, 5.0 Hz), 4.28–4.34 (m, 1H), 4.54 (dd, 1H, J = 14.0, 7.2 Hz), 4.68 (dd, 1H, J = 13.8, 4.2 Hz), 5.69 (d, 1H, J = 5.2 Hz), 6.72–6.74 (m, 2H), 7.04–7.08 (m, 2H), 7.13 (d, 1H, J = 8.0 Hz), 7.33 (td, 1H, J = 7.8, 1.6 Hz), 8.16 (dd, 1H, J = 7.8, 1.8 Hz), 8.36 (s, 1H);

^{13}C NMR (100 MHz, DMSO-d_6) δ 21.3, 23.1, 26.4, 53.1, 55.8, 68.4, 69.8, 111.9, 112.8, 119.6, 121.1, 121.6, 125.2, 126.0, 126.9, 129.2, 133.7, 136.4, 142.0, 155.6, 155.7; LC-MS (ESI) *m/z* calcd. for [$C_{22}H_{27}N_3O_3$+ H^+]: 382.21, found: 382.31; calcd. for [$C_{22}H_{27}N_3O_3$ + Na^+]: 404.19, found: 404.27.

*Synthesis of 1-(2-isopropyl-5-methylphenoxy)-3-(4-(3-methoxyphenyl)-1H-1,2,3-triazol-1-yl)propan-2-ol (**3j**).*

Compound **3j** was obtained as a white solid in 55% yield (0.844 g, 2.21 mmol) from 3-ethynyl anisole (0.636 g, 4.01 mmol), azide (**2**) (1.00 g, 4.01 mmol), sodium ascorbate (0.413 g, 2.09 mmol), $CuSO_4·5H_2O$ (0.260 g, 1.04 mmol), m.p. 84.2–86.4 °C. TLC: R_f = 0.40 (hexane/ethyl acetate 2:1 v v^{-1}); IR (ATR) $\bar{\nu}_{max}$ (cm^{-1}) 3187, 3151, 2958, 2945, 2870, 1617, 1581, 1491, 1453, 1366, 1277, 1236, 1176, 1153, 1094, 1044, 995, 941, 882, 845, 811, 777, 752, 693, 641; ^1H NMR (400 MHz, DMSO-d_6) δ 1.15 (s, 3H), 1.17 (s, 3H), 2.25 (s, 3H), 3.28 (sept, 1H, *J* = 6.8 Hz), 3.81 (s, 3H), 3.93 (dd, 1H, *J* = 9.6, 5.6 Hz), 3.96 (dd, 1H, *J* = 9.8, 5.0 Hz), 4.28–4.35 (m, 1H), 4.50 (dd, 1H, *J* = 14.0, 7.6 Hz), 4.66 (dd, 1H, *J* = 13.8, 3.8 Hz), 5.61 (d, 1H, *J* = 5,2 Hz), 6.72–6.74 (m, 2H), 6.89–6.91 (m, 1H), 7.07 (d, 1H, *J* = 7.6 Hz), 7.36 (t_{ap}, 1H, *J* = 7.8 Hz), 7.44–7.46 (m, 2H), 8.59 (s, 1H); ^{13}C NMR (100 MHz, DMSO-d_6) δ 21.3, 23.1, 26.4, 53.4, 55.5, 68.4, 69.9, 110.7, 112.8, 113.9, 117.9, 121.6, 123.1, 126.0, 130.4, 132.6, 133.7, 136.4, 146.4, 155.7, 160.1; LC-MS (ESI) *m/z* calcd. [$C_{22}H_{27}N_3O_3$ + Na^+]: 404.19, found: 404.27.

*Synthesis of 1-(2-isopropyl-5-methylphenoxy)-3-(4-(4-methoxyphenyl)-1H-1,2,3-triazol-1-yl) propan-2-ol (**3k**).*

Compound **3k** was obtained as a white solid in 68% yield (1.030 g, 2.71 mmol) from 4-ethynylanisole (0.636 g, 4.01 mmol), azide (**2**) (1.00 g, 4.01 mmol), sodium ascorbate (0.413 g, 2.09 mmol), $CuSO_4·5H_2O$ (0.260 g, 1.04 mmol), m.p. 123.9–125.3 °C. TLC: R_f = 0.40 (hexane/ethyl acetate 2:1 v v^{-1}); IR (ATR) $\bar{\nu}_{max}$ (cm^{-1}) 3152, 2965, 2869, 2830, 1615, 1561, 1503,

1455, 1413, 1360, 1250, 1224, 1176, 1115, 1091, 1030, 982, 942, 842, 806, 749, 609; ^{1}H NMR (400 MHz, DMSO-d_6) δ 1.15 (s, 3H), 1.17 (s, 3H), 2.25 (s, 3H), 3.27 (sept, 1H, J = 7.0 Hz), 3.79 (s, 3H), 3.93 (dd, 1H, J = 9.8, 5.8 Hz), 3.98 (dd, 1H, J = 10.0, 4.8 Hz), 4.27–4.34 (m, 1H), 4.49 (dd, 1H, J = 14.0, 7.6 Hz), 4.64 (dd, 1H, J = 14.0, 4.0 Hz), 5.60 (d, 1H, J = 5.2 Hz), 6.71–6.74 (m, 2H), 7.01 (d, 2H, J = 8.8 Hz), 7.06 (d, 1H, J = 7.6 Hz), 7.79 (d, 2H, J = 8.8 Hz), 8.45 (s, 1H); ^{13}C NMR (100 MHz, DMSO-d_6) δ 21.3, 23.1, 26.4, 53.4, 55.5, 68.4, 69.9, 112.8, 114.7, 121.6, 121.8, 123.9, 126.0, 126.9, 133.6, 136.3, 146.5, 155.7, 159.4; LC-MS (ESI) *m/z* calcd. for [$C_{22}H_{27}N_3O_3$ + H^+]: 382.30, found: 382.31; calcd. for [$C_{22}H_{27}N_3O_3$ + Na^+]: 404.19, found: 404.22.

*Synthesis of 1-(4-(2-fluorophenyl)-1H-1,2,3-triazol-1-yl)-3-(2-isopropyl-5-methylphenoxy) propan-2-ol (**3l**).*

Compound **3l** was obtained as a brown solid in 70% yield (1.04 g, 2.82 mmol) from 1-ethynyl-2-fluoro benzene (0.578 g, 4.01 mmol), azide (**2**) (1.00 g, 4.01 mmol), sodium ascorbate (0.413 g, 2.09 mmol), $CuSO_4 \cdot 5H_2O$ (0.260 g, 1.04 mmol), m.p. 105.0–106.1 °C. TLC: R_f = 0.47 (hexane/ethyl acetate 3:1 v v^{-1}); IR (ATR) $\bar{\nu}_{max}$ (cm^{-1}) 3249, 3165, 2965, 2918, 2875, 1611, 1580, 1506, 1490, 1435, 1410, 1344, 1257, 1216, 1153, 1082, 1036, 985, 858, 807, 754, 665; ^{1}H NMR (400 MHz, DMSO-d_6) δ 0.50 (s, 3H), 0.51 (s, 3H), 1.60 (s, 3H), 2.62 (sept, 1H, J = 6.8 Hz), 3.27 (dd, 1H, J = 9.8, 5.8 Hz), 3.32 (dd, 1H, J = 9.8, 5.0 Hz), 3.64–3.71 (m, 1H), 3.92 (dd, 1H, J = 13.8, 7.4 Hz), 4.05 (dd, 1H, J = 13.6, 4.0 Hz), 4.95 (d, 1H, J = 5.2 Hz), 6.06–6.08 (m, 2H), 6.41 (d, 1H, J = 7.6 Hz), 6.65–6.78 (m, 3H), 7.50 (td, 1H, J = 7.7, 1.8 Hz), 7.76 (d, 1H, J = 4.0 Hz); ^{13}C NMR (100 MHz, DMSO-d_6) δ 21.3, 23.1, 26.3, 53.3, 68.3, 69.9, 112.8, 116.4 (d, J = 21.0 Hz), 118.9 (d, J = 13.0 Hz), 121.7, 125.1 (d, J = 11.0 Hz), 125.4 (d, J = 3.0 Hz), 126.0, 127.7 (d, J = 4.0 Hz), 130.0 (d, J = 9.0 Hz), 133.7, 136.2, 139.9 (d, J = 3.0 Hz), 155.7, 158.9 (d, J = 241.0 Hz); LC-MS (ESI) *m/z*, calcd. for [$C_{21}H_{24}N_3O_2F$ + H^+]: 370.19, found: 370.27; calcd. for [$C_{21}H_{24}N_3O_2F$ + Na^+]: 392.17, found: 392.25.

*Synthesis of 1-(4-(4-fluorophenyl)-1H-1,2,3-triazol-1-yl)-3-(2-isopropyl-5-methylphenoxy) propan-2-ol (**3m**).*

Compound **3m** was obtained as a yellow solid in 51% yield (0.760 g, 2.06 mmol) from 1-ethynyl-4-fluoro benzene (0.578 g, 4.01 mmol), azide (**2**) (1.00 g, 4.01 mmol), sodium ascorbate (0.413 g, 2.09 mmol), $CuSO_4 \cdot 5H_2O$ (0.260 g, 1.04 mmol), m.p. 91.3–93.0 °C. TLC: R_f = 0.47 (hexane/ethyl acetate 3:1 v v^{-1}); IR (ATR) $\bar{\nu}_{max}$ (cm^{-1}) 3289, 2962, 2930, 2877, 1615, 1559, 1500, 1463, 1410, 1385, 1288, 1255, 1225, 1172, 1155, 1154, 1095, 1049, 944, 843, 811, 746, 655; ^1H NMR (300 MHz, DMSO-d_6) δ 1.13 (s, 3H), 1.15 (s, 3H), 2.23 (s, 3H), 3.24 (sept, 1H, J = 6.9 Hz), 3.91 (dd, 1H, J = 9.7, 5.8 Hz), 3.97 (dd, 1H, J = 10.0, 5.2 Hz), 4.26–4.33 (m, 1H), 4.49 (dd, 1H, J = 13.8, 7.5 Hz), 4.65 (dd, 1H, J = 13.9, 3.7 Hz), 5.59 (d, 1H, J = 5.4 Hz), 6.69–6.72 (m, 2H), 7.04 (d, 1H, J = 7.5 Hz), 7.26 (t_{ap}, 2H, J = 8.8 Hz), 7.89 (dd, 2H, J = 8.8, 5.5 Hz), 8.55 (s, 1H); ^{13}C NMR (75 MHz, DMSO-d_6) δ 21.3, 23.1, 26.4, 53.4, 68.4, 69.9, 112.8, 116.2 (d, J = 21.7 Hz), 121.6, 122.7, 126.0, 127.5 (d, J = 8.2 Hz), 127.9 (d, J = 3.0 Hz), 133.6, 136.3, 145.6, 155.7, 162.1 (d, J = 248.2 Hz); LC-MS (ESI) m/z, calcd. for $[C_{21}H_{24}N_3O_2F + H^+]$: 370.19, found: 370.31; calcd. for $[C_{21}H_{24}N_3O_2F + Na^+]$: 392.17, found: 392.27.

*Synthesis of 1-(4-(2,4-difluorophenyl)-1H-1,2,3-triazol-1-yl)-3-(2-isopropyl-5-methylphenoxy) propan-2-ol (**3n**).*

Compound **3n** was obtained as a yellow solid in 69% yield (1.04 g, 2.68 mmol) from 1-ethynyl-2,4-difluoro benzene (0.665 g, 4.01 mmol), azide (**2**) (1.00 g, 4.01 mmol), sodium ascorbate (0.413 g, 2.09 mmol), $CuSO_4 \cdot 5H_2O$ (0.260 g, 1.04 mmol), m.p. 95.4–96.2 °C. TLC: R_f = 0.33 (hexane/ethyl acetate 3:1 v v^{-1}); IR (ATR) $\bar{\nu}_{max}$ (cm^{-1}) 3501, 3171, 2956, 2925, 2865, 1628, 1614, 1603, 1563, 1495, 1462, 1414, 1361, 1289, 1277, 1255, 1169, 1146, 1075, 1047, 987, 959, 873, 810, 733, 664; ^1H NMR (300 MHz, DMSO-d_6) δ 1.12 (s, 3H), 1.15 (s, 3H), 2.23 (s, 3H), 3.25 (sept, 1H, J = 6.9 Hz), 3.90 (dd, 1H, J = 9.7, 5.8 Hz), 3.96 (dd, 1H, J = 9.9, 4.8 Hz), 4.26–4.35 (m, 1H), 4.55 (dd, 1H, J = 13.8, 7.2 Hz), 4.68 (dd, 1H, J = 13.8, 3.9 Hz), 5.58 (d, 1H, J = 5.4 Hz), 6.68–6.71 (m, 2H), 7.04

(d, 1H, J = 7.2 Hz), 7.20 (td, 1H, J = 8.3, 2.5 Hz), 7.37 (td, 1H, J = 10.2, 2.4 Hz), 8.11–8.19 (m, 1H), 8.38 (d, 1H, J = 3.6 Hz); ^{13}C NMR (75 MHz, DMSO-d_6) δ 21.2, 23.1, 26.3, 53.3, 68.3, 69.9, 104.9 (t, J = 25.8 Hz), 112.5 (d, J = 3.7 Hz), 112.8, 115.7 (dd, J = 12.7, 3.7 Hz), 121.4, 124.8 (d, J = 9.7 Hz), 125.9, 128.9 (dd, J = 9.7, 5.2 Hz), 133.5, 136.2, 139.3 (d, J = 3.0 Hz), 155.6, 158.8 (dd, J = 238.1, 10.1 Hz), 162.1 (dd, J = 235.8, 10.1 Hz);); LC-MS (ESI) *m/z*, calcd. for [$C_{21}H_{23}N_3O_2F_2$ + H^+]: 388.18, found: 388.33; calcd. for [$C_{21}H_{23}N_3O_2F_2$ + Na^+]: 410.16, found: 410.26.

*Synthesis of 1-(4-(3,5-difluorophenyl)-1H-1,2,3-triazol-1-yl)-3-(2-isopropyl-5-methylphenoxy) propan-2-ol (**3o**).*

Compound **3o** was obtained as a yellow solid in 64% yield (1.00 g, 2.58 mmol) from 1-ethynyl-3,5-difluorobenzene (0.665 g, 4.01 mmol), azide (**2**) (1.00 g, 4.01 mmol), sodium ascorbate (0.413 g, 2.09 mmol), $CuSO_4{\cdot}5H_2O$ (0.260 g, 1.04 mmol), m.p. 83.0–85.8 °C. TLC: R_f = 0.33 (hexane/ethyl acetate 3:1 v v^{-1}); IR (ATR) $\bar{\nu}_{max}$ (cm^{-1}) 3317, 3137, 2959, 2934, 2869, 1635, 1600, 1508, 1471, 1421, 1369, 1290, 1256, 1169, 1115, 1095, 1049, 985, 926, 864, 848, 835, 806, 747, 665; ^{1}H NMR (300 MHz, DMSO-d_6) δ 1.13 (s, 3H), 1.15 (s, 3H), 2.23 (s, 3H), 3.24 (sept, 1H, J = 6.9 Hz), 3.91 (dd, 1H, J = 9.7, 5.8 Hz), 3.97 (dd, 1H, J = 9.9, 4.8 Hz), 4.23–4.32 (m, 1H), 4.50 (dd, 1H, J = 13.8, 7.5 Hz), 4.66 (dd, 1H, J = 13.9, 3.7 Hz), 5.62 (d, 1H, J = 5.4 Hz), 6.69–6.72 (m, 2H), 7.04 (d, 1H, J = 7.5 Hz), 7.17 (t_{ap}, 1H, J = 9.4 Hz), 7.58 (d_{ap}, 2H, J = 6.6 Hz), 8.69 (s, 1H); ^{13}C NMR (75 MHz, DMSO-d_6) δ 21.3, 23.0, 26.4, 53.6, 68.3, 69.8, 103.3 (t, J = 28.1 Hz), 108.4 (dd, J = 18.0, 8.2 Hz), 112.8, 121.4, 124.1, 126.0, 133.5, 134.8 (t, J = 10.5 Hz), 136.2, 144.3, 155.6, 163.3 (dd, J = 243.7, 13.5 Hz); LC-MS (ESI) *m/z*, calcd. for [$C_{21}H_{23}N_3O_2F_2$ + H^+]: 388.18, found: 388.34; calcd. for [$C_{21}H_{23}N_3O_2F_2$ + Na^+]: 410.16, found: 410.26.

*Synthesis of 1-(4-(3,4-difluorophenyl)-1H-1,2,3-triazol-1-yl)-3-(2-isopropyl-5-methylphenoxy) propan-2-ol (**3p**).*

Compound **3p** was obtained as a yellow solid in 79% yield (1.23 g, 3.17 mmol) from 1-ethynyl-3,4-difluoro benzene (0.665 g, 4.01 mmol), azide (**2**) (1.00 g, 4.01 mmol), sodium ascorbate (0.413 g, 2.09 mmol), $CuSO_4·5H_2O$ (0.260 g, 1.04 mmol), m.p. 69.3–71.5 °C. TLC: R_f = 0.33 (hexane/ethyl acetate 3:1 v v^{-1}); IR (ATR) $\bar{\nu}_{max}$ (cm^{-1}) 3277, 3133, 2956, 2921, 2865, 1610, 1506, 1442, 1363, 1323, 1275, 1256, 1215, 1169, 1115, 1095, 1076, 1051, 999, 924, 890, 875, 804, 776, 748, 634; ^1H NMR (300 MHz, DMSO-d_6) δ 1.13 (s, 3H), 1.15 (s, 3H), 2.23 (s, 3H), 3.24 (sept, 1H, J = 6.9 Hz), 3.91 (dd, 1H, J = 9.7, 5.4 Hz), 3.97 (dd, 1H, J = 9.9, 4.8 Hz), 4.28–4.32 (m, 1H), 4.49 (dd, 1H, J = 13.8, 7.5 Hz), 4.65 (dd, 1H, J = 13.8, 3.9 Hz), 5.61 (d, 1H, J = 5.1 Hz), 6.69–6.71 (m, 2H), 7.04 (d, 1H, J = 7.5 Hz), 7.45–7.54 (m, 1H), 7.70–7.74 (m, 1H), 7.85–7.92 (m, 1H), 8.62 (s, 1H); ^{13}C NMR (75 MHz, DMSO-d_6) δ 21.2, 23.1, 26.3, 53.5, 68.4, 69.8, 112.8, 114.4 (d, J = 18.7 Hz), 118.5 (d, J = 18.0 Hz), 121.4, 122.3 (dd, J = 6.3, 3.3 Hz), 123.4, 126.0, 129.0 (dd, J = 7.5, 3.7 Hz), 133.5, 136.2, 144.5, 149.3 (dd, J = 238.1, 11.6 Hz), 150.2 (dd, J = 244.1, 12.7 Hz), 155.6; LC-MS (ESI) *m/z*, calcd. for $[C_{21}H_{23}N_3O_2F_2 + H^+]$: 388.18, found: 388.32; calcd. for $[C_{21}H_{23}N_3O_2F_2 + Na^+]$: 410.16, found: 410.25.

*Synthesis of 1-(2-isopropyl-5-methylphenoxy)-3-(4-(2-(trifluoromethyl)phenyl)-1H-1,2,3-triazol-1-yl) propan-2-ol (**3q**).*

Compound **3q** was obtained as a white solid in 51% yield (0.832 g, 1.98 mmol) from 1-ethynyl-2-trifluoromethyl benzene (0.819 g, 4.01 mmol), azide (**2**) (1.00 g, 4.01 mmol), sodium ascorbate (0.413 g, 2.09 mmol), $CuSO_4·5H_2O$ (0.260 g, 1.04 mmol), m.p. 121.3–123.8 °C. TLC: R_f = 0.45 (hexane/ethyl acetate 2:1 v v^{-1}); IR (ATR) $\bar{\nu}_{max}$ (cm^{-1}) 3255, 2962, 2930, 2887, 1612, 1581, 1507, 1438, 1418, 1366, 1313, 1302, 1264, 1179, 1120, 1095, 1054, 1033, 981, 948, 807, 768, 695; ^1H NMR (300 MHz, DMSO-d_6) δ 1.14 (s, 3H), 1.16 (s, 3H), 2.23 (s, 3H), 3.26 (sept, 1H, J = 6.8 Hz), 3.89 (dd, 1H, J = 9.7, 5.8 Hz), 3.95 (dd, 1H, J = 10.0, 5.2 Hz), 4.27–4.34 (m, 1H), 4.56 (dd, 1H, J =

13.8, 7.2 Hz), 4.69 (dd, 1H, J = 13.8, 3.9 Hz), 5.59 (d, 1H, J = 5.4 Hz), 6.69–6.72 (m, 2H), 7.05 (d, 1H, J = 8.1 Hz), 7.61 (t_{ap}, 1H, J = 7.5 Hz), 7.75 (t_{ap}, 1H, J = 7.5 Hz), 7.83 (t_{ap}, 2H, J = 6.9 Hz), 8.27 (d, 1H, J = 3.6 Hz); ^{13}C NMR (75 MHz, DMSO-d_6) δ 21.2, 23.1, 26.4, 53.2, 68.4, 69.8, 112.8, 121.6, 124.4 (q, J = 271.7 Hz), 125.2 (q, J = 4.0 Hz), 126.0, 126.4–126.8 (m), 129.1, 130.0 (q, J = 1.7 Hz), 132.1, 133.0, 133.6, 136.3, 143.4, 155.6; LC-MS (ESI) *m/z*, calcd. for [$C_{22}H_{24}N_3O_2F_3$ + H^+]: 420.18, found: 420.33; calcd. for [$C_{22}H_{24}N_3O_2F_3$ + Na^+]: 442.17, found: 442.28.

*Synthesis of 1-(2-isopropyl-5-methylphenoxy)-3-(4-(4-(trifluoromethyl)phenyl)-1H-1,2,3-triazol-1-yl) propan-2-ol (**3r**).*

Compound **3r** was obtained as a brown solid in 61% yield (1.01 g, 2.42 mmol) from 1-ethynyl-4-(trifluoromethyl)benzene (0.819 g, 4.01 mmol), azide (**2**) (1.00 g, 4.01 mmol), sodium ascorbate (0.413 g, 2.09 mmol), $CuSO_4{\cdot}5H_2O$ (0.260 g, 1.04 mmol), m.p. 117.1–119.0 °C. TLC: R_f = 0.45 (hexane/ethyl acetate 2:1 v v^{-1}); IR (ATR) $\bar{\nu}_{max}$ (cm^{-1}) 3427, 2959, 2921, 2875, 1621, 1581, 1503, 1456, 1415, 1323, 1259, 1235, 1160, 1125, 1105, 1065, 1038, 976, 942, 837, 814, 752, 649; ^{1}H NMR (300 MHz, DMSO-d_6) δ 1.13 (s, 3H), 1.15 (s, 3H), 2.23 (s, 3H), 3.25 (sept, 1H, J = 6.8 Hz), 3.93 (dd, 1H, J = 9.7, 5.8 Hz), 3.99 (dd, 1H, J = 10.0, 4.9 Hz), 4.26–4.35 (m, 1H), 4.52 (dd, 1H, J = 13.9, 7.6 Hz), 4.68 (dd, 1H, J = 13.8, 3.6 Hz), 5.62 (d, 1H, J = 5.4 Hz), 6.69–6.73 (m, 2H), 7.04 (d, 1H, J = 7.5 Hz), 7.79 (d, 2H, J = 8.2 Hz), 8.09 (d, 2H, J = 8.2 Hz), 8.74 (s, 1H); ^{13}C NMR (75 MHz, DMSO-d_6) δ 21.3, 23.1, 26.4, 53.6, 68.4, 69.9, 112.8, 121.6, 124.1, 124.7 (q, J = 270.0 Hz), 126.0, 126.0, 126.2 (q, J = 3.7 Hz), 128.3 (q, J = 32.2 Hz), 133.7, 135.2, 136.3, 145.1, 155.6; LC-MS (ESI) *m/z*, calcd. for [$C_{22}H_{24}N_3O_2F_3$ + H^+]: 420.18, found: 420.34; calcd. for [$C_{22}H_{24}N_3O_2F_3$ + Na^+]: 442.17, found: 442.28.

*Synthesis of 1-(4-(3,5-bis(trifluoromethyl)phenyl)-1H-1,2,3-triazol-1-yl)-3-(2-isopropyl-5-methylphenoxy) propan-2-ol (**3s**).*

Compound **3s** was obtained as a brown solid in 57% yield (1.11 g, 2.28 mmol) from 1-ethynyl-3,5-bis(trifluoromethyl)benzene (1.15 g, 4.01 mmol), azide (**2**) (1.00 g, 4.01 mmol), sodium ascorbate (0.413 g, 2.09 mmol), $CuSO_4{\cdot}5H_2O$ (0.260 g, 1.04 mmol), m.p. 105.1–106.7 °C. TLC: R_f = 0.45 (hexane/ethyl acetate 3:1 v v^{-1}); IR (ATR) $\bar{\nu}_{max}$ (cm^{-1}) 3386, 2962, 2925, 2875, 1618, 1578, 1508, 1468, 1386, 1333, 1321, 1275, 1257, 1183, 1127, 1106, 1054, 896, 843, 814, 761, 702, 682; ^1H NMR (300 MHz, DMSO-d_6) δ 1.12 (s, 3H), 1.15 (s, 3H), 2.23 (s, 3H), 3.24 (sept, 1H, J = 6.8 Hz), 3.94 (dd, 1H, J = 10.2, 4.2 Hz), 4.00 (dd, 1H, J = 12.3, 5.4 Hz), 4.24–4.36 (m, 1H), 4.53 (dd, 1H, J = 13.8, 7.5 Hz), 4.69 (dd, 1H, J = 13.8, 3.6 Hz), 5.63 (d, 1H, J = 5.4 Hz), 6.68–6.72 (m, 2H), 7.03 (d, 1H, J = 7.5 Hz), 8.02 (s, 1H), 8.53 (s, 2H), 9,00 (s, 1H); ^{13}C NMR (75 MHz, DMSO-d_6) δ 21.3, 23.0, 26.4, 53.7, 68.3, 69.8, 112.8, 121.2–121.3 (m), 123.7 (q, J = 271.7 Hz), 125.7, 131.4 (q, J = 33.7 Hz), 121.6, 124.9, 125.9, 133.6, 133.9, 136.3, 143.9, 155.3; LC-MS (ESI) *m/z*, calcd. for [$C_{23}H_{23}N_3O_2F_6$ + H^+]: 488.17, found: 488.33; calcd. for [$C_{23}H_{23}N_3O_2F_6$ + Na^+]: 510.15, found: 510.27.

*Synthesis of 1-(4-((4-allyl-2-methoxyphenoxy)methyl)-1H-1,2,3-triazol-1-yl)-3-(2-isopropyl-5-methylphenoxy) propan-2-ol (**3t**).*

Compound **3t** was obtained as a yellow solid in 59% yield (0.532 g, 1.18 mmol) from 4-allyl-2-methoxy-1-(prop-2-yn-1-yloxy) benzene (0.497 g, 2.05 mmol), azide (**2**) (0.500 g, 2.05 mmol), sodium ascorbate (0.211 g, 1.06 mmol), $CuSO_4{\cdot}5H_2O$ (0.133 g, 0.532 mmol), m.p. 85.5–87.7 °C. TLC: R_f = 0.30 (hexane/ethyl acetate 3:2 v v^{-1}); IR (ATR) $\bar{\nu}_{max}$ (cm^{-1}) 3270, 2958, 2921, 1639, 1611, 1510, 1467, 1421, 1387, 1259, 1221, 1146, 1099, 1048, 1021, 992, 920, 852, 810, 749, 679; ^1H NMR (300 MHz, DMSO-d_6) δ 1.14 (s, 3H), 1.16 (s, 3H), 2.24 (s, 3H), 3.21–3.29 (m, 2H), 3.86 (dd, 1H, J = 9.7, 5.8 Hz), 3.93 (dd, 1H, J = 9.7, 4.9 Hz), 4.24 (m, 1H), 4.46 (dd, 1H, J = 13.8, 7.5 Hz), 4.61 (dd, 1H, J = 13.8, 3.6 Hz), 5.00–5.08 (m, 4H), 5.56 (d, 1H, J = 4.8 Hz), 5.93 (ddt_{ap}, 1H, J

= 16.8, 10.0, 6.7 Hz), 6.65–6.71 (m, 3H), 8.09 (s, 1H), 7.01–7.06 (m, 2H), 8.15 (s, 1H); ^{13}C NMR (75 MHz, DMSO-d_6) δ 21.3, 23.1, 26.4, 53.1, 55.8, 62.3, 68.3, 69.8, 112.8, 112.9, 114.3, 115.9, 120.5, 121.6, 125.9, 126.0, 133.3, 133.9, 136.3, 138.3, 143.1, 146.2, 149.4, 155.6; LC-MS (ESI) *m/z*, calcd. for [$C_{26}H_{33}N_3O_4$ + H^+]: 452.25, found: 452.39; calcd. for [$C_{26}H_{33}N_3O_4$ + Na^+]: 474.23, found: 474.39.

2. Spectroscopic data of compounds 1, 2, and 3a–3t

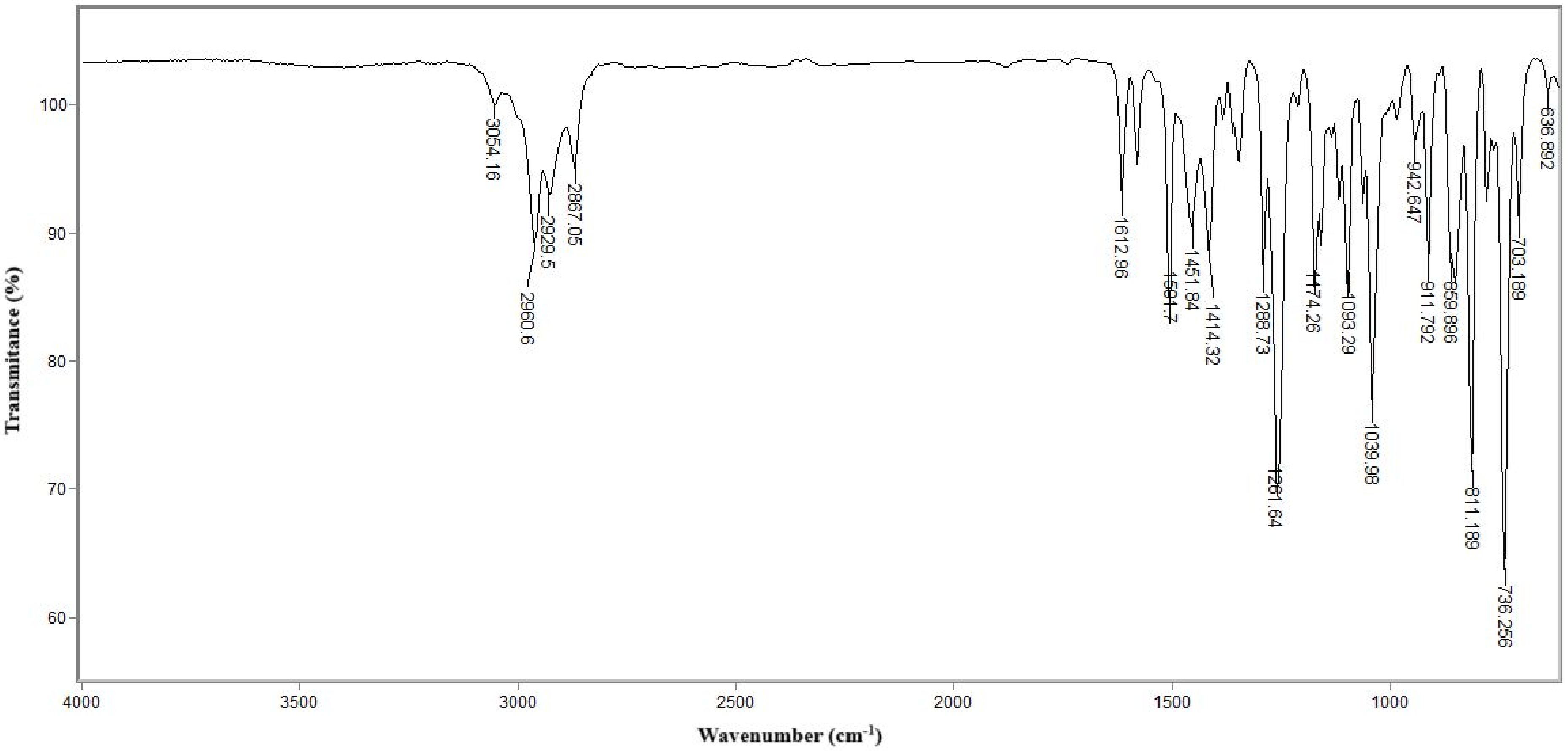

Figure S1. FTIR spectrum (ATR) of 2-((2-isopropyl-5-methylphenoxy)methyl) oxirane (**1**).

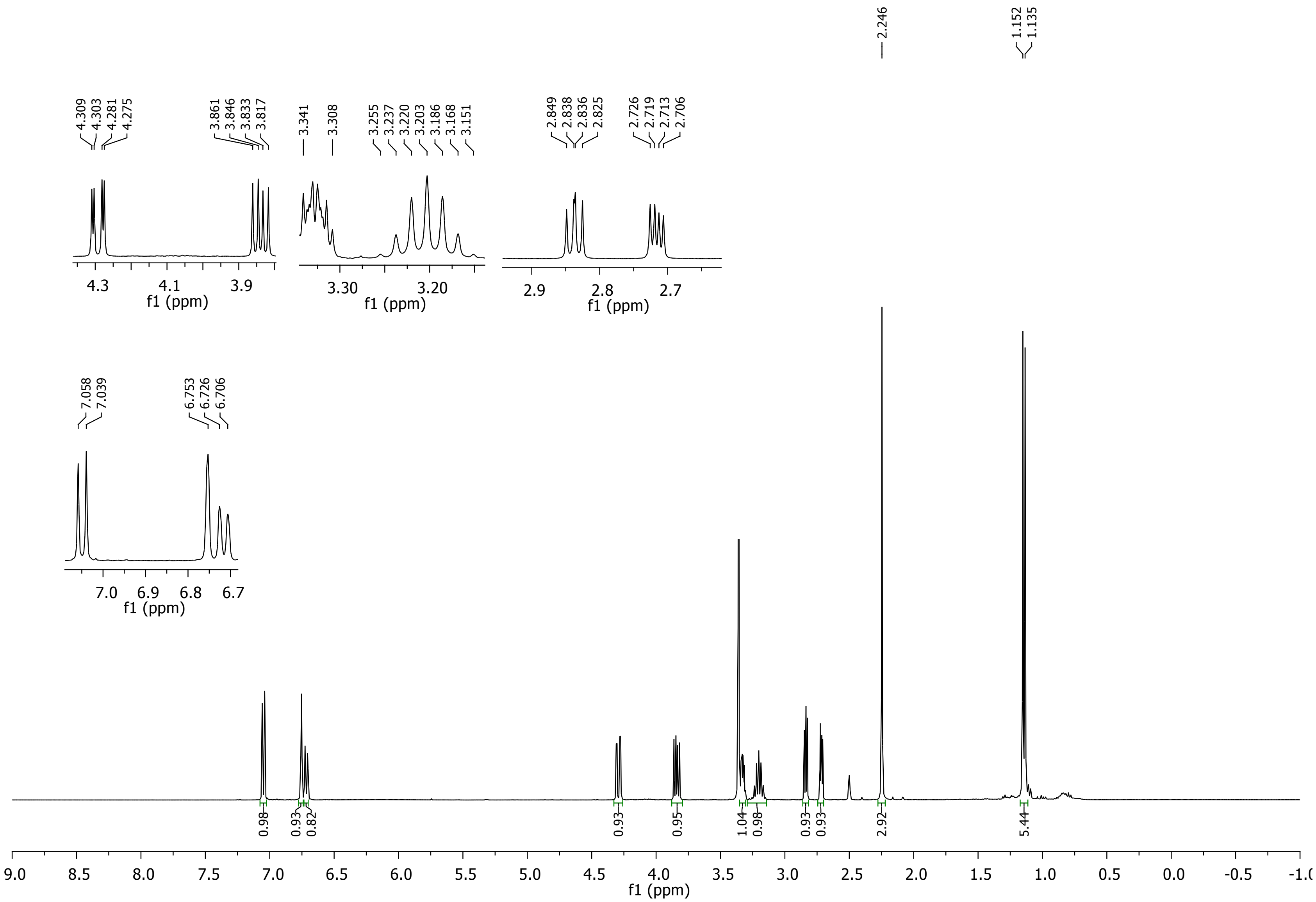

Figure S2. ^{1}H NMR spectrum (400 MHz, DMSO-d_6) of 2-((2-isopropyl-5-methylphenoxy)methyl) oxirane (**1**).

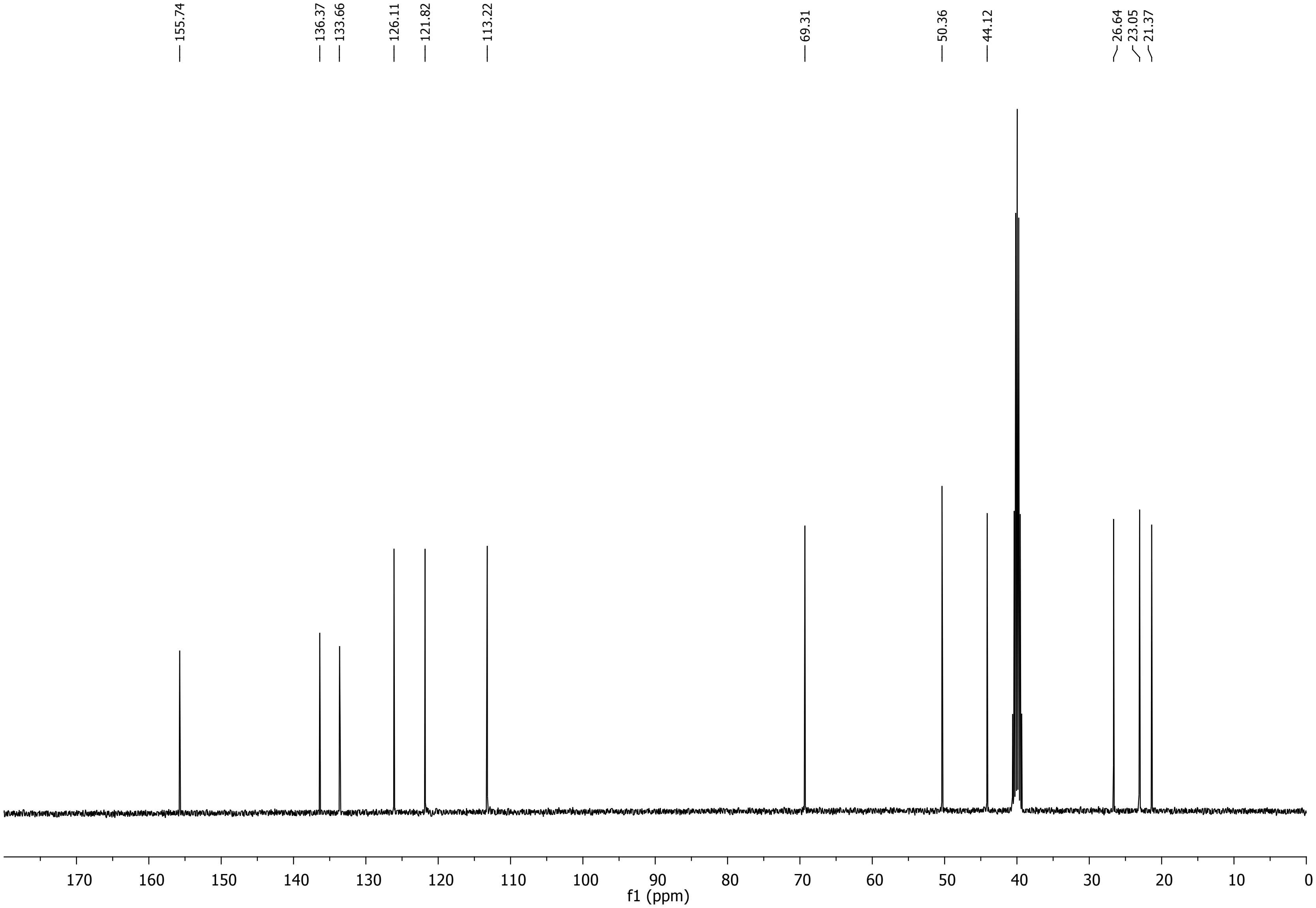

Figure S3. ^{13}C NMR spectrum (100 MHz, DMSO-d_6) of 2-((2-isopropyl-5-methylphenoxy)methyl) oxirane (**1**).

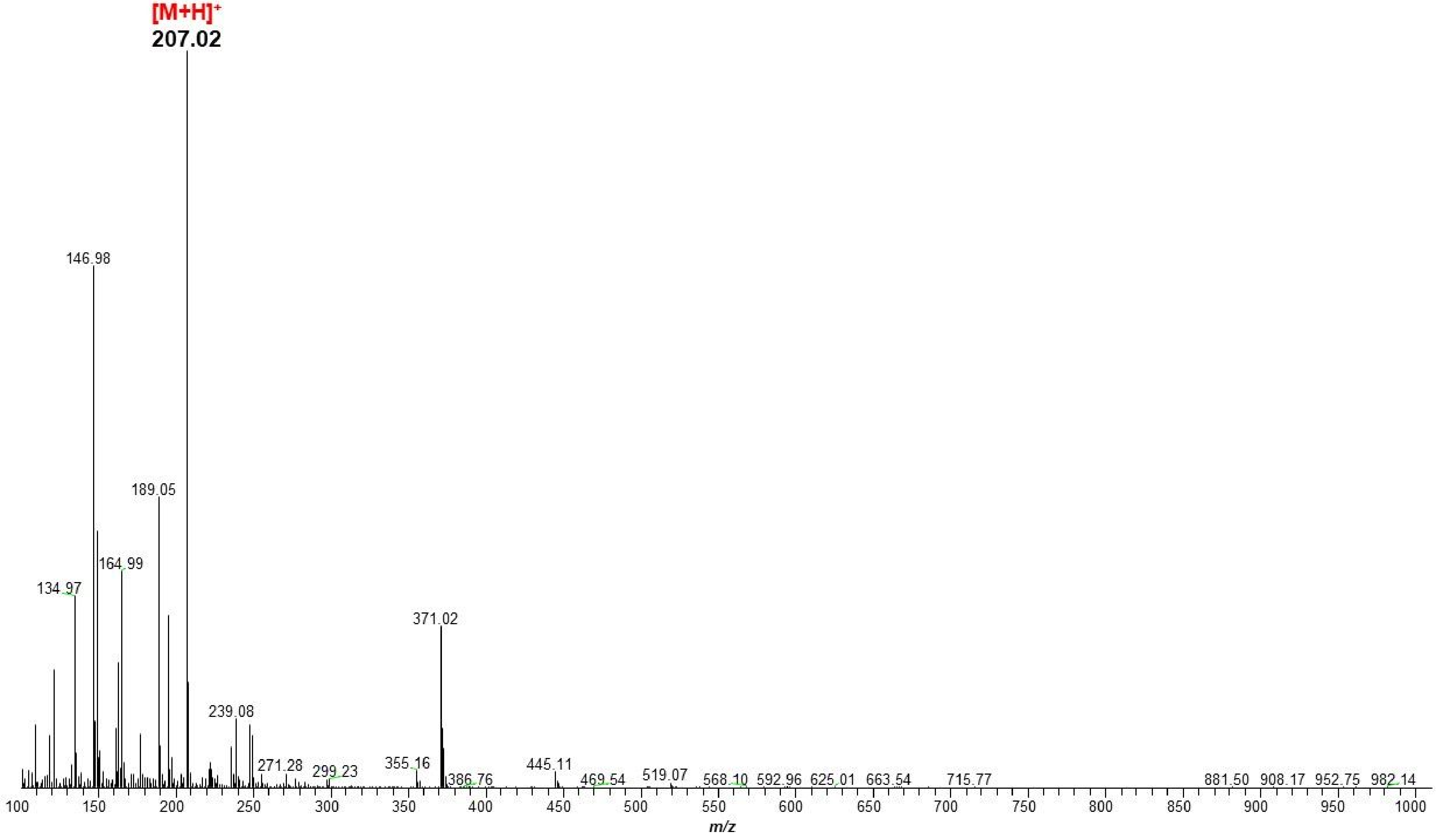

Figure S4. LC-MS spectrum of 2-((2-isopropyl-5-methylphenoxy)methyl) oxirane (**1**).

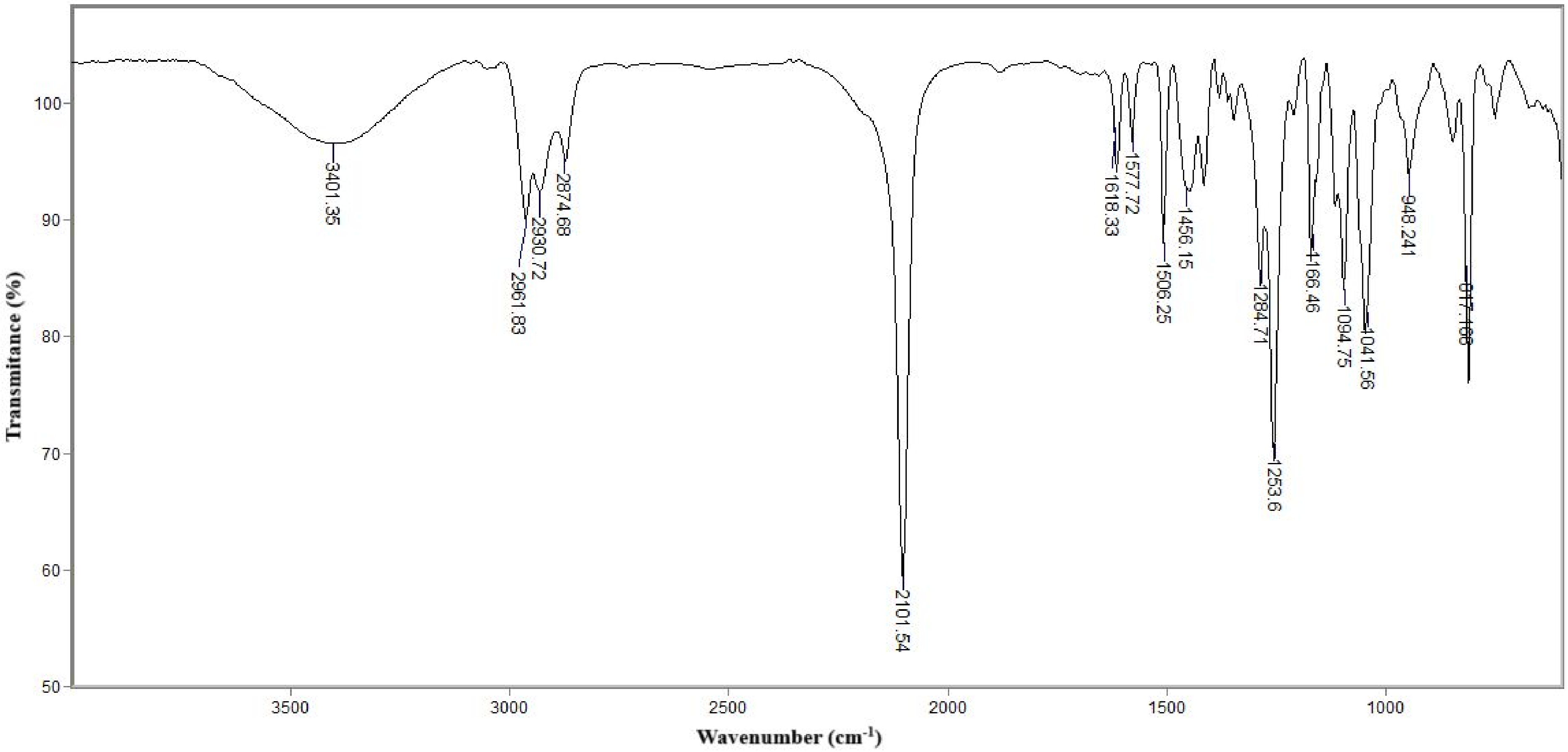

Figure S5. FTIR spectrum (ATR) of 1-azido-3-(2-isopropyl-5-methylphenoxy) propan-2-ol (**2**).

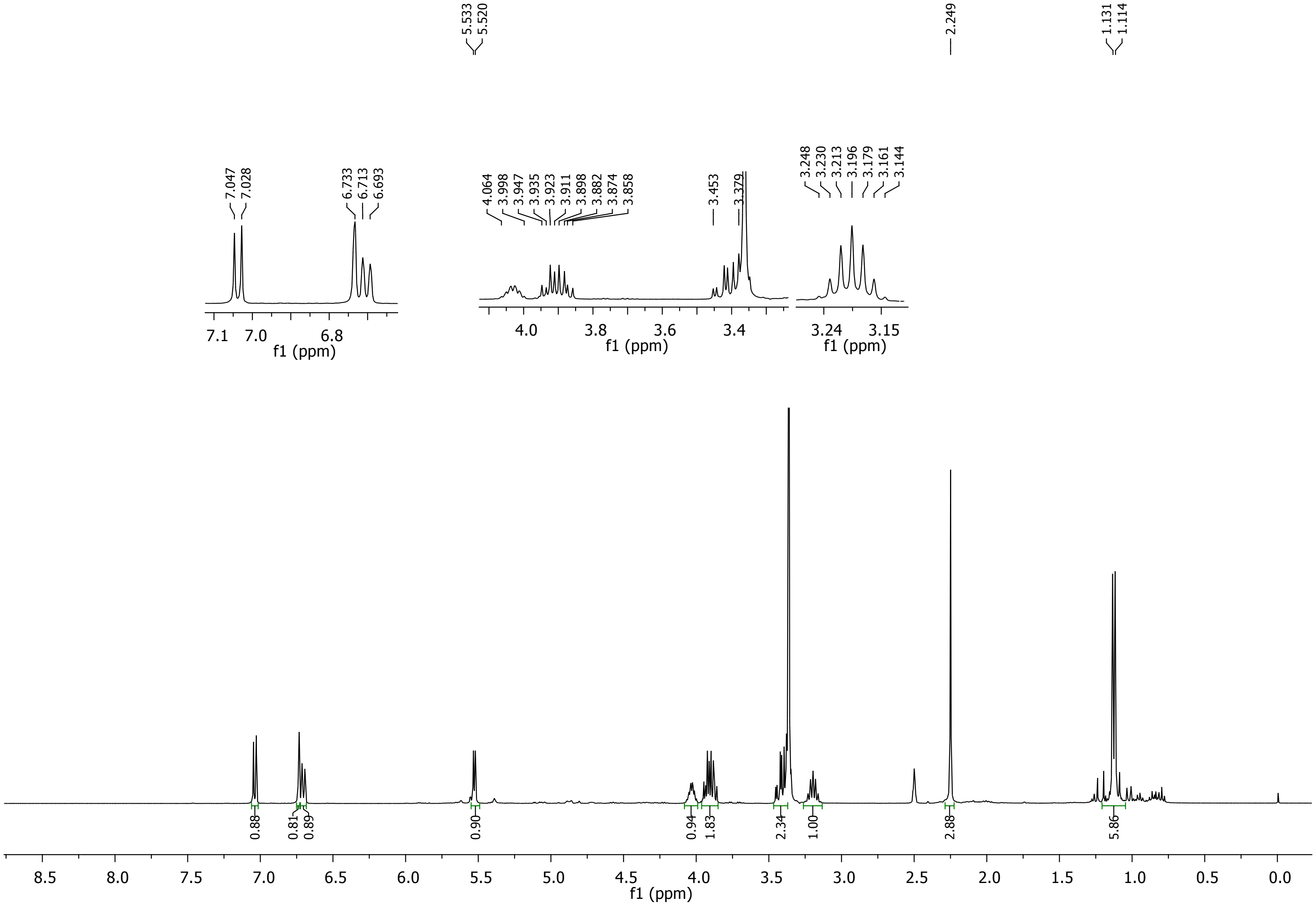

Figure S6. ^{1}H NMR spectrum (400 MHz, DMSO-d_6) of 1-azido-3-(2-isopropyl-5-methylphenoxy) propan-2-ol (**2**).

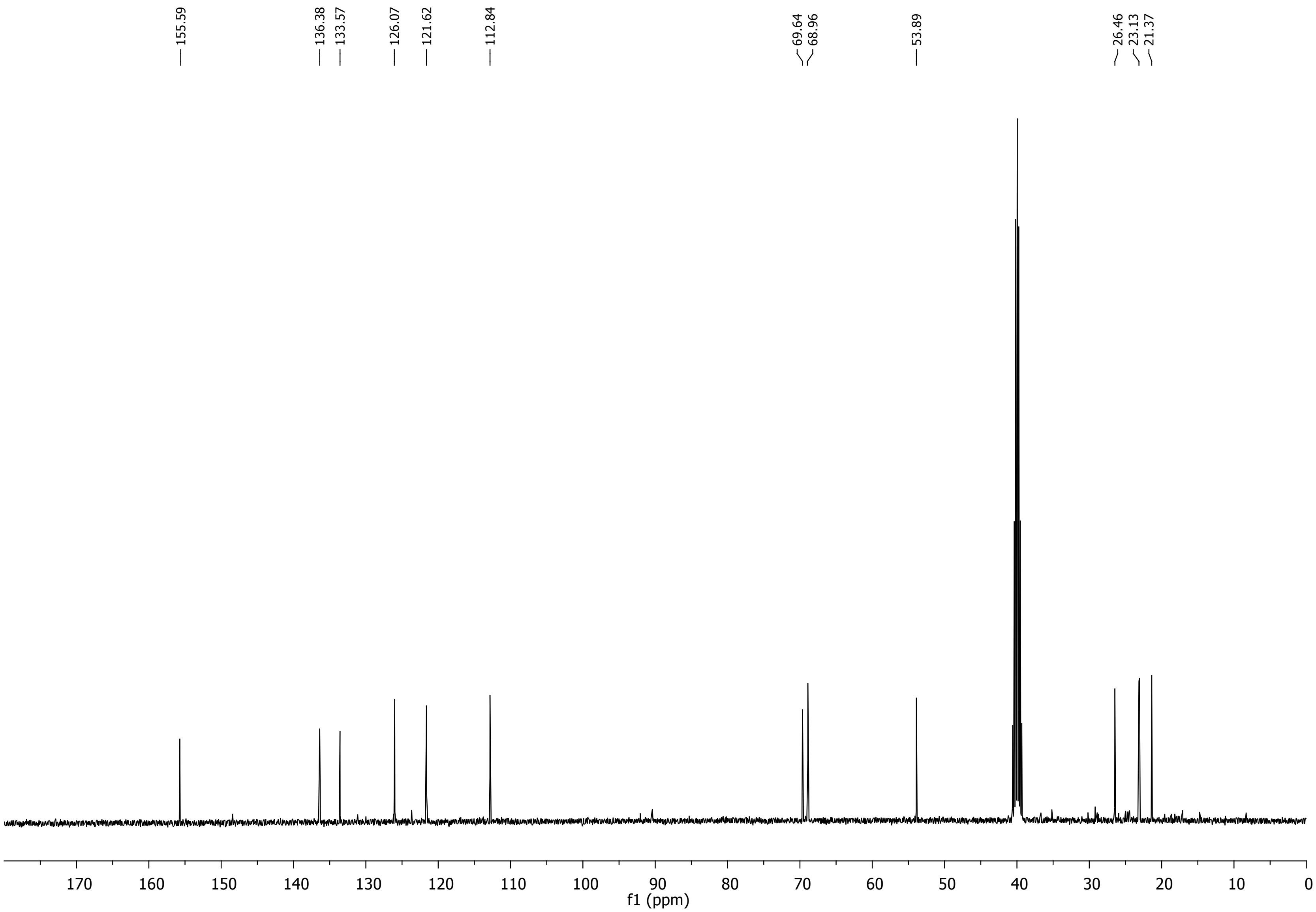

Figure S7. ^{13}C NMR spectrum (100 MHz, DMSO-d_6) of 1-azido-3-(2-isopropyl-5-methylphenoxy) propan-2-ol (**2**).

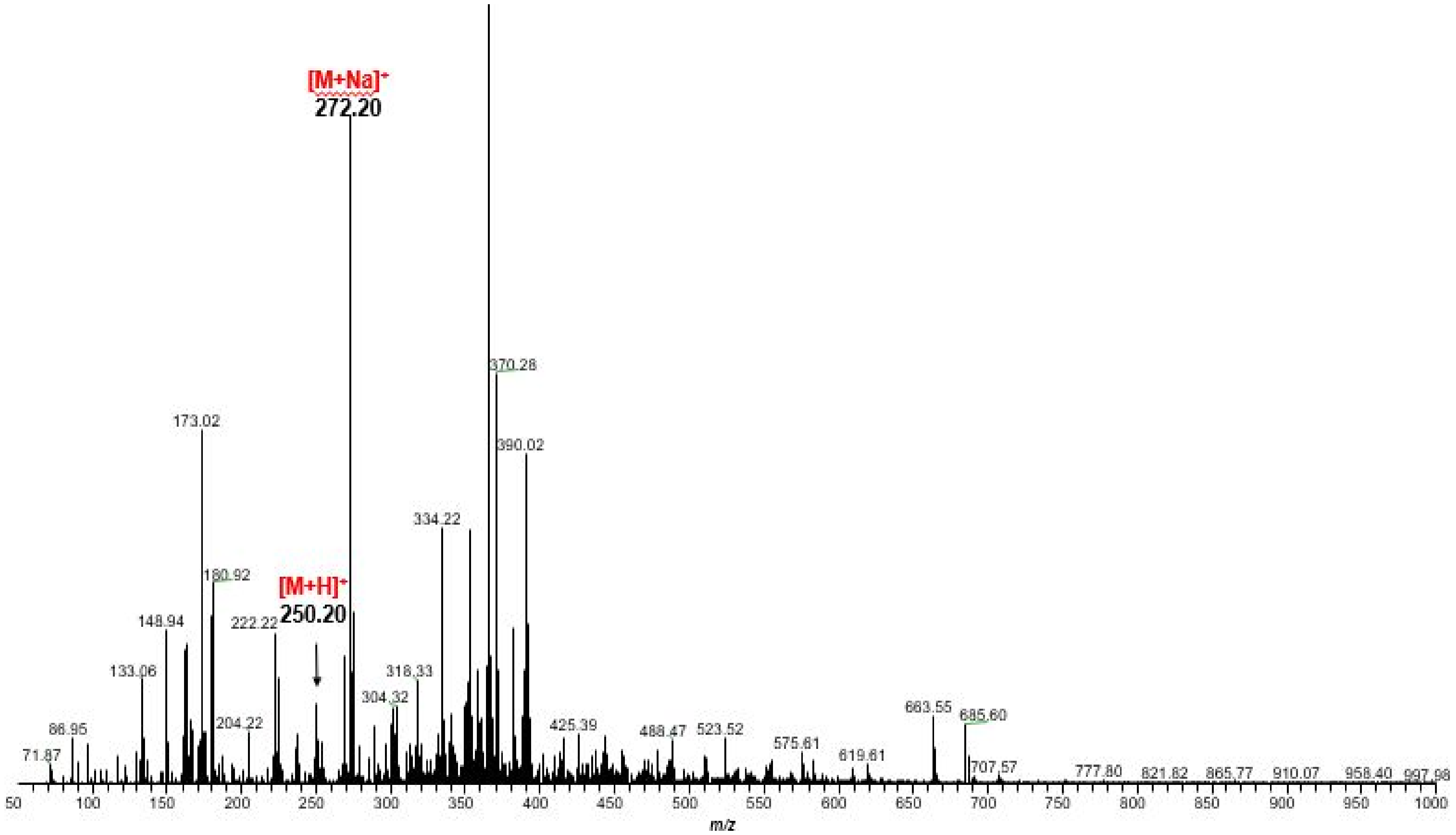

Figure S8. LC-MS spectrum of 1-azido-3-(2-isopropyl-5-methylphenoxy) propan-2-ol (**2**).

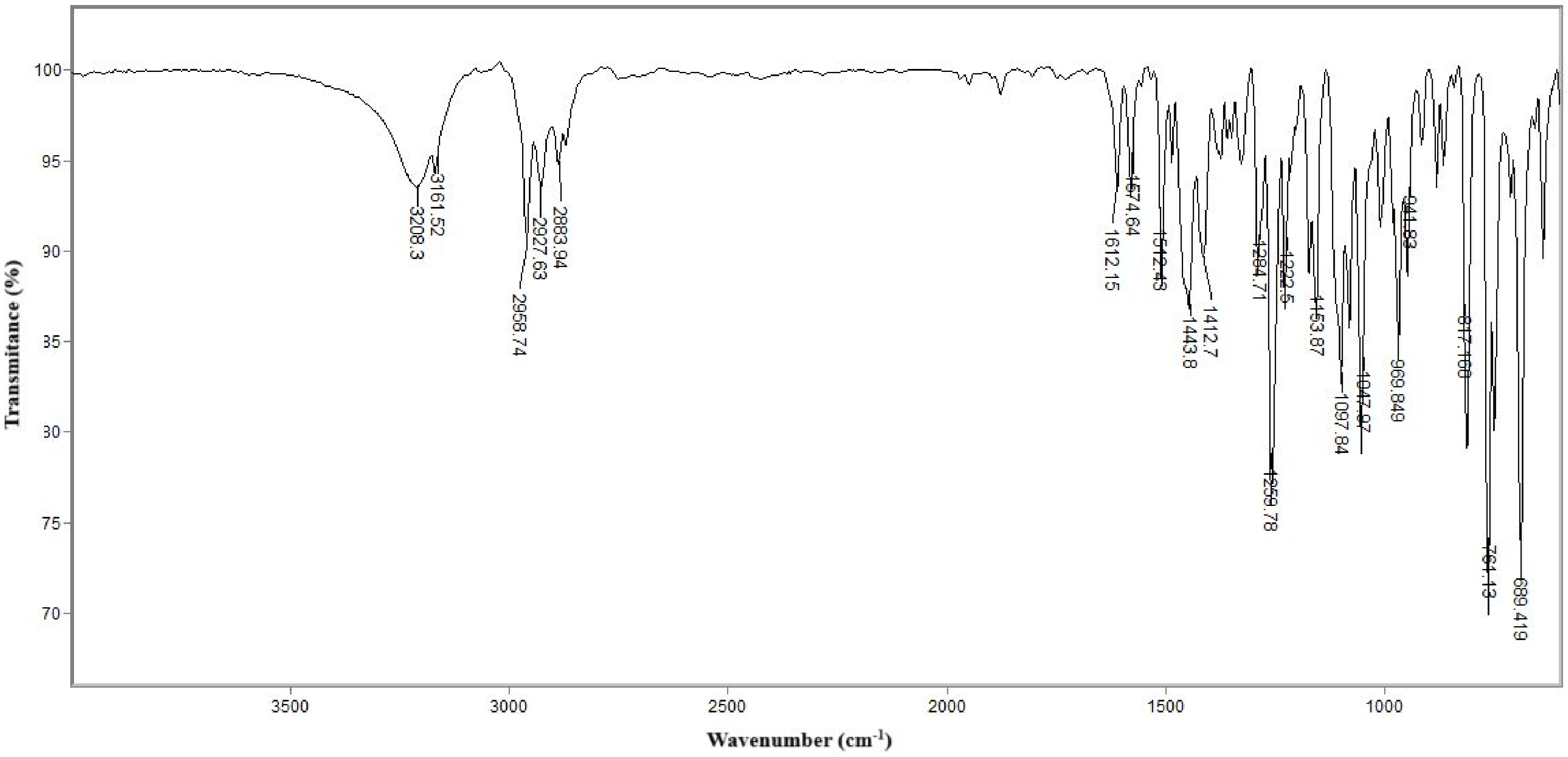

Figure S9. FTIR spectrum (ATR) of 1-(2-isopropyl-5-methylphenoxy)-3-(4-phenyl-1*H*-1,2,3-triazol-1-yl) propan-2-ol (**3a**).

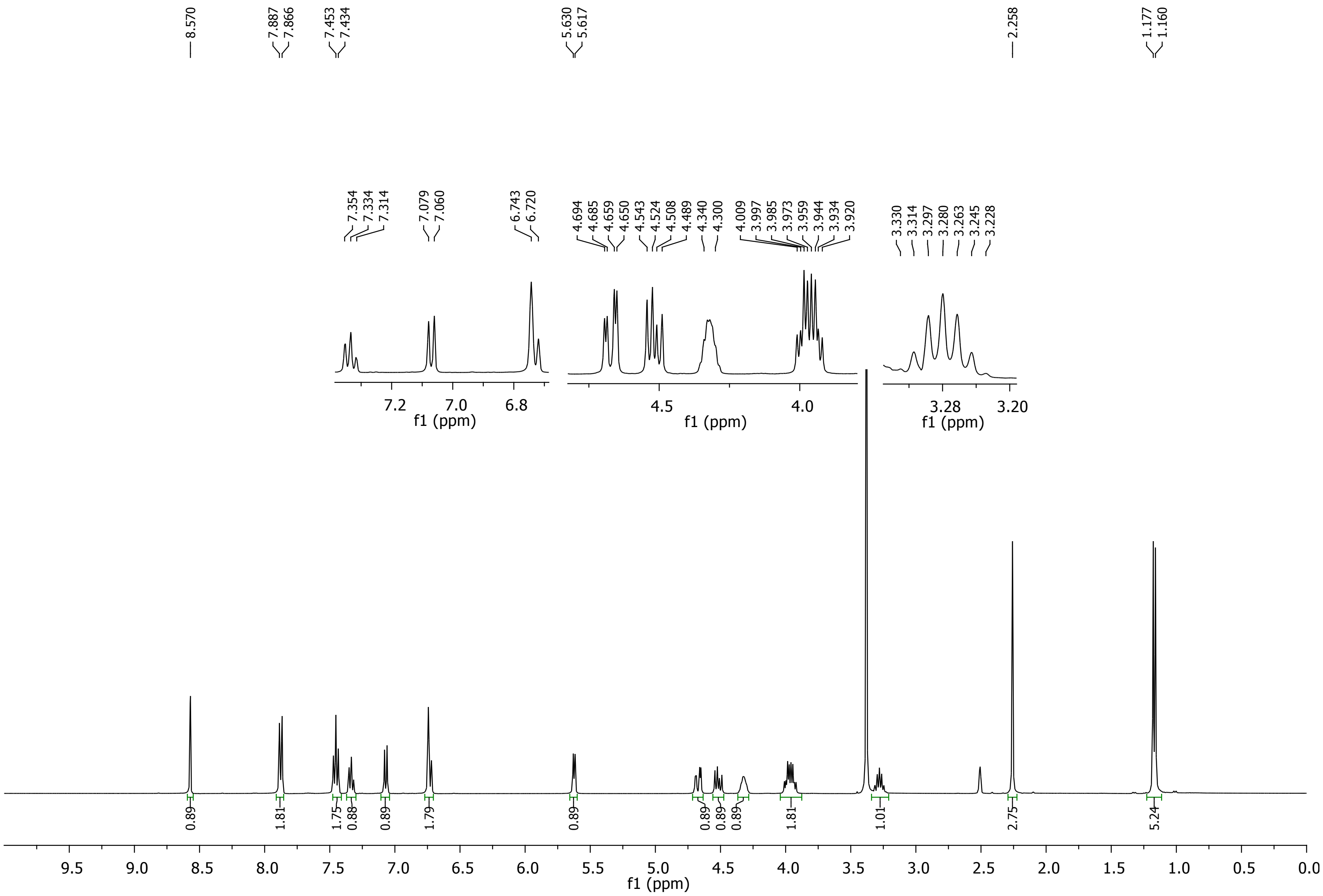

Figure S10. ^{1}H NMR spectrum (400 MHz, DMSO-d_6) of 1-(2-isopropyl-5-methylphenoxy)-3-(4-phenyl-1*H*-1,2,3-triazol-1-yl) propan-2-ol (**3a**).

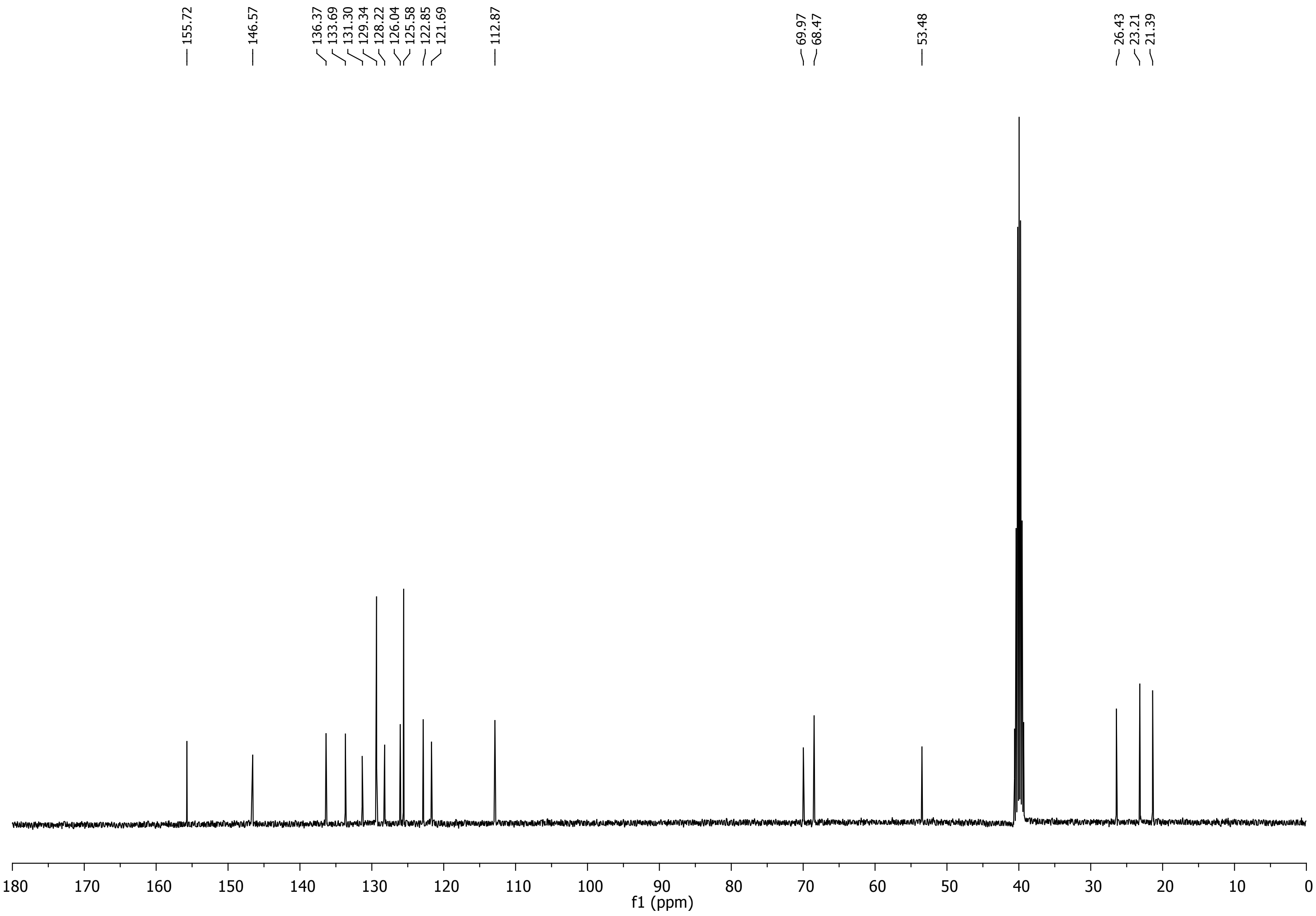

Figure S11. ^{13}C NMR spectrum (100 MHz, DMSO-d_6) of 1-(2-isopropyl-5-methylphenoxy)-3-(4-phenyl-1*H*-1,2,3-triazol-1-yl) propan-2-ol (**3a**).

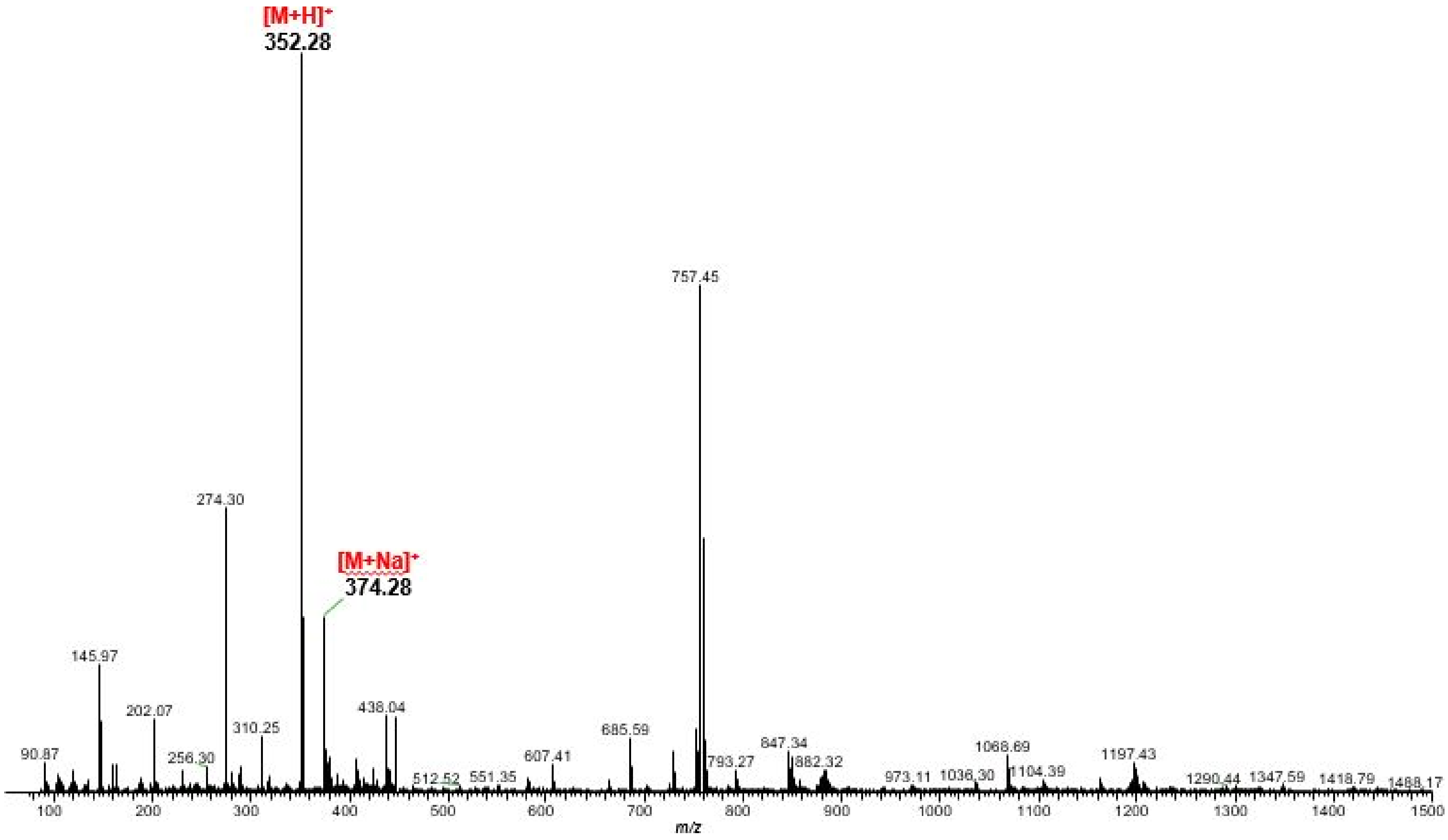

Figure S12. LC-MS spectrum of 1-(2-isopropyl-5-methylphenoxy)-3-(4-phenyl-1*H*-1,2,3-triazol-1-yl)propan-2-ol (**3a**).

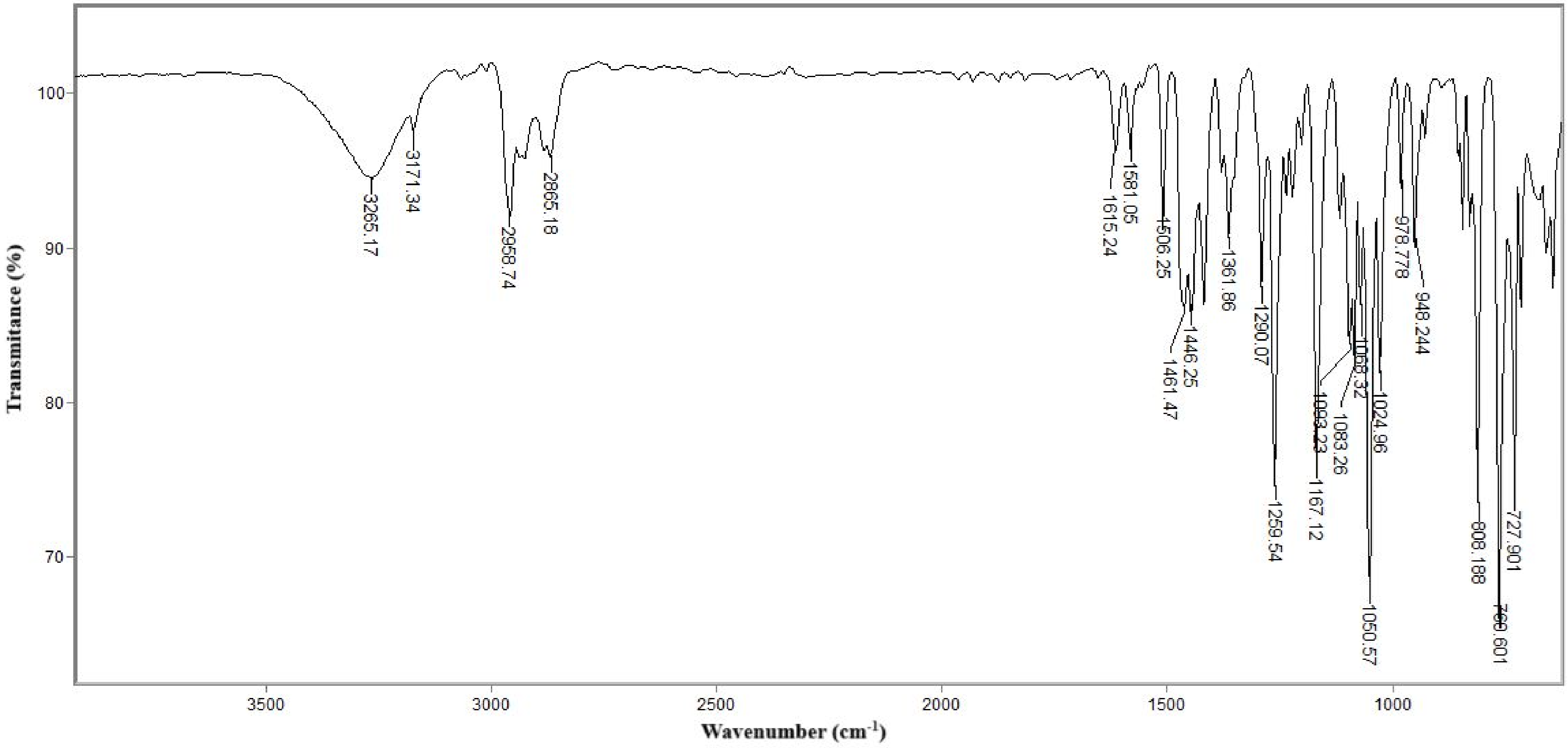

Figure S13. FTIR spectrum (ATR) of 1-(4-(2-bromophenyl)-1*H*-1,2,3-triazol-1-yl)-3-(2-isopropyl-5-methylphenoxy) propan-2-ol (**3b**).

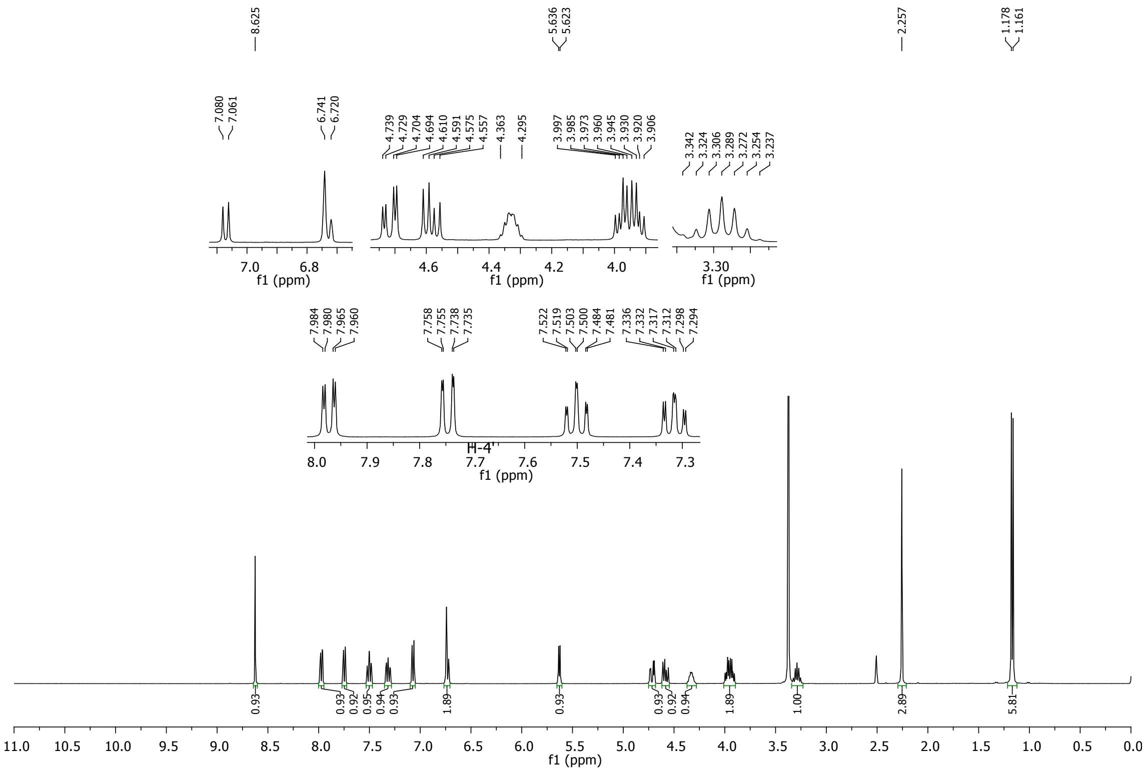

Figure S14. ^{1}H NMR spectrum (400 MHz, DMSO-d_6) of 1-(4-(2-bromophenyl)-1*H*-1,2,3-triazol-1-yl)-3-(2-isopropyl-5-methylphenoxy) propan-2-ol (**3b**).

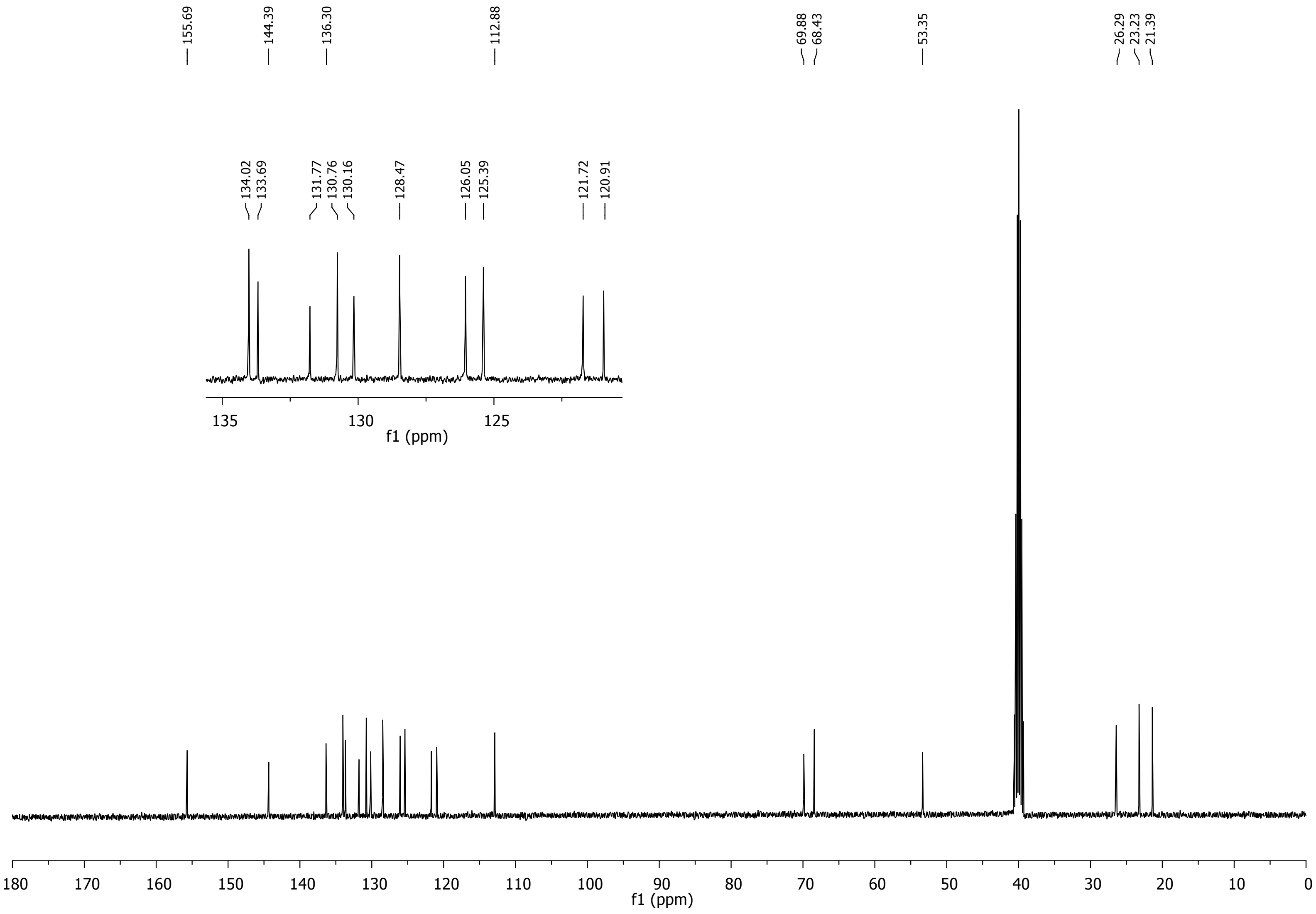

Figure S15. ^{13}C NMR spectrum (100 MHz, DMSO-d_6) of 1-(4-(2-bromophenyl)-1*H*-1,2,3-triazol-1-yl)-3-(2-isopropyl-5-methylphenoxy) propan-2-ol (**3b**).

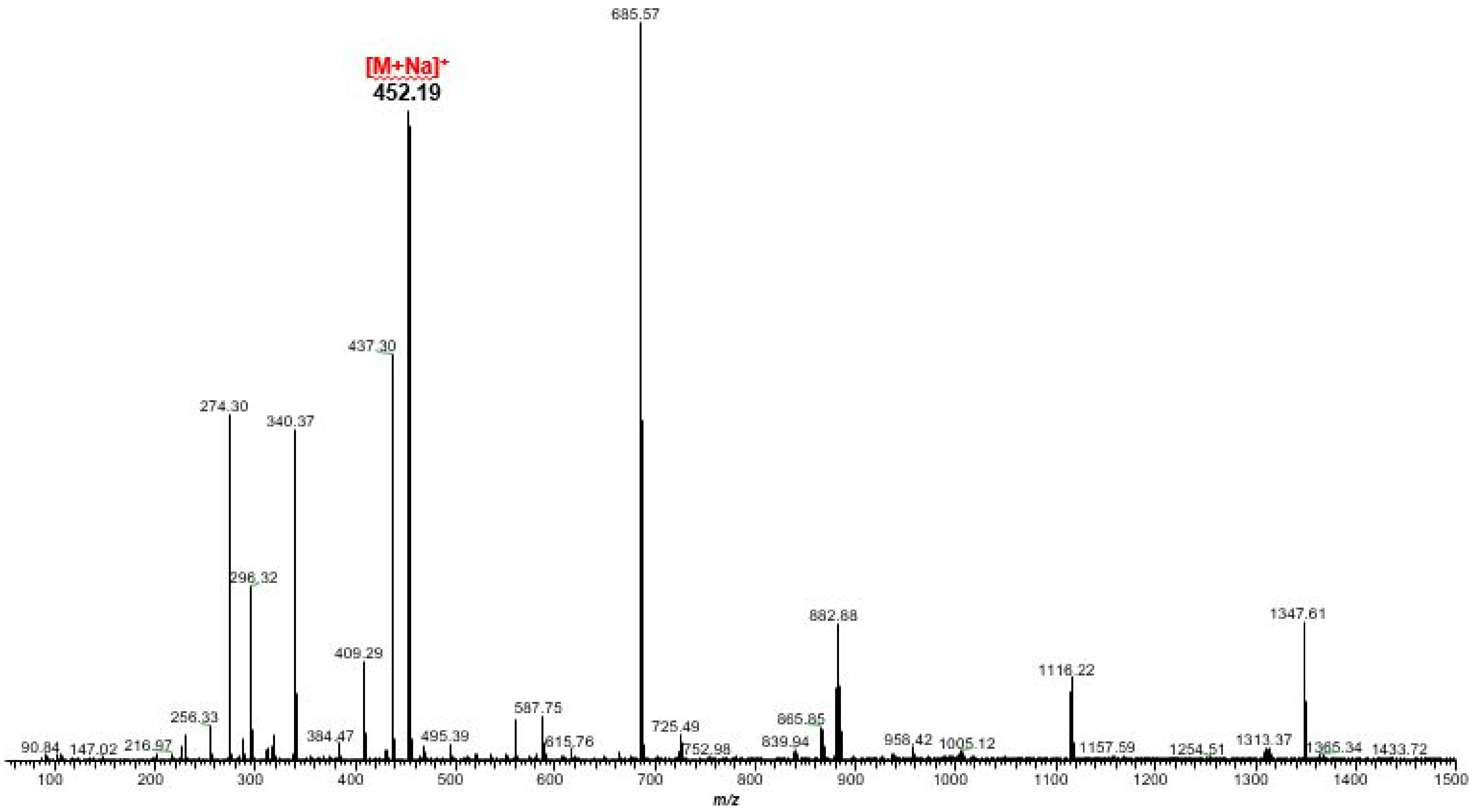

Figure S16. LC-MS spectrum of 1-(4-(2-bromophenyl)-1*H*-1,2,3-triazol-1-yl)-3-(2-isopropyl-5-methylphenoxy) propan-2-ol (**3b**).

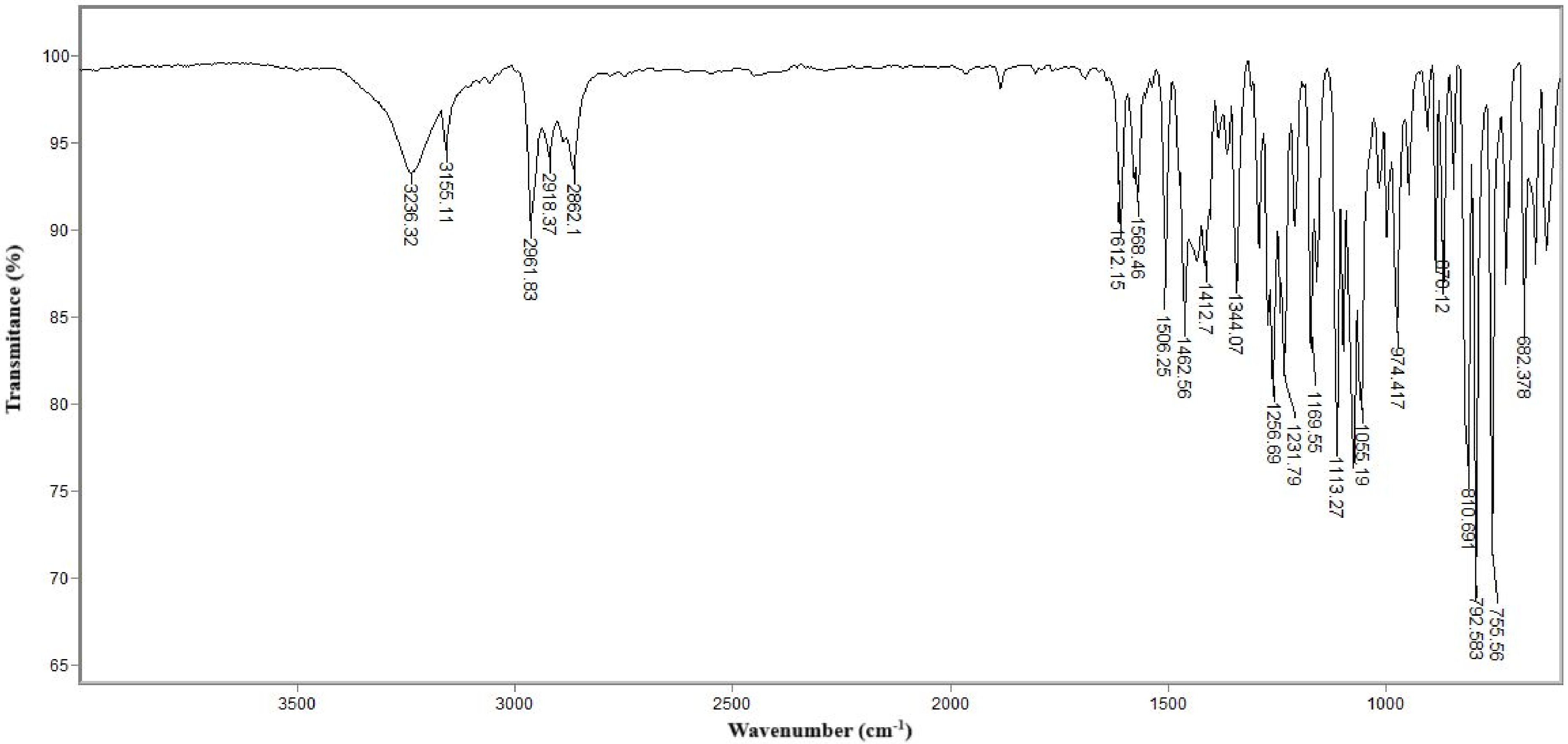

Figure S17. FTIR spectrum (ATR) of 1-(4-(3-bromophenyl)-1*H*-1,2,3-triazol-1-yl)-3-(2-isopropyl-5-methylphenoxy) propan-2-ol (**3c**).

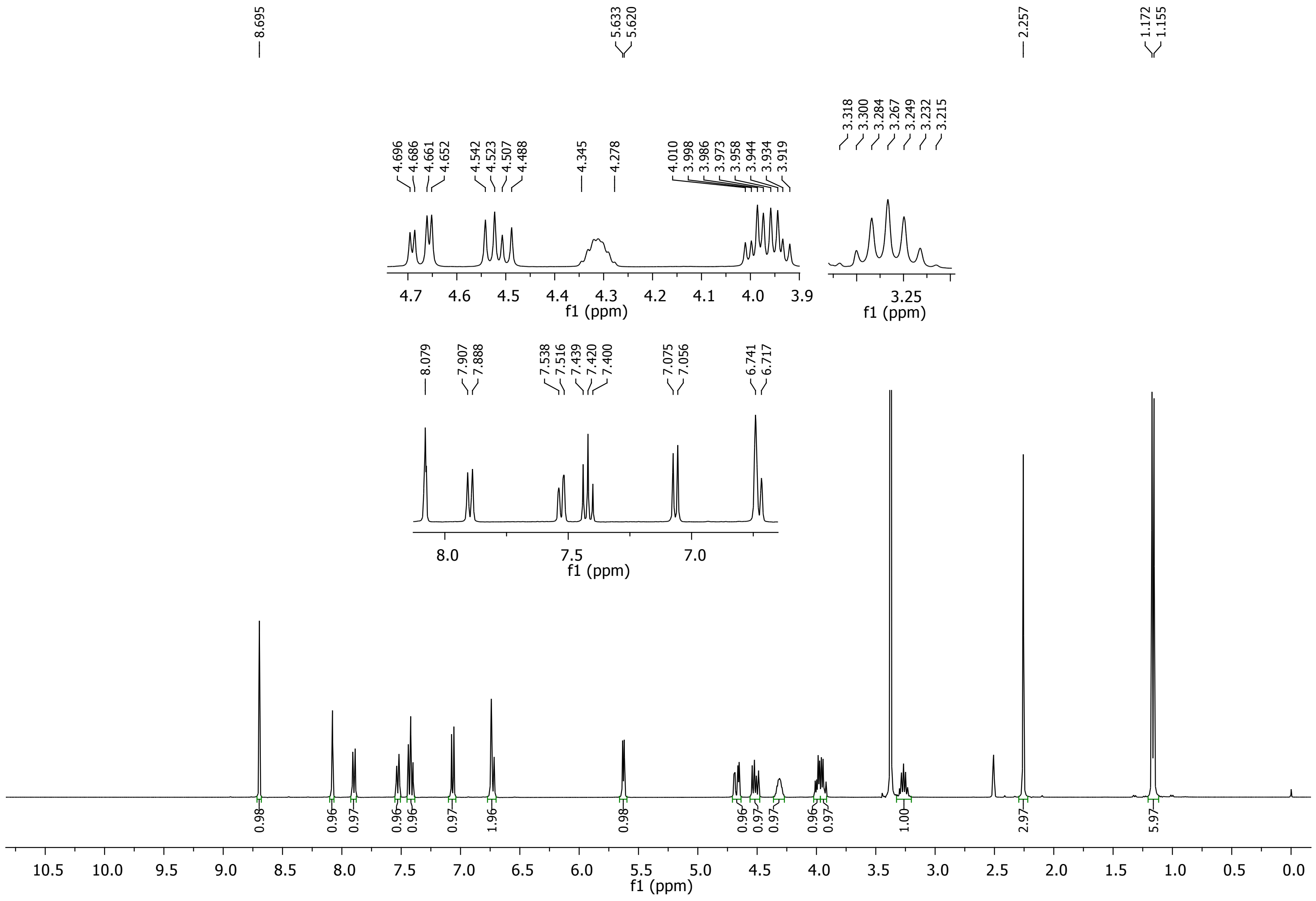

Figure S18. ^{1}H NMR spectrum (400 MHz, DMSO-d_6) of 1-(4-(3-bromophenyl)-1*H*-1,2,3-triazol-1-yl)-3-(2-isopropyl-5-methylphenoxy) propan-2-ol (**3c**).

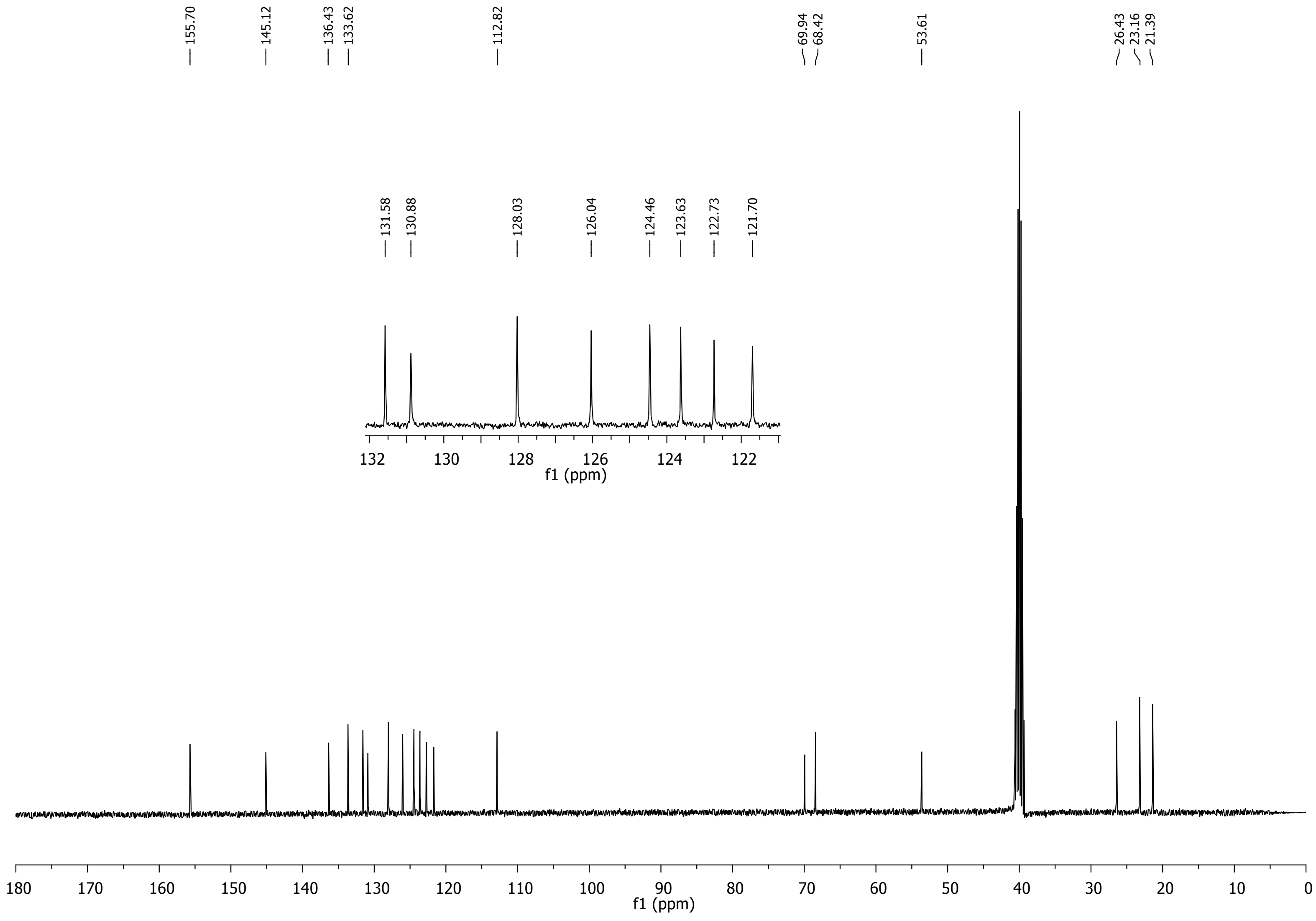

Figure S19. ^{13}C NMR spectrum (100 MHz, DMSO-d_6) of 1-(4-(3-bromophenyl)-1*H*-1,2,3-triazol-1-yl)-3-(2-isopropyl-5-methylphenoxy)propan-2-ol (**3c**).

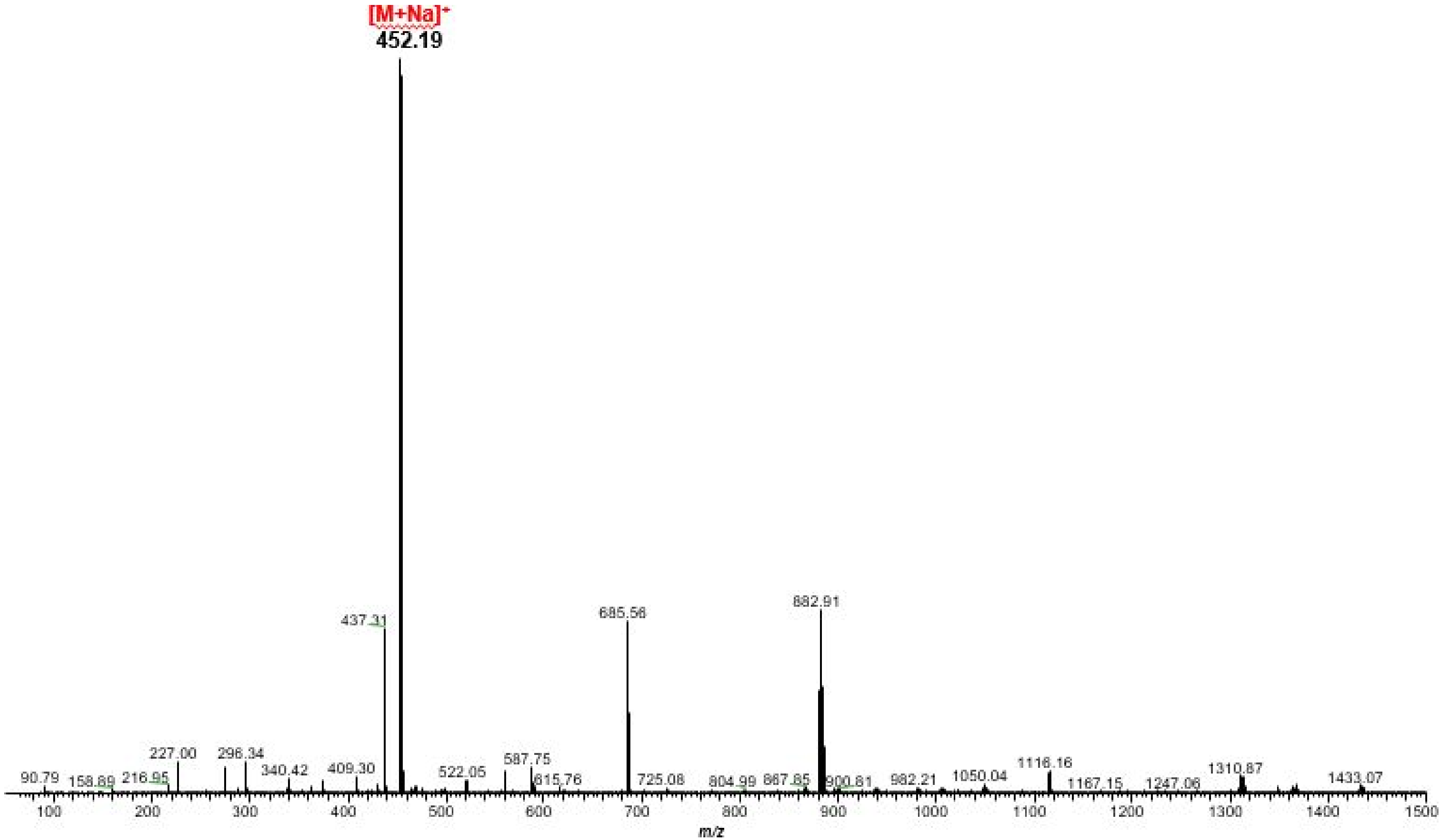

Figure S20. LC-MS spectrum of 1-(4-(3-bromophenyl)-1*H*-1,2,3-triazol-1-yl)-3-(2-isopropyl-5-methylphenoxy) propan-2-ol (**3c**).

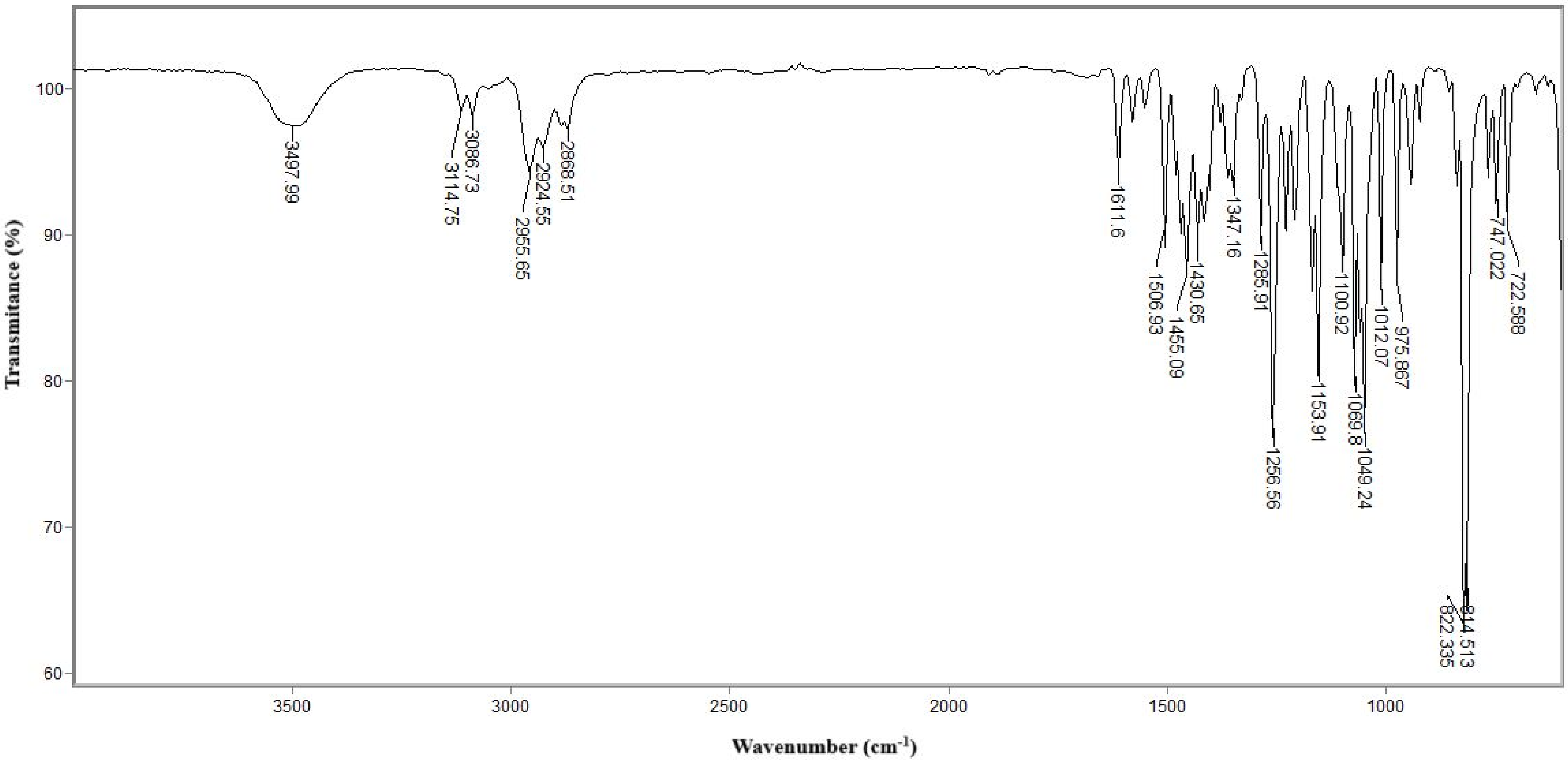

Figure S21. FTIR spectrum (ATR) of 1-(4-(4-bromophenyl)-1*H*-1,2,3-triazol-1-yl)-3-(2-isopropyl-5-methylphenoxy) propan-2-ol (**3d**).

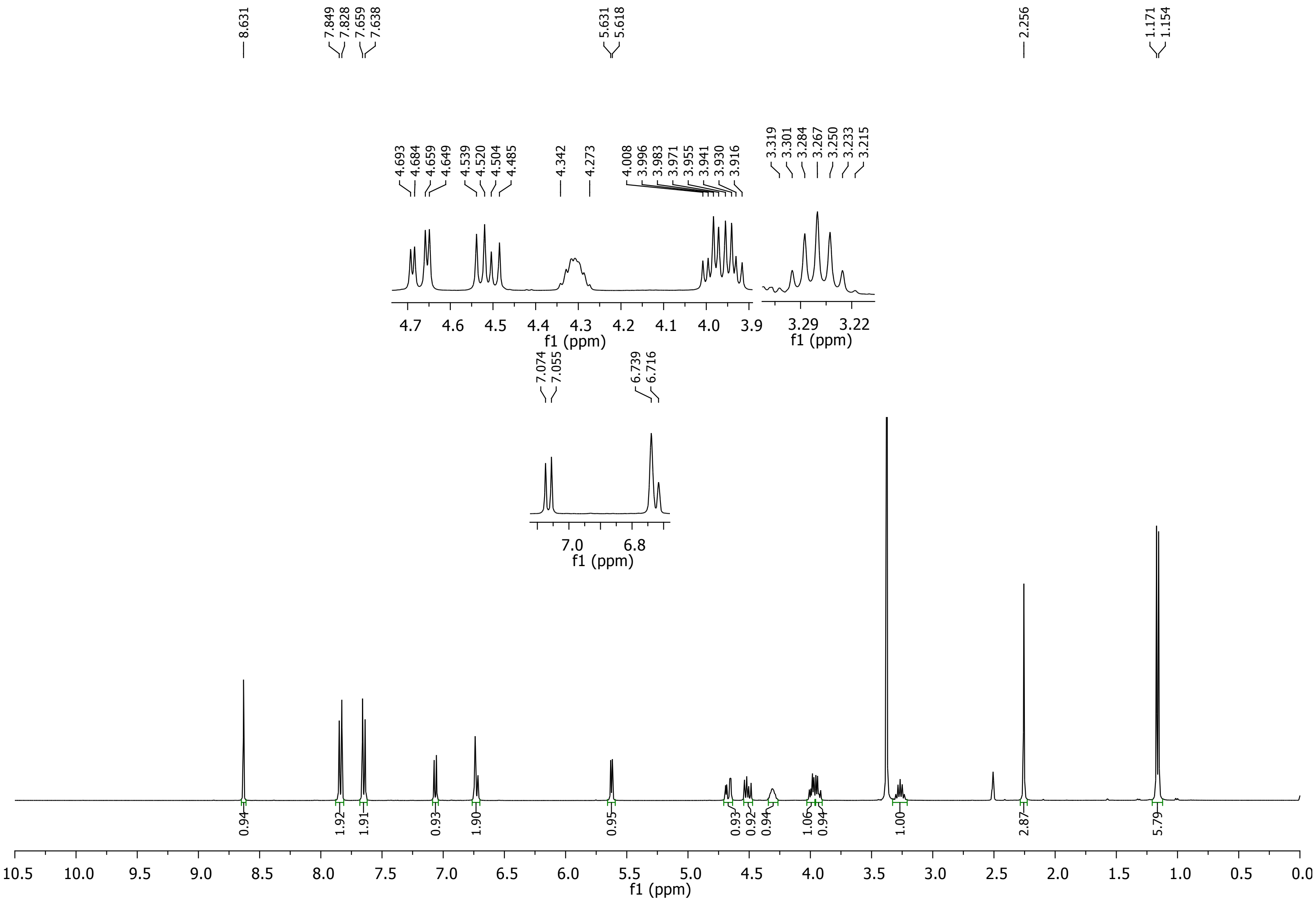

Figure S22. ^{1}H NMR spectrum (400 MHz, DMSO-d_6) of 1-(4-(4-bromophenyl)-1*H*-1,2,3-triazol-1-yl)-3-(2-isopropyl-5-methylphenoxy) propan-2-ol (**3d**).

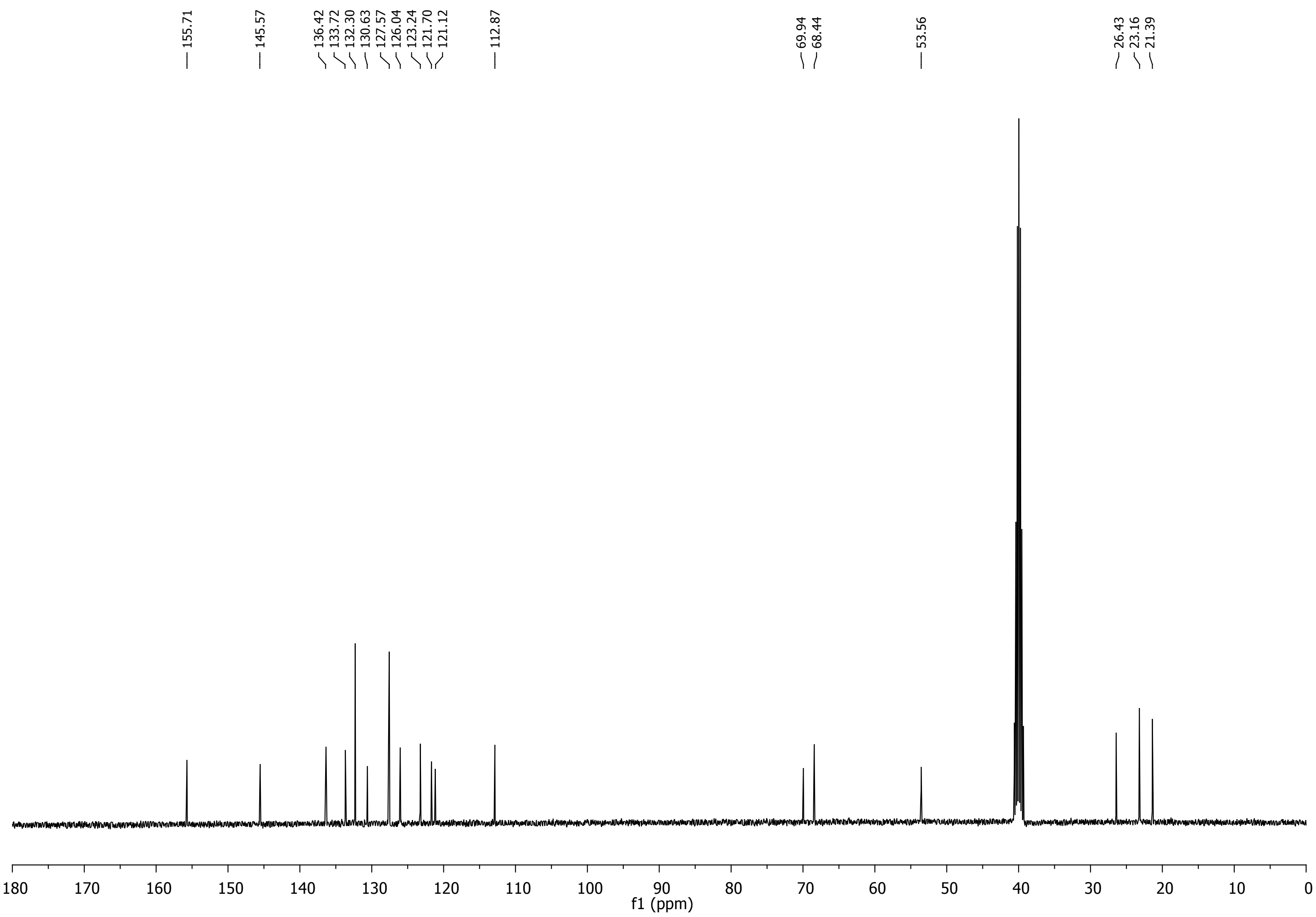

Figure S23. ^{13}C NMR spectrum (100 MHz, DMSO-d_6) of 1-(4-(4-bromophenyl)-1*H*-1,2,3-triazol-1-yl)-3-(2-isopropyl-5-methylphenoxy) propan-2-ol (**3d**).

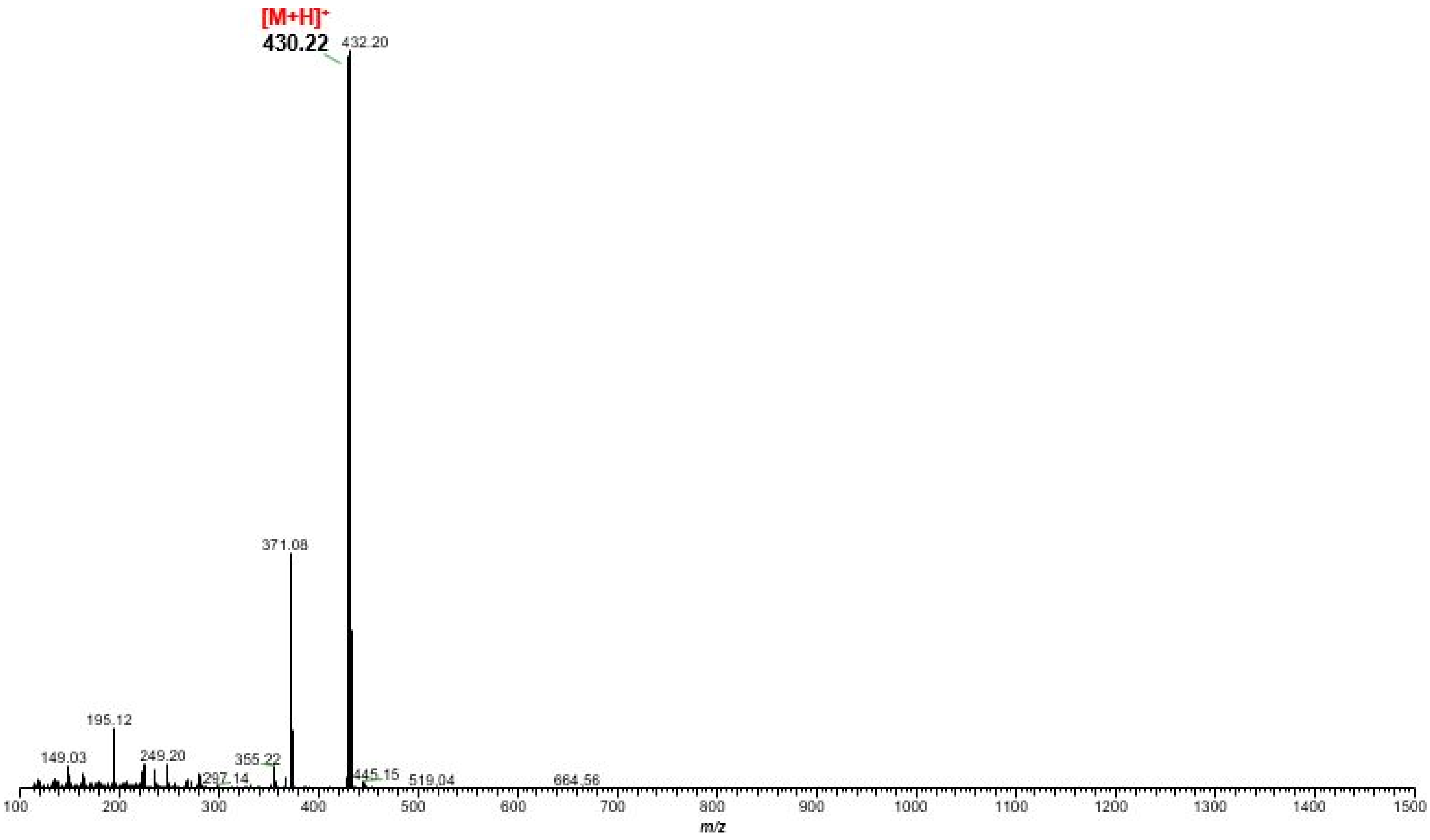

Figure S24. LC-MS spectrum of 1-(4-(4-bromophenyl)-1*H*-1,2,3-triazol-1-yl)-3-(2-isopropyl-5-methylphenoxy) propan-2-ol (**3d**).

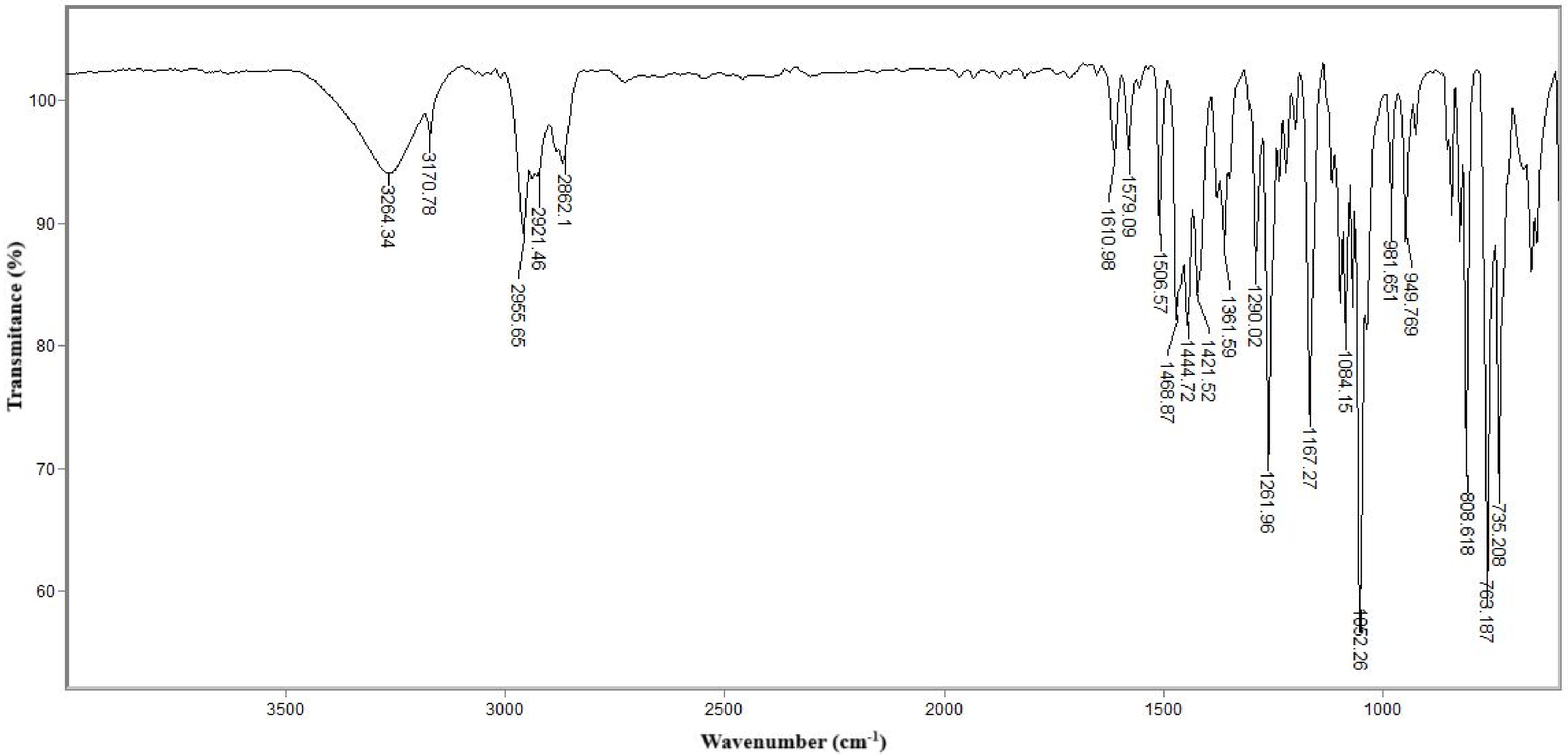

Figure S25. FTIR spectrum (ATR) of 1-(4-(2-chlorophenyl)-1*H*-1,2,3-triazol-1-yl)-3-(2-isopropyl-5-methylphenoxy) propan-2-ol (**3e**).

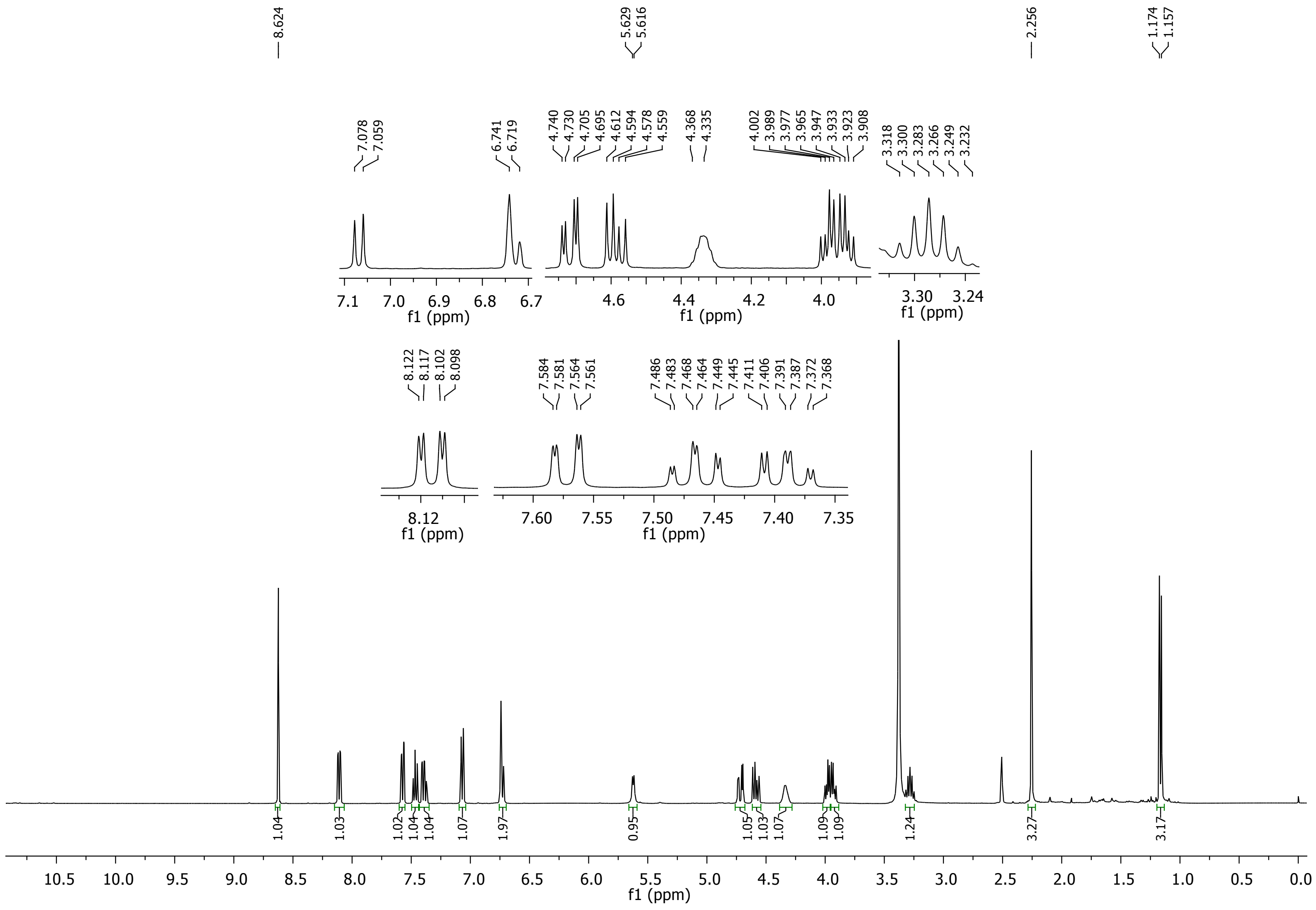

Figure S26. ^{1}H NMR spectrum (400 MHz, DMSO-d_6) of 1-(4-(2-chlorophenyl)-1*H*-1,2,3-triazol-1-yl)-3-(2-isopropyl-5-methylphenoxy) propan-2-ol (**3e**).

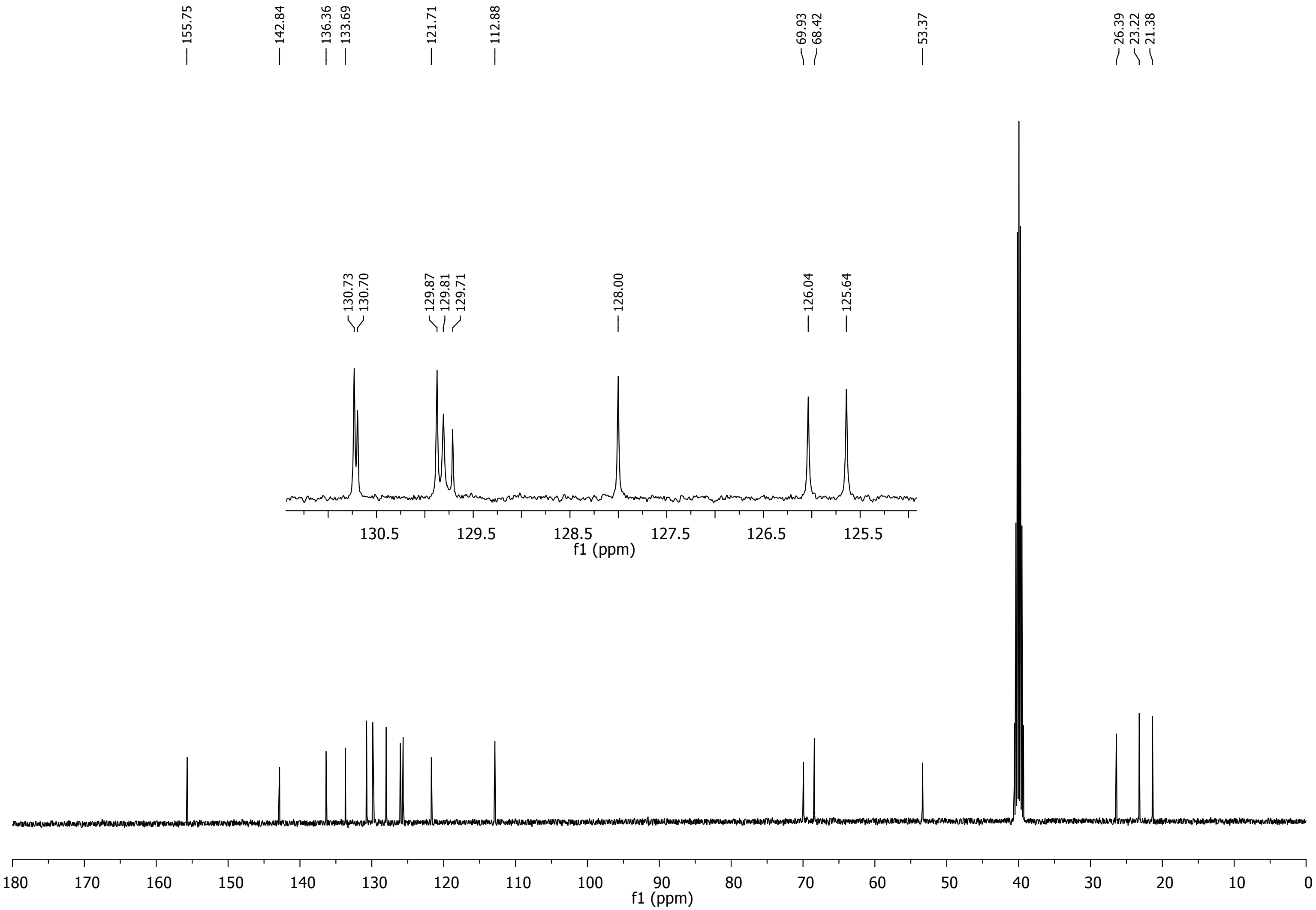

Figure S27. ^{13}C NMR spectrum (100 MHz, DMSO-d_6) of 1-(4-(2-chlorophenyl)-1*H*-1,2,3-triazol-1-yl)-3-(2-isopropyl-5-methylphenoxy)propan-2-ol (**3e**).

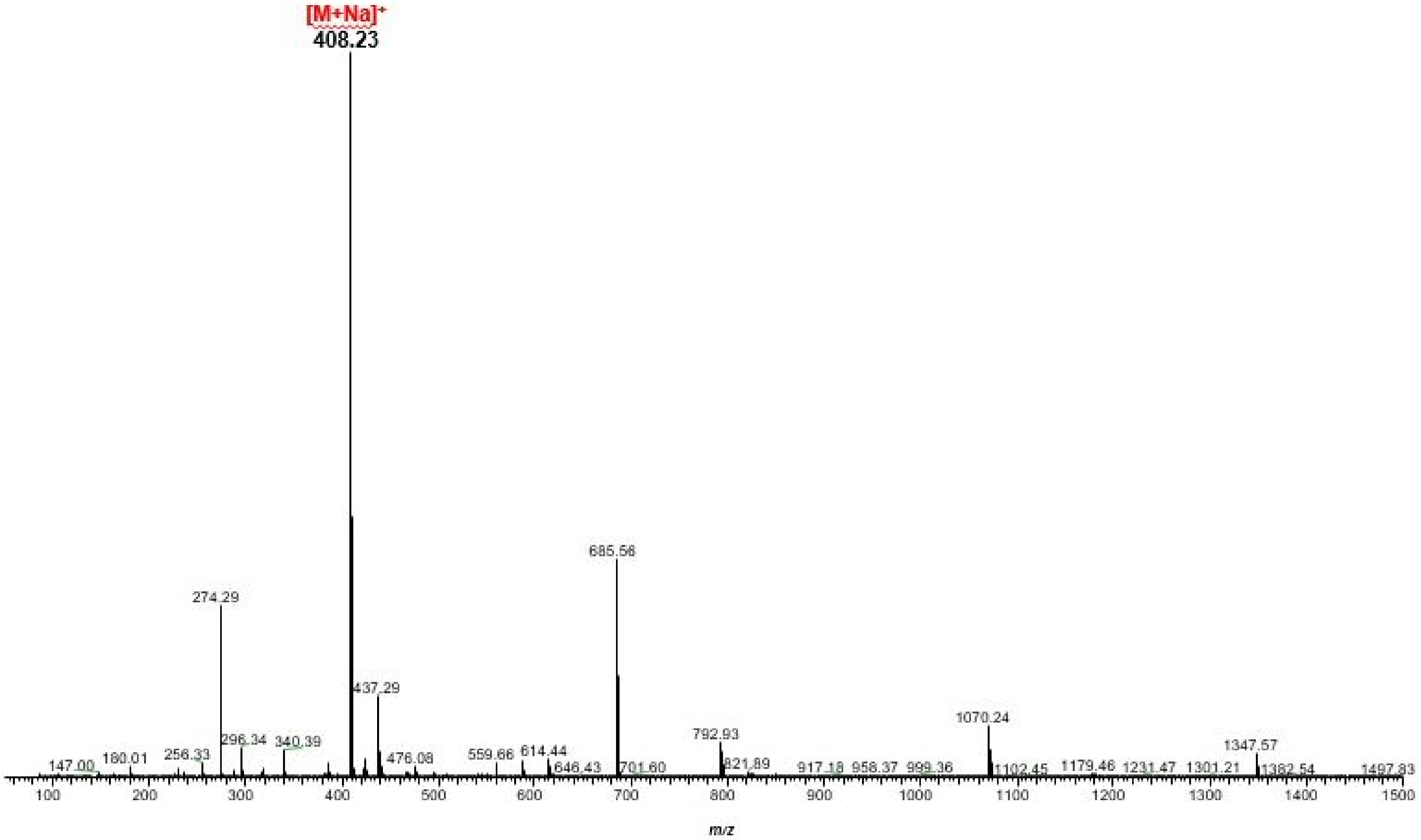

Figure S28. LC-MS spectrum of 1-(4-(2-chlorophenyl)-1*H*-1,2,3-triazol-1-yl)-3-(2-isopropyl-5-methylphenoxy) propan-2-ol (**3e**).

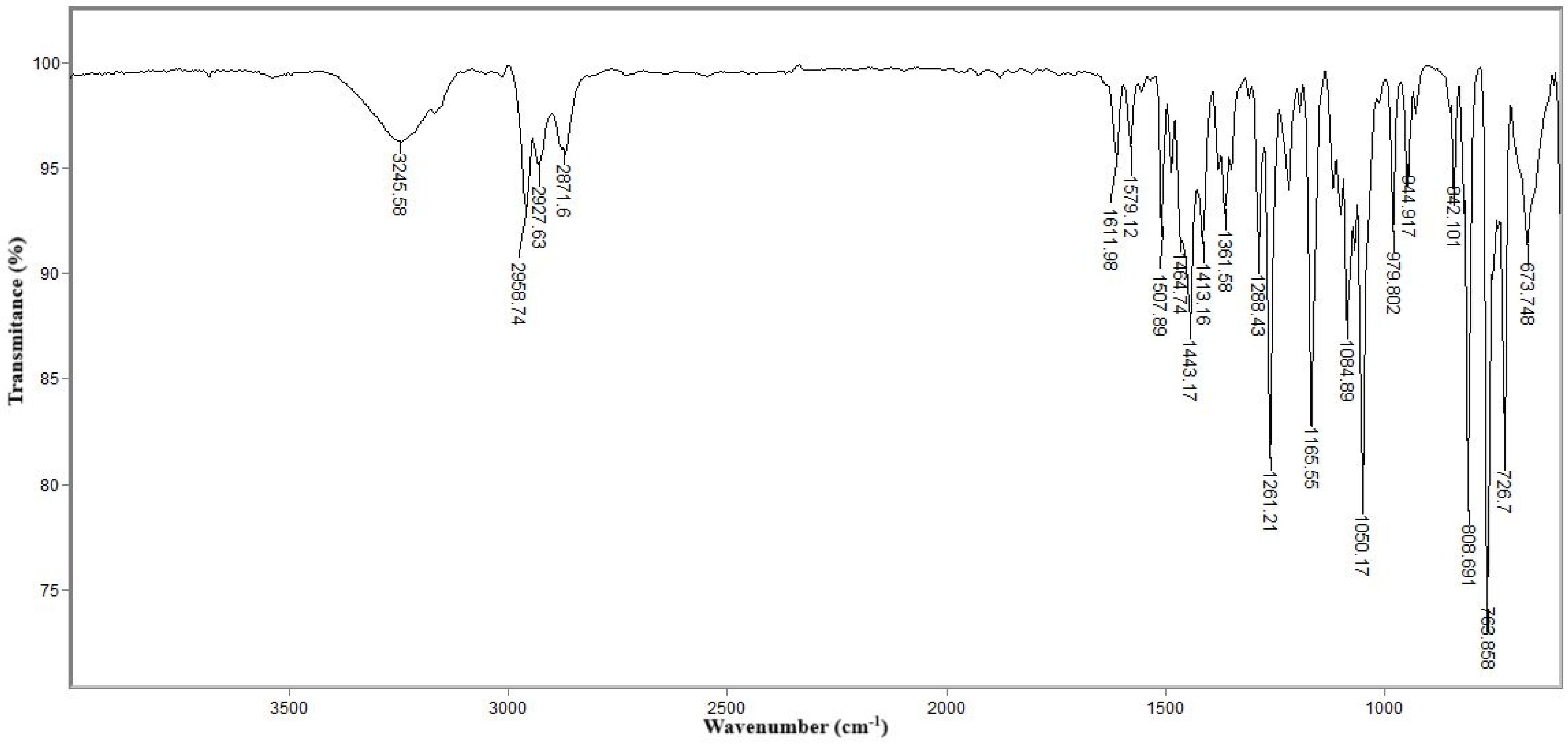

Figure S29. FTIR spectrum (ATR) of 1-(2-isopropyl-5-methylphenoxy)-3-(4-(*o*-tolyl)-1*H*-1,2,3-triazol-1-yl) propan-2-ol (**3f**).

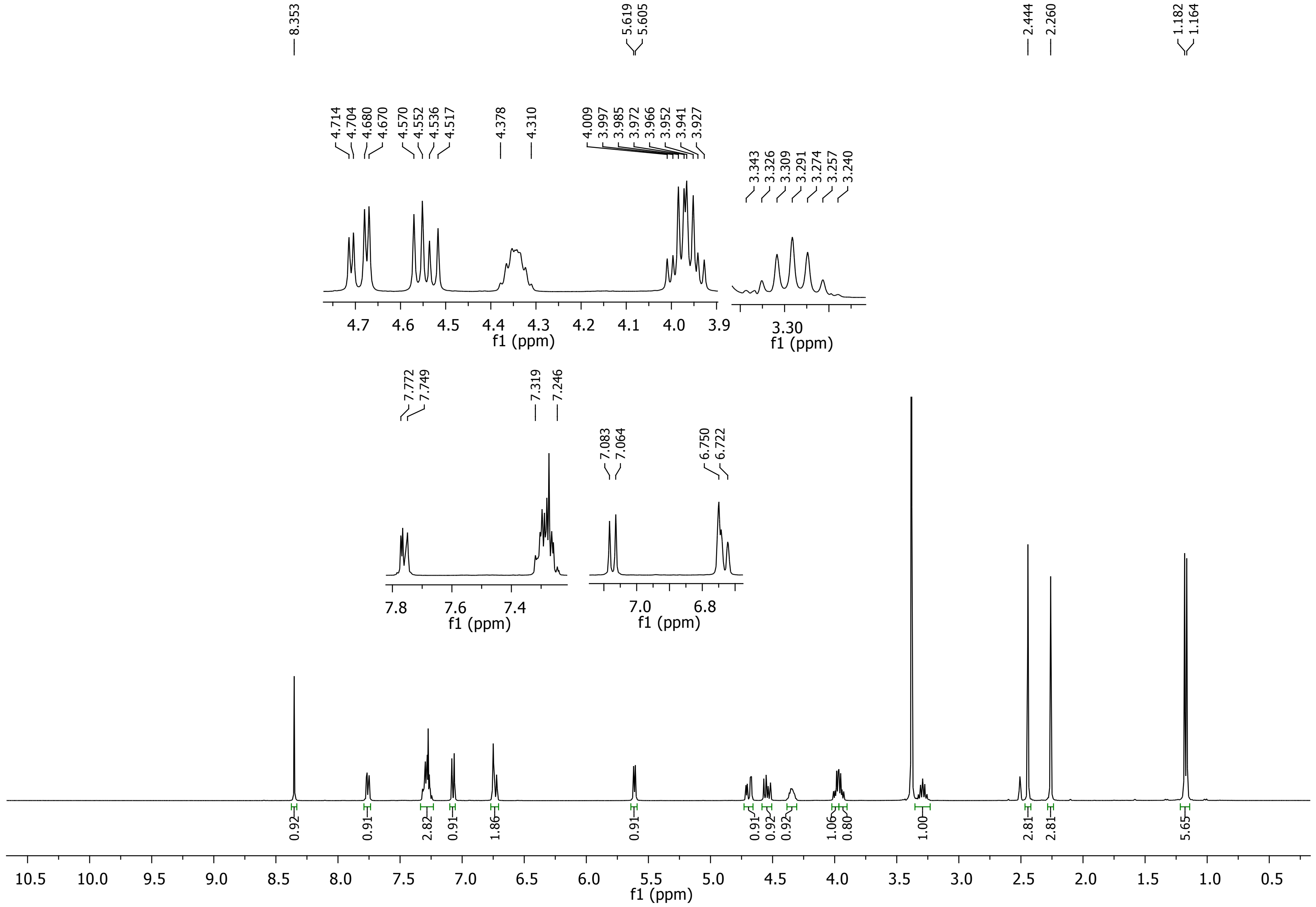

Figure S30. ^{1}H NMR spectrum (400 MHz, DMSO-d_6) of 1-(2-isopropyl-5-methylphenoxy)-3-(4-(*o*-tolyl)-1*H*-1,2,3-triazol-1-yl) propan-2-ol (**3f**).

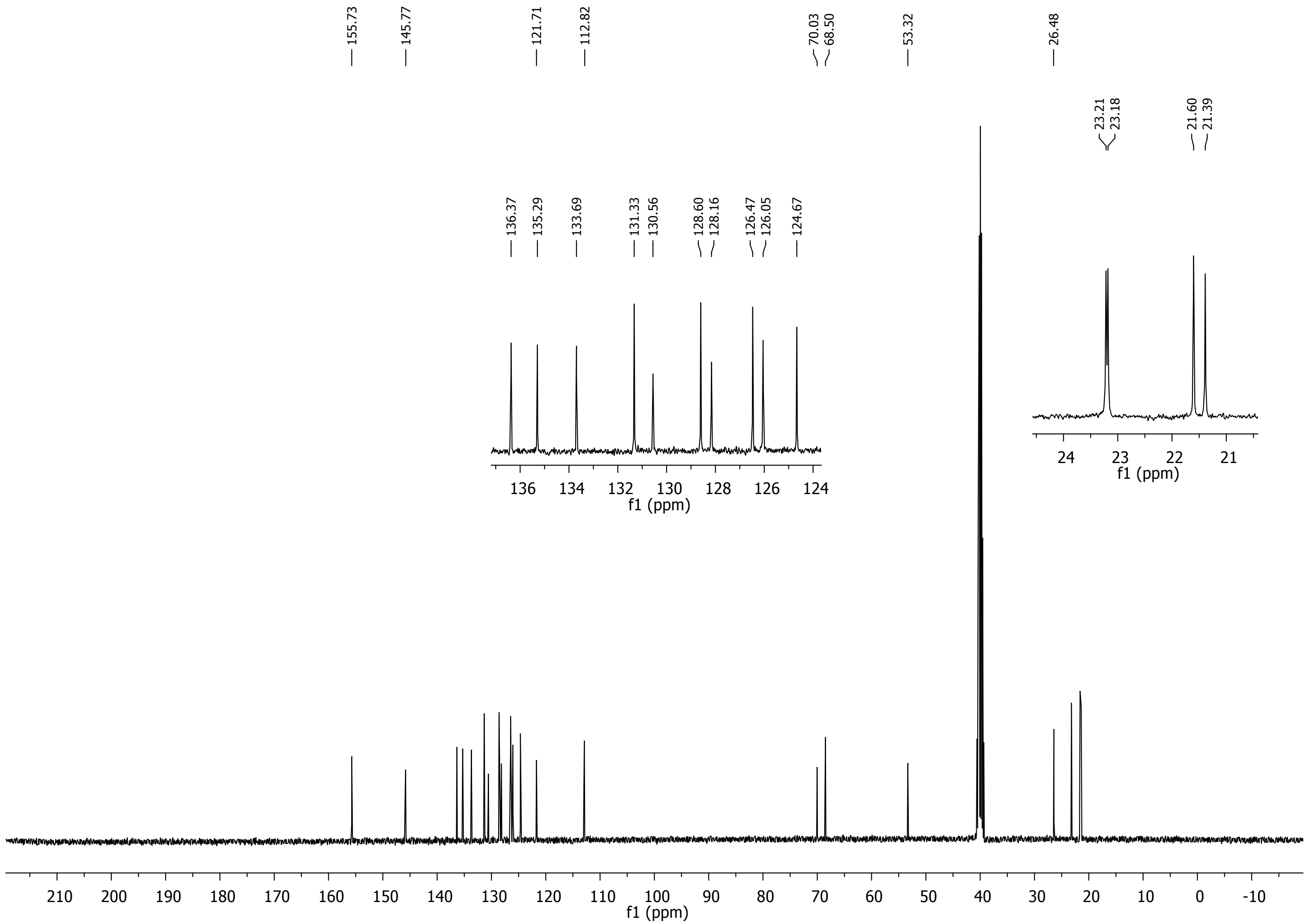

Figure S31. ^{13}C NMR spectrum (100 MHz, DMSO-d_6) of 1-(2-isopropyl-5-methylphenoxy)-3-(4-(*o*-tolyl)-1*H*-1,2,3-triazol-1-yl) propan-2-ol (**3f**)

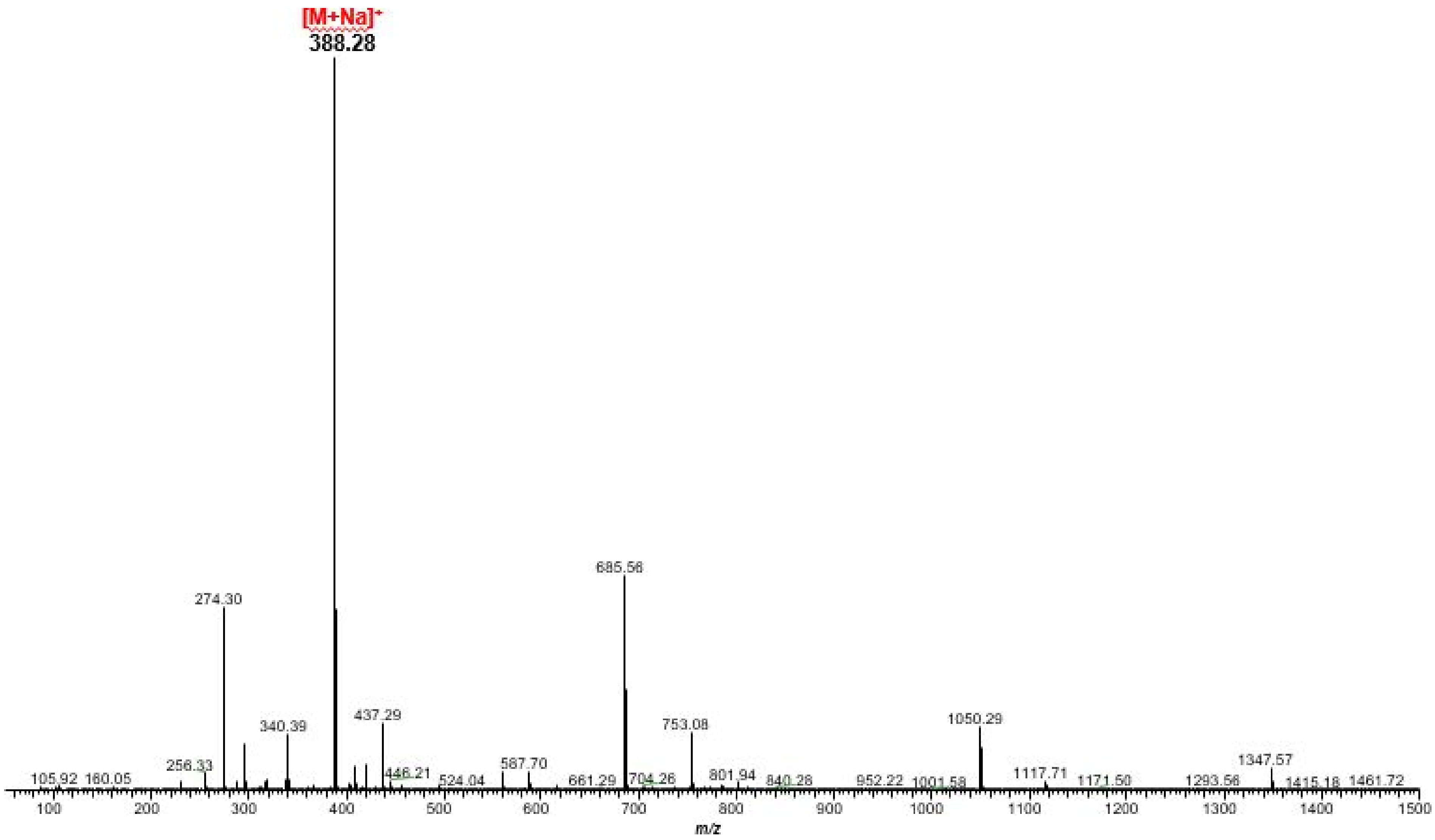

Figure S32. LC-MS spectrum of 1-(2-isopropyl-5-methylphenoxy)-3-(4-(*o*-tolyl)-1*H*-1,2,3-triazol-1-yl) propan-2-ol (**3f**).

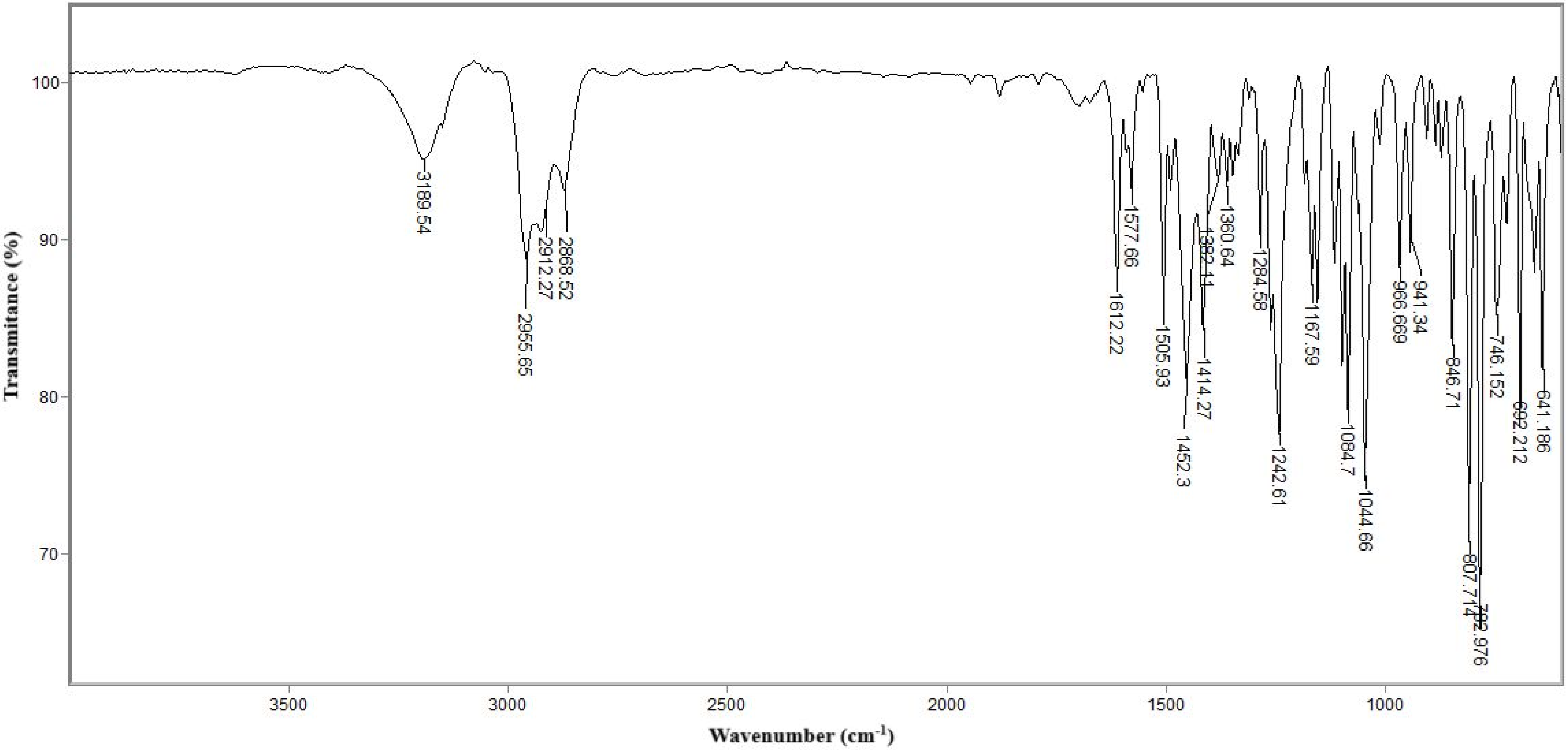

Figure S33. FTIR spectrum (ATR) of 1-(2-isopropyl-5-methylphenoxy)-3-(4-(*m*-tolyl)-1*H*-1,2,3-triazol-1-yl) propan-2-ol (**3g**).

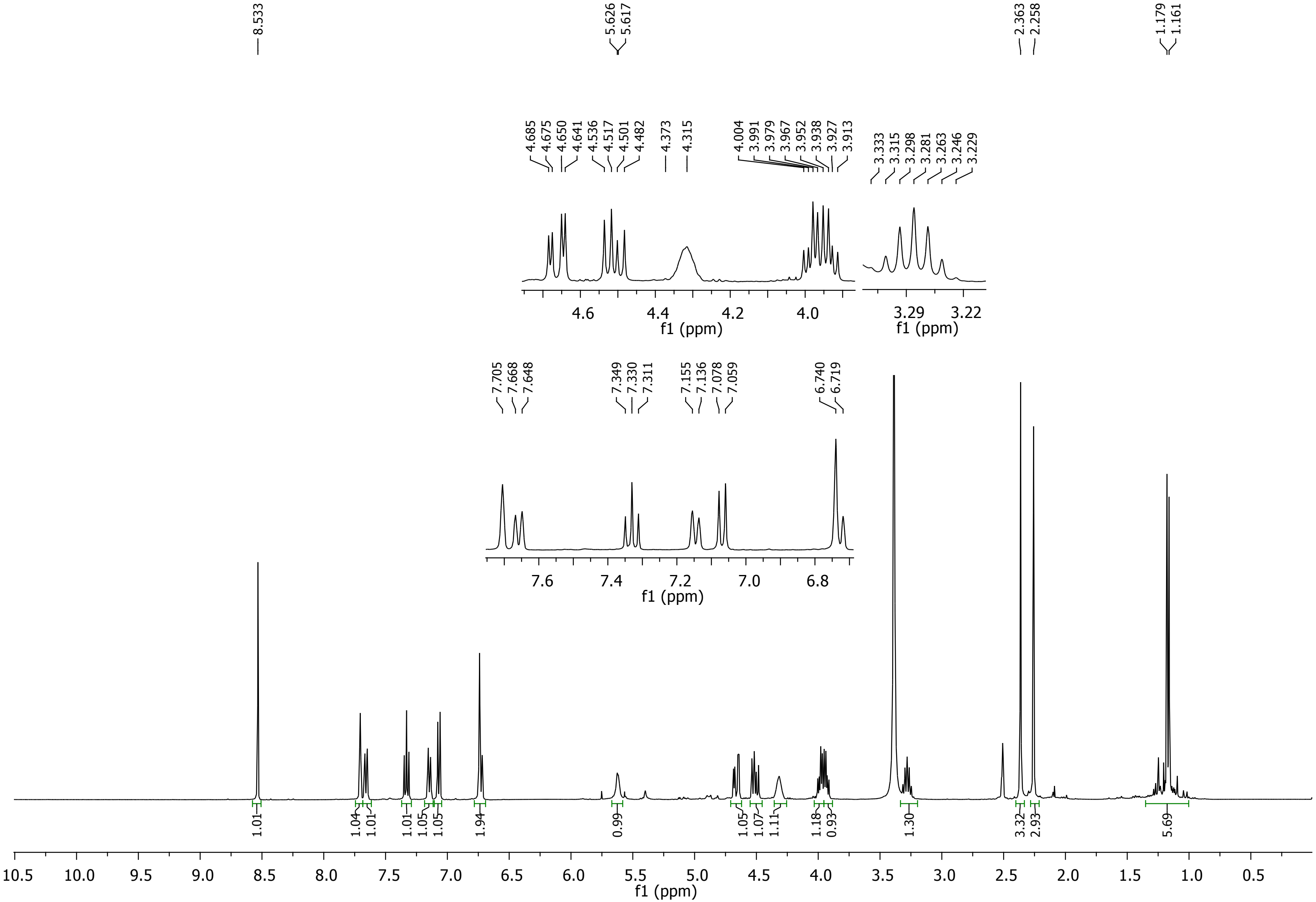

Figure S34. ^{1}H NMR spectrum (400 MHz, DMSO-d_6) of 1-(2-isopropyl-5-methylphenoxy)-3-(4-(*m*-tolyl)-1*H*-1,2,3-triazol-1-yl) propan-2-ol (**3g**).

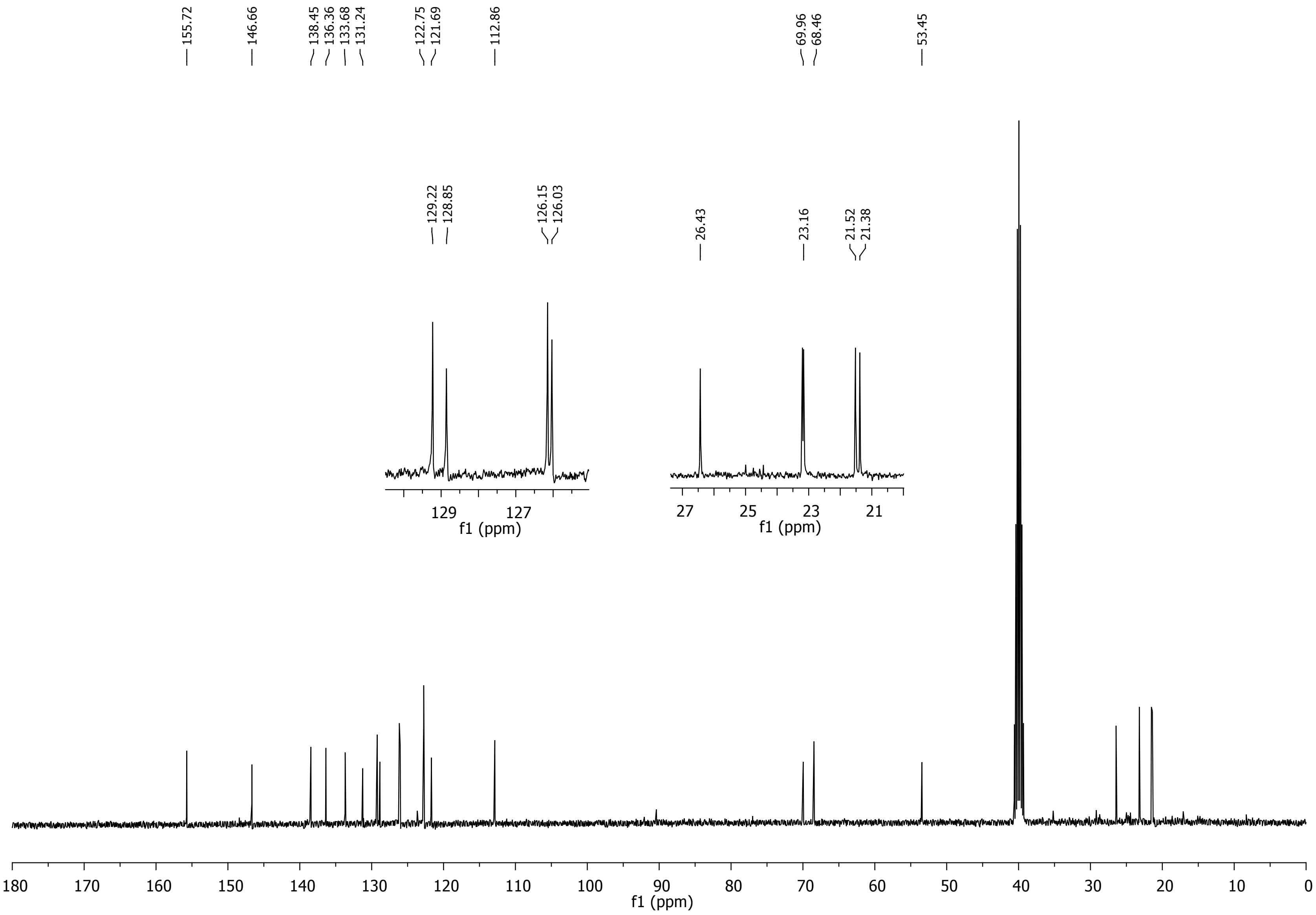

Figure S35. ^{13}C NMR spectrum (100 MHz, DMSO-d_6) of 1-(2-isopropyl-5-methylphenoxy)-3-(4-(*m*-tolyl)-1*H*-1,2,3-triazol-1-yl) propan-2-ol (**3g**).

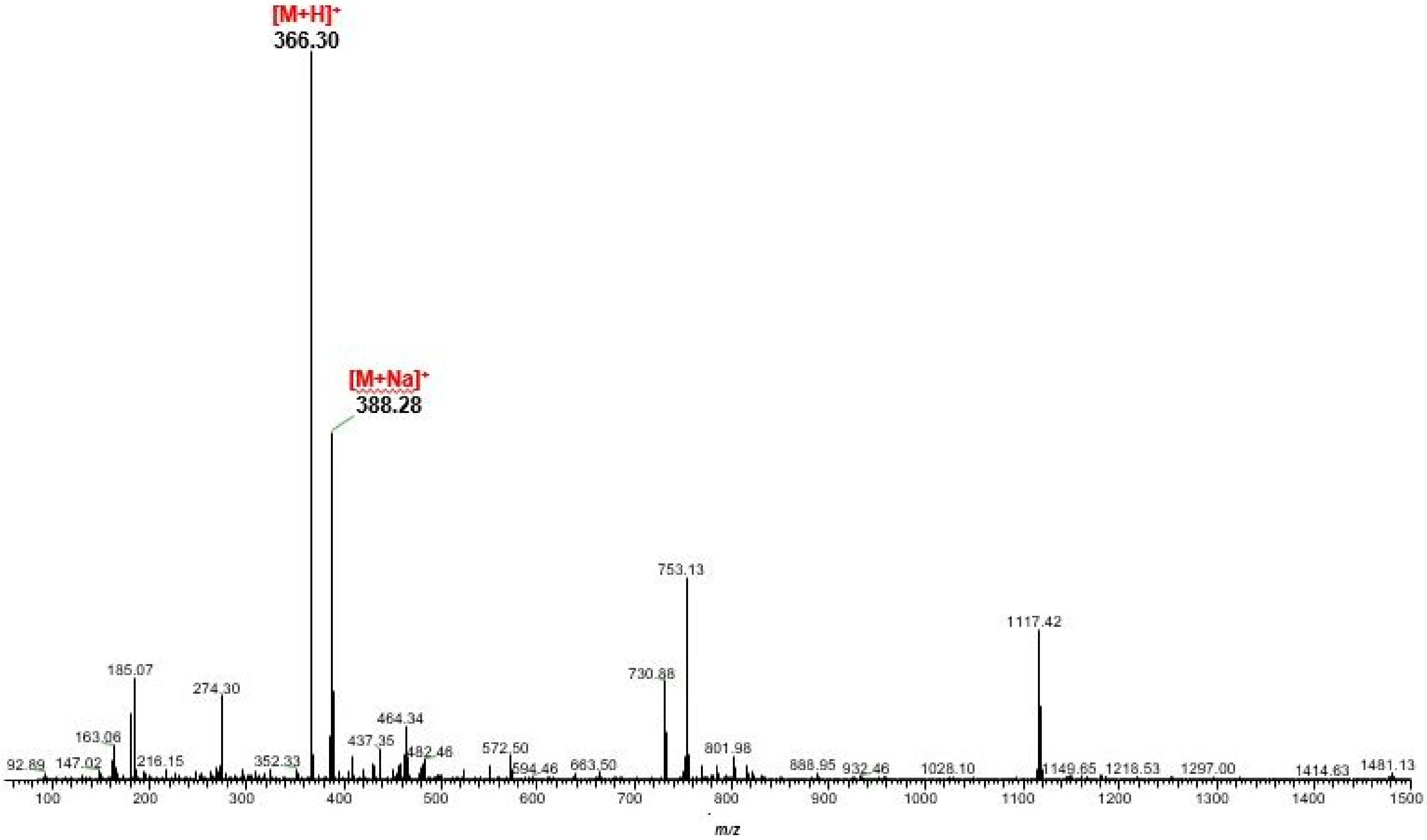

Figure S36. LC-MS spectrum of 1-(2-isopropyl-5-methylphenoxy)-3-(4-(*m*-tolyl)-1*H*-1,2,3-triazol-1-yl) propan-2-ol (**3g**).

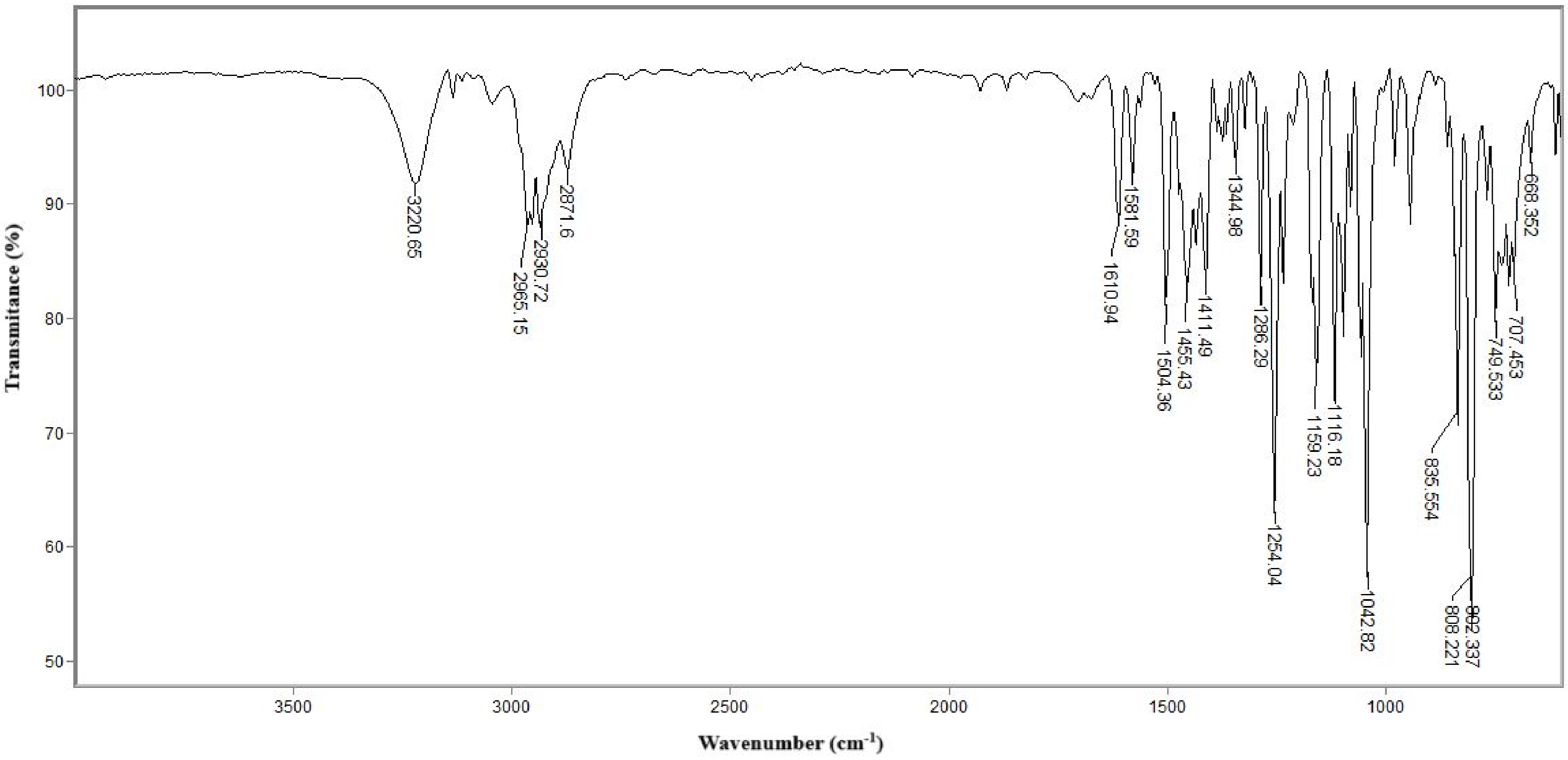

Figure S37. FTIR spectrum (ATR) of 1-(2-isopropyl-5-methylphenoxy)-3-(4-(*p*-tolyl)-1*H*-1,2,3-triazol-1-yl) propan-2-ol (**3h**).

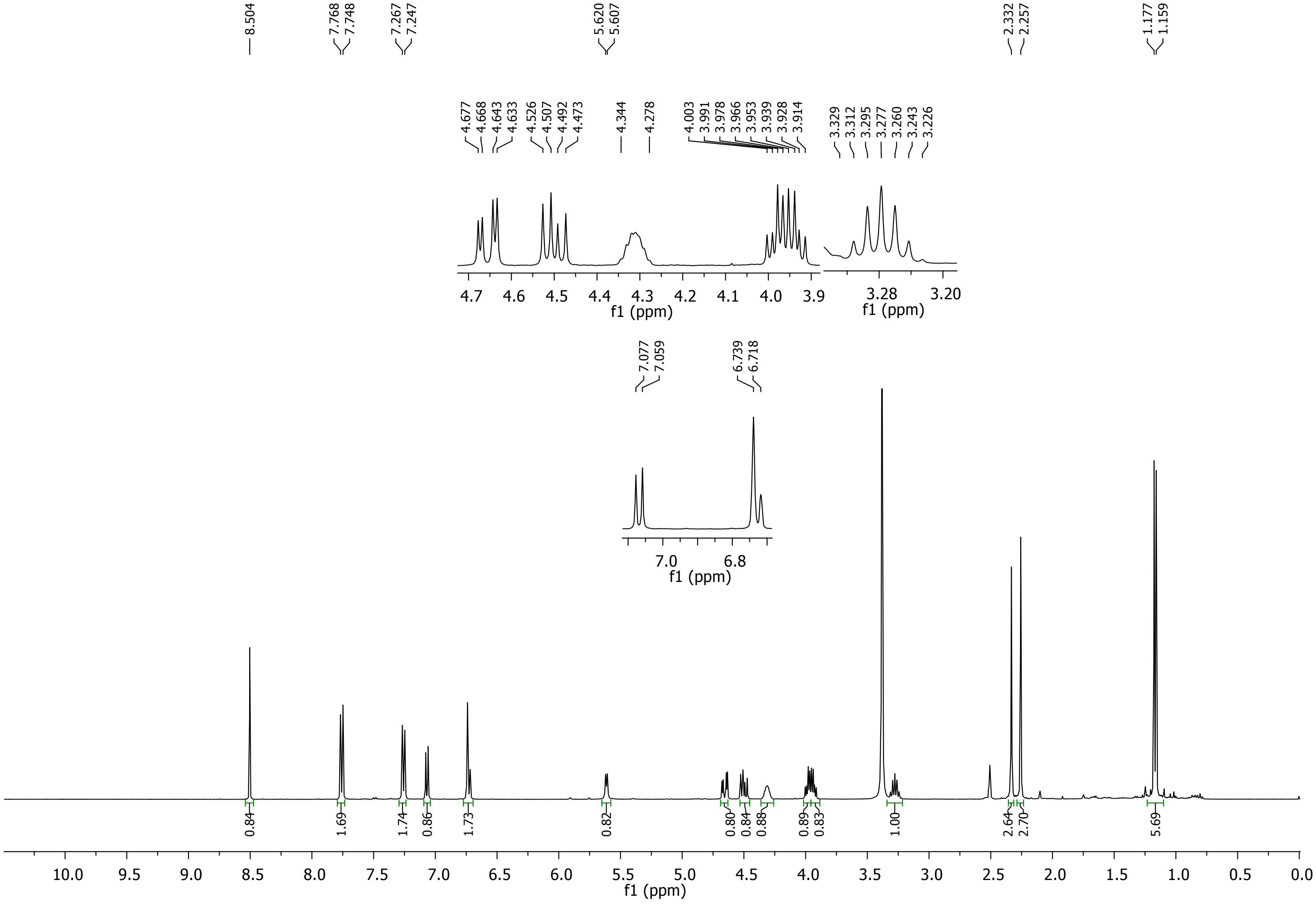

Figure S38. ^{1}H NMR spectrum (400 MHz, DMSO-d_6) of 1-(2-isopropyl-5-methylphenoxy)-3-(4-(*p*-tolyl)-1*H*-1,2,3-triazol-1-yl) propan-2-ol (**3h**).

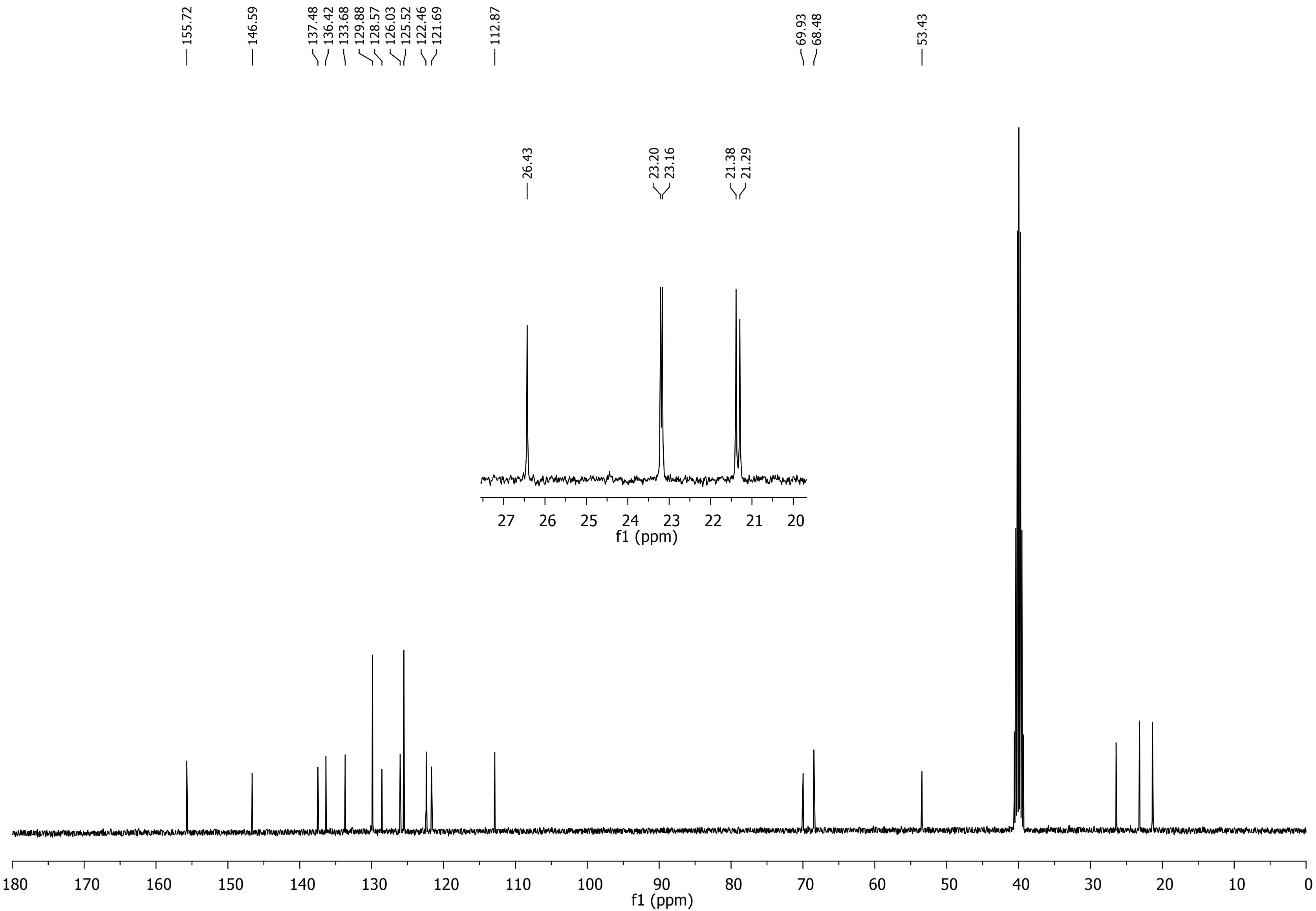

Figure S39. ^{13}C NMR spectrum (100 MHz, DMSO-d_6) of 1-(2-isopropyl-5-methylphenoxy)-3-(4-(*p*-tolyl)-1*H*-1,2,3-triazol-1-yl) propan-2-ol (**3h**).

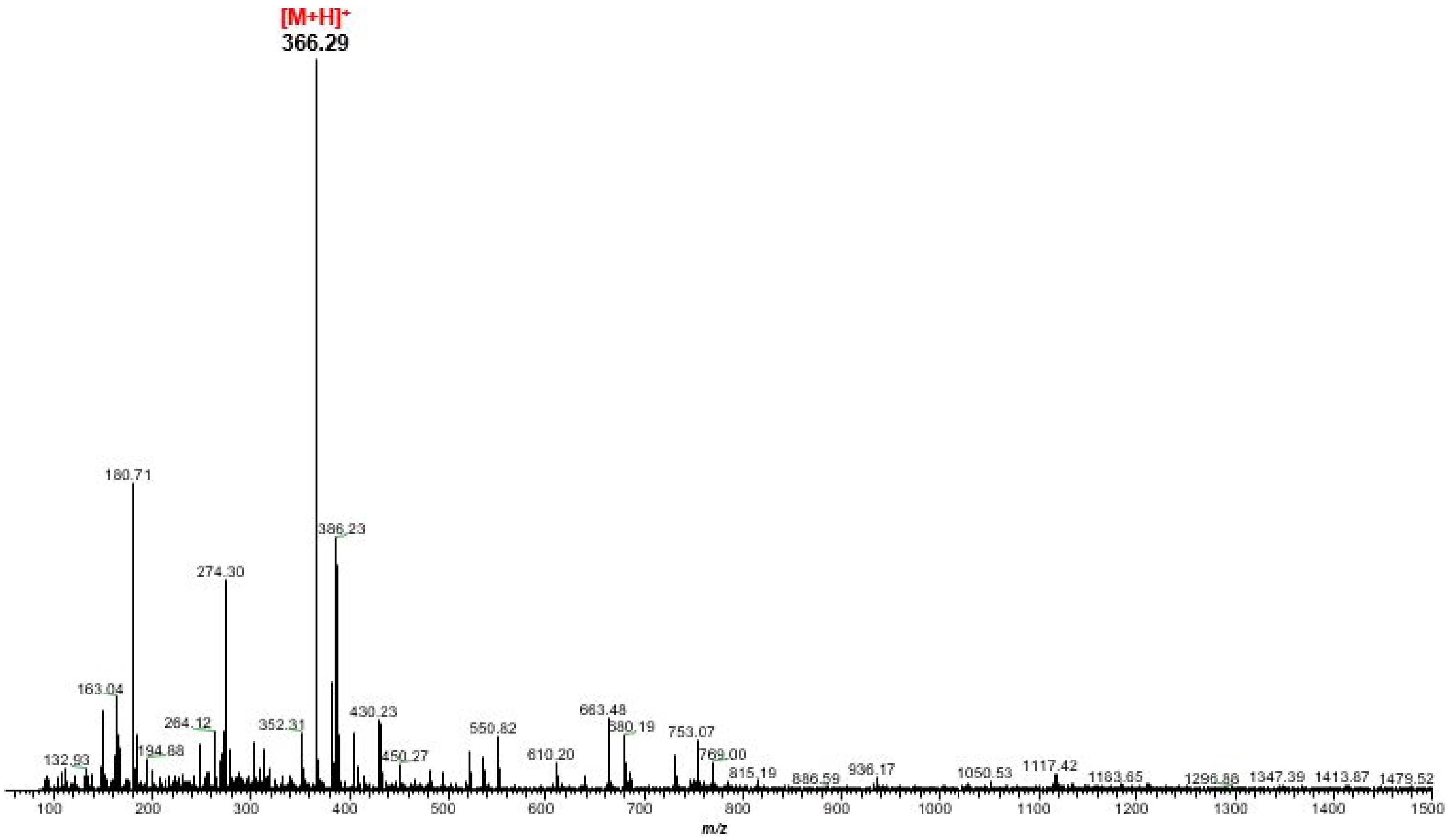

Figure S40. LC-MS spectrum of 1-(2-isopropyl-5-methylphenoxy)-3-(4-(*p*-tolyl)-1*H*-1,2,3-triazol-1-yl) propan-2-ol (**3h**).

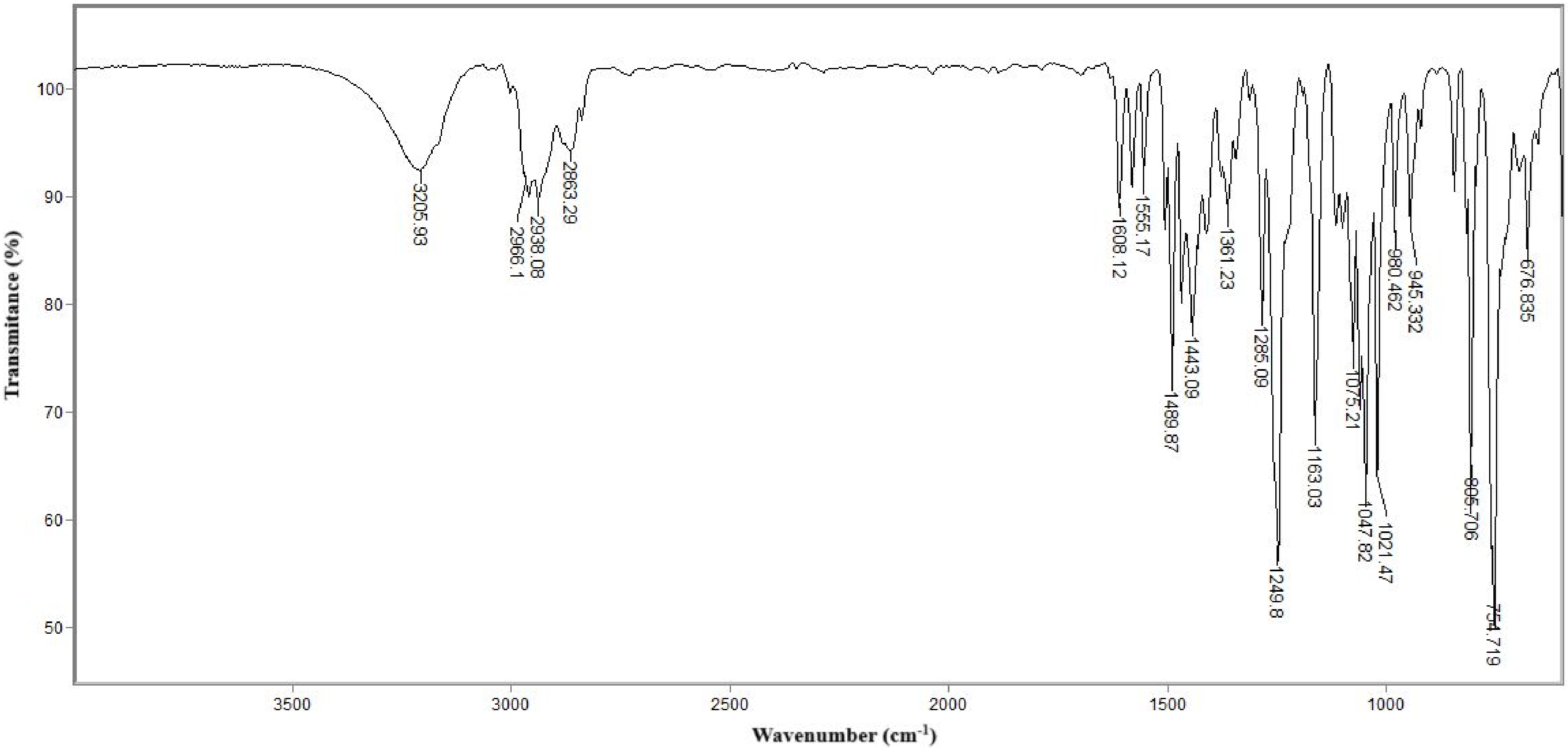

Figure S41. FTIR spectrum (ATR) of 1-(2-isopropyl-5-methylphenoxy)-3-(4-(2-methoxyphenyl)-1*H*-1,2,3-triazol-1-yl) propan-2-ol (**3i**).

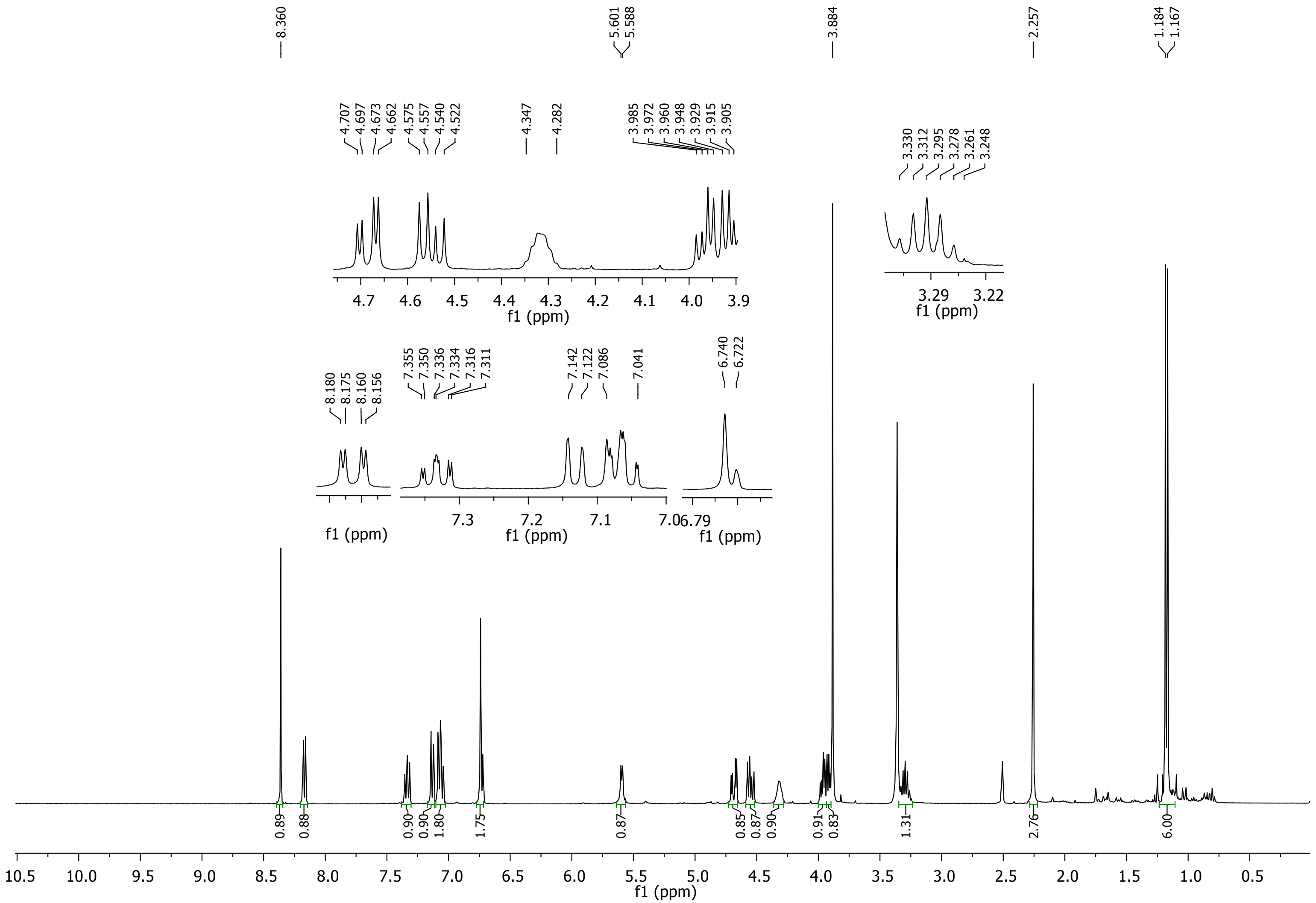

Figure S42. ^{1}H NMR spectrum (400 MHz, DMSO-d_6) of 1-(2-isopropyl-5-methylphenoxy)-3-(4-(2-methoxyphenyl)-1H-1,2,3-triazol-1-yl) propan-2-ol (**3i**).

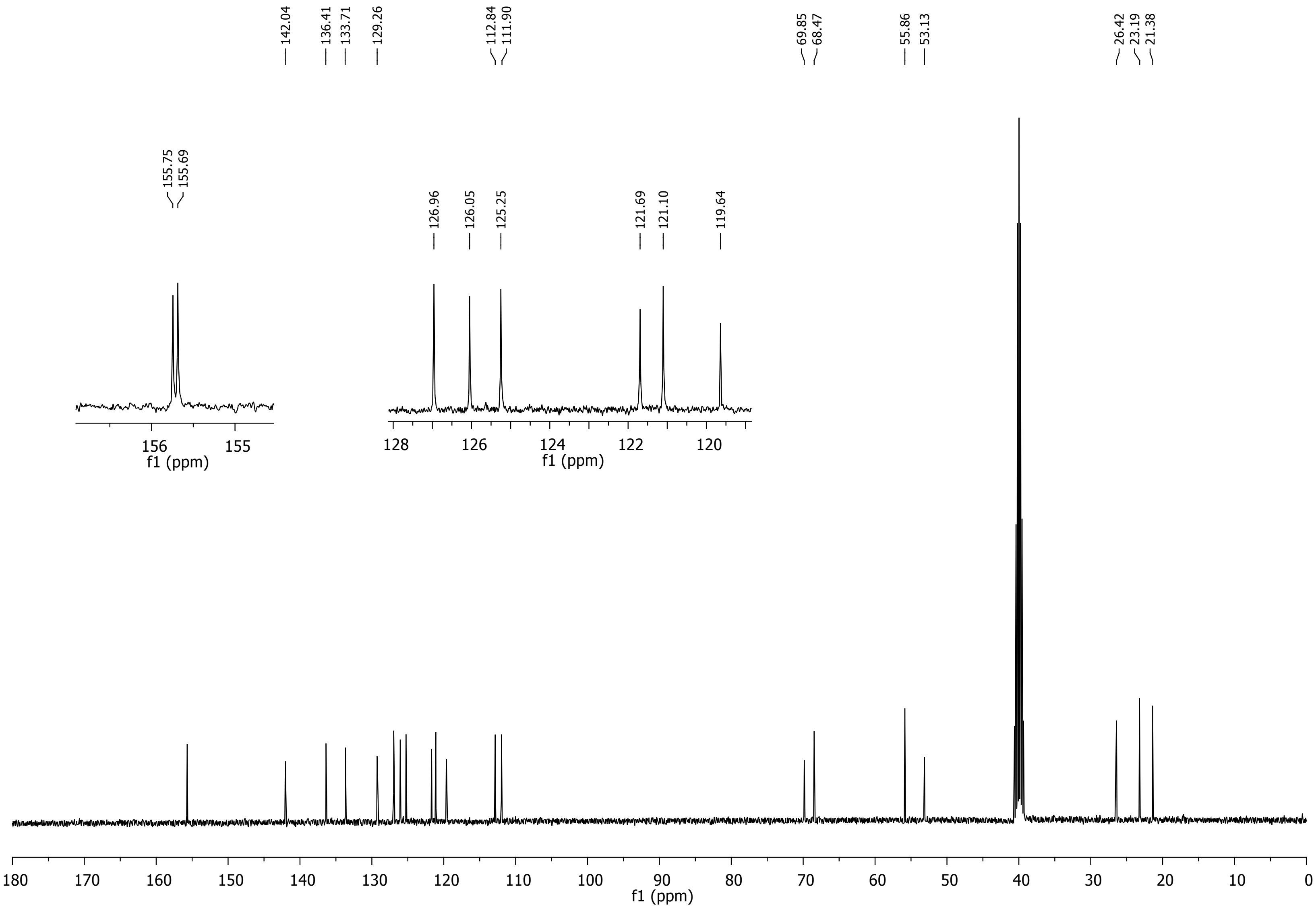

Figure S43. ^{13}C NMR spectrum (100 MHz, DMSO-d_6) of 1-(2-isopropyl-5-methylphenoxy)-3-(4-(2-methoxyphenyl)-1*H*-1,2,3-triazol-1-yl) propan-2-ol (**3i**).

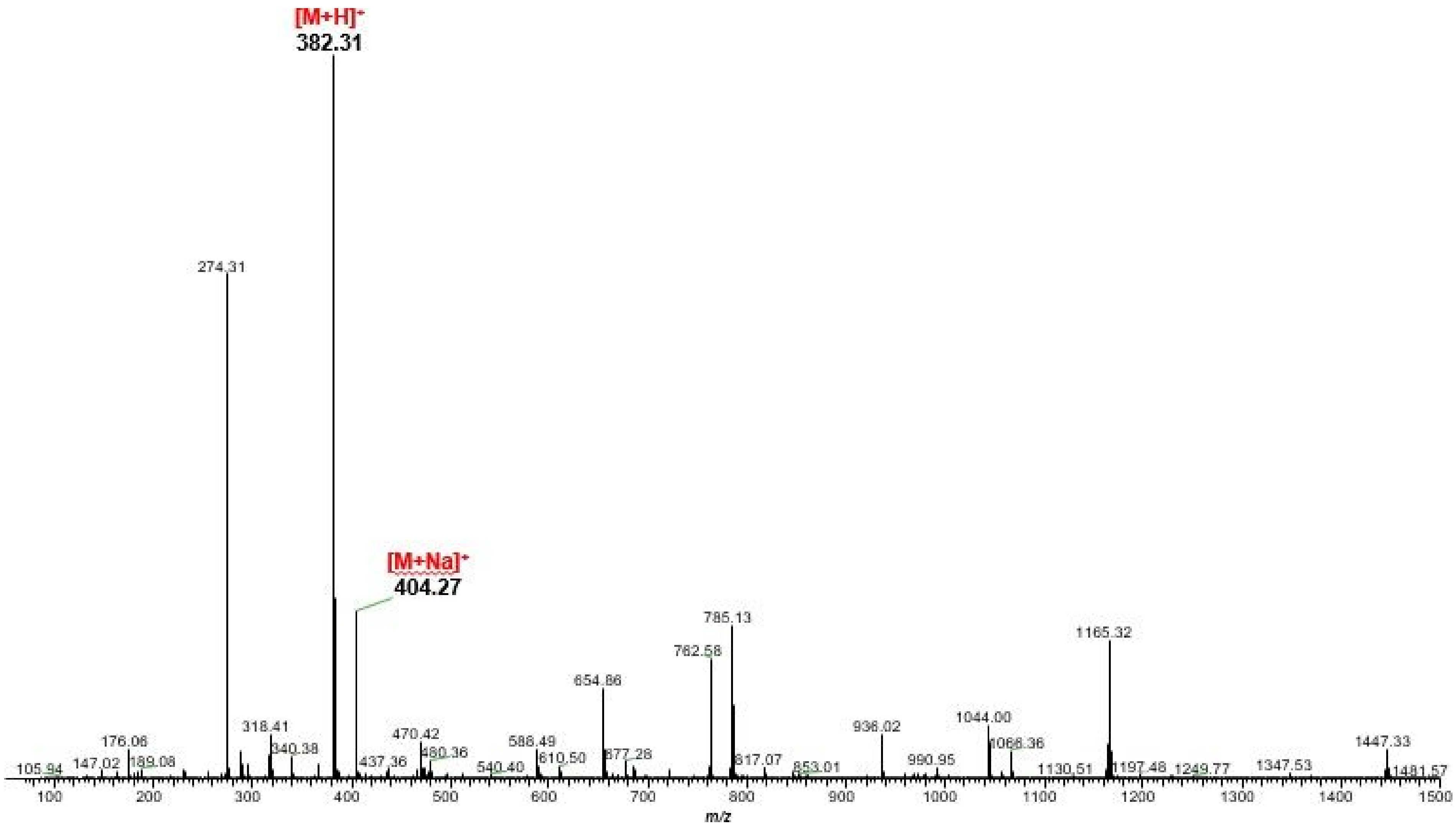

Figure S44. LC-MS spectrum of 1-(2-isopropyl-5-methylphenoxy)-3-(4-(2-methoxyphenyl)-1*H*-1,2,3-triazol-1-yl) propan-2-ol (**3i**).

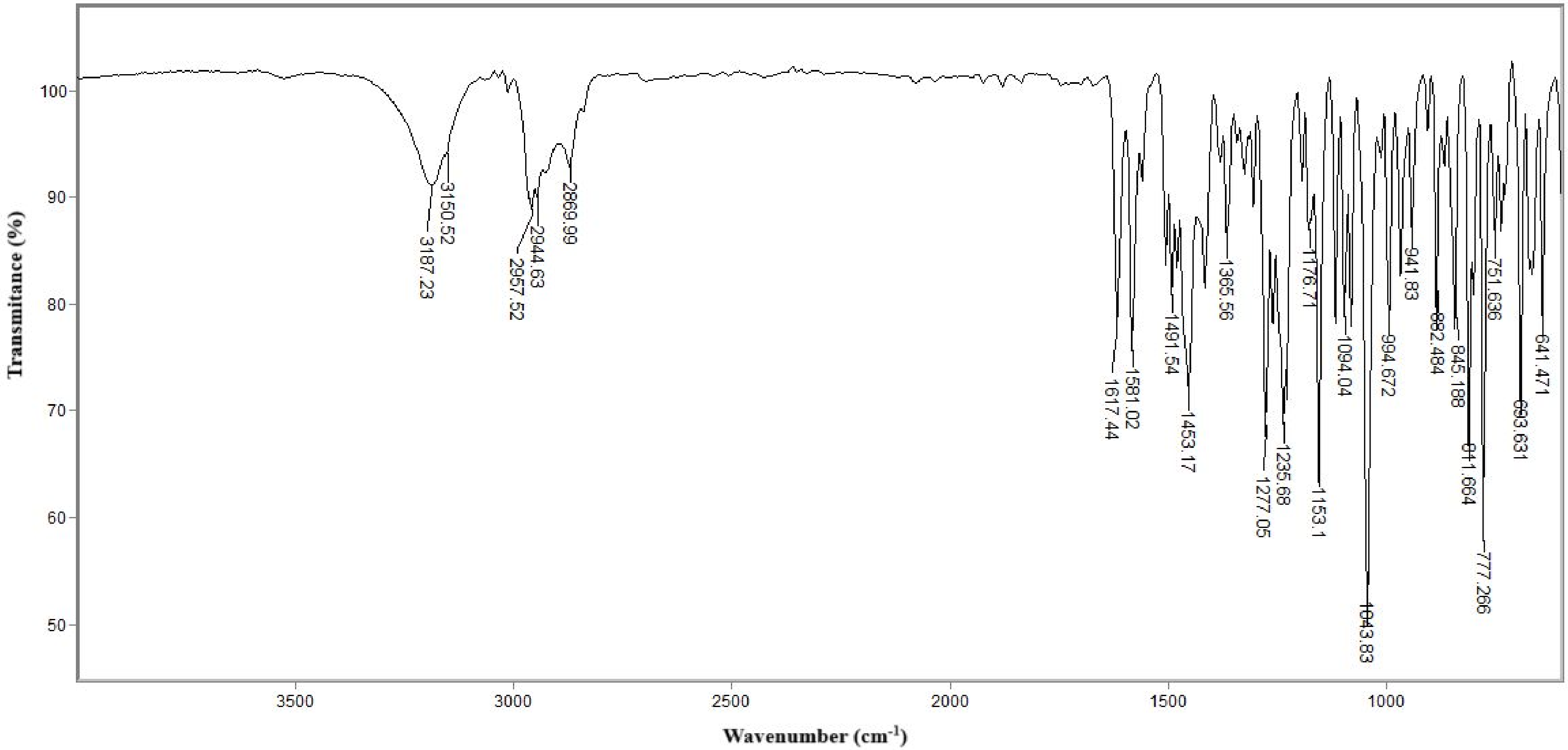

Figure S45. FTIR spectrum (ATR) of 1-(2-isopropyl-5-methylphenoxy)-3-(4-(3-methoxyphenyl)-1*H*-1,2,3-triazol-1-yl) propan-2-ol (**3j**).

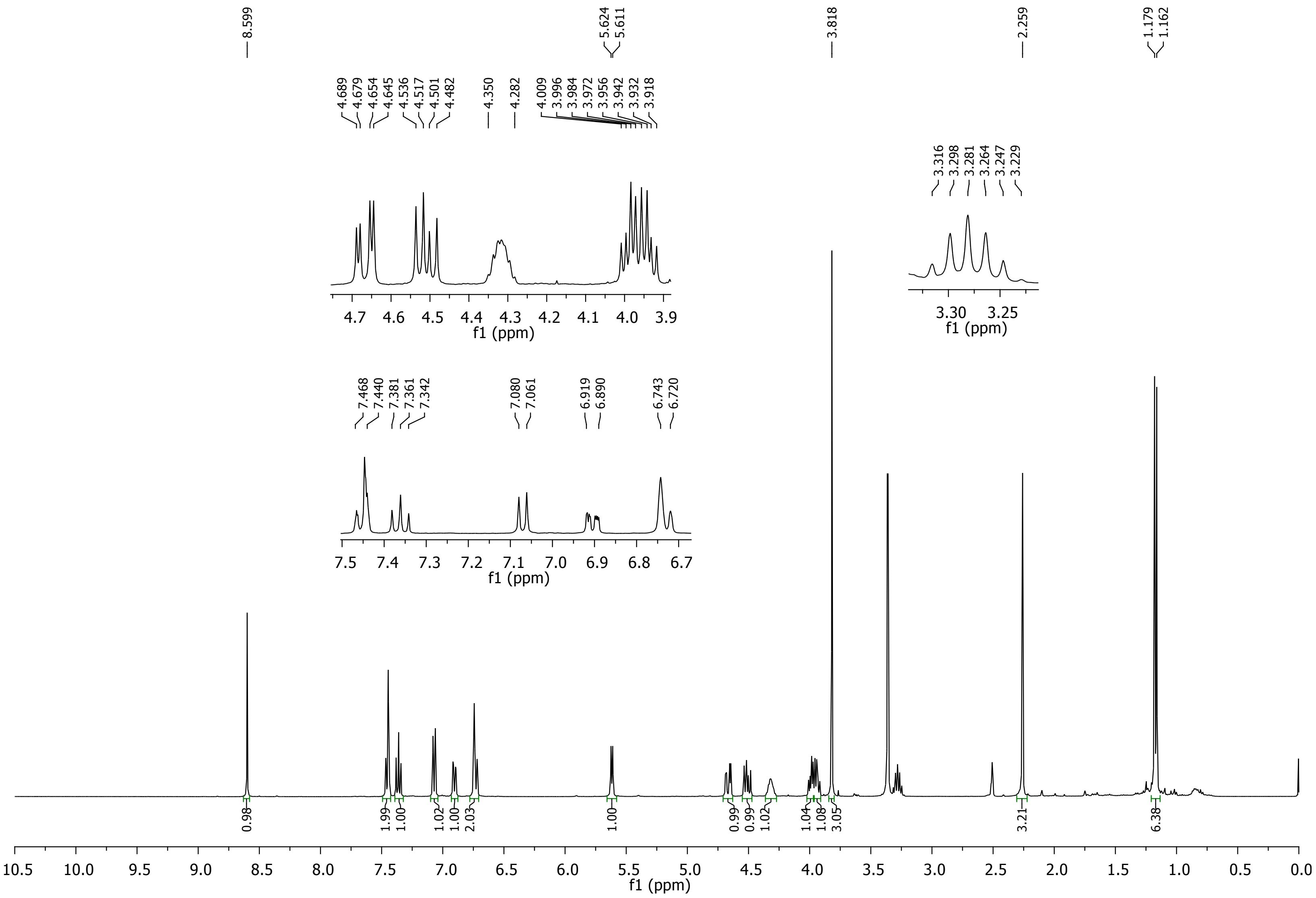

Figure S46. ^{1}H NMR spectrum (400 MHz, DMSO-d_6) of 1-(2-isopropyl-5-methylphenoxy)-3-(4-(3-methoxyphenyl)-1*H*-1,2,3-triazol-1-yl) propan-2-ol (**3j**).

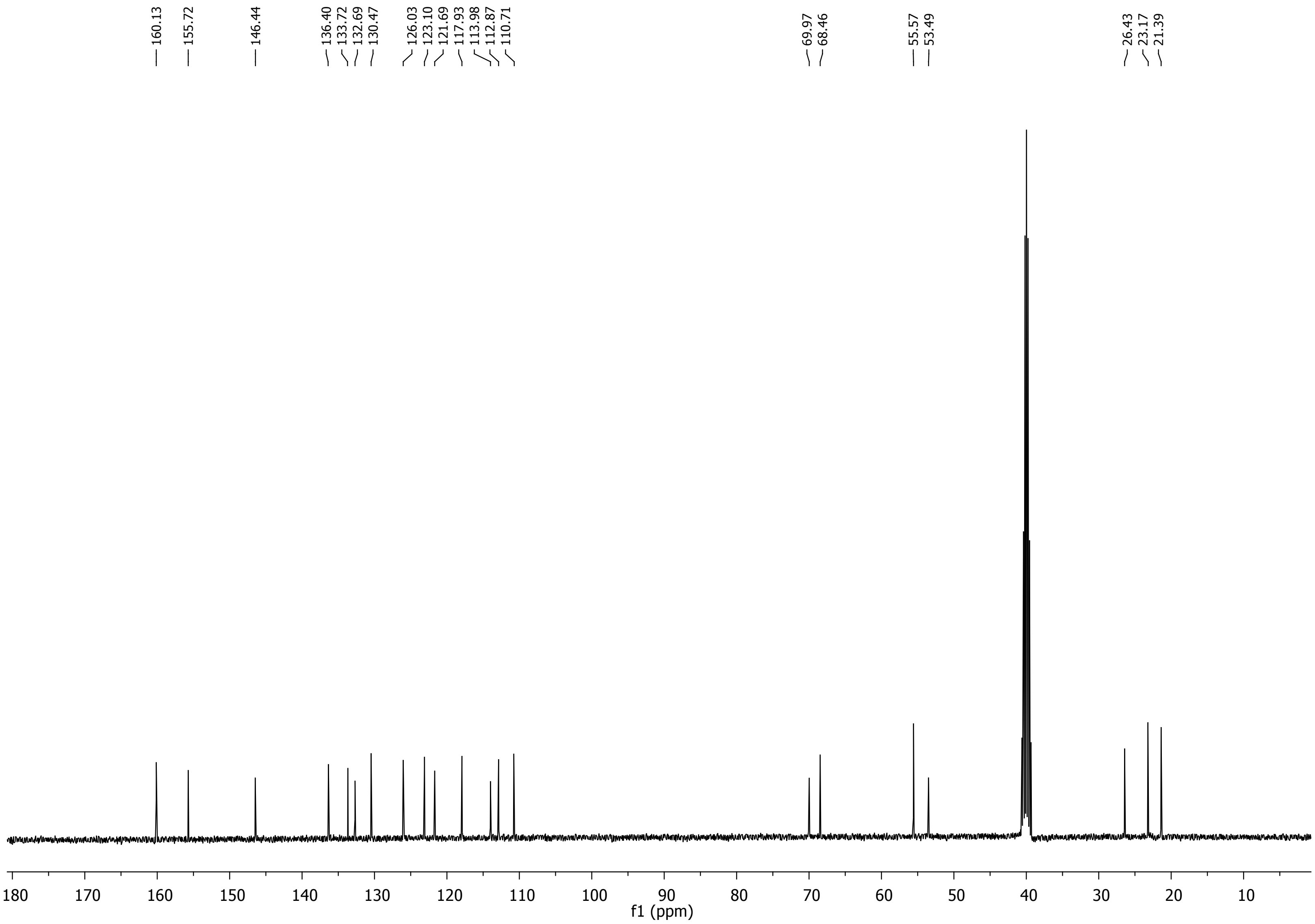

Figure S47. ^{13}C NMR spectrum (100 MHz, DMSO-d_6) of 1-(2-isopropyl-5-methylphenoxy)-3-(4-(3-methoxyphenyl)-1*H*-1,2,3-triazol-1-yl) propan-2-ol (**3j**).

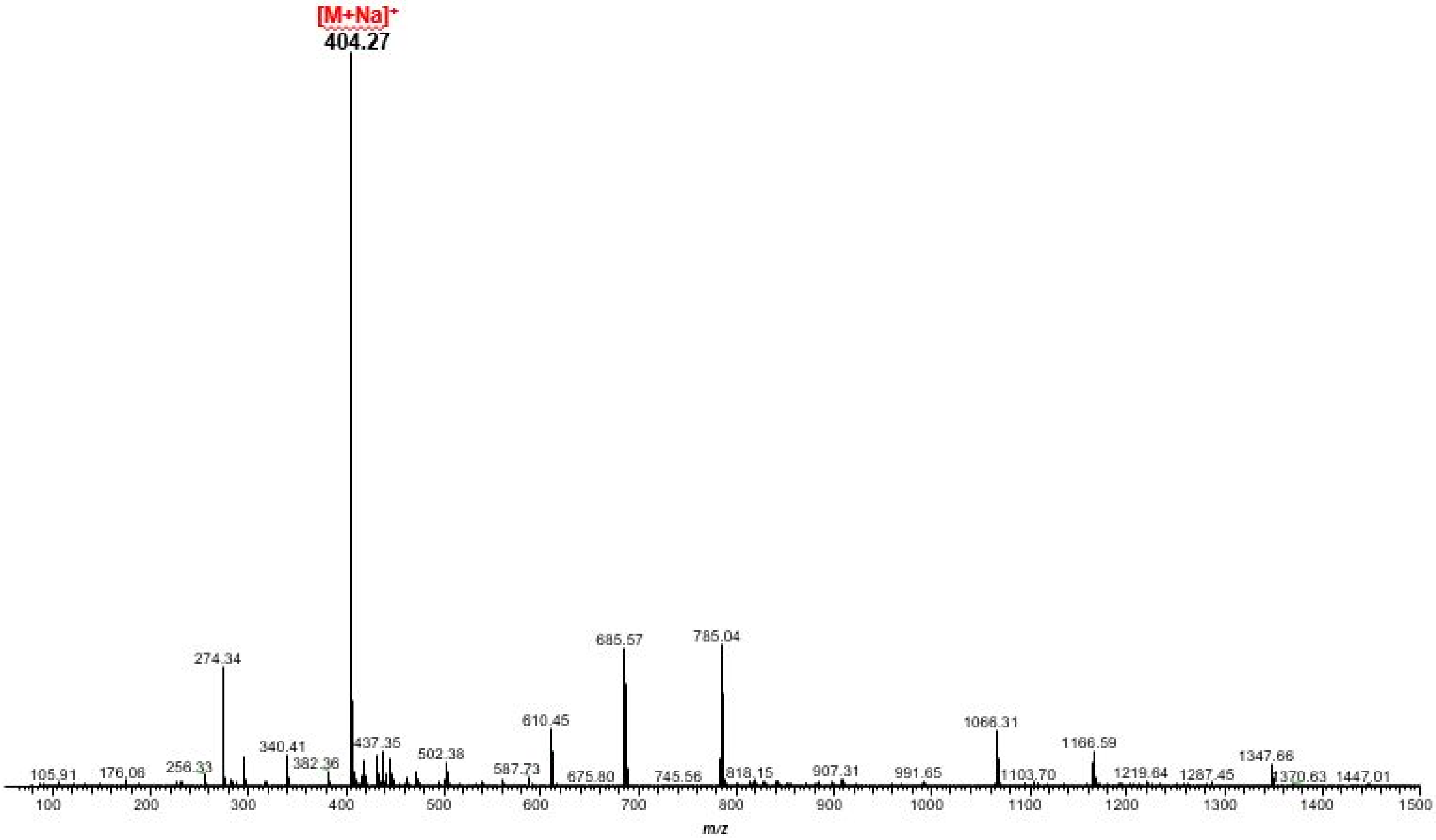

Figure S48. LC-MS spectrum of 1-(2-isopropyl-5-methylphenoxy)-3-(4-(3-methoxyphenyl)-1*H*-1,2,3-triazol-1-yl) propan-2-ol (**3j**).

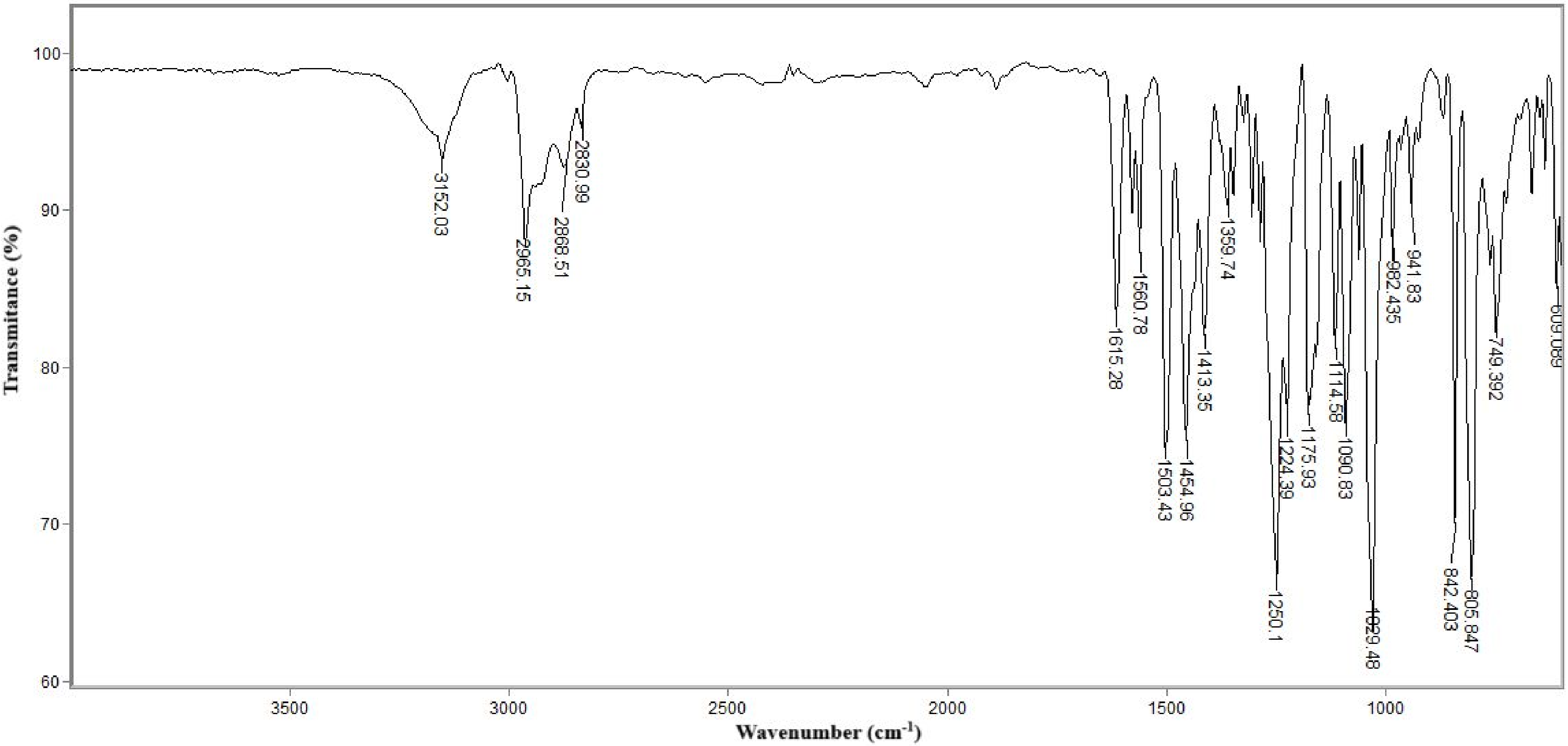

Figure S49. FTIR spectrum (ATR) of 1-(2-isopropyl-5-methylphenoxy)-3-(4-(4-methoxyphenyl)-1*H*-1,2,3-triazol-1-yl) propan-2-ol (**3k**).

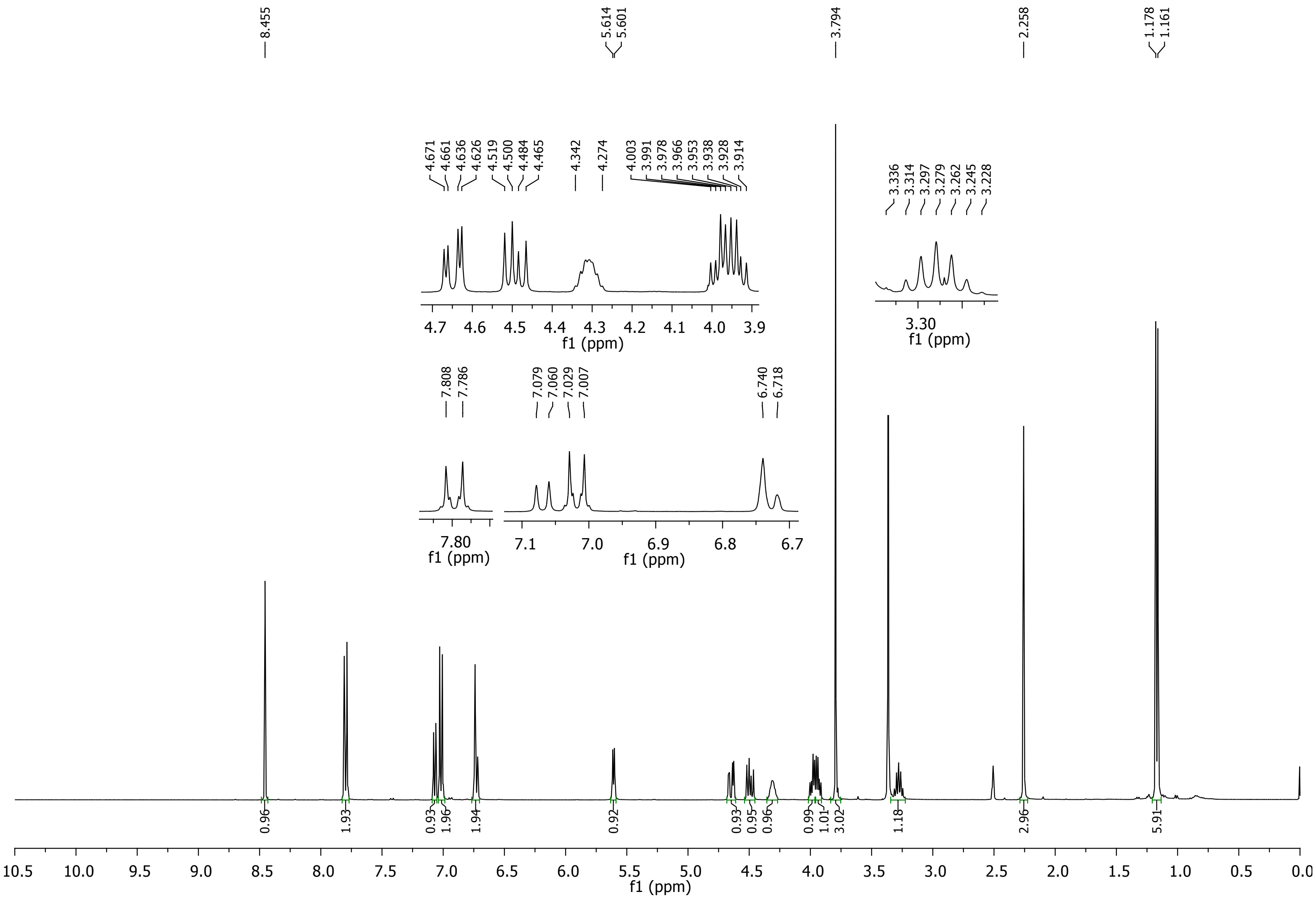

Figure S50. ^{1}H NMR spectrum (400 MHz, DMSO-d_6) of 1-(2-isopropyl-5-methylphenoxy)-3-(4-(4-methoxyphenyl)-1*H*-1,2,3-triazol-1-yl) propan-2-ol (**3k**).

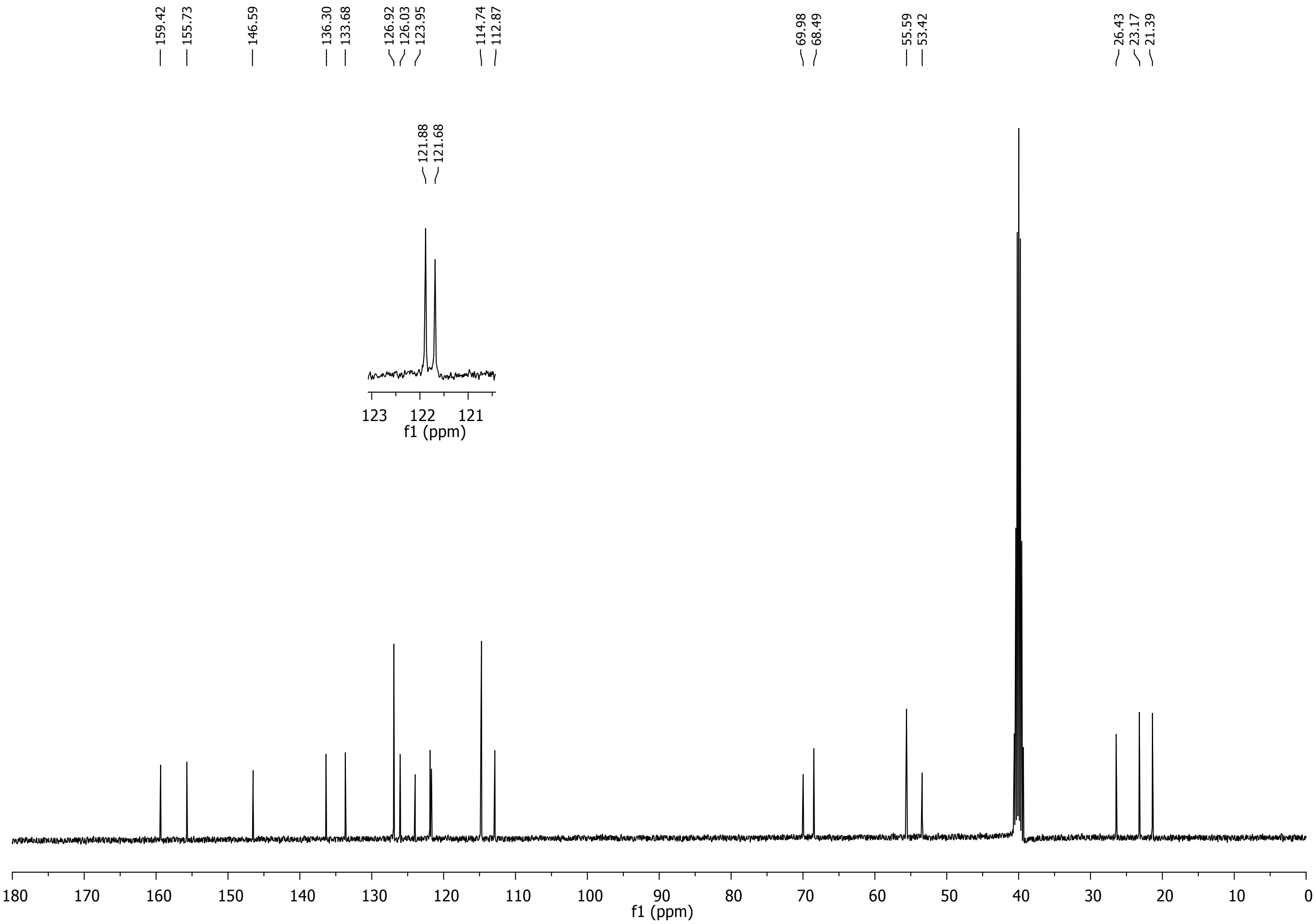

Figure S51. ^{13}C NMR spectrum (100 MHz, DMSO-d_6) of 1-(2-isopropyl-5-methylphenoxy)-3-(4-(4-methoxyphenyl)-1*H*-1,2,3-triazol-1-yl) propan-2-ol (**3k**).

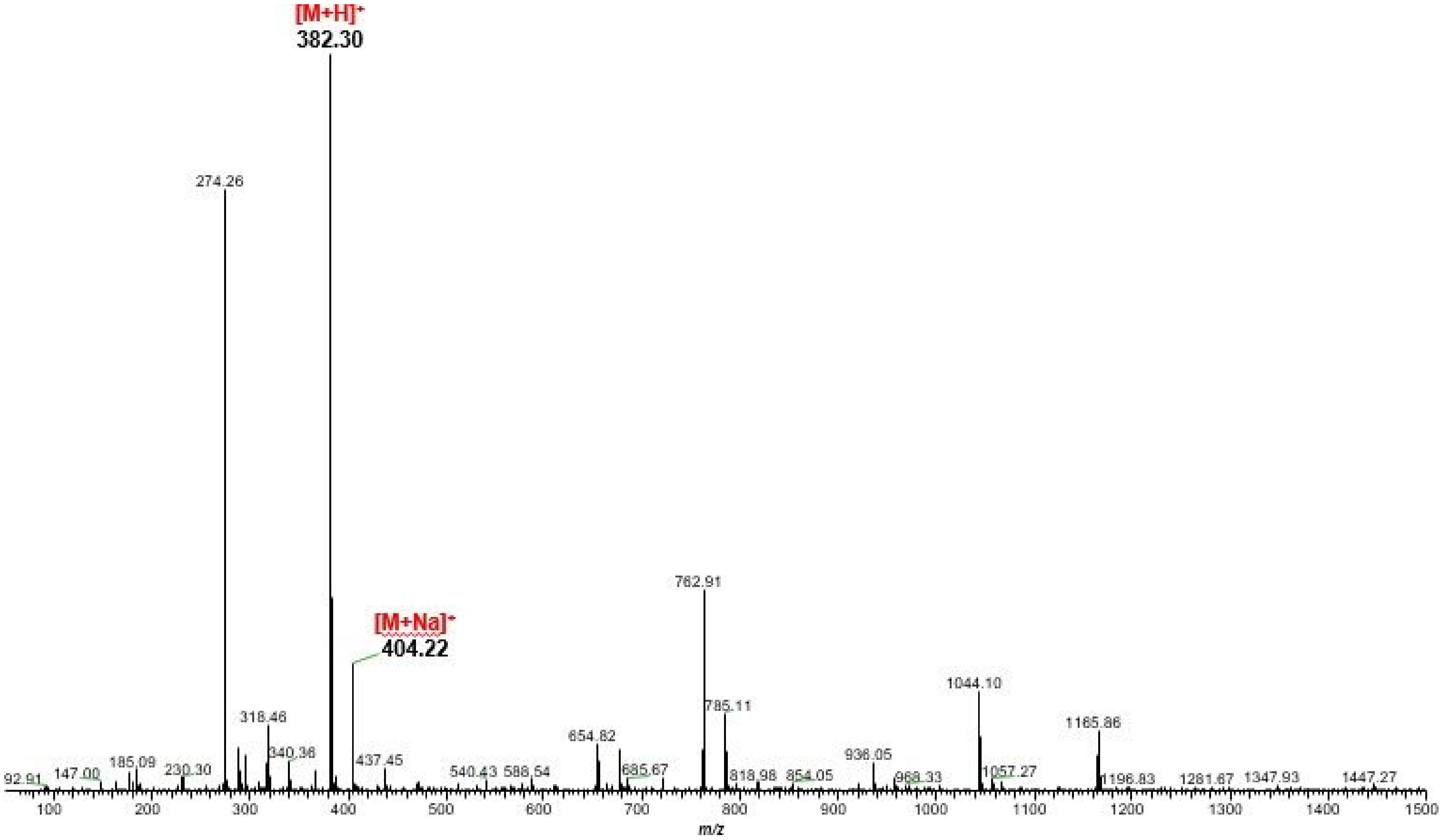

Figure S52. LC-MS spectrum of 1-(2-isopropyl-5-methylphenoxy)-3-(4-(4-methoxyphenyl)-1*H*-1,2,3-triazol-1-yl) propan-2-ol (**3k**).

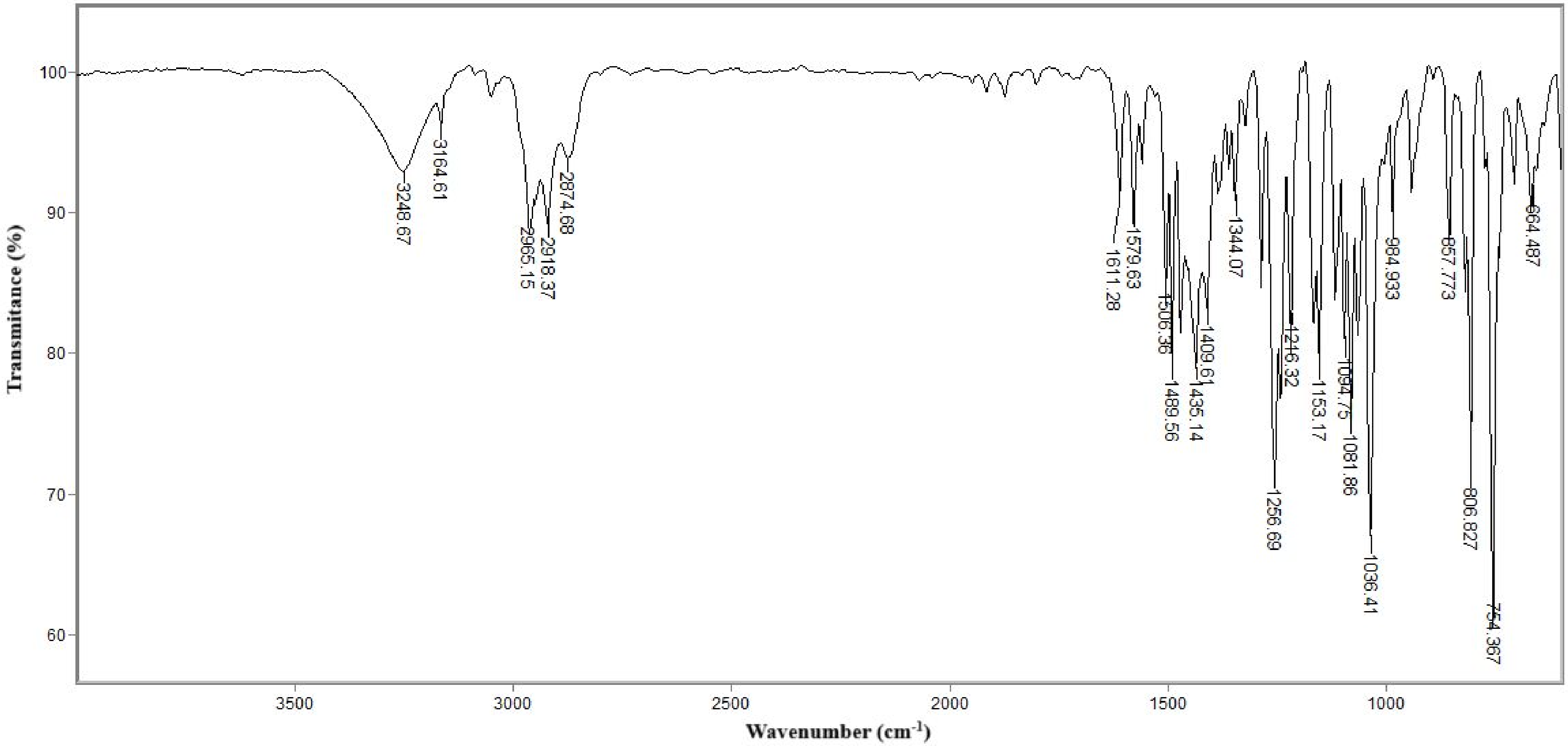

Figure S53. FTIR spectrum (ATR) of 1-(4-(2-fluorophenyl)-1*H*-1,2,3-triazol-1-yl)-3-(2-isopropyl-5-methylphenoxy) propan-2-ol (**3l**).

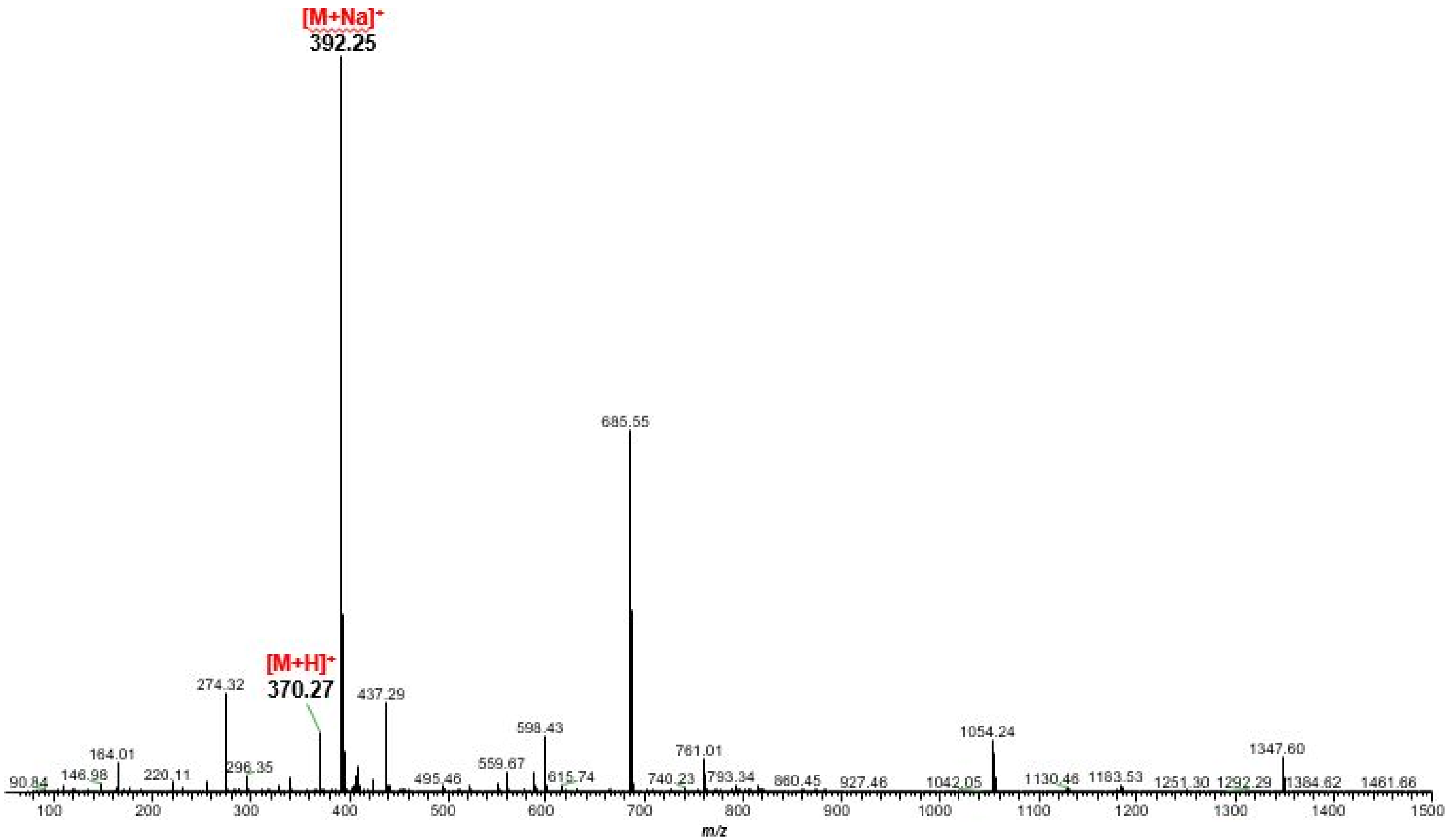

Figure S54. LC-MS spectrum of 1-(4-(2-fluorophenyl)-1*H*-1,2,3-triazol-1-yl)-3-(2-isopropyl-5-methylphenoxy) propan-2-ol (**3l**).

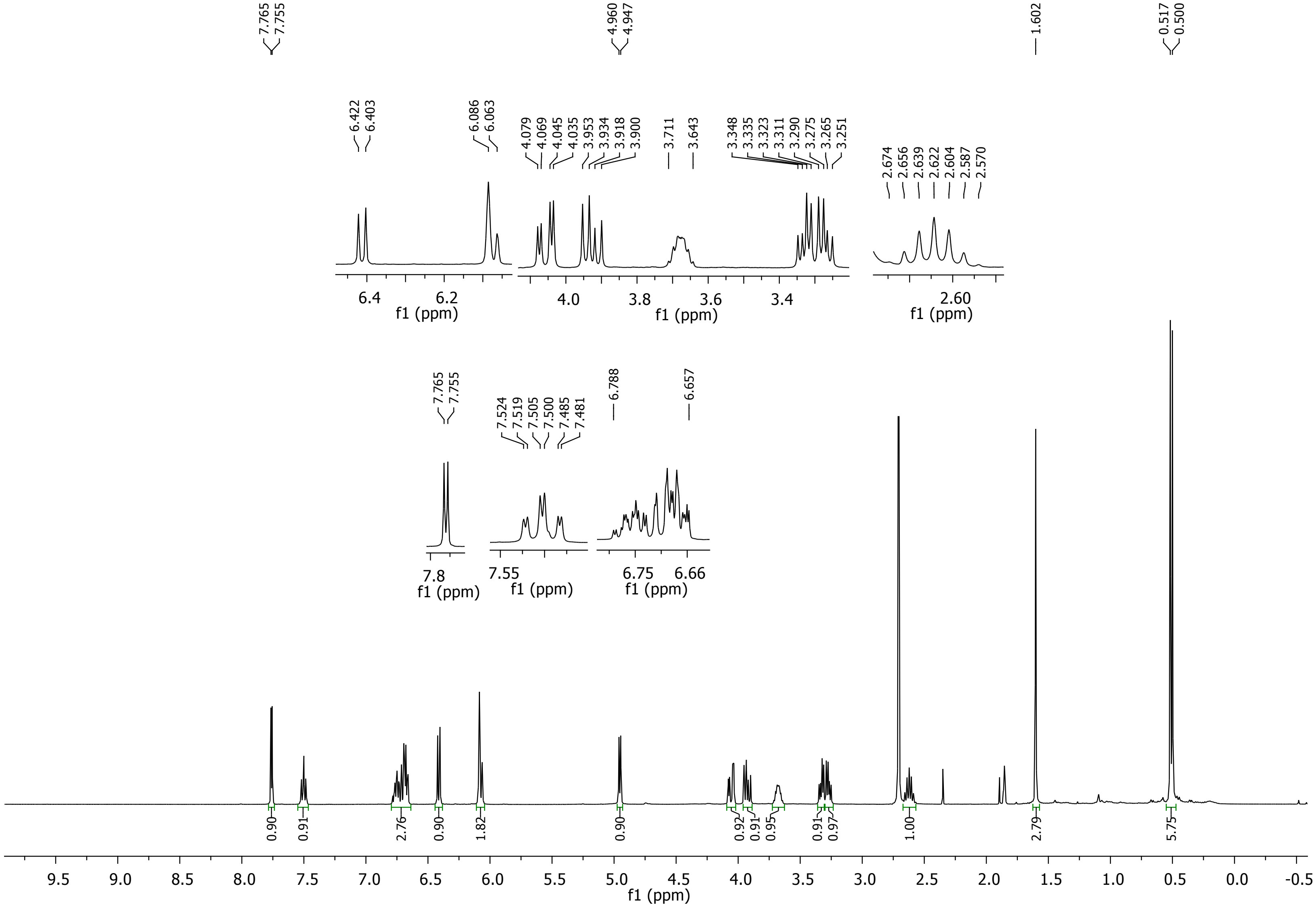

Figure S55. ^{1}H NMR spectrum (400 MHz, DMSO-d_6) of 1-(4-(2-fluorophenyl)-1*H*-1,2,3-triazol-1-yl)-3-(2-isopropyl-5-methylphenoxy) propan-2-ol (**3l**).

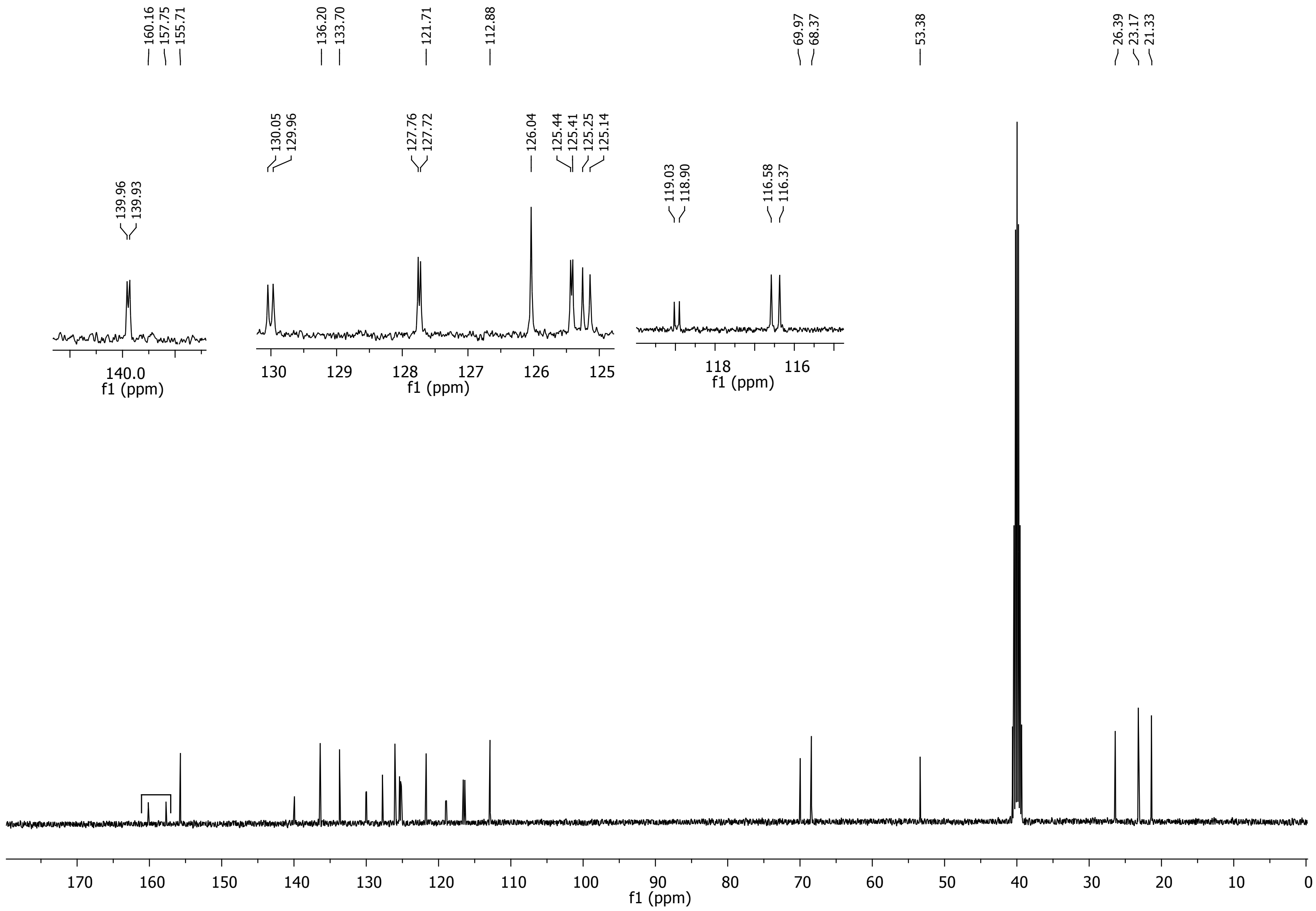

Figure S56. ^{13}C NMR spectrum (100 MHz, DMSO-d_6) of 1-(4-(2-fluorophenyl)-1*H*-1,2,3-triazol-1-yl)-3-(2-isopropyl-5-methylphenoxy) propan-2-ol (**3l**).

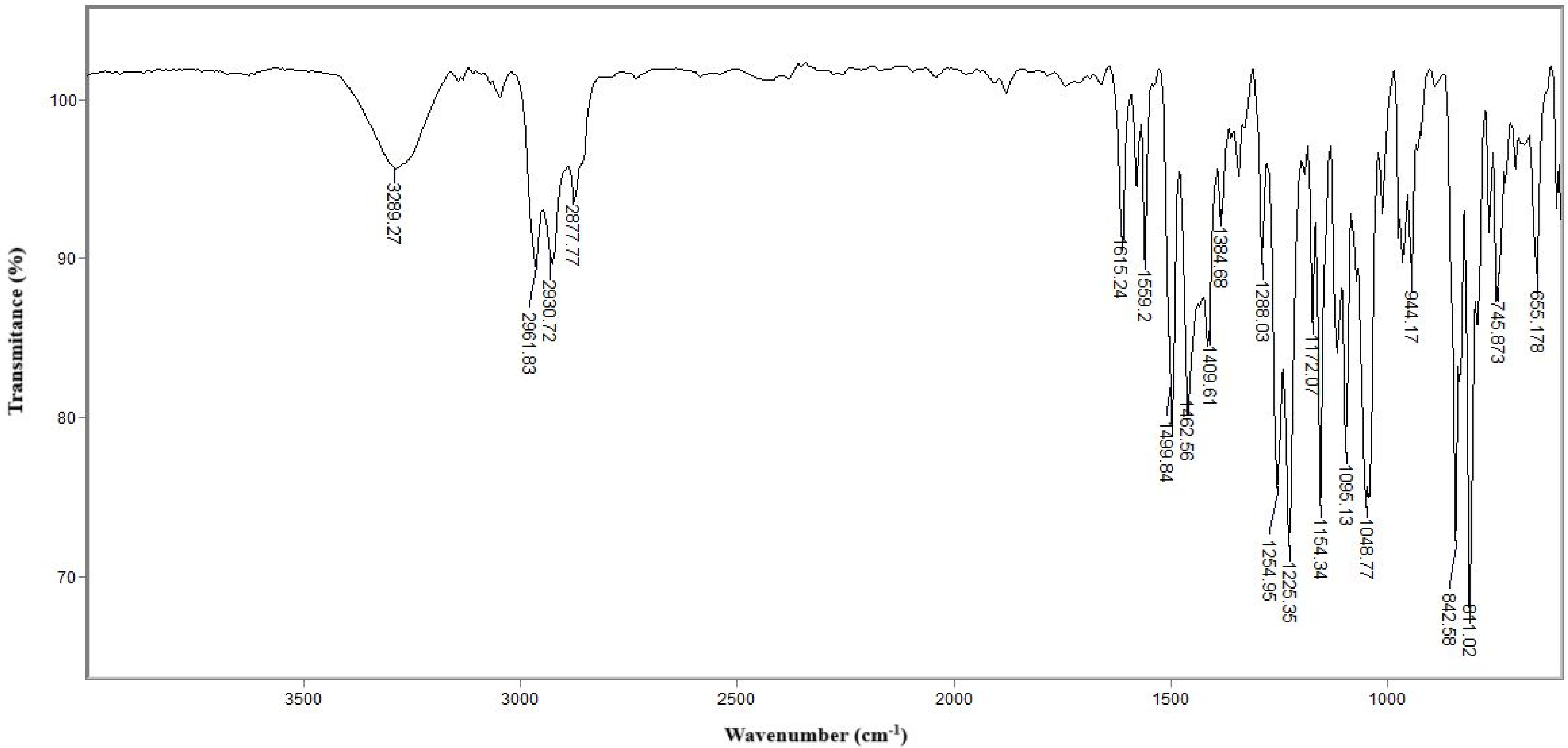

Figure S57. FTIR spectrum (ATR) of 1-(4-(4-fluorophenyl)-1*H*-1,2,3-triazol-1-yl)-3-(2-isopropyl-5-methylphenoxy) propan-2-ol (**3m**).

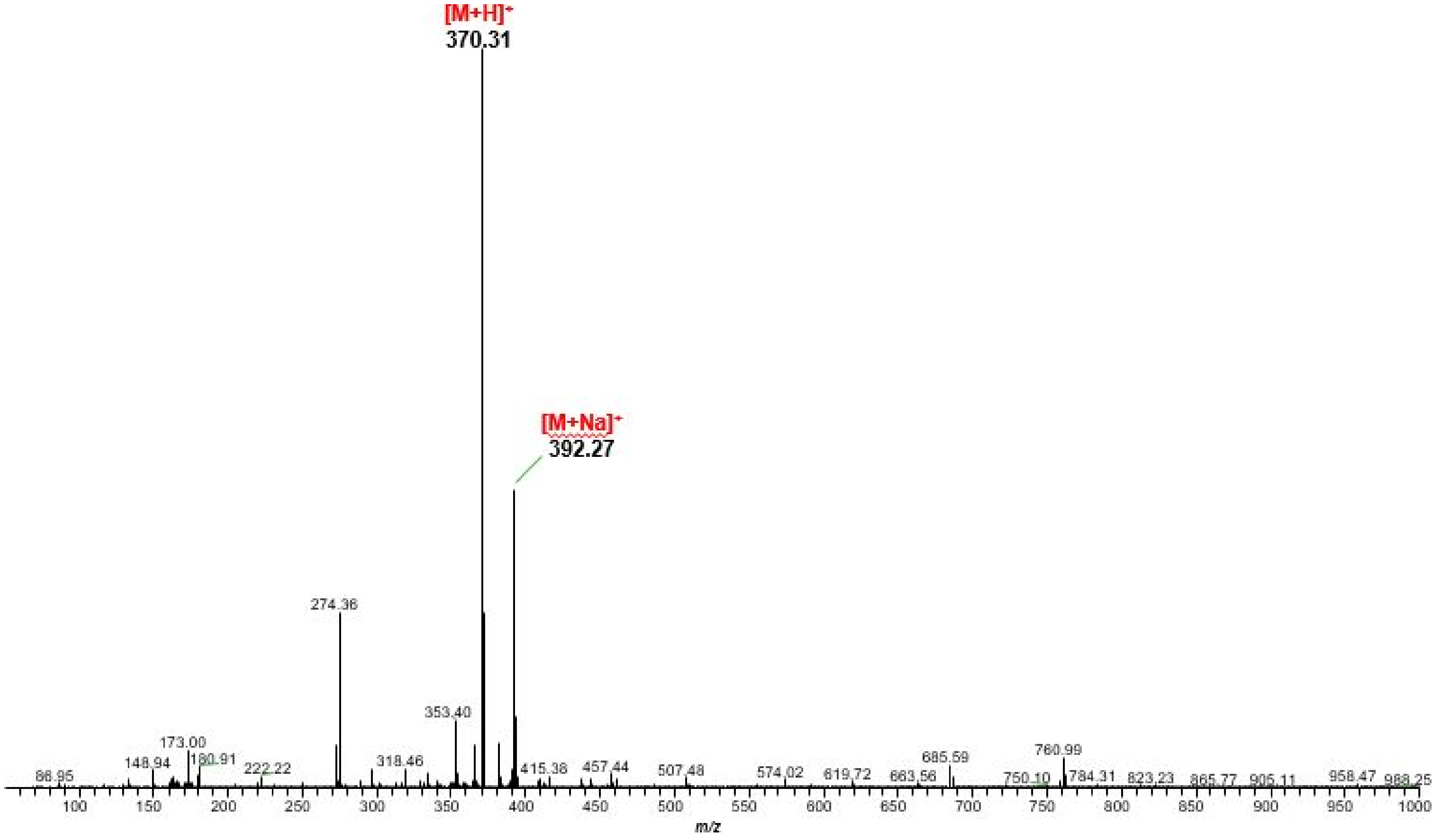

Figure S58. LC-MS spectrum of 1-(4-(4-fluorophenyl)-1*H*-1,2,3-triazol-1-yl)-3-(2-isopropyl-5-methylphenoxy) propan-2-ol (**3m**).

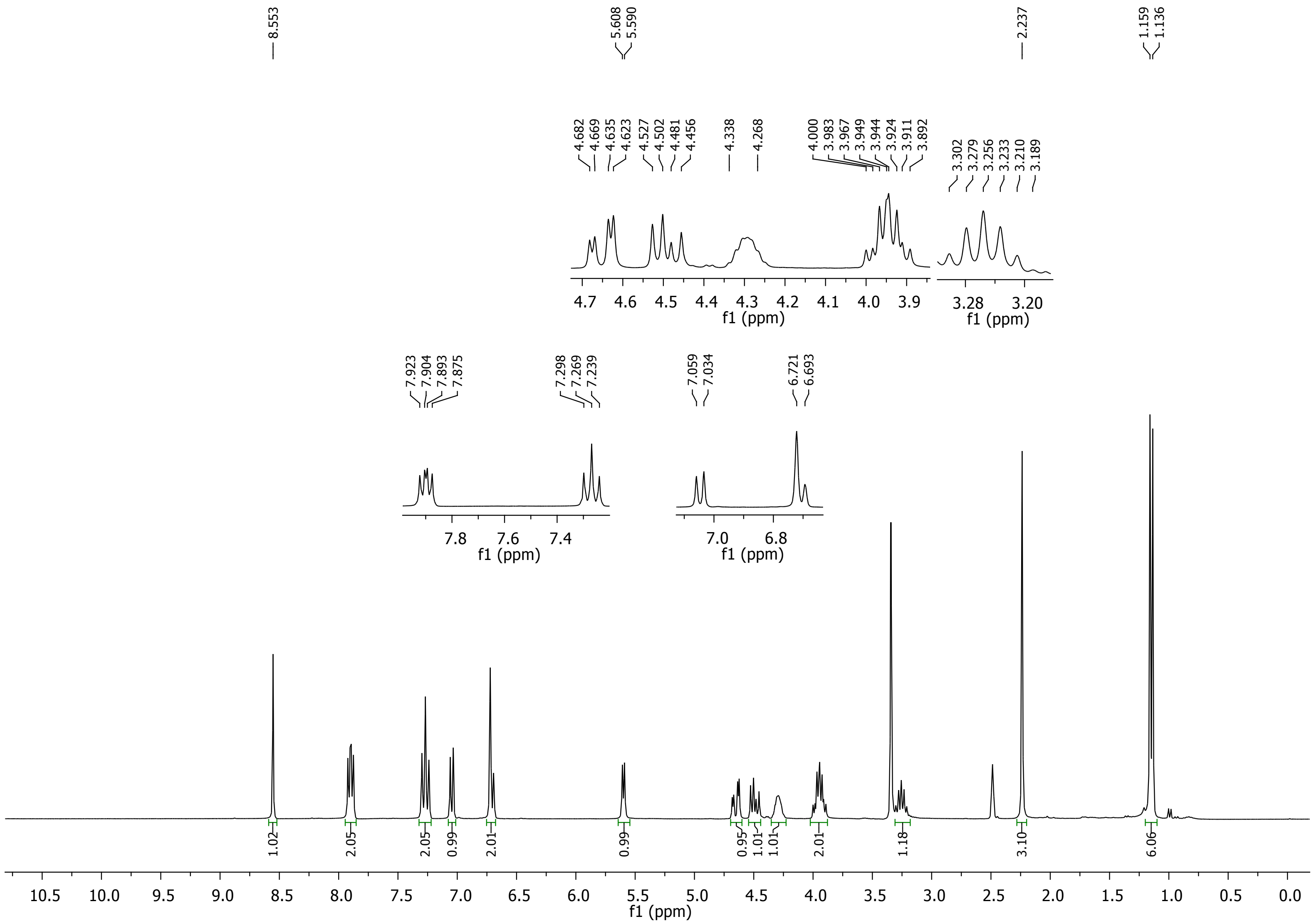

Figure S59. ^{1}H NMR spectrum (300 MHz, DMSO-d_6) of 1-(4-(4-fluorophenyl)-1*H*-1,2,3-triazol-1-yl)-3-(2-isopropyl-5-methylphenoxy) propan-2-ol (**3m**).

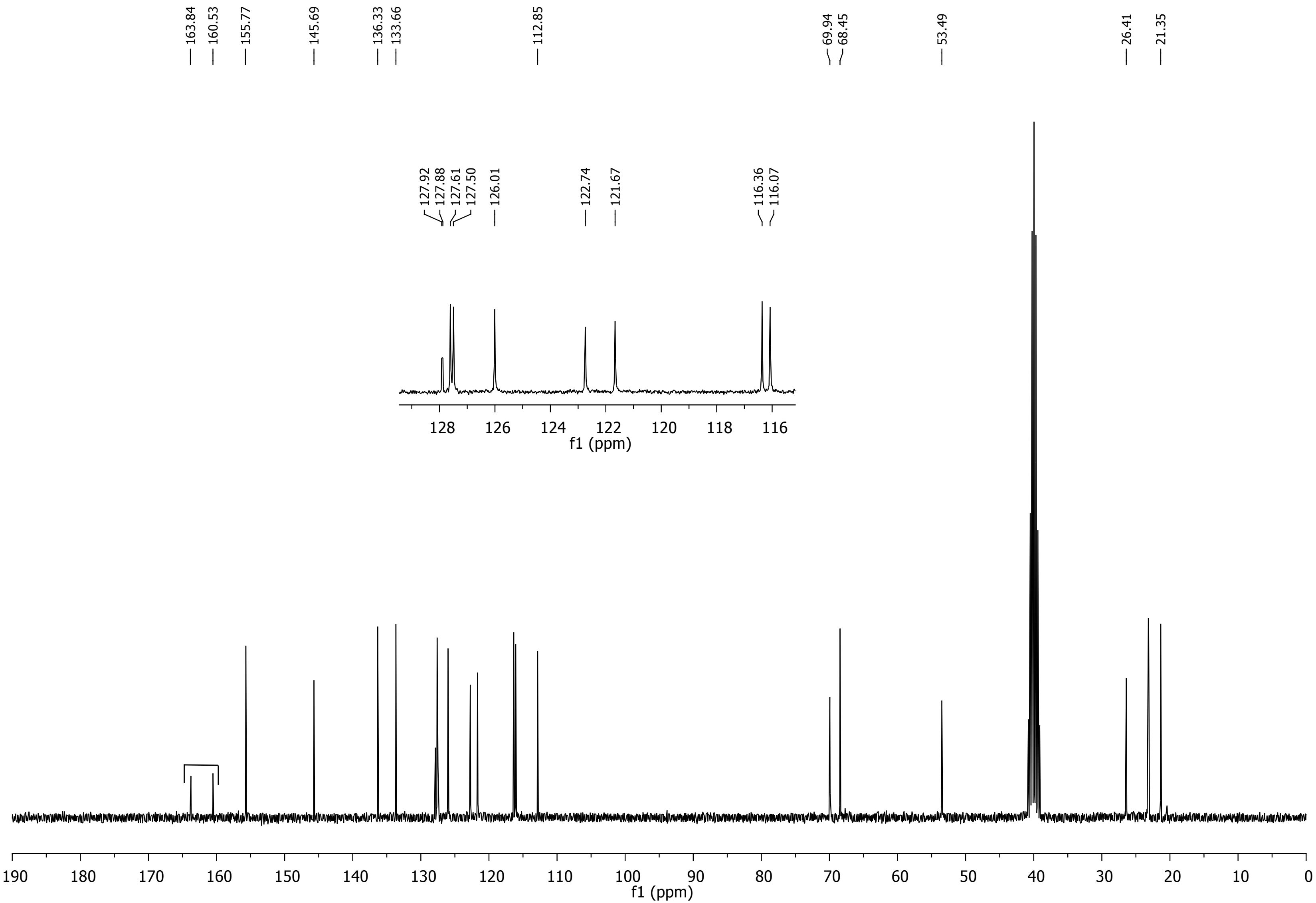

Figure S60. ^{13}C NMR spectrum (75 MHz, DMSO-d_6) of 1-(4-(4-fluorophenyl)-1*H*-1,2,3-triazol-1-yl)-3-(2-isopropyl-5-methylphenoxy) propan-2-ol (**3m**).

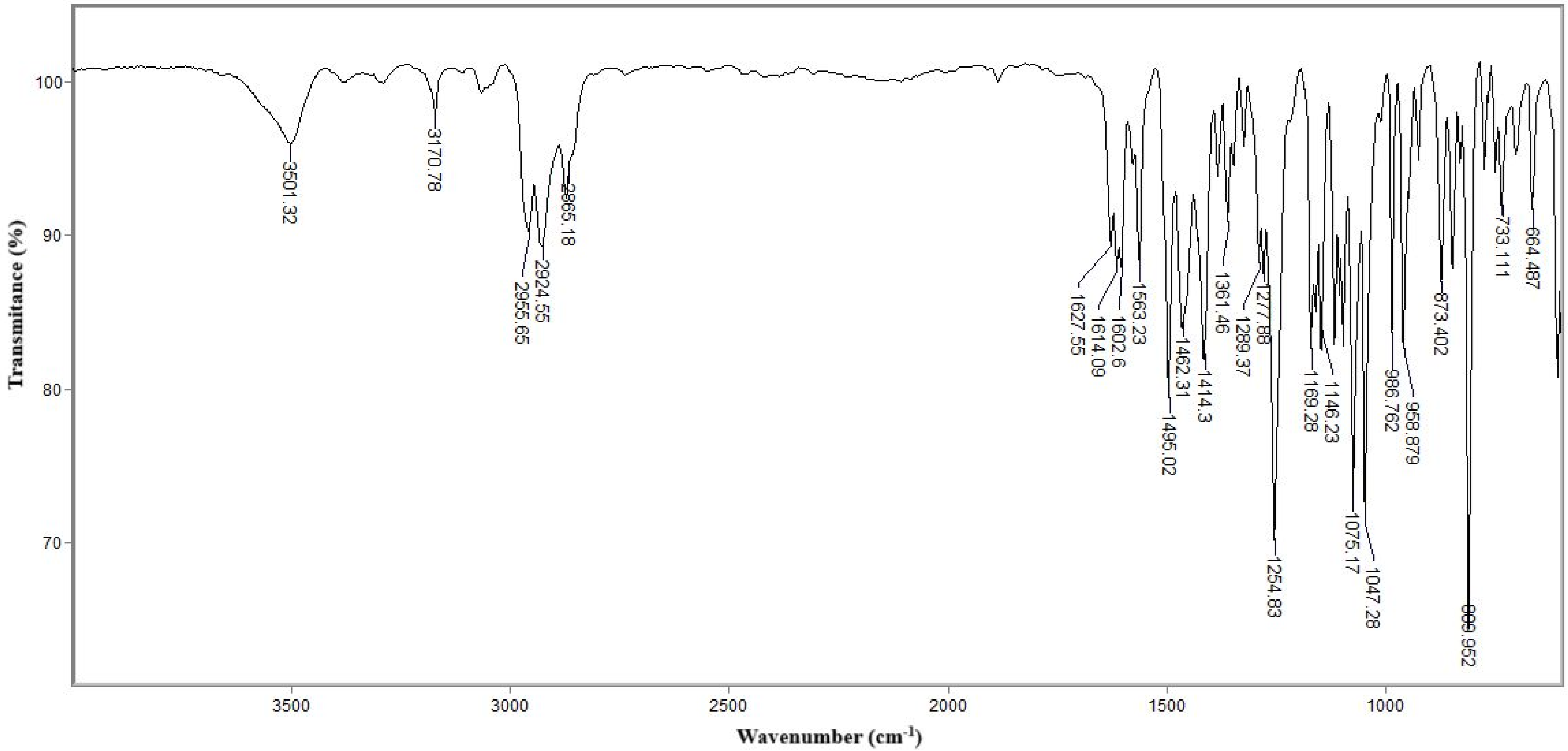

Figure S61. FTIR spectrum (ATR) of 1-(4-(2,4-difluorophenyl)-1*H*-1,2,3-triazol-1-yl)-3-(2-isopropyl-5-methylphenoxy) propan-2-ol (**3n**).

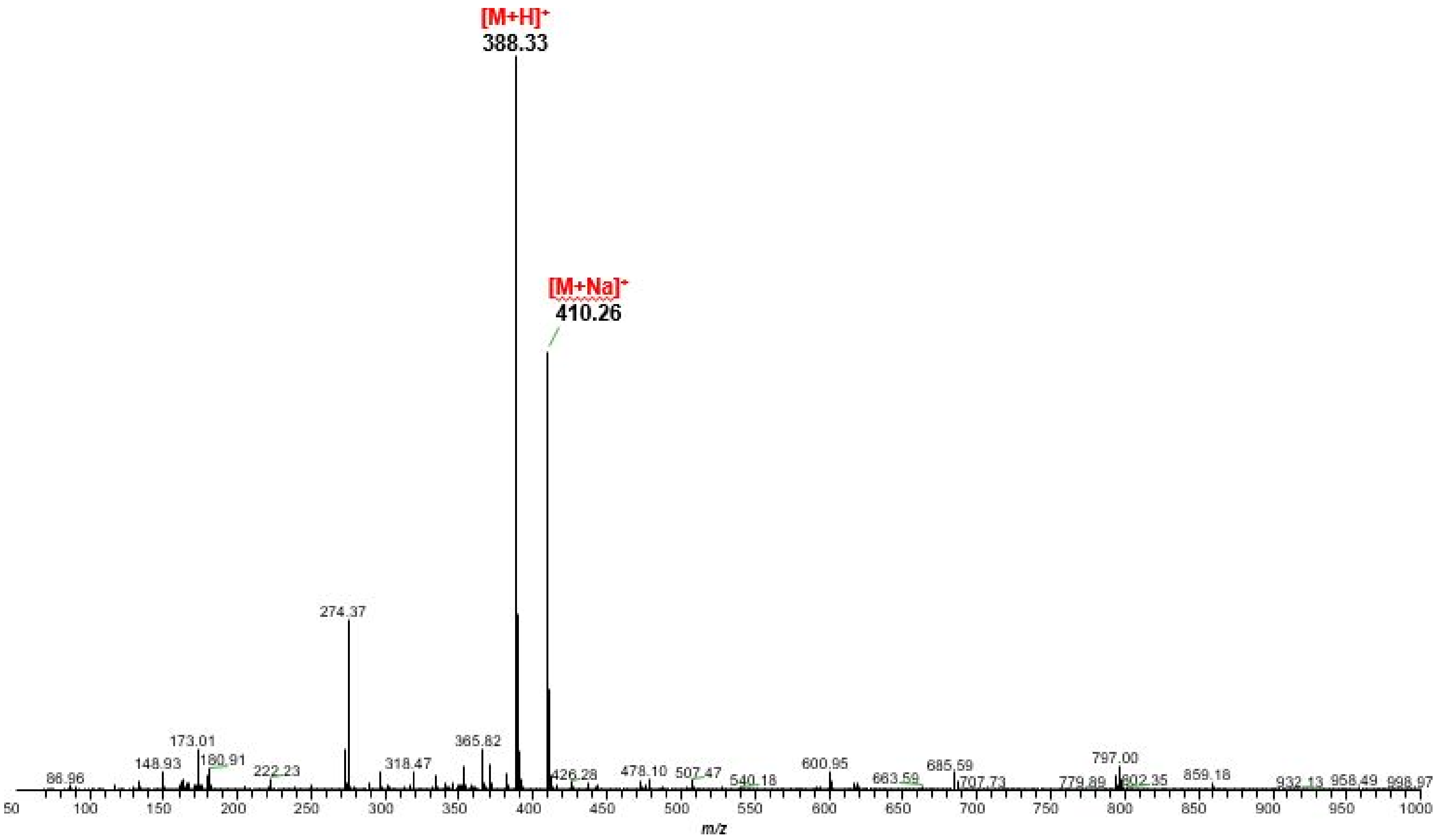

Figure S62. LC-MS spectrum of 1-(4-(2,4-difluorophenyl)-1*H*-1,2,3-triazol-1-yl)-3-(2-isopropyl-5-methylphenoxy) propan-2-ol (**3n**).

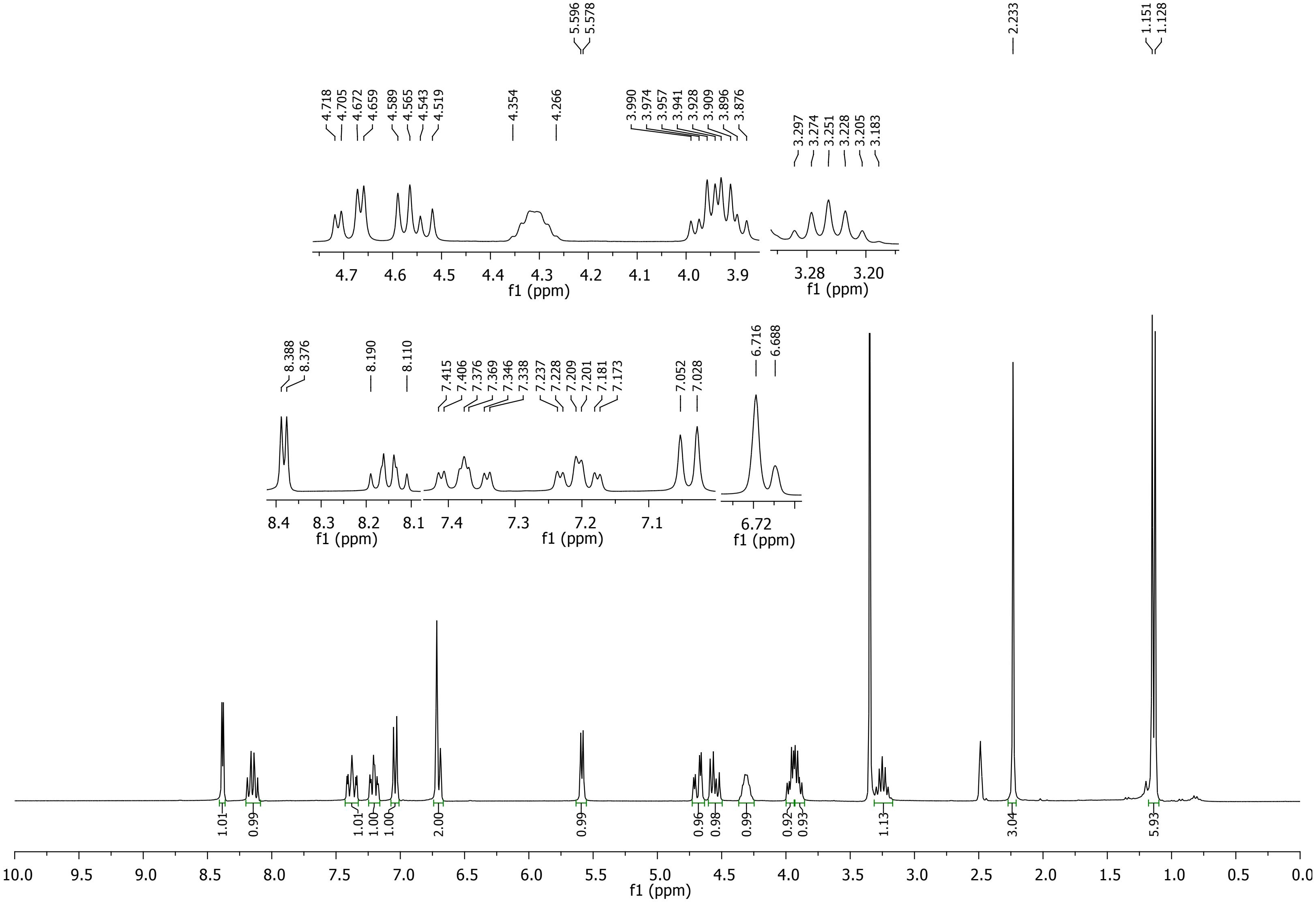

Figure S63. ^{1}H NMR spectrum (300 MHz, DMSO-d_6) of 1-(4-(2,4-difluorophenyl)-1*H*-1,2,3-triazol-1-yl)-3-(2-isopropyl-5-methylphenoxy) propan-2-ol (**3n**).

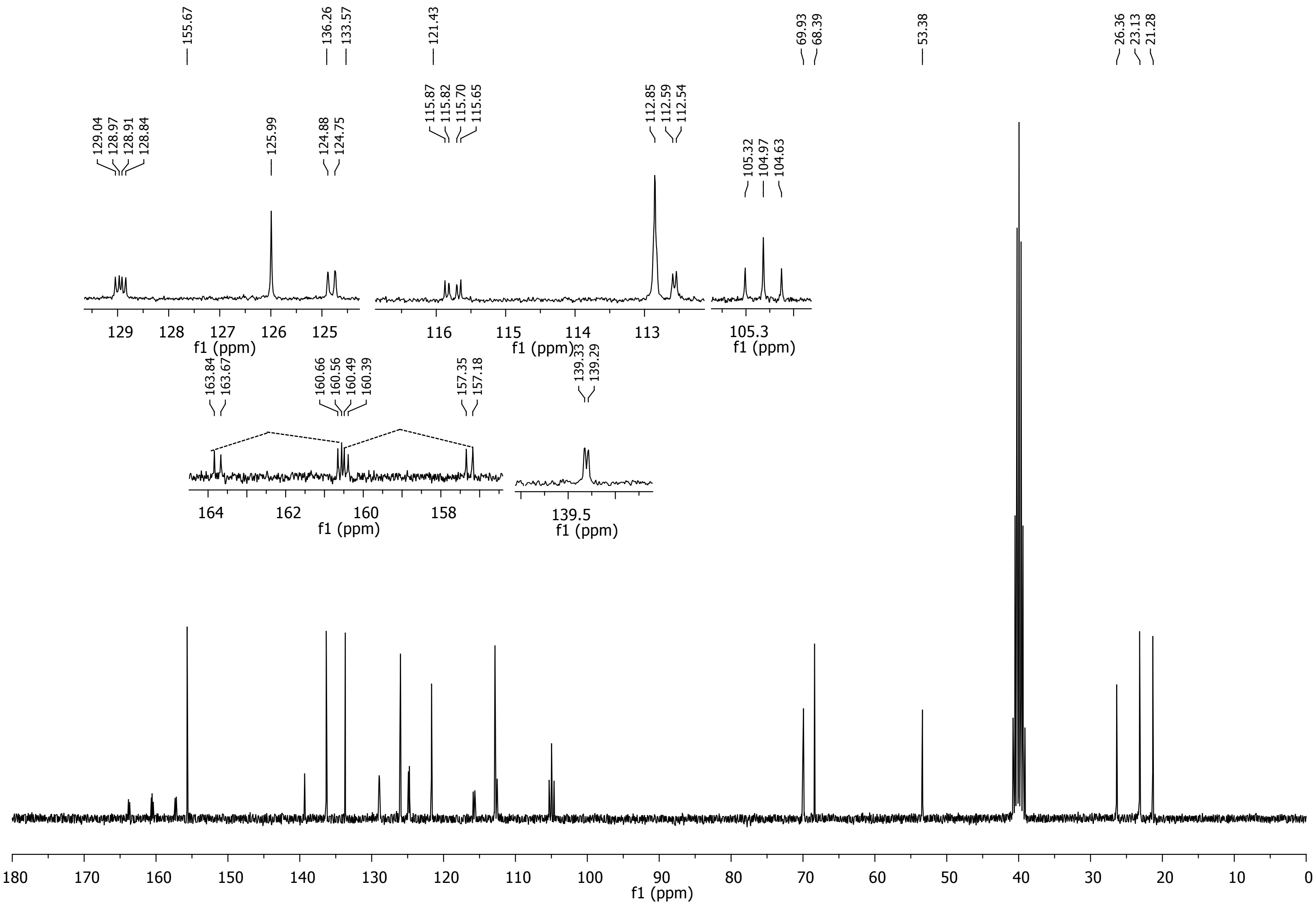

Figure S64. ^{13}C NMR spectrum (75 MHz, DMSO-d_6) of 1-(4-(2,4-difluorophenyl)-1*H*-1,2,3-triazol-1-yl)-3-(2-isopropyl-5-methylphenoxy) propan-2-ol (**3n**).

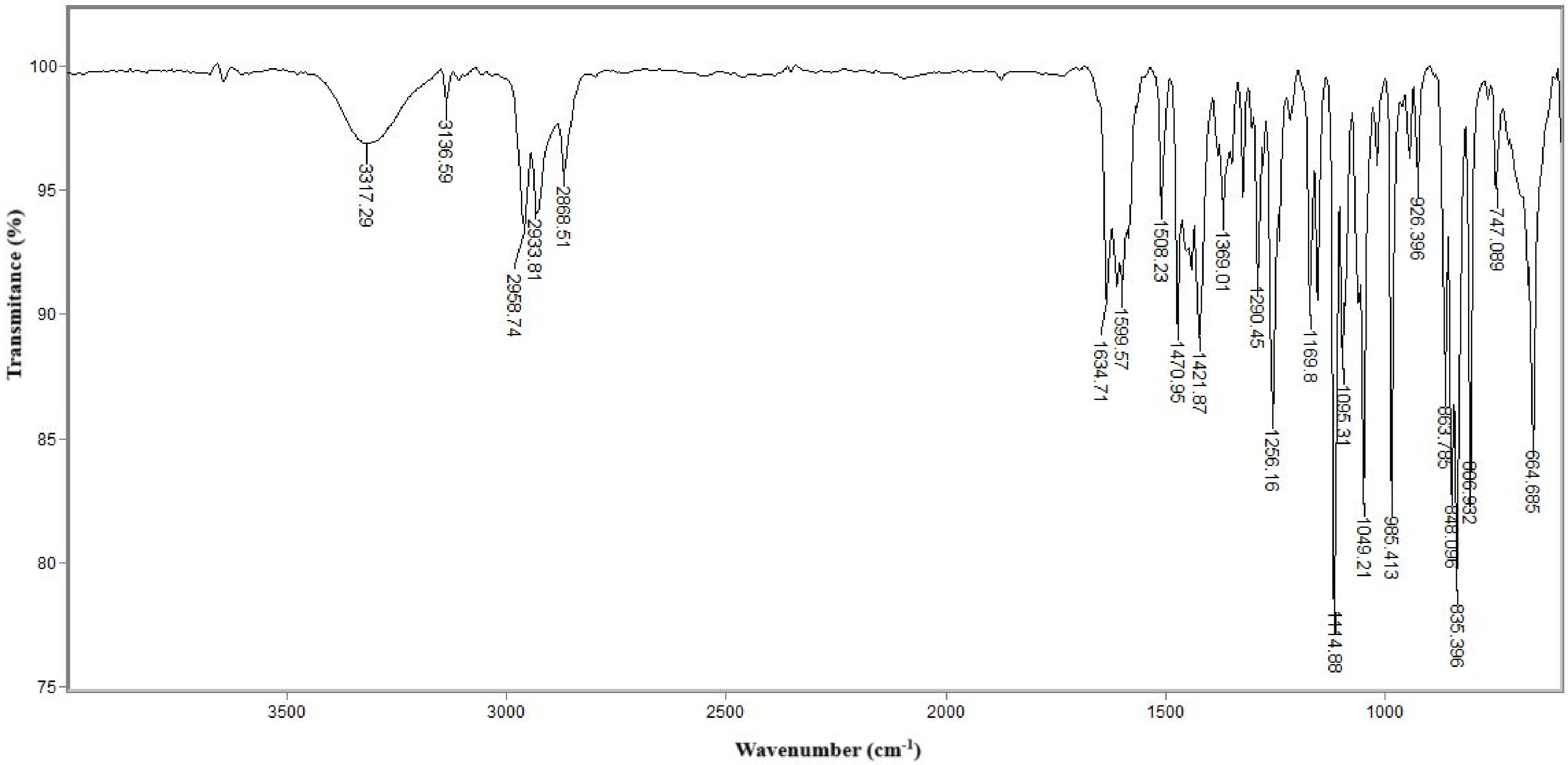

Figure S65. FTIR spectrum (ATR) of 1-(4-(3,5-difluorophenyl)-1*H*-1,2,3-triazol-1-yl)-3-(2-isopropyl-5-methylphenoxy) propan-2-ol (**3o**).

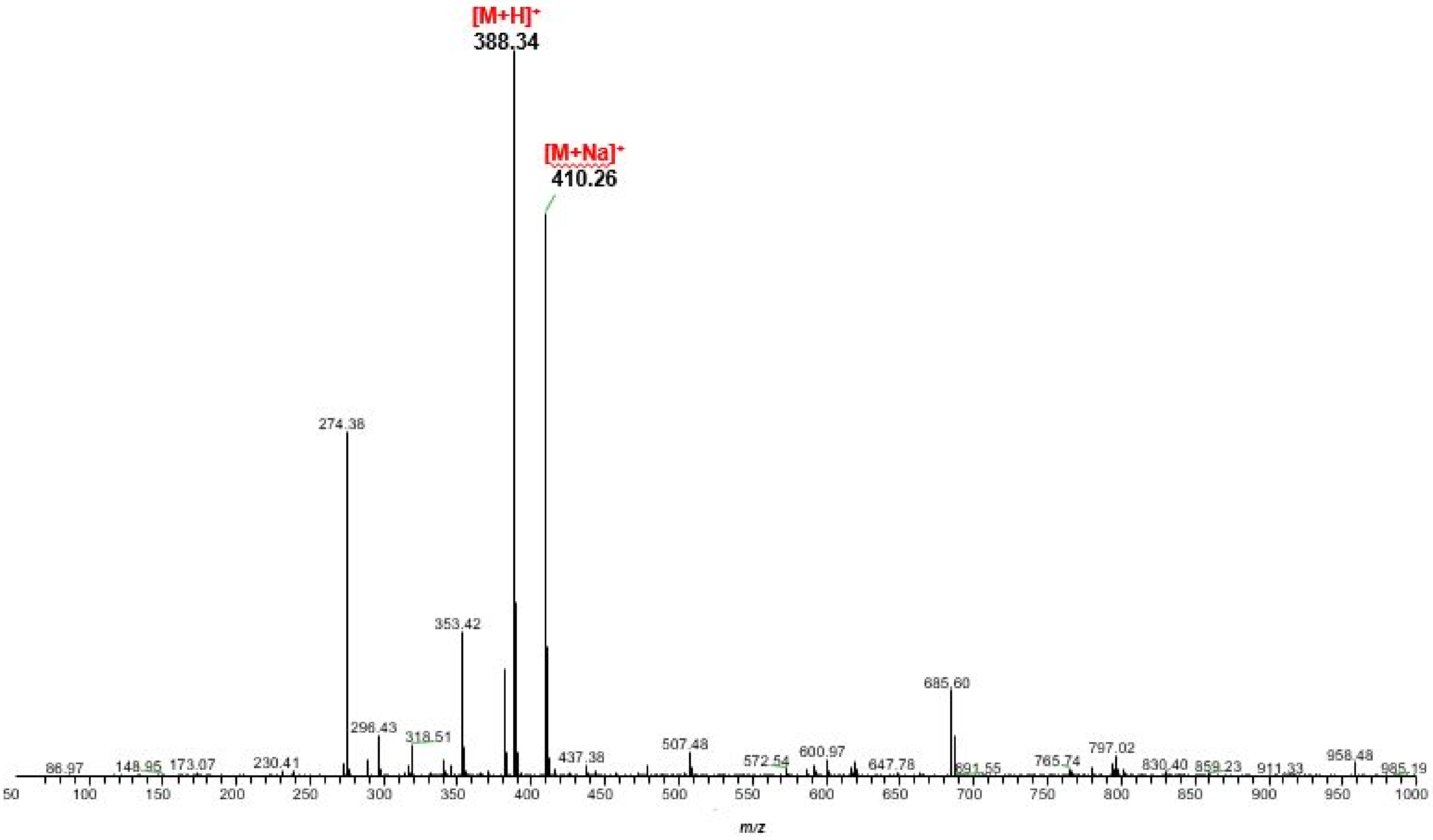

Figure S66. LC-MS spectrum of 1-(4-(3,5-difluorophenyl)-1*H*-1,2,3-triazol-1-yl)-3-(2-isopropyl-5-methylphenoxy) propan-2-ol (**3o**).

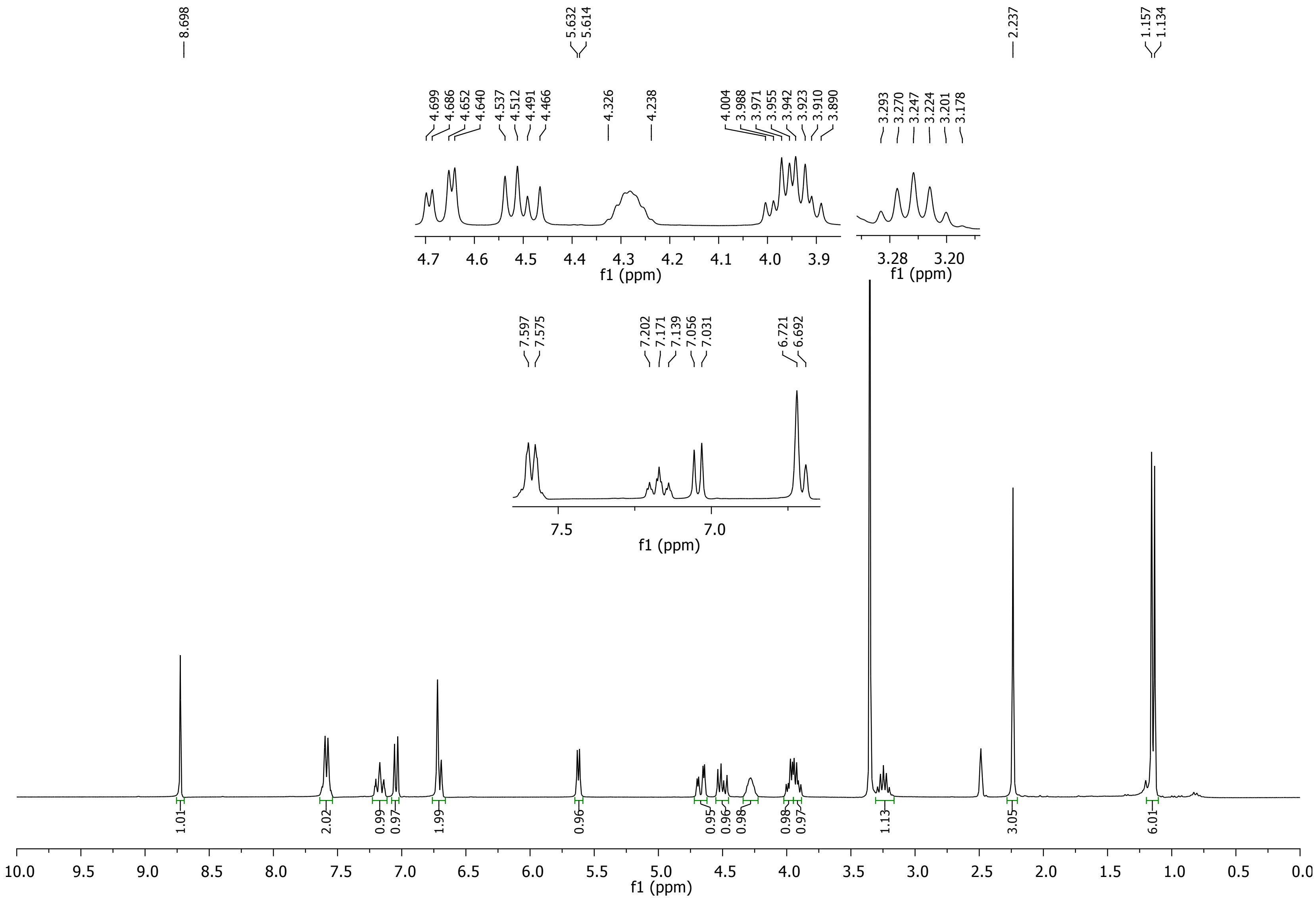

Figure S67. ^{1}H NMR spectrum (300 MHz, DMSO-d_6) of 1-(4-(3,5-difluorophenyl)-1*H*-1,2,3-triazol-1-yl)-3-(2-isopropyl-5-methylphenoxy) propan-2-ol (**3o**).

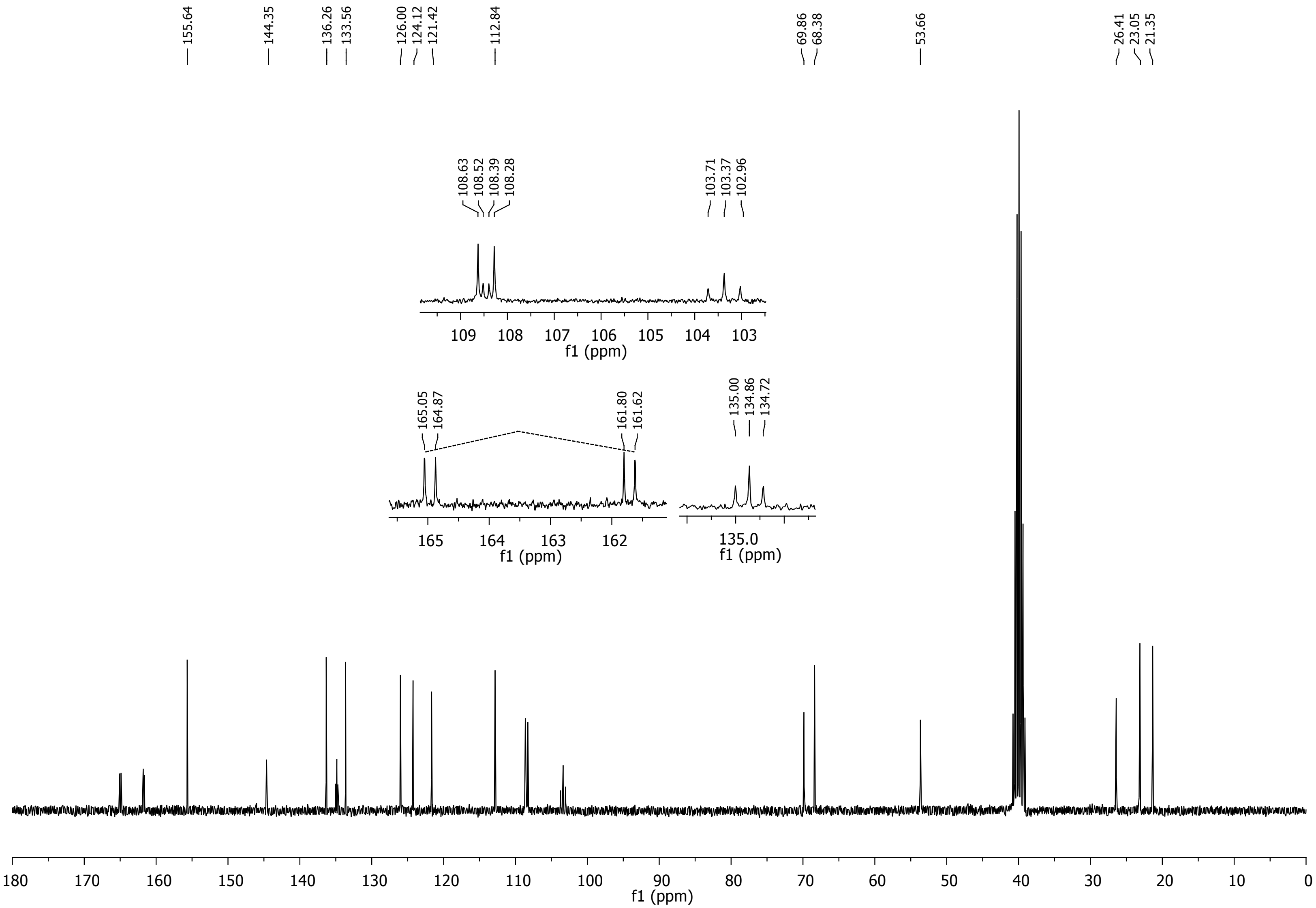

Figure S68. ^{13}C NMR spectrum (75 MHz, DMSO-d_6) of 1-(4-(3,5-difluorophenyl)-1*H*-1,2,3-triazol-1-yl)-3-(2-isopropyl-5-methylphenoxy) propan-2-ol (**3o**).

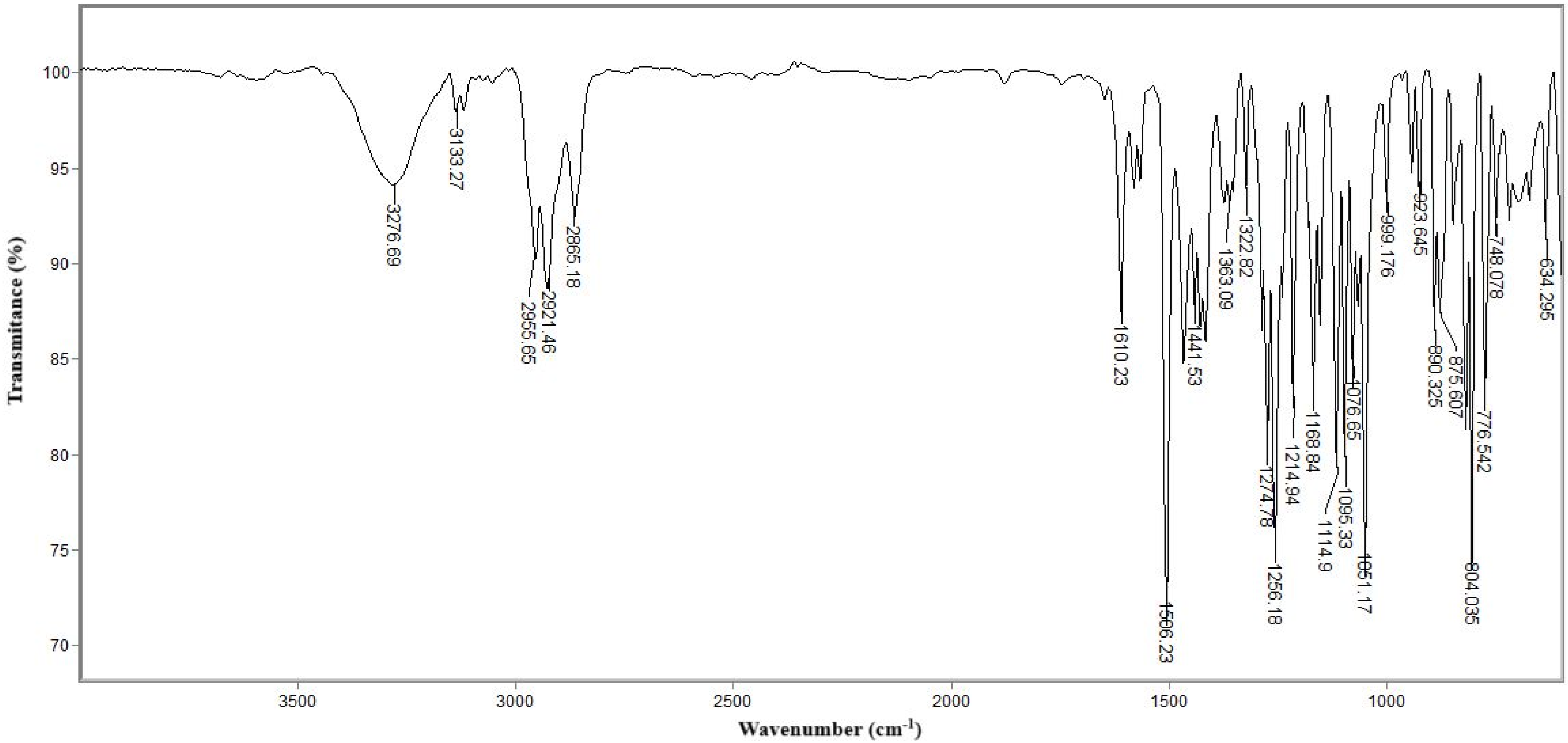

Figure S69. FTIR spectrum (ATR) of 1-(4-(3,4-difluorophenyl)-1*H*-1,2,3-triazol-1-yl)-3-(2-isopropyl-5-methylphenoxy) propan-2-ol (**3p**).

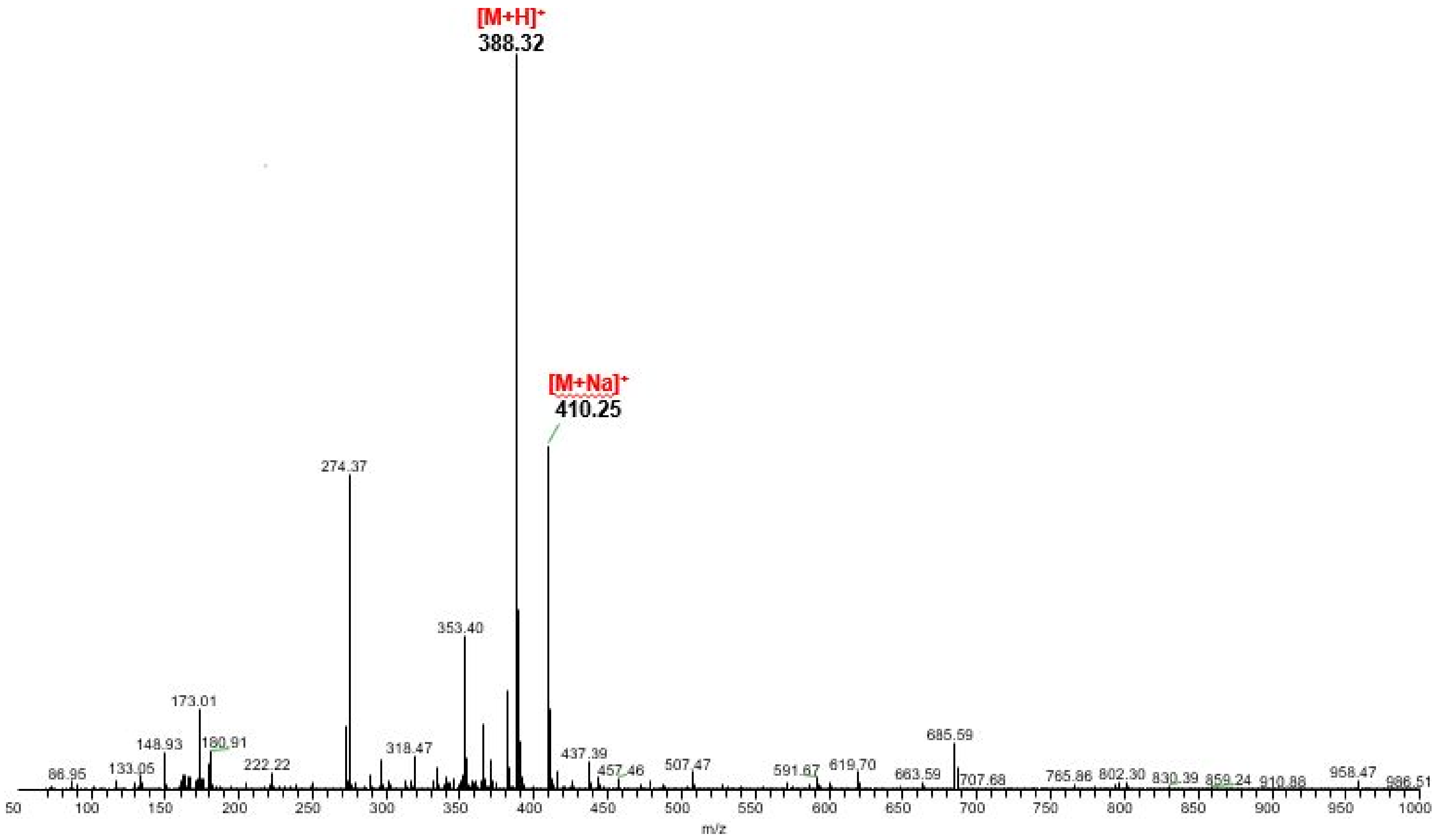

Figure S70. LC-MS spectrum of 1-(4-(3,4-difluorophenyl)-1*H*-1,2,3-triazol-1-yl)-3-(2-isopropyl-5-methylphenoxy) propan-2-ol (**3p**).

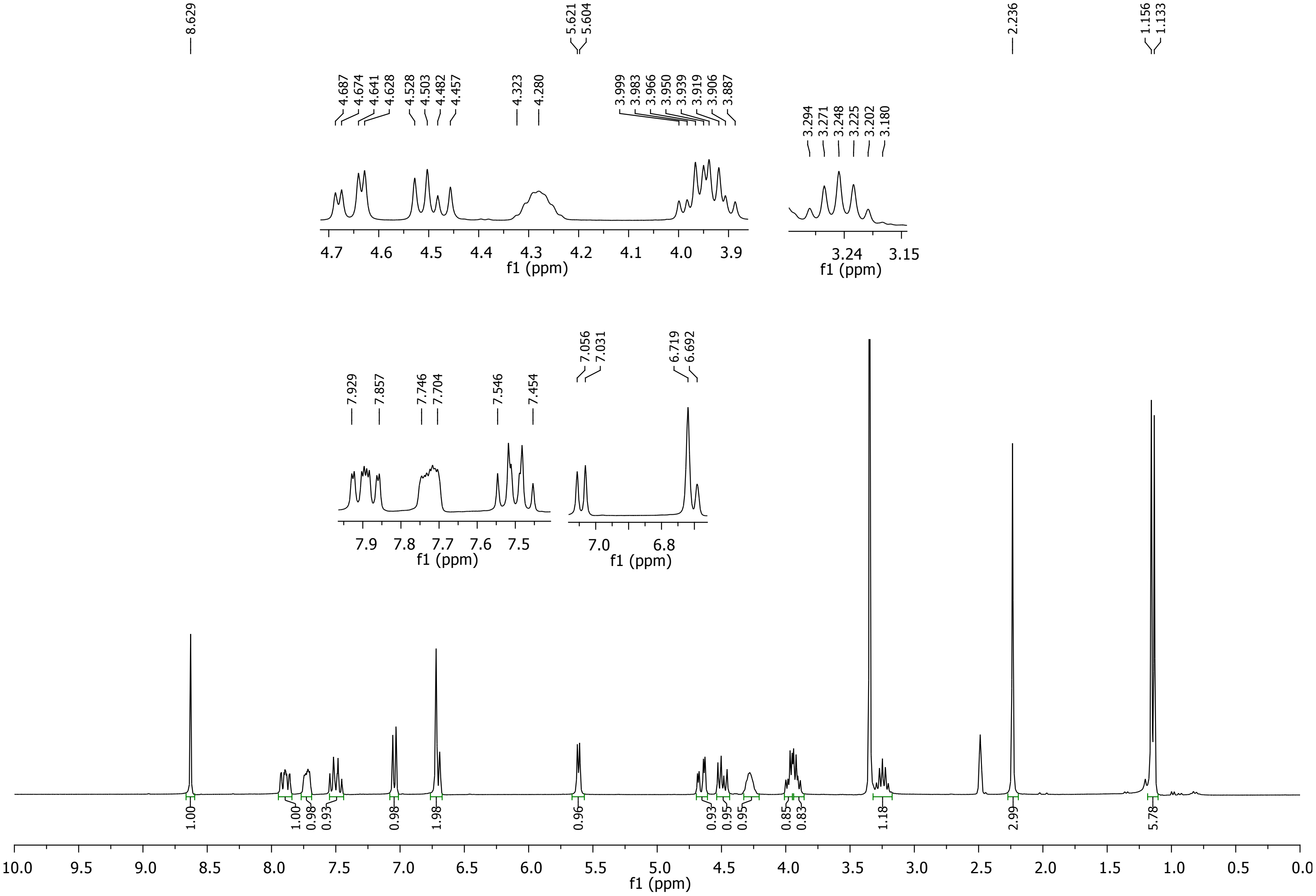

Figure S71. ^{1}H NMR spectrum (300 MHz, DMSO-d_6) of 1-(4-(3,4-difluorophenyl)-1*H*-1,2,3-triazol-1-yl)-3-(2-isopropyl-5-methylphenoxy) propan-2-ol (**3p**).

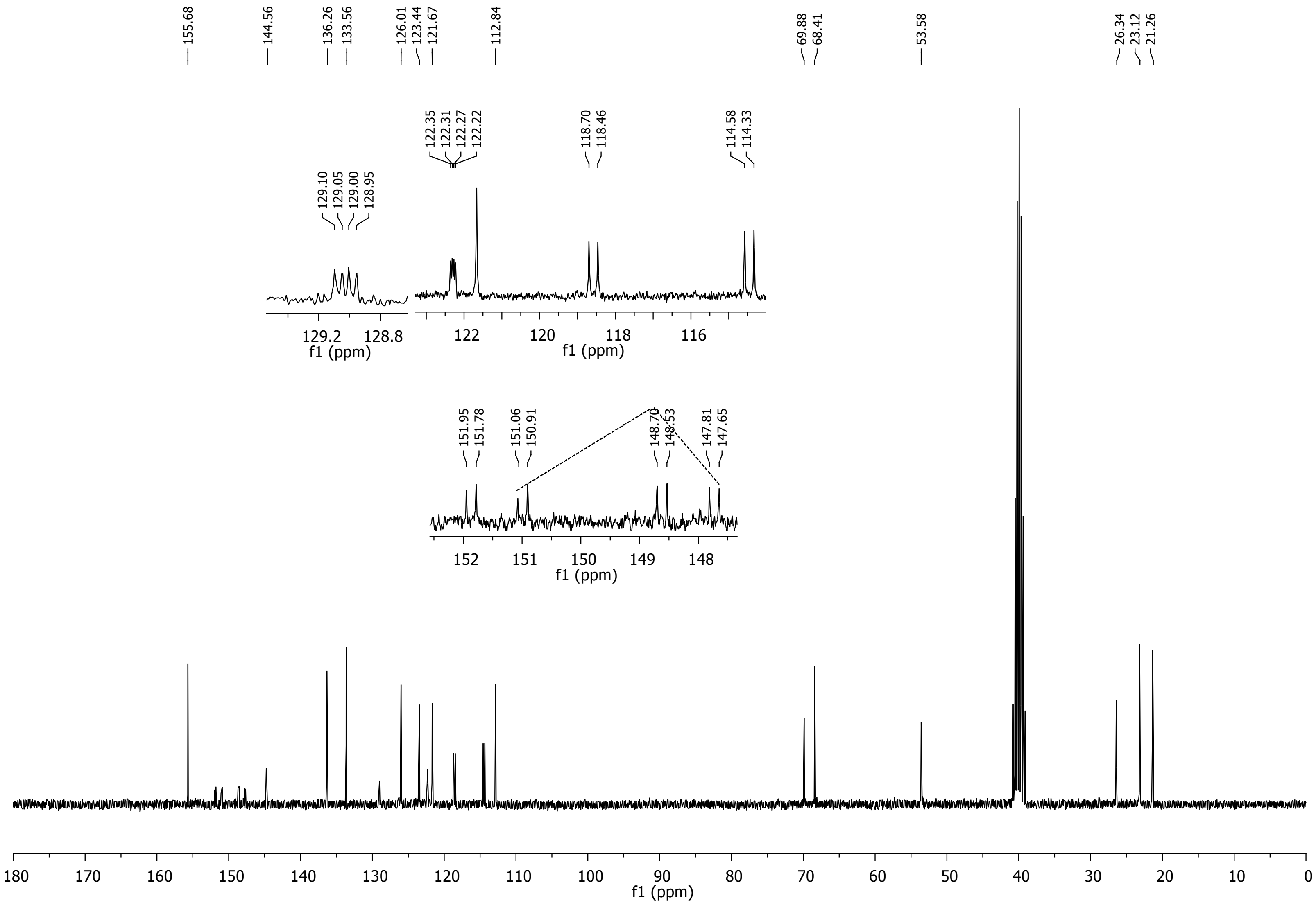

Figure S72. ^{13}C NMR spectrum (75 MHz, DMSO-d_6) of 1-(4-(3,4-difluorophenyl)-1*H*-1,2,3-triazol-1-yl)-3-(2-isopropyl-5-methylphenoxy) propan-2-ol (**3p**).

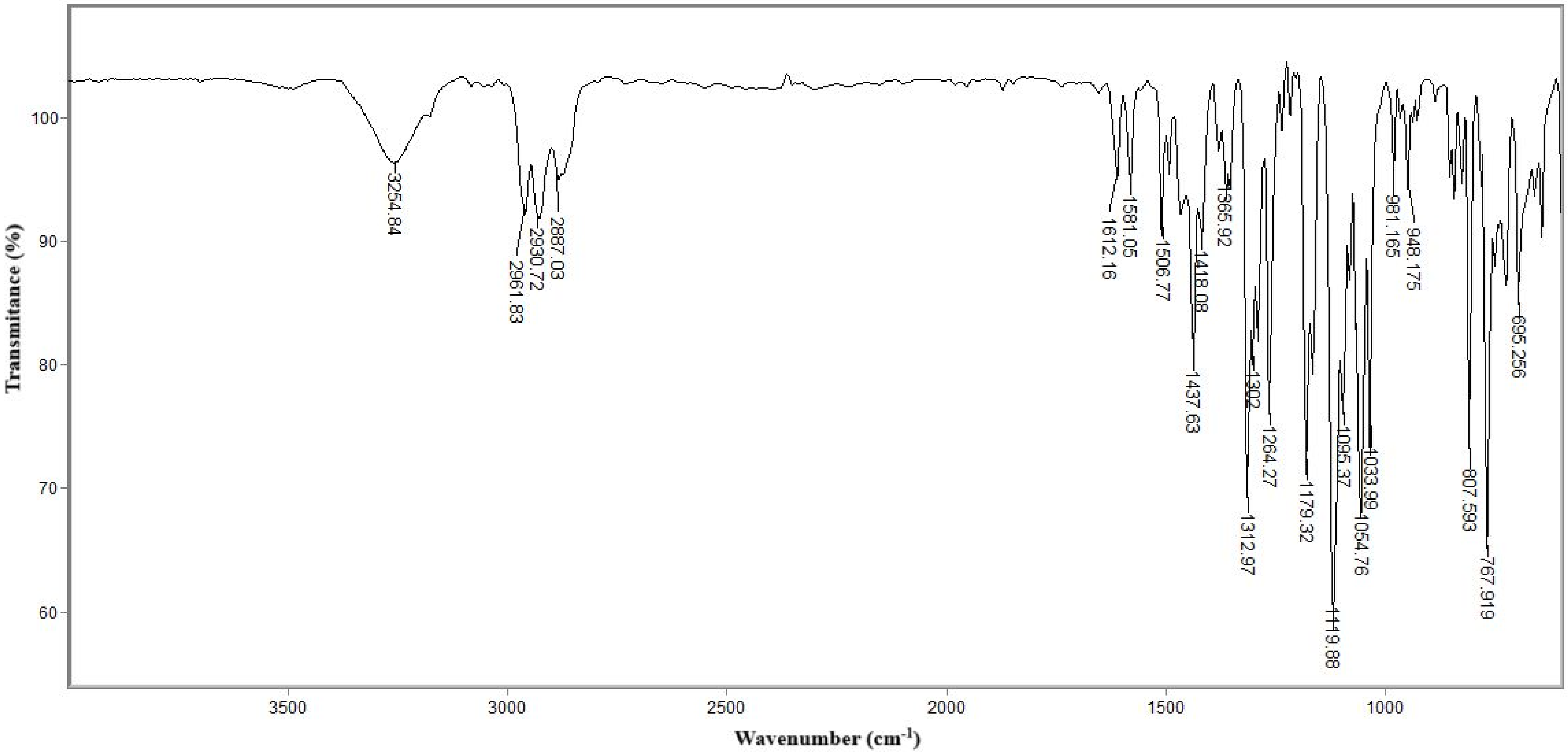

Figure S73. FTIR spectrum (ATR) of 1-(2-isopropyl-5-methylphenoxy)-3-(4-(2-(trifluoromethyl)phenyl)-1*H*-1,2,3-triazol-1-yl) propan-2-ol (**3q**).

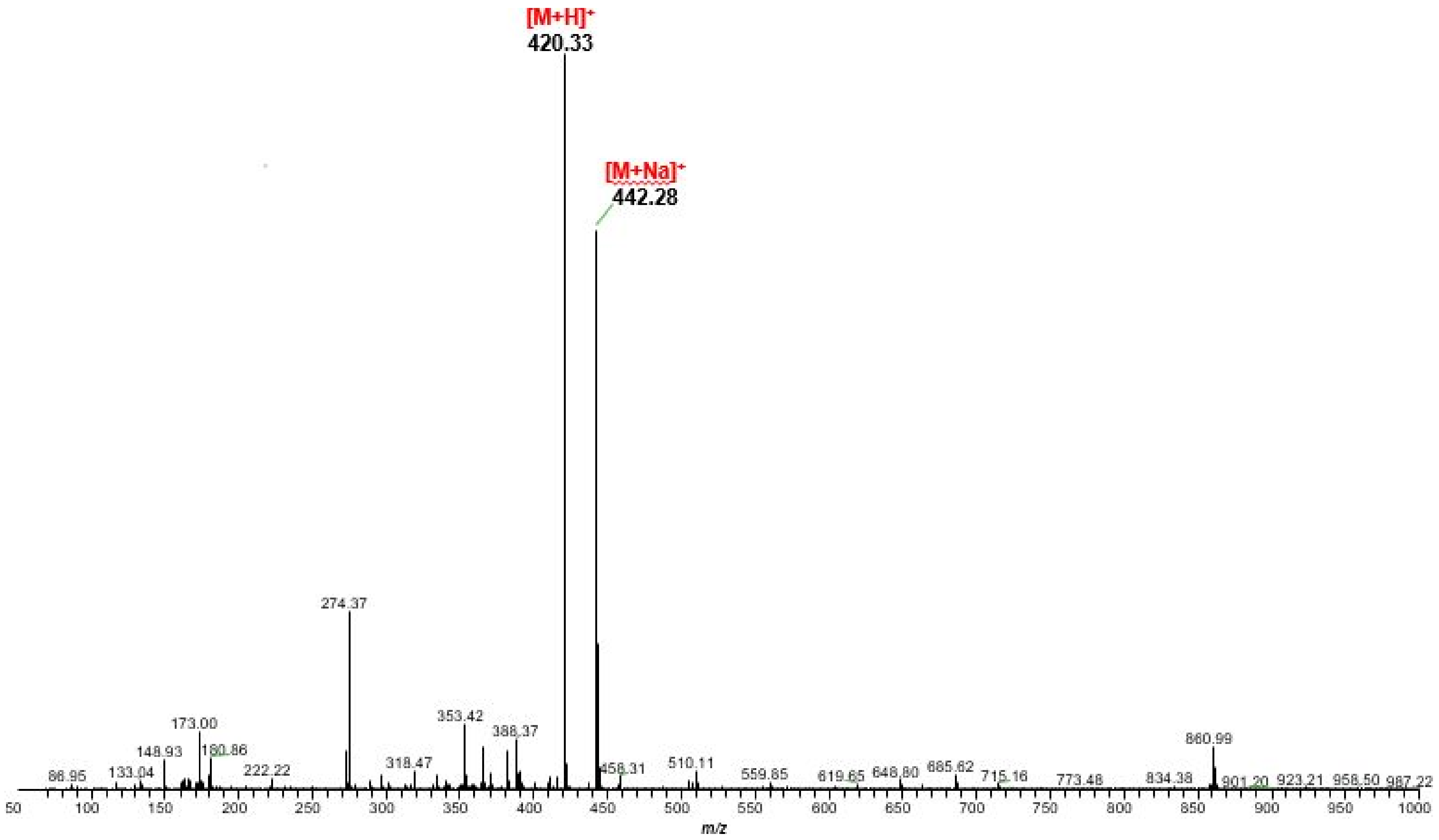

Figure S74. LC-MS spectrum of 1-(2-isopropyl-5-methylphenoxy)-3-(4-(2-(trifluoromethyl)phenyl)-1*H*-1,2,3-triazol-1-yl) propan-2-ol (**3q**).

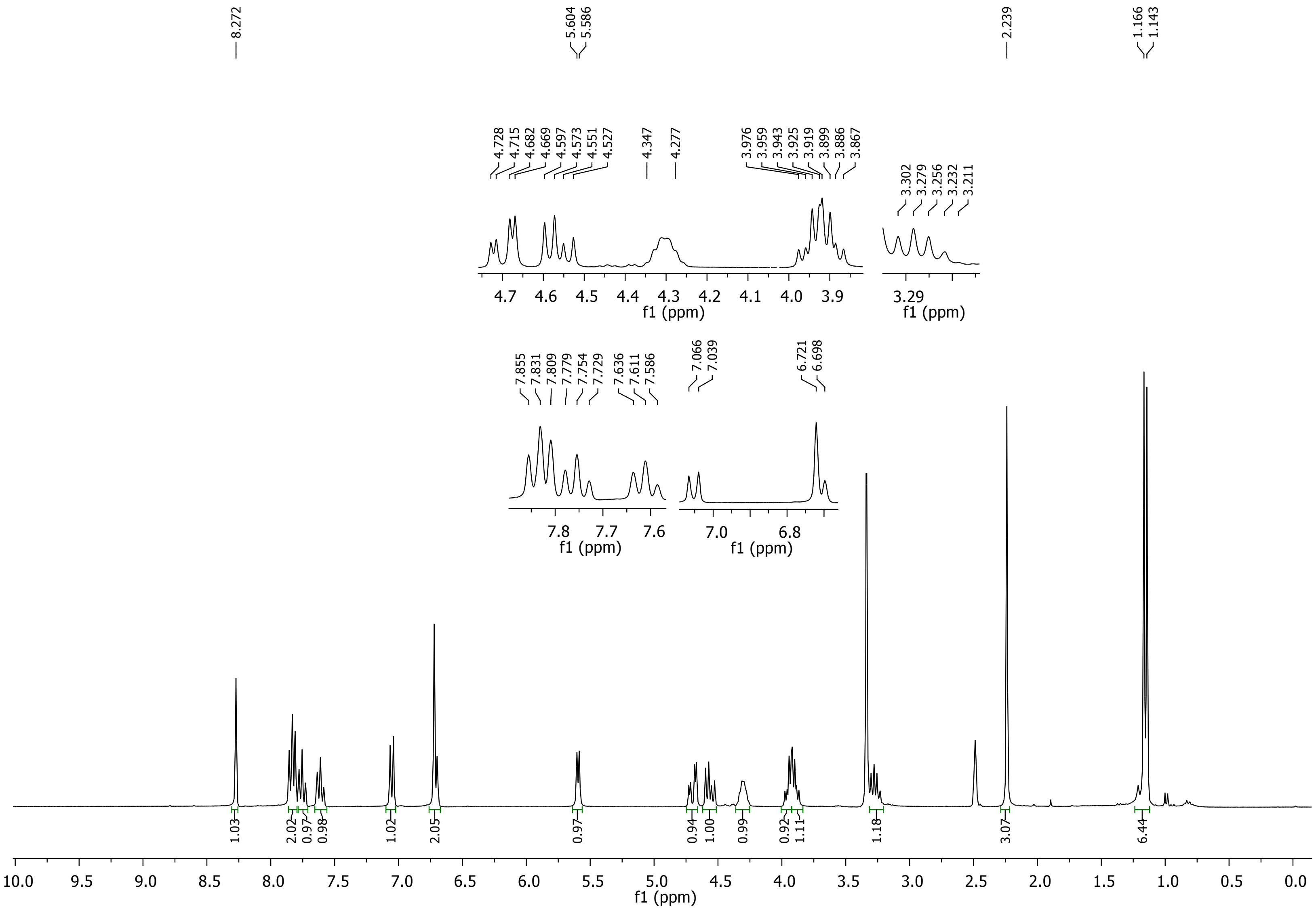

Figure S75. ^{1}H NMR spectrum (300 MHz, DMSO-d_6) of 1-(2-isopropyl-5-methylphenoxy)-3-(4-(2-(trifluoromethyl)phenyl)-1*H*-1,2,3-triazol-1-yl) propan-2-ol (**3q**).

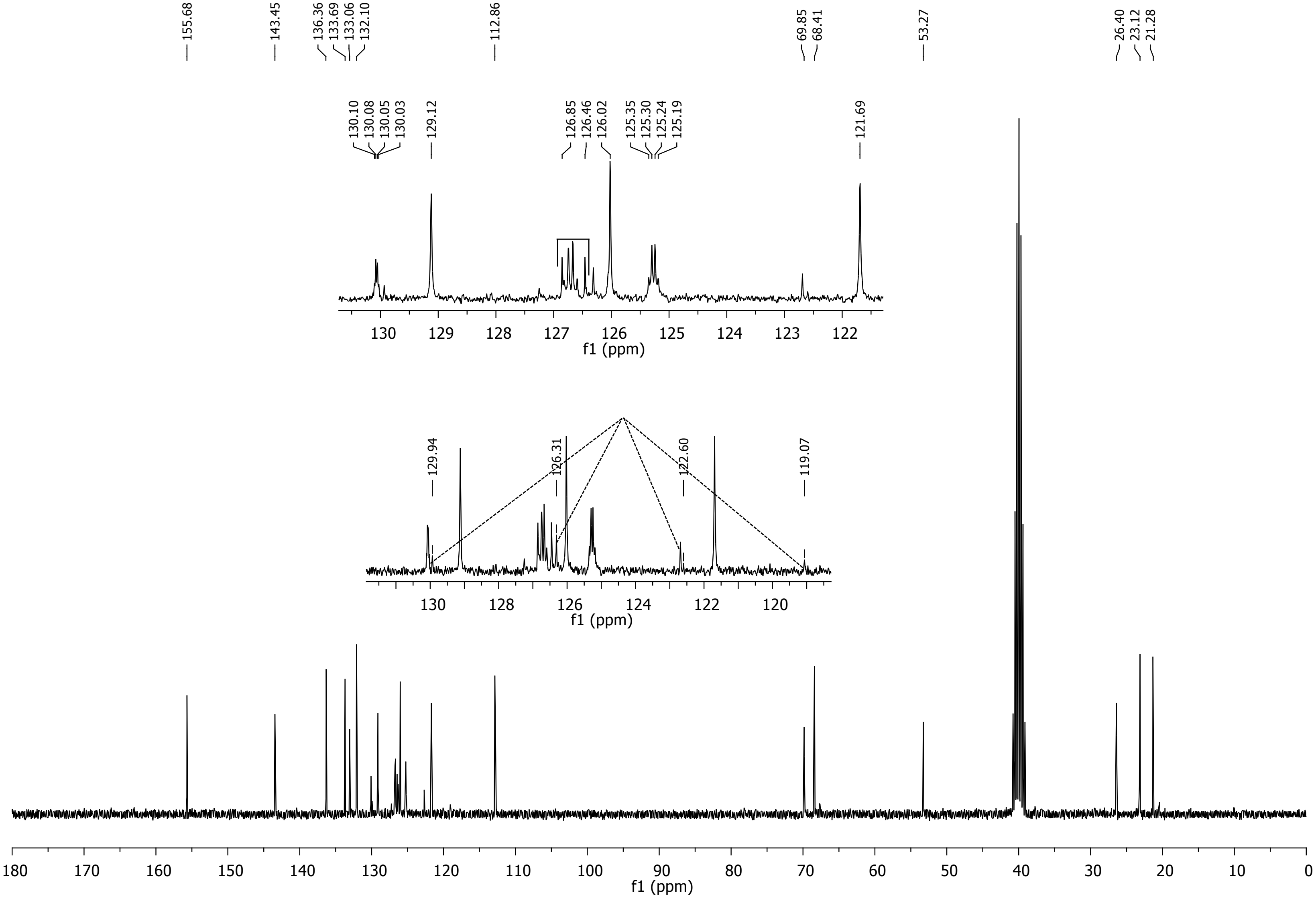

Figure. S76. ^{13}C NMR spectrum (75 MHz, DMSO-d_6) of 1-(2-isopropyl-5-methylphenoxy)-3-(4-(2-(trifluoromethyl)phenyl)-1*H*-1,2,3-triazol-1-yl)propan-2-ol (**3q**).

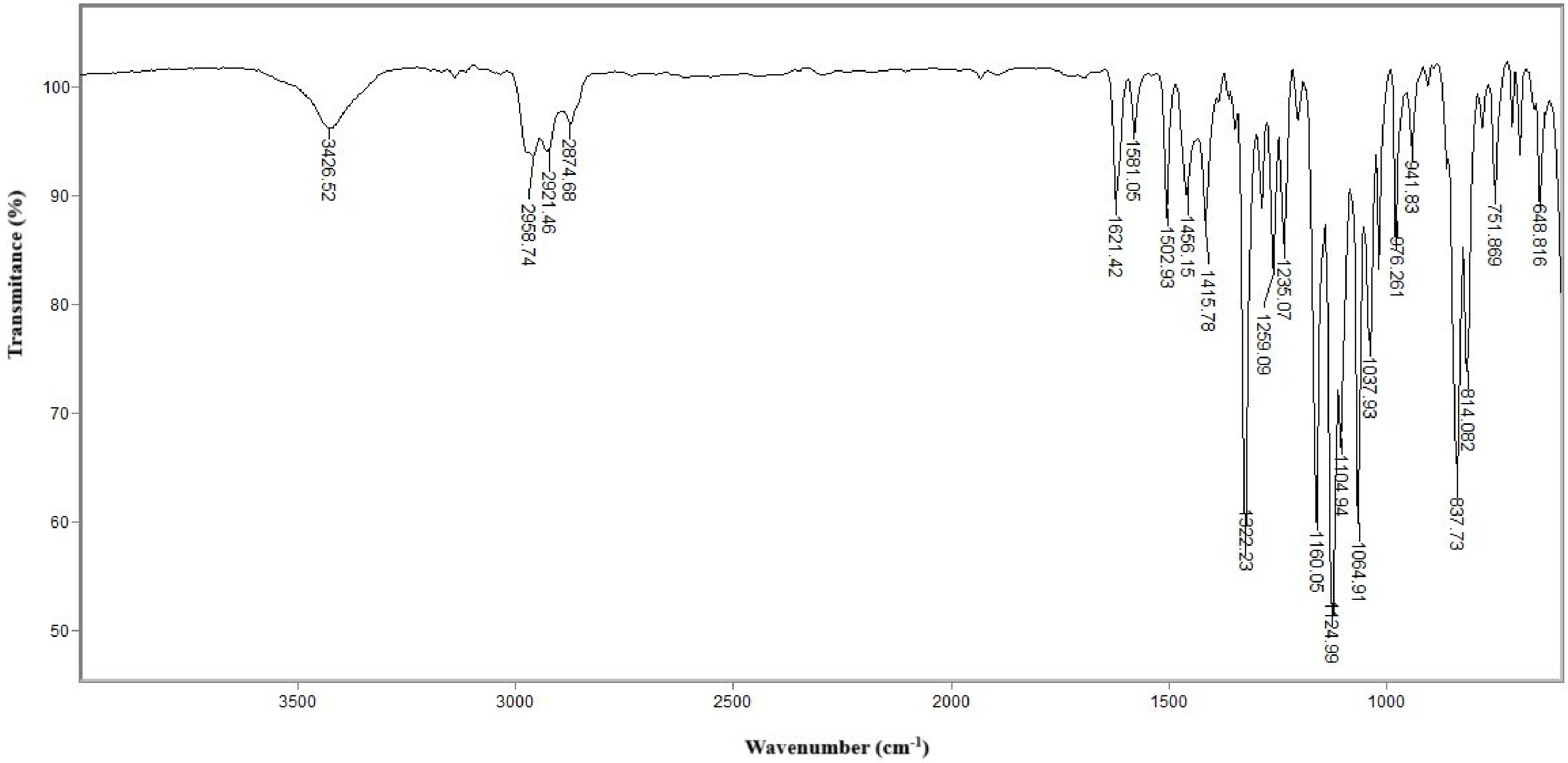

Figure S77. FTIR spectrum (ATR) of 1-(2-isopropyl-5-methylphenoxy)-3-(4-(4-(trifluoromethyl)phenyl)-1*H*-1,2,3-triazol-1-yl) propan-2-ol (**3r**).

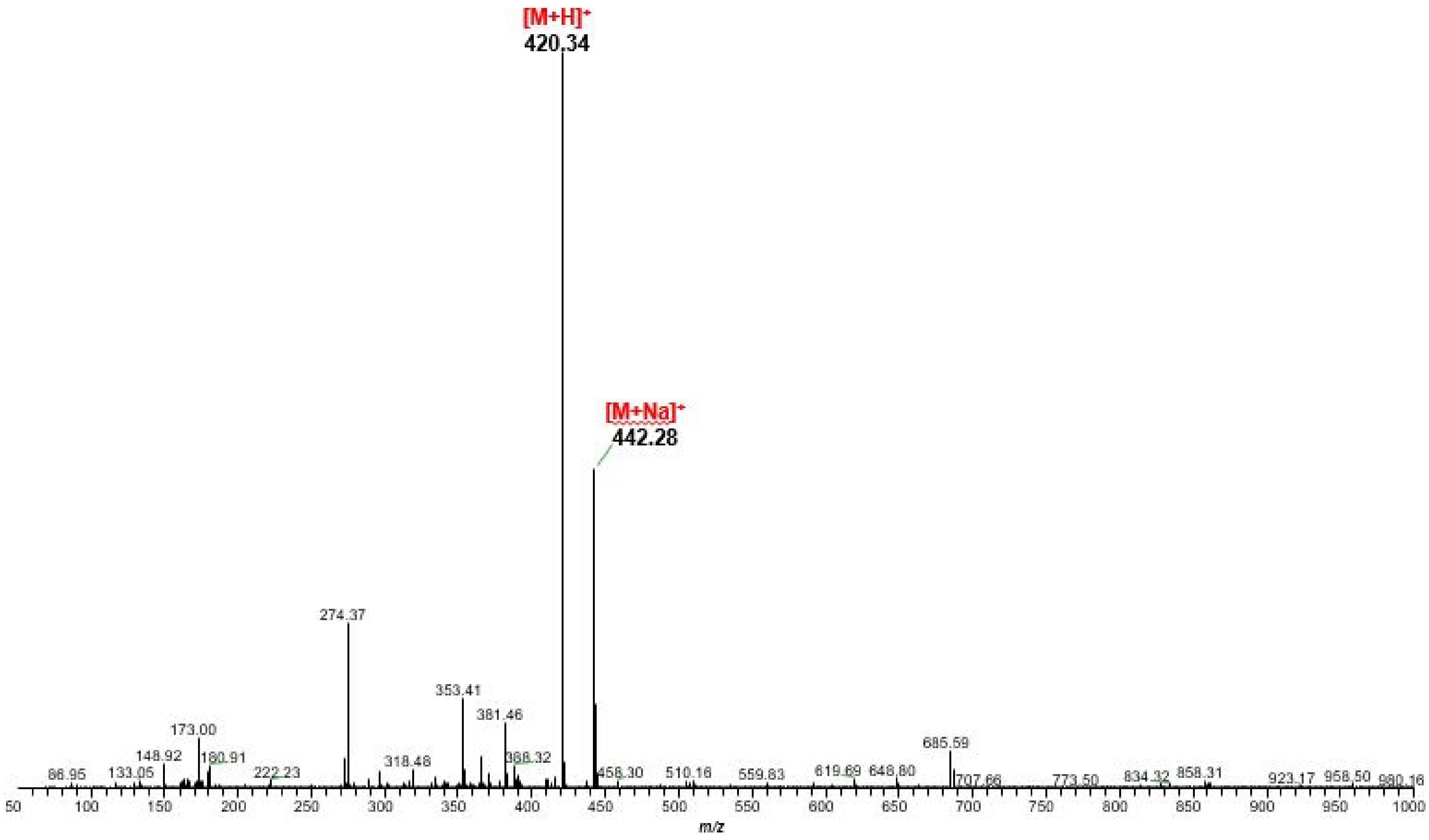

Figure S78. LC-MS spectrum of 1-(2-isopropyl-5-methylphenoxy)-3-(4-(4-(trifluoromethyl)phenyl)-1*H*-1,2,3-triazol-1-yl) propan-2-ol (**3r**).

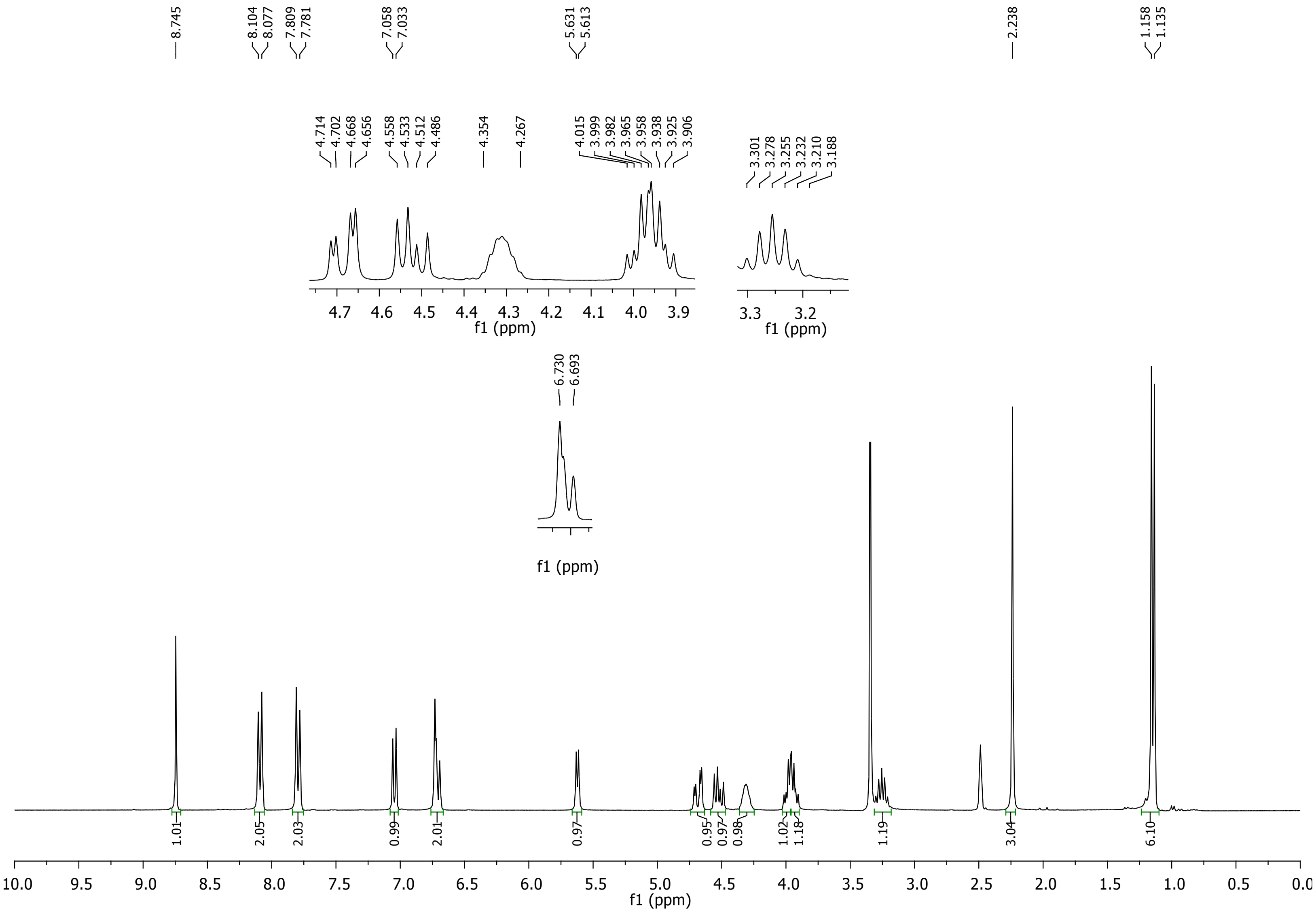

Figure S79. ^{1}H NMR spectrum (300 MHz, DMSO-d_6) of 1-(2-isopropyl-5-methylphenoxy)-3-(4-(4-(trifluoromethyl)phenyl)-1*H*-1,2,3-triazol-1-yl) propan-2-ol (**3r**).

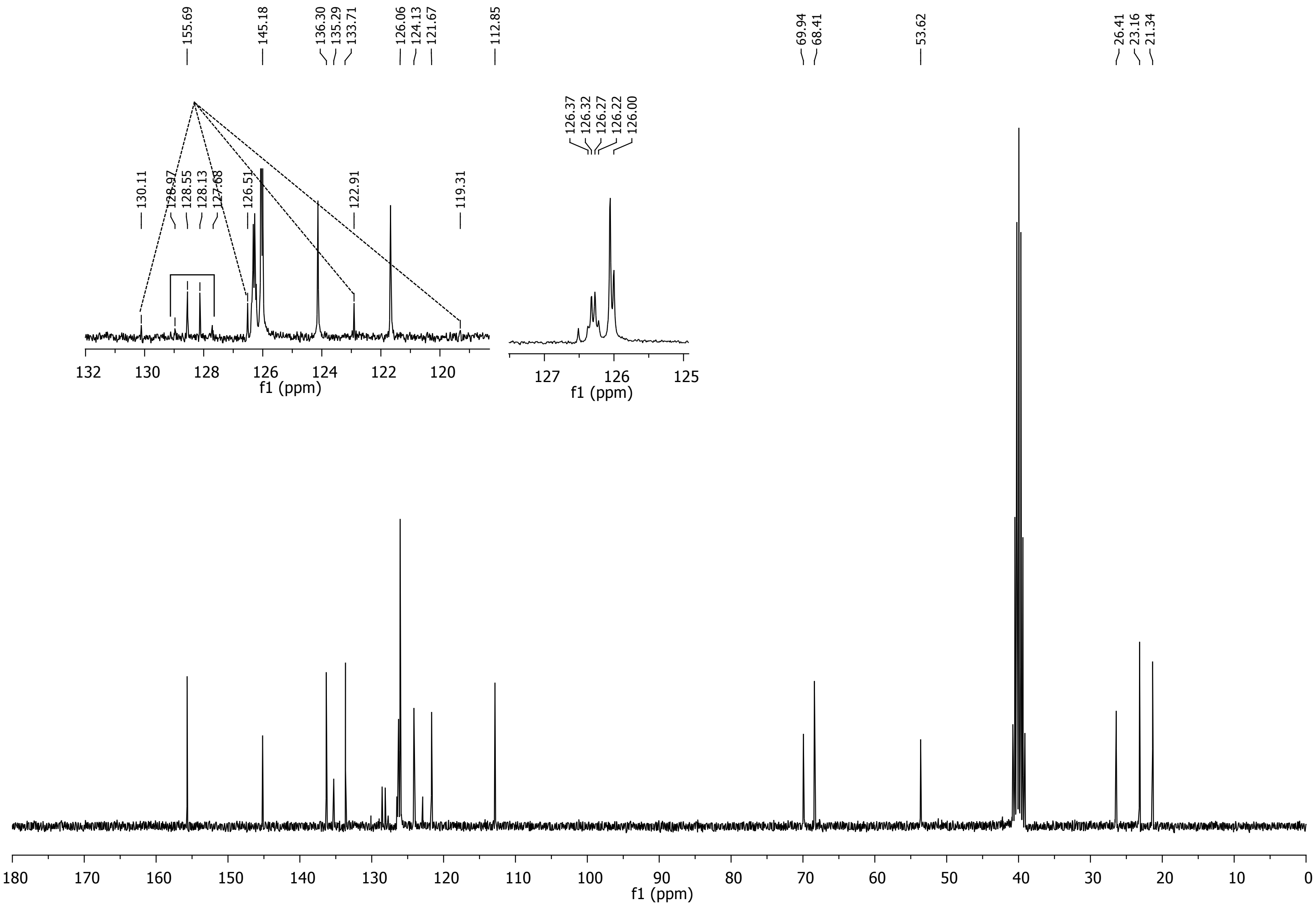

Figure S80. ^{13}C NMR spectrum (75 MHz, DMSO-d_6) of 1-(2-isopropyl-5-methylphenoxy)-3-(4-(4-(trifluoromethyl)phenyl)-1*H*-1,2,3-triazol-1-yl) propan-2-ol (**3r**).

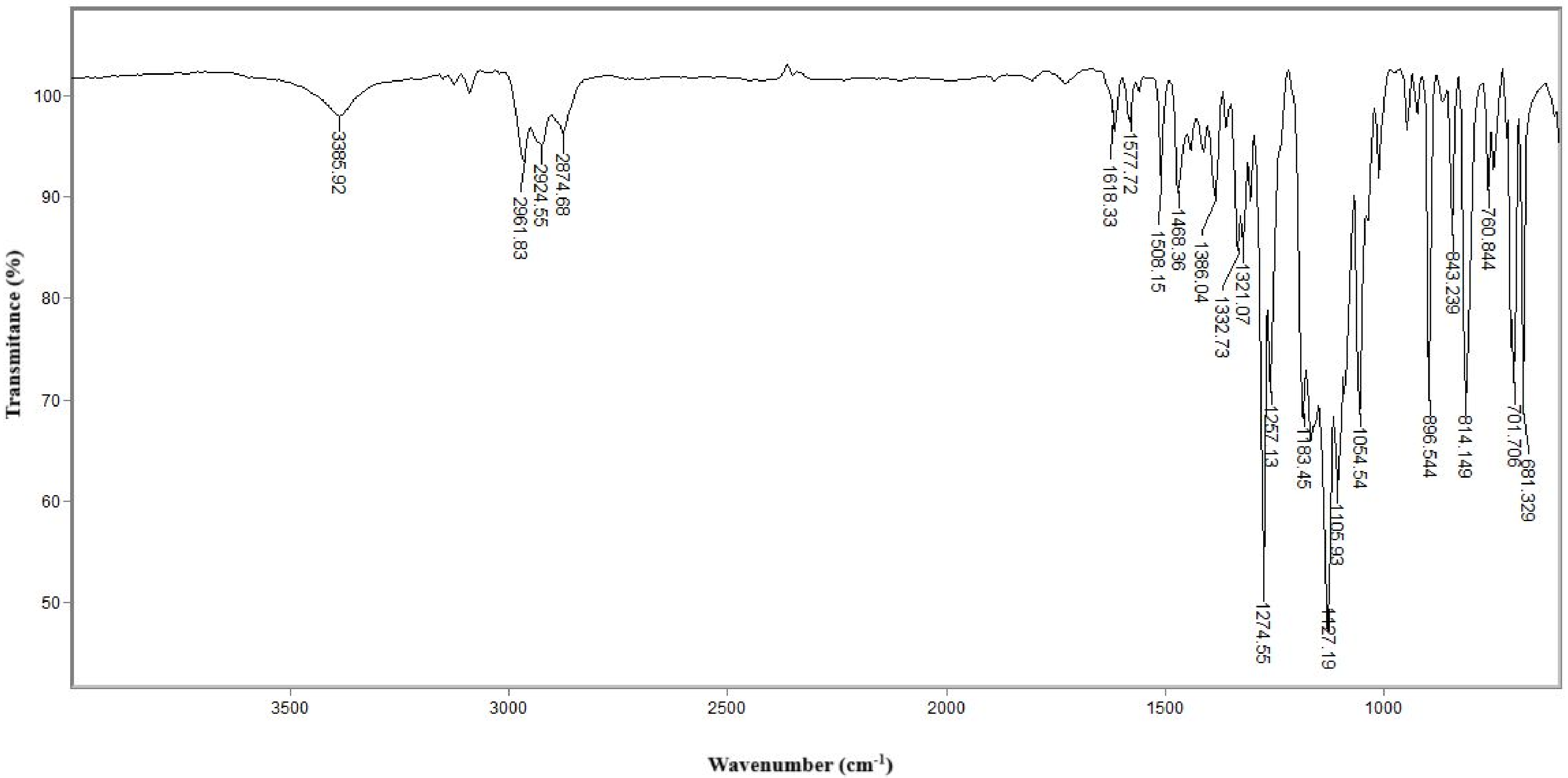

Figure S81. FTIR spectrum (ATR) of 1-(4-(3,5-bis(trifluoromethyl)phenyl)-1*H*-1,2,3-triazol-1-yl)-3-(2-isopropyl-5-methylphenoxy) propan-2-ol (**3s**).

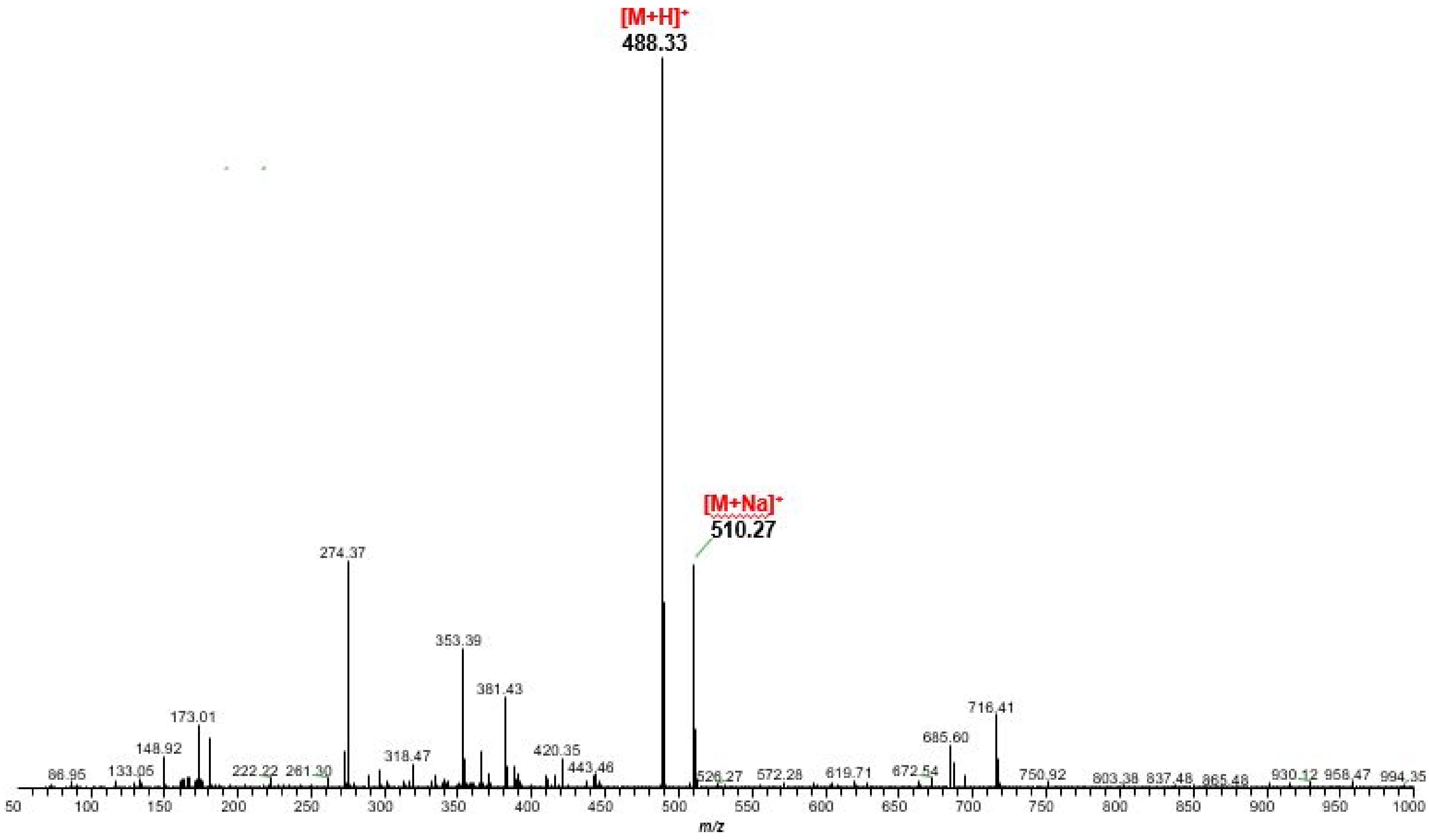

Figure S82. LC-MS spectrum of 1-(4-(3,5-bis(trifluoromethyl)phenyl)-1*H*-1,2,3-triazol-1-yl)-3-(2-isopropyl-5-methylphenoxy) propan-2-ol (**3s**).

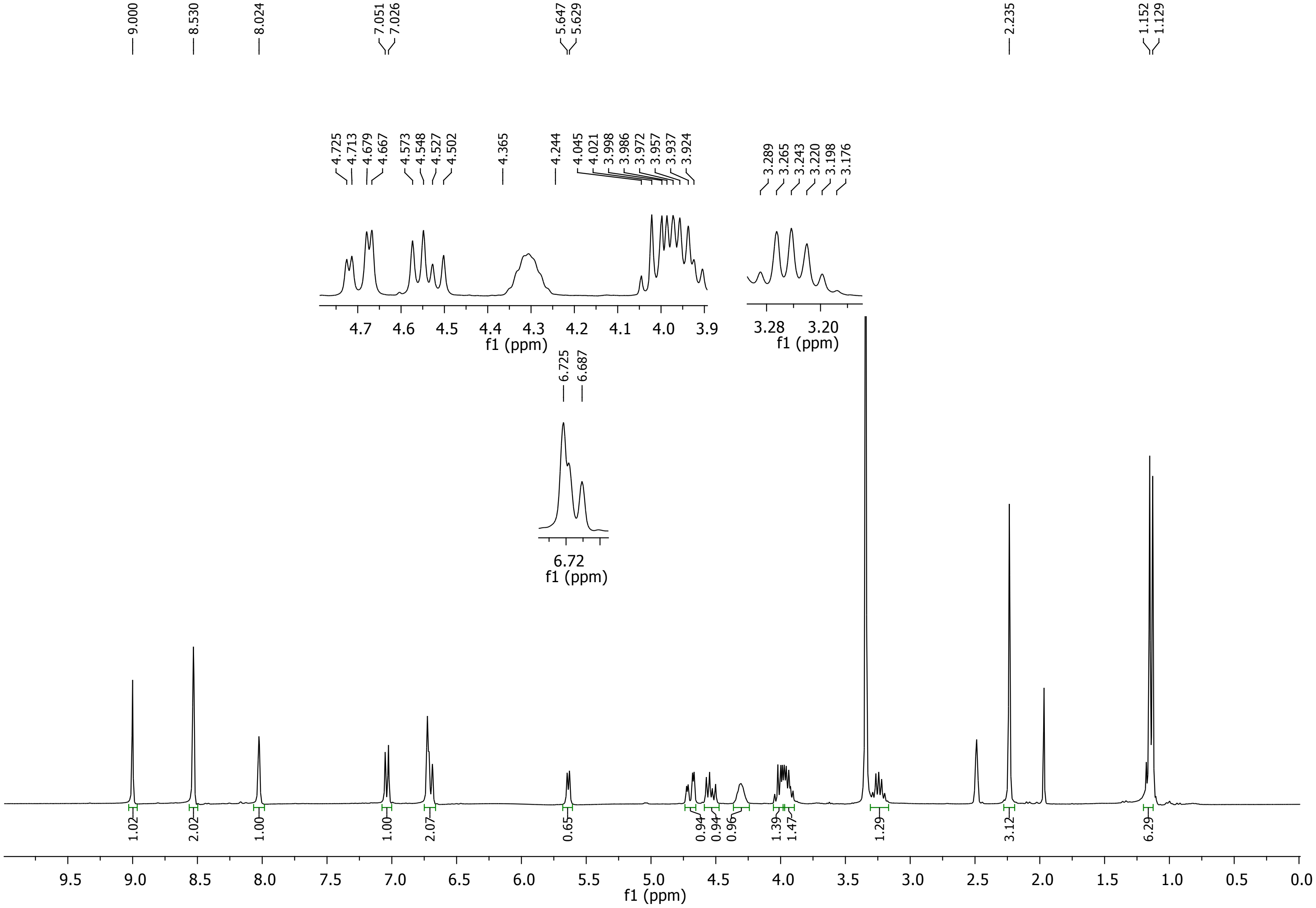

Figure S83. ^{1}H NMR spectrum (300 MHz, DMSO-d_6) of 1-(4-(3,5-bis(trifluoromethyl)phenyl)-1*H*-1,2,3-triazol-1-yl)-3-(2-isopropyl-5-methylphenoxy) propan-2-ol (**3s**).

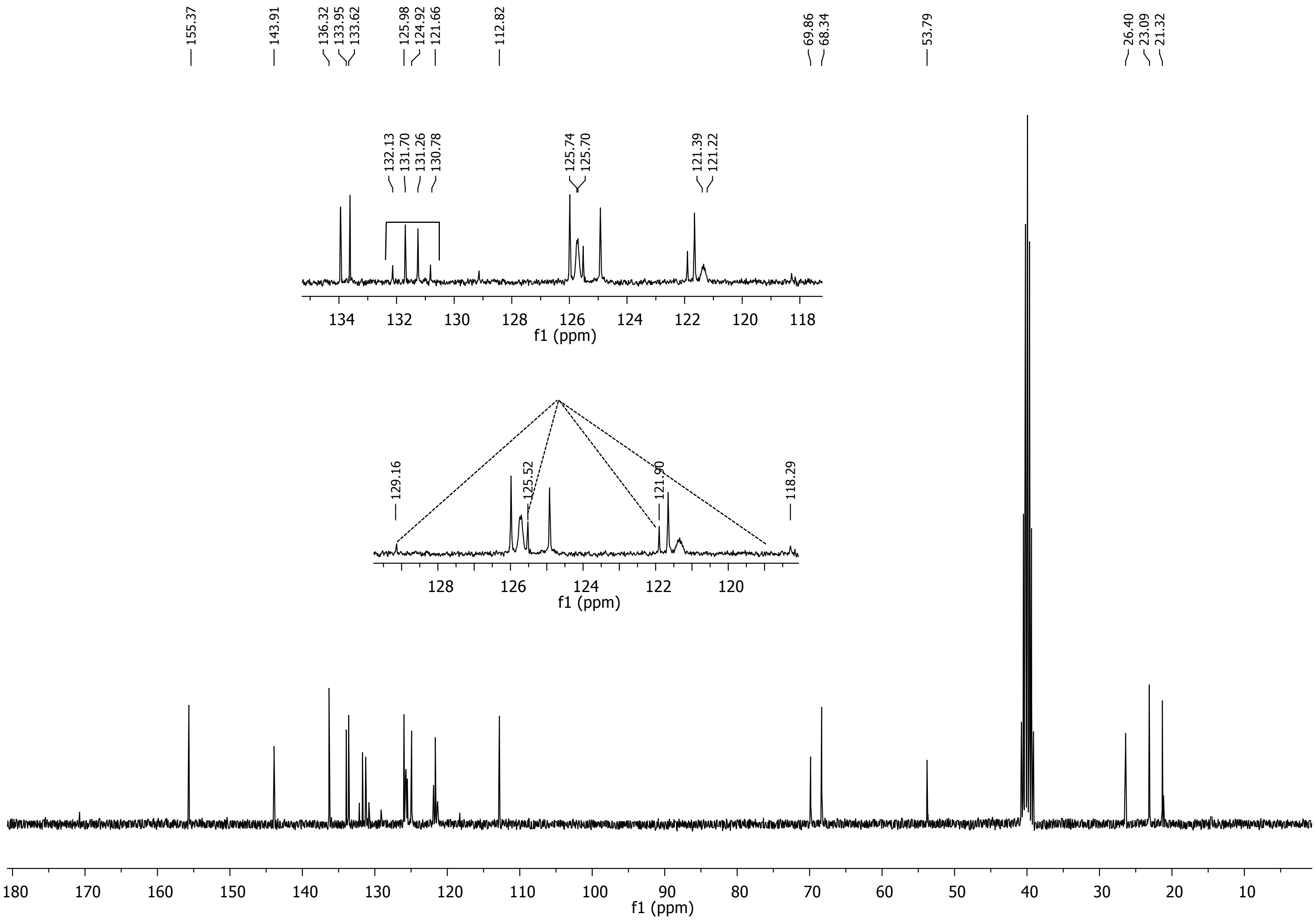

Figure S84. ^{13}C NMR spectrum (75 MHz, DMSO-d_6) of 1-(4-(3,5-bis(trifluoromethyl)phenyl)-1*H*-1,2,3-triazol-1-yl)-3-(2-isopropyl-5-methylphenoxy) propan-2-ol (**3s**).

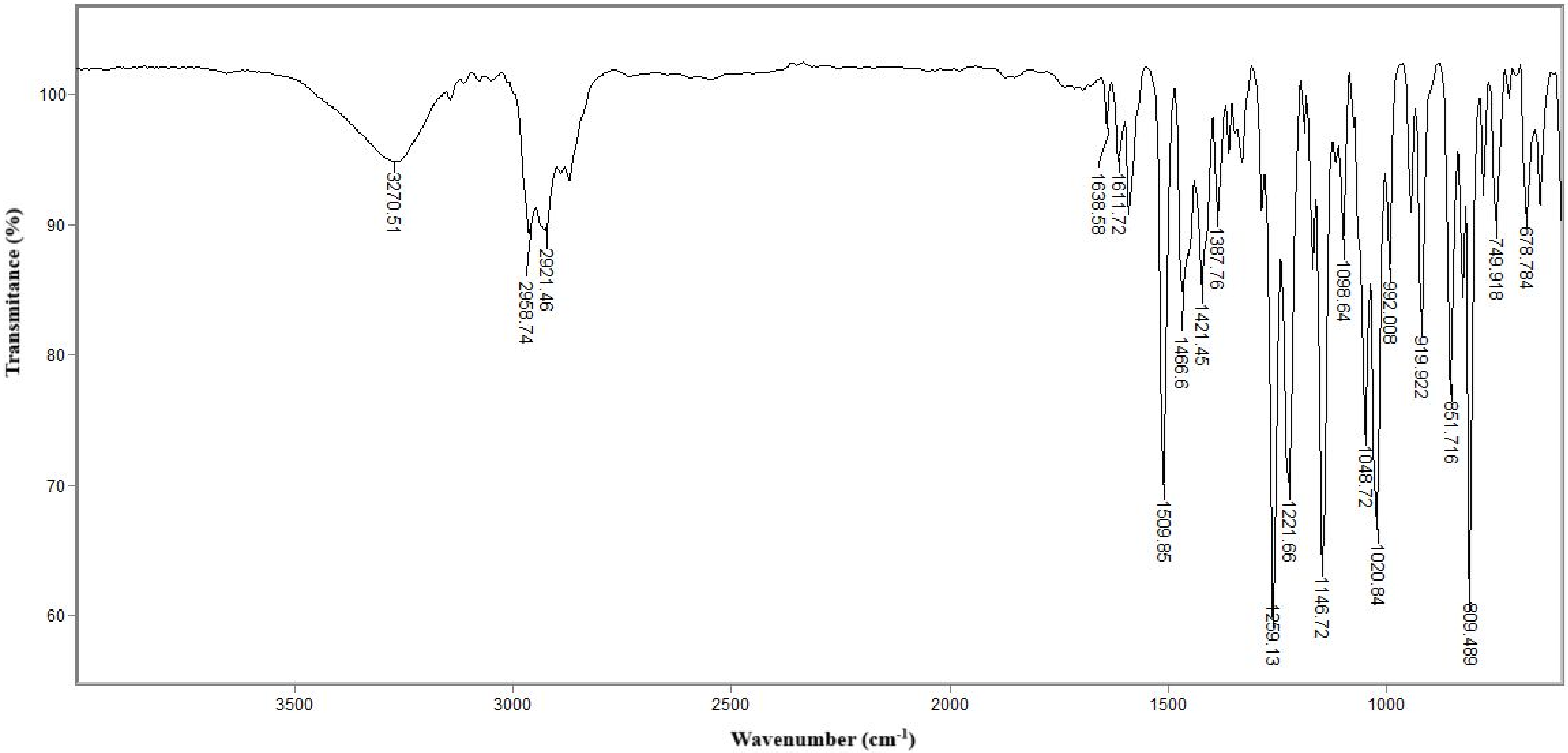

Figure S85. FTIR spectrum (ATR) of 1-(4-((4-allyl-2-methoxyphenoxy)methyl)-1*H*-1,2,3-triazol-1-yl)-3-(2-isopropyl-5-methylphenoxy) propan-2-ol (**3t**).

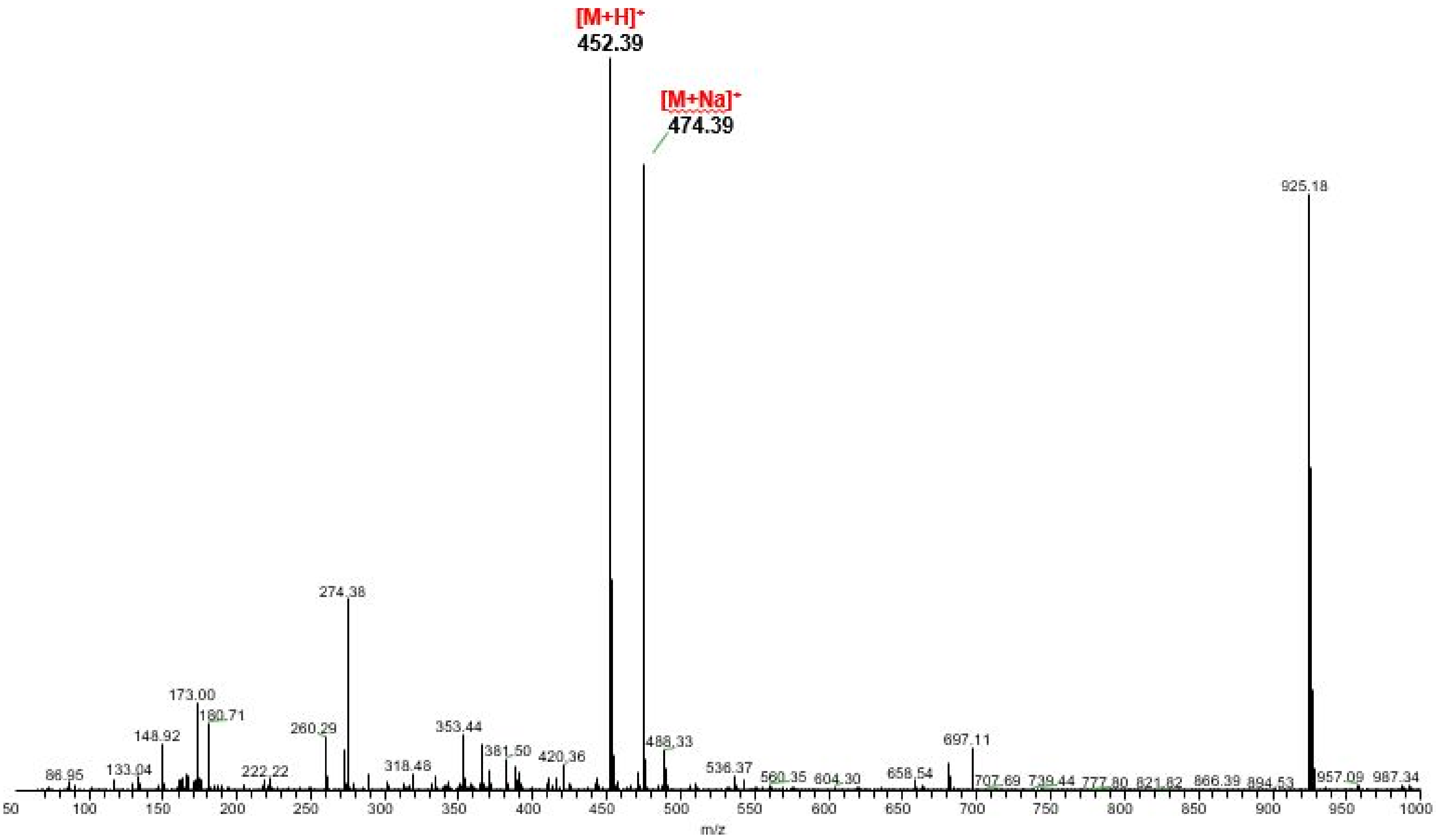

Figure S88. LC-MS spectrum of 1-(4-((4-allyl-2-methoxyphenoxy)methyl)-1*H*-1,2,3-triazol-1-yl)-3-(2-isopropyl-5-methylphenoxy) propan-2-ol (**3t**).

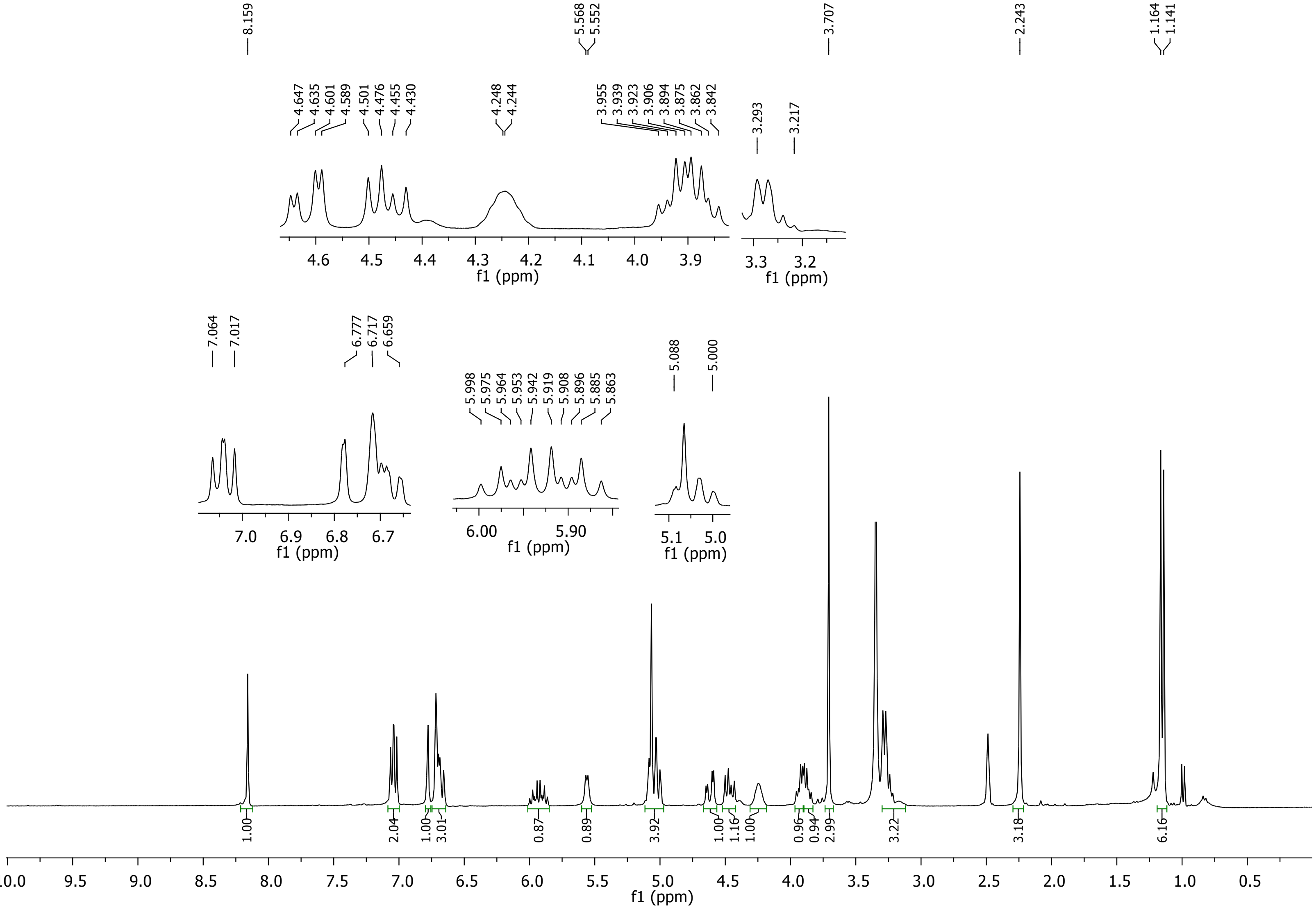

Figure S89. ^{1}H NMR spectrum (300 MHz, DMSO-d_6) of 1-(4-((4-allyl-2-methoxyphenoxy)methyl)-1*H*-1,2,3-triazol-1-yl)-3-(2-isopropyl-5-methylphenoxy) propan-2-ol (**3t**).

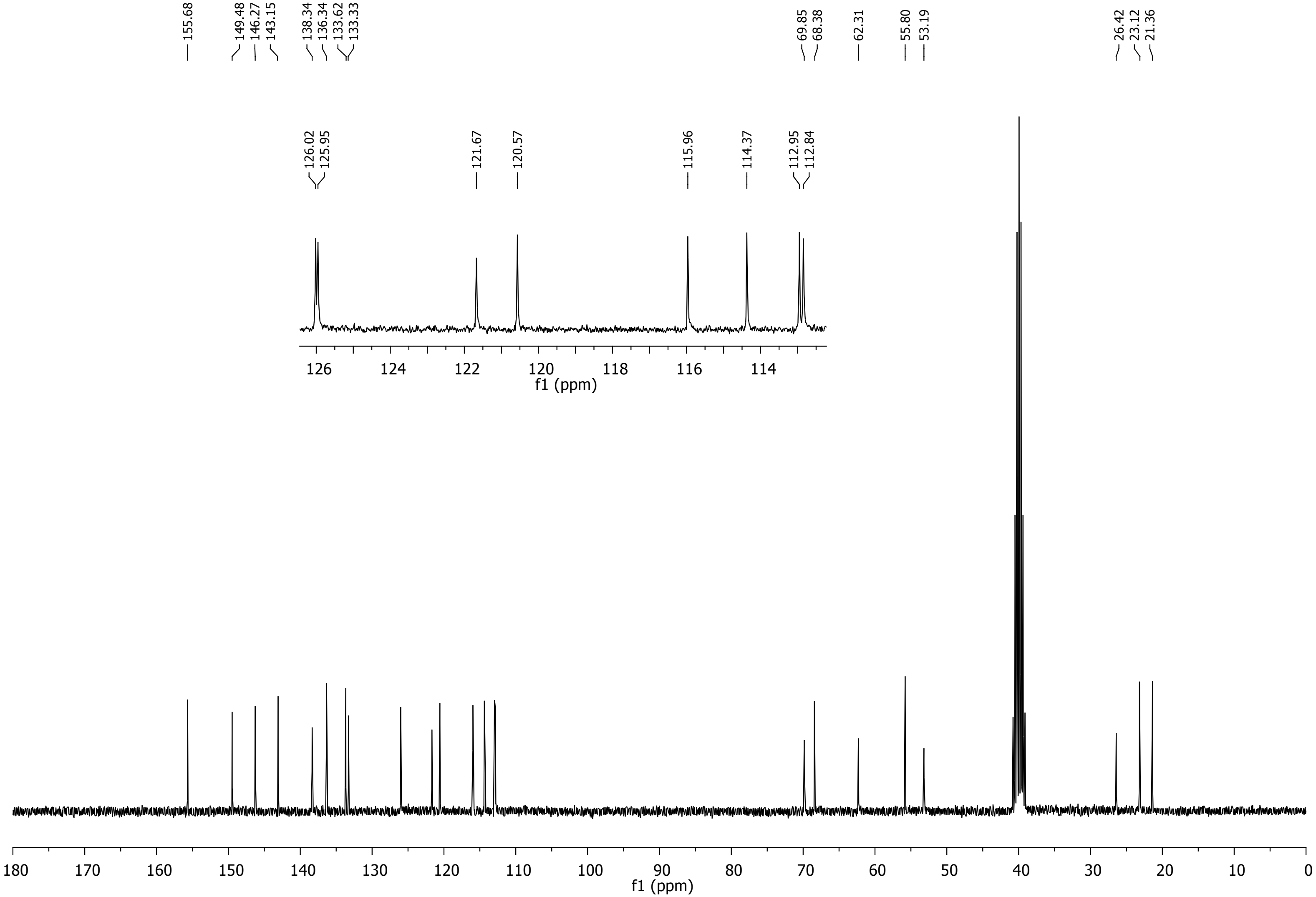

Figure S90. ^{13}C NMR spectrum (75 MHz, DMSO-d_6) of 1-(4-((4-allyl-2-methoxyphenoxy)methyl)-1*H*-1,2,3-triazol-1-yl)-3-(2-isopropyl-5-methylphenoxy) propan-2-ol (**3t**).

Figure S90. ^{13}C NMR spectrum (75 MHz, DMSO-d_6) of 1-(4-((4-allyl-2-methoxyphenoxy)methyl)-1*H*-1,2,3-triazol-1-yl)-3-(2-isopropyl-5-methylphenoxy) propan-2-ol (**3t**).
